# Towards High-throughput Immunomics for Infectious Diseases: Use of Next-generation Peptide Microarrays for Rapid Discovery and Mapping of Antigenic Determinants*[Fn FN2]

**DOI:** 10.1074/mcp.M114.045906

**Published:** 2015-07

**Authors:** Santiago J. Carmona, Morten Nielsen, Claus Schafer-Nielsen, Juan Mucci, Jaime Altcheh, Virginia Balouz, Valeria Tekiel, Alberto C. Frasch, Oscar Campetella, Carlos A. Buscaglia, Fernán Agüero

**Affiliations:** From the ‡Instituto de Investigaciones Biotecnológicas – Instituto Tecnológico de Chascomús, Universidad de San Martín – CONICET, Sede San Martín, B 1650 HMP, San Martín, Buenos Aires, Argentina;; §Center for Biological Sequence Analysis, Department of Systems Biology, Technical University of Denmark, 2800 Lyngby, Denmark;; ¶Schafer-N ApS, 2100 Copenhagen, Denmark;; ‖Servicio de Parasitología y Chagas, Hospital de Niños Ricardo Gutiérrez, Ciudad de Buenos Aires, Argentina

## Abstract

Complete characterization of antibody specificities associated to natural infections is expected to provide a rich source of serologic biomarkers with potential applications in molecular diagnosis, follow-up of chemotherapeutic treatments, and prioritization of targets for vaccine development. Here, we developed a highly-multiplexed platform based on next-generation high-density peptide microarrays to map these specificities in Chagas Disease, an exemplar of a human infectious disease caused by the protozoan *Trypanosoma cruzi.* We designed a high-density peptide microarray containing more than 175,000 overlapping 15mer peptides derived from *T. cruzi* proteins. Peptides were synthesized *in situ* on microarray slides, spanning the complete length of 457 parasite proteins with fully overlapped 15mers (1 residue shift). Screening of these slides with antibodies purified from infected patients and healthy donors demonstrated both a high technical reproducibility as well as epitope mapping consistency when compared with earlier low-throughput technologies. Using a conservative signal threshold to classify positive (reactive) peptides we identified 2,031 disease-specific peptides and 97 novel parasite antigens, effectively doubling the number of known antigens and providing a 10-fold increase in the number of fine mapped antigenic determinants for this disease. Finally, further analysis of the chip data showed that optimizing the amount of sequence overlap of displayed peptides can increase the protein space covered in a single chip by at least ∼threefold without sacrificing sensitivity. In conclusion, we show the power of high-density peptide chips for the discovery of pathogen-specific linear B-cell epitopes from clinical samples, thus setting the stage for high-throughput biomarker discovery screenings and proteome-wide studies of immune responses against pathogens.

Detailed knowledge of antigens and epitopes recognized in the context of naturally acquired human infections has important implications for our understanding of immune system responses against pathogens, and of the immunopathogenesis of infectious diseases. This knowledge is also important for practical clinical applications such as the development of improved vaccines, intervention strategies, and diagnostics.

In the last decades, significant progress has been made in the discovery of antigens and epitopes thanks to a number of methodologies such as cDNA expression libraries (1), combinatorial peptide libraries (2), and peptide and protein microarrays (3, 4). However, current knowledge of the B-cell antigens and the epitope repertoire recognized by the immune system in human infections is still scarce. Indeed, the Immune Epitope Database (5) currently contains an average of only 10 antigens with mapped B-cell epitopes recognized from naturally acquired human infections for bacterial or eukaryotic pathogens. The reasons for this are many, but can be largely attributed to different limitations in the mentioned screening technologies. Heterologous expression of cDNA libraries has been used to guide antigen discovery, but mapping of epitopes most often lags behind as it is a much more costly exercise. Similarly, combinatorial peptide libraries greatly facilitate the identification of peptides that are specifically recognized by antibodies, but these peptides have sequences that can greatly differ from those of the native epitopes (they are mimotopes), thus making it difficult to identify the original antigens. As a result, we currently have only limited detailed information on the fine specificities of the antibody response against complex pathogens.

The number of tools for studying immune responses has recently expanded with the inclusion of peptide and protein microarrays, which have been used to identify pathogen-specific antigens and linear epitopes (6–13). Although whole-protein arrays can successfully identify antigens recognized by antibodies, they present the typical difficulties associated with the production of recombinant proteins in heterologous or *in vitro* systems, do not provide information on the nature and precise location of the epitope(s) in a protein, and are more likely to suffer from nonspecific antibody binding because of the exposure of a large number of potentially antigenic regions. In contrast, peptide arrays can provide exquisite detail of epitope localization, but until now had other limitations mostly associated with their reduced capacity, preventing the complete scanning of large numbers of candidate proteins.

Recent advances in computerized photolithography and photochemistry have led to the development of a novel high-density peptide microarray technology, where individual peptides can be synthesized *in situ* on a glass slide at high densities (14–17). This technology makes the production of high-density peptide arrays highly cost effective compared with previous approaches, while allowing the interrogation of complex immune responses with unprecedented throughput and mapping precision. Previous applications of this technology were limited to the fine mapping of epitopes in single proteins, using monoclonal antibodies, or using immunized animal sera as the source of polyclonal antibodies (16–18).

Using these high-density peptide arrays, we here describe the first large-scale study of fine antibody specificities associated with Chagas Disease, which is an exemplar of a chronic human infectious disease. Chagas Disease, caused by the protozoan *Trypanosoma cruzi,* is an endemic disease of the Americas, affecting ∼8 million people (19). The parasite invades and replicates within host cells, and briefly enters the bloodstream to reach other target tissues. Initially, the disease goes through an acute stage, characterized by patent parasitaemia and the appearance of antibodies against acute-phase antigens, such as SAPA (20), followed by a delayed specific humoral response. In general, the parasite-specific immune response mounted during *T. cruzi* infections is insufficient to completely eradicate the pathogen, leading to chronic infection (19). In this chronic stage circulating parasites are difficult to detect, even by extremely sensitive methods such as PCR. Therefore, detection of antibodies against whole-parasite extracts or defined antigens (21, 22) remains the standard for diagnosis of Chagas Disease.

In this work, we screened high-density microarray slides containing peptides derived from *T. cruzi* proteins with mixtures of immunoglobulins purified directly from blood samples of Chagas Disease patients. This led to the identification of novel antigens and the simultaneous mapping of their linear B-cell epitopes, thus demonstrating the capacity and performance of this platform for studying antibody specificities associated with human infectious diseases.

## EXPERIMENTAL PROCEDURES

### 

#### 

##### Peptide Microarray Content Design

A total of 457 *T. cruzi* protein sequences annotated in the CL-Brener genome (version Jan. 2012) (23) were selected for inclusion in the microarray (according to the groups defined in Table I). Proteins in *Group 1* were randomly sampled from the proteome. If a sampled protein was a putative ortholog of a previously picked protein (as defined by the OrthoMCL algorithm (24)), that protein was skipped. Hence, the final protein set for this group contained no homologous proteins. *Group 2* contained *T. cruzi* proteins without previous serology evidence, selected based on shared features with known antigens such as evidence of expression, subcellular localization, presence of tandem repeats, disordered regions and B-cell epitope predictions, using an integrative method developed in our group (25). Homologous proteins were removed from this group, as described for Group 1. *Group 3* is composed of selected members of the MASP (mucin-associated surface proteins) family. This is a multigene family composed of ∼1300 genes, with a high level of polymorphisms (26, 27), localized at the surface of infective forms of the parasite. This protein family has earlier been proposed to participate in host-parasite interactions (28). The six MASP subgroups (29) were represented in the group. *Group 4* is composed of 68 proteins with previous evidence of seroreactivity in *T. cruzi* infected humans (these were obtained from the IEDB (5) or manually curated from the literature), more information in supplemental Table S3. Protein *Group 5* is composed by 54 neo-proteins of random protein sequences. These random sequences have the same mono- and di-peptide distribution as found in the *T. cruzi* proteome. All unique 15-mer derived from protein sequences in these five groups were included in the array in a single copy, except for a random subset of peptides, which were replicated for technical variability assessment (6549 peptides in two or more copies, 992 in five or more copies). Peptide fields were distributed in 12 microarray sectors of ∼18,700 peptides each. Peptides from the same protein were synthesized at randomized positions within a single sector. The chip contains additional 2386 unique peptides corresponding to eight proteins not accounted within the protein groups defined above (and of which 2 proteins resulted seropositive), including members of the *T. cruzi* TASV protein family (30, 31) and 21 epitopes of high prevalence in healthy humans (not associated to Chagas disease, identified in the IEDB with a search for linear B-cell epitopes from any source with sero-prevalence > = 50%, assayed in at least 20 subjects). The sequences of these 532 proteins (457 *T. cruzi* proteins, 54 neo-proteins and the additional 21 short peptidic epitopes of high prevalence in healthy humans) are available in supplemental Table S4.

##### Derivatization of Synthesis Slides

Synthesis slides (Nexterion P) for the photolithographic synthesis of peptide arrays were purchased from Schott AG, Germany and derivatized with a 2% w/v linear copolymer of N,N′-dimethylacrylamide and aminoethyl methacrylate (both from Sigma-Aldrich) mixed in a 20:1 w/w ratio before polymerization for 2 h at room temperature in freshly degassed 0.1 m sodium borate buffer, pH 8 containing 0.025% v/v TEMED and 0.1% w/v ammonium persulfate (18).

##### Peptide Arrays Synthesis

Each peptide field was composed of 2 × 2 mirrors controlled by the Digital Micro-mirror Device, and with a single-mirror border separation within fields. This setup allows high-throughput while maintaining a high field resolution. The spacer polypeptide DAPAD was added to all peptides at their C-terminal. The FLAG peptide DYKDDDDKK extended c-terminally with a linker peptide (GAPAGAP) was included in the microarray in 852 copies for peptide synthesis quality control and as corner (reference positions) markers. Synthesis of the arrays was performed as described previously (18).

##### Human Sera Samples

*T. cruzi*—infected human sera samples used in this study were obtained from the Laboratorio de Enfermedad de Chagas, Hospital de Niños “Dr. Ricardo Gutierrez” (Buenos Aires, Argentina). All procedures were approved by the institutional review board of this institution. Written informed consent was obtained from all individuals, and all samples were decoded and de-identified before they were provided for research purposes. Sera were collected from clotted blood obtained by venipuncture and analyzed for *T. cruzi*-specific antibodies by commercially available kits: enzyme-linked immunosorbent assay (ELISA) using total parasite homogenate (Wiener lab, Argentina) and indirect hemagglutination (Polychaco, Buenos Aires, Argentina). The negative panel was composed of 10 samples from healthy, non-infected individuals that gave negative results in the mentioned tests. Using these 10 samples, four different *T. cruzi* seronegative sera pools were prepared by taking five random samples (3 μl per serum) in each case, and labeled them A_neg, B_neg, C_neg and D_neg. In the case of Chagas-positive samples, we selected nine samples from patients that had no clinical symptoms, and were classified in the Chagas chronic indeterminate stage (19). Using these nine samples, four different *T. cruzi* seropositive sera pools were prepared by taking five random samples (3 μl per serum) in each case, and labeled them A, B, C, and D. We used the smallest pool size, which would cover most of the high prevalence antibody specificities. For this, we estimated that all epitopes of a prevalence of 50% or higher will be present at least in 1 individual serum with a probability higher than 95%. Briefly, for an epitope of prevalence ***x*** in the infected population, the probability of randomly taking ***n*** subjects without specific antibodies, assuming independence is (1 - ***x***)***^n^***. Then, we calculated the minimum ***x***, such that (1 - ***x***)***^n^*** < 0.05 (*i.e.* that the probability of not sampling an epitope of prevalence ***x***% or higher in ***n*** subjects is less than 0.05). Performing this calculation, we find *n* = 5, *i.e.* (1 - 0.5)^5^ = 0. IgGs were purified from 15 μl sera pools using Melon Gel IgG spin purification kit (Thermo Scientific), following the manufacturer's protocol. Purified IgG samples were checked in 12% SDS-PAGE gels. IgG concentration was estimated after staining by Coomassie Brilliant Blue, by comparison against a standard curve made by electrophoresing different quantities of purified bovine γ-globulin (IgG, 150 kDa, BioRad Laboratories, Hercules, CA).

##### Assays with Peptide Arrays and Data Acquisition

Microarray slides were incubated essentially as described in (16) with a few modifications to accommodate sequential incubations. Briefly, slides were incubated at room temperature overnight with 1 ml of purified pooled IgGs, diluted to 20 μg/ml in 0.15 m Tris/Acetate pH 8.0, 0.1% v/v Tween20. After washing with the incubation buffer, slides were incubated for 2 h with secondary antibody (Cy3 goat anti-human IgG, Abcam Cat. No. Ab97170) at 1 μg/ml. After a second washing step with incubation buffer, followed by air-drying of the slides in a nitrogen jet, the peptide array slides were scanned and recorded with an Innoscan 900, Cambridge, MA laser scanner (INNOPSYS, Carbonne, France) at 1 μm resolution, with an excitation wavelength of 532 nm. The images recorded with the laser scanner were analyzed using the PepArray analysis program (Schafer-N, Copenhagen Denmark). Auxiliary “marker” peptides with sequence DYKDDDKKGAPAGAP containing the FLAG epitope tag, were used for positioning of the grid and to quantify spots' intensities. For all microarray experiments described, the same procedure was performed first with the negative sample (from healthy subjects), and then sequentially with the positive sample (from infected patients) in the same conditions. Therefore two readouts were obtained per slide: the negative sample data and the cumulative signal of negative and positive sample data. A total of 8 microarray chips were assayed, labeled A1 (Sample A_neg followed by Sample A, Replicate 1), A2 (Sample A_neg followed by Sample A, Replicate 2), A3 (Sample A_neg followed by Sample A, Replicate 3), B1 (Sample B_neg followed by Sample B, Replicate 1), B2 (Sample B_neg followed by Sample B, Replicate 2), C1 (Sample C_neg followed by Sample C, Replicate 1), C2 (Sample C_neg followed by Sample C, Replicate 2), and D1 (Sample D_neg followed by Sample D, Replicate 1). Raw intensity data sets for each chip and sample are available in public databases (See Data availability Section).

##### Peptide Mapping, Data Normalization, Signal Smoothing and Negative Subtraction

**Fig. 1.**
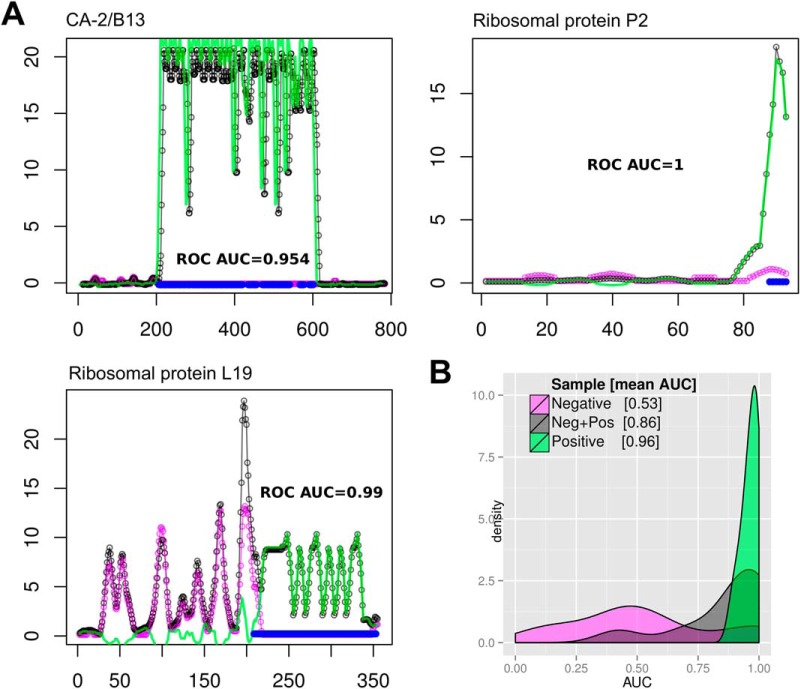
**Epitope Mapping Performance using a single peptide microarray.** *A*, Protein Antigenicity profiles of three validated *T. cruzi* antigens: CA-2/B13, 60 S Ribosomal protein P2 and Ribosomal Protein L19. Horizontal axes show the amino acid positions of the protein sequences; vertical axes show the smoothed and normalized mean intensity values from a single peptide chip (sera pool C, replicate 1). In magenta: signal from negative sample (N). In black: cumulative signal from the negative and positive sample (NP). In green: Chagas-specific signal (subtraction of NP-N). Blue marks near the baseline show the position of known B-cell epitopes (antigenic regions within these antigens), as reported in the literature. *B*, Density plots showing the distribution of area under the ROC curve (AUC) values, where AUC = 1 means perfect residue by residue correspondence of signal with localization of previous known epitopes for 9 validation antigens. Color code for samples is the same as for panel A. Magenta: AUCs centered on 0.5, meaning no predictive performance. Black: average AUC = 0.861, for cumulative negative + positive signal. Green: AUC = 0.96, corresponding to Chagas-specific signal (for the single best performing assay).

In previous successful applications of HD-peptide microarrays (16, 18, 32, 33), minimal data normalization was performed. However, unlike previous work, here we analyzed a complex antibody sample and compared relative intensities across different experiments. This demanded further normalization. A first normalization step was implemented, aimed at equalizing two sequential reads of a single chip (the negative and the cumulated negative+positive data) and decreasing sector (spatial) effects across the chips. For this, we defined an intensity baseline for each sector as its most frequent intensity value (mode). Each peptide was then centered by subtracting its associated baseline value. Once baseline signal was adjusted, intensity dispersion across sequential reads of a single chip was adjusted by dividing each value by the 90th percentile of the intensity values of the 24,000 random peptides in the same assay, therefore bringing the negative and negative+positive cumulated signals into a common scale. Peptide sequences and their fluorescence values were mapped back to the parental proteins sequences (only allowing a perfect sequence match of the 15 residues). The plots display intensity values for individual peptides along the protein sequence (*i.e.* from position 1, to protein length minus 14). We devised a simple smoothing procedure to improve the signal to noise ratio of individual measurements. Smoothing was performed on each protein profile, and consisted in a running median filter of window length 5, followed by a running mean filter of window length 7. Basically, the normalized signal of the peptide at position *i* in the parental protein sequence is first replaced by the median value calculated from the five peptides at positions *i* - 2 to *i* + 2. In a second run, after extreme values had been removed, the value of the peptide at position *i* in the parental protein sequence is now replaced by the mean value calculated from the seven peptides at positions *i* - 3 to *i* + 3. When reaching the C-terminal end, we padded the window length with zeroes to complete the scan. Epitope mapping performance (average AUC, as presented in Results), was incremented by ∼5% after signal smoothing. Smoothed negative sample signal was subtracted from smoothed cumulated negative+positive signal to obtain a smoothed Chagas specific antibody-binding signal, which was plotted as shown in Fig. 1. In many proteins, such as the Ribosomal protein L19 and antigen CA-2/B13 in Fig. 1, a repetitive signal pattern is produced by tandemly repeated (antigenic) amino acid motifs in those protein sequences. Because in each microarray experiment we obtain a single reactivity value for each distinct peptide, if the same peptide has to be mapped to different places in a protein sequence, its associated value would also be repeated, therefore producing such a regular signal pattern. In many cases some of these repeats are not strictly conserved, because of sequence polymorphisms (they are imperfect repeats, but still recognizable as repeat units). This creates an irregular or imperfect repetitive signal pattern for some proteins, usually at the borders of the tandemly repeated motifs. To compare intensity values from different chips and biological samples, an additional step was required to compensate for chip-to-chip intensity distribution disparities. In this case, quantile normalization (34) was performed to force the 3 signal distributions (pre-normalized and smoothed intensity values of negative samples, negative + positive samples and subtracted signals) to be the same across chips/samples. This was done independently for the negative, cumulative and subtracted data sets, using the R package *preprocessCore* (34). The three normalized data sets obtained for each chip after this transformation were used for all subsequent analysis and figures shown in this paper (distributions are shown in supplemental Fig. S3). In the subtracted signal distribution, 1 unit is approximately equivalent to the 95th percentile. The normalized and smoothed values from a total of 8 chips assayed (see above) are publicly available (refer to “Data Availability” section).

##### Epitope Mapping Performance Analysis

For this analysis, we compiled a validation data set of antigens with B-cell epitope mapping data, that met the following criteria: (1) had specific reactivity against chronic *T. cruzi* infected human sera; (2) the mapped epitopes had been reported in at least 2 independent publications; (3) were able to discriminate *T. cruzi* seropositive and seronegative humans in ELISA format using synthetic peptides. From an initial data set of 24 proteins with mapped B-cell epitopes, only nine remained that met this criteria, after removing paralogs and redundant epitopes (peptide sequences and references are listed in supplemental Table S3, column F “High-confidence mapped B-cell Linear Epitopes”). In each antigen sequence, we mapped the exact location of the reported epitope onto the parental sequence. In the case of reported epitopes of length >5 residues, we split the original epitope into smaller “units” of length 5. This extension of the epitope definition was especially relevant in the case of repetitive antigens, where the presence of cross-reactive degenerate repeats (with minor amino acid substitutions) is frequent. Then, we classified as “positive” every 15-mer peptide containing any of these epitopes, and as ‘negative’ the remaining peptides. Receiver operating characteristic (ROC)^1^ analyses were performed for each protein, to assess whether higher chip intensities were associated with epitope localizations. The area under the ROC curve (AUC) was measured for each antigen using the R package ROCR (35). Redundant peptides (identical 15-mers) were excluded from the analysis. The supplemental Fig. S1 shows ROC curves, AUC values, and sensitivities and specificities *versus* cut-off, for each of these 9 antigens, considering individual sera samples or their average. To assess the performance of combined data from different experiments, the normalized and negative sample-subtracted peptides values of different chip/assays were averaged and ROC analysis performed as described.

##### Sensitivity, Positive Predictive Value, and Cut-Off Definition

The cut-off used to define “positive” and “negative” peptide reactivity was set to maximize classification performance measures of sensitivity (true positives/(true positives + false negatives)) and positive predicted values (PPV = true positives/(true positives + false positives)). Positive predictive value is used in this paper in the context of binary classification of antigens and non-antigens, equivalently to classification precision; it should not be confused with classification of subjects based on infection status as frequently used in diagnosis. The validation set of 9 antigens with mapped linear B-cell epitopes of high confidence (Epitopes listed in supplemental Table S3, column F “High-confidence mapped B-cell Linear Epitopes”) was used as true positive (*i.e.* if classification is perfect, these proteins should contain highly reactive peptides and hence be classified as ‘positives’ proteins). The set of 54 neo-proteins (Group 5) was used as negative controls (*i.e.* if classification is perfect, these ‘proteins’ should not contain highly reactive peptides and hence be classified as negative proteins). For this task, the averaged normalized signal from the 8 chips was used. As observed in Fig. 4*C*, it is clear that in the cut-off range around 3 (from 2.6 to 3.4) all positive controls (validated epitopes; dark blue curve) are detected but no negative controls are (random sequences; black curve), *i.e.* both sensitivity/recall and PPV/precision are optimal in this interval. Therefore, a cut-off of 3 was chosen for the averaged signal. Conducting the same analysis on individual samples required a higher cut-off to maintain a PPV of 100% at the expense of sensitivity. In this case, a threshold of seven resulted in a PPV of 100% in all samples and a sensitivity of 0.83 ± 0.11, illustrating the previously described variation between different biological samples.

##### Definition of Antigenic Regions

For simplification, any continuous protein sequence range where all its peptide reactivities were above the cut-off (average signal > 3) was defined as an *antigenic region*. These are shown in supplemental Table S2. Here, we have also included a list of non-redundant antigenic regions (Sheet 2, “unique15mers”) by removing those regions that are identical, in terms of the sequence of their most reactive 15-mer peptide*s (Max pep sequ*ence in supplemental Table S2), to other regions of higher ranks (according to seroreactivity). Finally, an additional filter was applied to remove related antigenic regions (Sheet 3, “unique7mers”). Here, an antigenic region was removed if its most reactive 15-mer had an internal 7-mer sub-sequence matching the most reactive 15-mer of an antigenic region of higher rank.

##### Analysis of Technical Reproducibility and Biological Variability

Variability between identical peptides was assessed from 992 different peptide sequences (a random sample) that were each replicated in at least 5 random positions in the microarray (and up to 25 replicates per peptide). Coefficients of variation associated to these peptides (raw values, ranging from 0 to 255) were calculated per chip, as the standard deviation of intensity divided by mean intensity. Mean intensities were corrected by adding one, to avoid divisions by zero. Because the length of most continuous B-cell epitopes is less than the length of the synthesized peptides, antibody-binding signals are expected to be shared among multiple overlapped peptides (*i.e.* among adjacent peptides covering a common antigenic determinant). The intensity correlation between all pairs of peptides derived from the same protein (but physically located at random microarray positions) was high for highly overlapped peptides (PCC of 0.86 ± 0.02 (S.D.), 14 residue overlap, *i.e.* with single residue shifts) and monotonically decreases for lower sequence overlaps (down to a PCC <0.25 for pairs of peptides with a six residue overlap). This analysis was performed with a subset of 105 reactive and nonrepetitive proteins (*i.e.* excluding those proteins where >20% of sequence is composed of internal tandem repeats, as detected by the TRUST repeat detection method (36)).

**Fig. 2.**
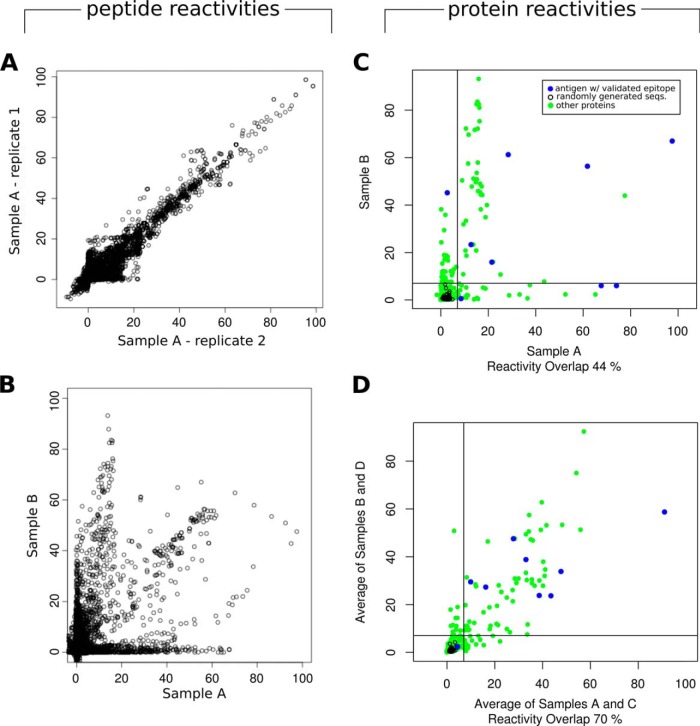
**Technical reproducibility and biological variability.** On the left, scatterplots of normalized peptide reactivity values of (*A*): 2 technical replicates assayed with sample A (Pearson's correlation coefficient, PCC = 0.93), and (*B*) two different biological samples (sera pools A and B, PCC = 0.54). On the right, normalized peptide reactivities were grouped by source protein to calculate protein-wise reactivities, as described under “Experimental Procedures.” In these scatterplots of protein reactivities, biological variability between sera pools at the level of proteins can be observed. In panel *C*, signals from 2 individual sera pools/chip assays were compared, whereas in panel *D*, signals from pairs of pools were averaged before plotting. Green points represent query proteins, black points the randomly generated sequences and blue points, antigens with previously validated epitopes. Vertical and horizontal lines, mark the cut-off, calculated as described under “Experimental Procedures.” Using this cut-off, Sample A and B share 44% of reactive proteins (panel *C*), and the averaged signal from the two sera pools (labeled A and C) share 70% of reactive proteins with the averaged signal from two other pools (labeled B and D, panel *D*).

To assess technical variability across chips, PCC were calculated for all pairs of chips assayed with the same sample (three pairs of sample A replicates, one of sample B replicates and one of sample C replicates). In this case, the signal correlation for the same peptide across different chips, was calculated from normalized and smoothed intensity values of ∼199,000 unique peptides. Correlations of peptide intensities between chips assayed with different samples (biological replicates) were calculated in the same way. In addition, protein-wise inter-sample comparisons were performed. First, each protein was assigned a reactivity value equal to the normalized value of its most reactive peptide. Then, we calculated for each pair of samples, the fraction of proteins that passed the reactivity signal cut-off (a value of seven was previously defined for individual samples) in any of the two samples (S1 OR S2) or in the two samples simultaneously (S1 AND S2). The proportion of shared positive proteins between two samples was calculated as (S1 AND S2)/(S1 OR S2). This is illustrated in the scatterplot in Fig. 2*C*.

##### ELISA Validation of TSSA Antigen

**Fig. 3.**
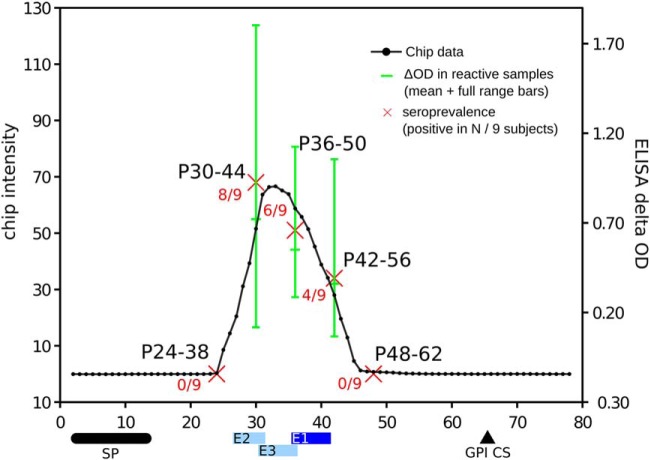
**Antigenicity profile of the TSSA antigen and ELISA validation of HD-Chip data.** *x*-axis represents each 15-mer peptide (from 1–15 to 78–92) and *y*-axis is the averaged normalized and subtracted signal from 8 chip replicates. Five peptides (P24–38, P30–44, P36–50, P42–56 and P48–62) were individually tested against 9 *T. cruzi* positive human sera samples in ELISA format. Red crosses depict seroprevalence of these peptides, ranging from 0% (P24.38 and P48–62) to 89% (P39–44, 8 of 9). Green bars represent full range of ELISA reactivity of these five peptides across different seropositive subjects (absorbance increment above negative controls. Middle mark is the average value). Horizontal bars at the bottom represent the major (E1, blue, from residue 41 to 50) and minor low-reactivity epitopes (light blue: E2, from 30 to 41, and E3, from 36 to 45) as reported previously (42). These bars are placed to cover all 15mer peptides containing the full epitope sequence (for example, E2 is colored by four peptides starting in positions 27–30). Horizontal black bar and triangle in the bottom represent signal peptide and glycophosphatidylinositol anchor cleave site respectively (protein regions absent in its mature form).

The Gluthatione S-transferase (GST)-fusion protein bearing the central and antigenic region of CL Brener TSSA (GST-TSSA-CL) has been described (22). GST-TSSA-CL deletion variants spanning the 15-mer peptides covering the antigen in region of TSSA with offset of six residues (equivalent to peptide-to-peptide overlap of nine residues) were constructed by fill-in of partially complementary forward and reverse oligonucleotides (supplemental Table S5) containing BamHI and EcoRI sites on their 5′ ends, respectively. All of these constructs were treated with restriction enzymes and cloned using the BamHI and EcoRI sites of the pGEX1λT vector (GE Healthcare). Colony-PCR was used for the initial screening of the colonies, which were subsequently confirmed using Sanger-based sequencing. GST-fusion molecules were expressed, purified and quantified as previously described (22). Each GST-TSSA-CL deletion variant (plus a GST and GST-TSSA-CL negative and positive control, respectively) was tested against individual human serum (nine *T. cruzi* positive and three *T. cruzi* negative samples, as described in Human Sera Samples section) by ELISA as described elsewhere (37). The seroprevalence of each peptide was calculated as the proportion of individual sera samples with mean absorbance above the cut-off (*n* = 9). ELISA Δ O.D. (absorbance) in reactive samples, the quantity plotted in Fig. 3 - right axis was calculated for each peptide, as the mean O.D. (3 replicates average) detected in *T. cruzi* positive samples (only if measured OD is above ELISA cut-off), minus the average O.D. measured in negative control samples. Agreement of ELISA *versus* chip data was evaluated by Spearman's rank correlation (*r* = 1, *p* = 0.017) of normalized and subtracted chip data *versus* average absorbance of the nine positive samples, after subtracting average absorbance of three negative sera samples + 5 standard deviations.

##### Data Availability

The array design and raw and processed array data have been deposited in the ArrayExpress database at the European Bioinformatics Institute under accession numbers A-MTAB-526 and E-MTAB-3008, respectively. The complete set of peptides was also submitted to the Immune Epitope Database Resource (5) under submission ID 1000642.

## RESULTS

### 

#### 

##### Design of a High-density Peptide Chip for Screening and Mapping B-cell Epitopes in Chagas Disease

We designed a high-density peptide chip layout containing 225,200 addressable spots, displaying 199,643 unique 15-mer peptides. These peptides were derived from 381 serologically uncharacterized protein sequences derived from the *T. cruzi* proteome, along with 68 *T. cruzi* protein sequences from antigens with previous serological evidence in infected humans (from weak evidence in some cases to very strong evidence including epitope mapping in others), and 54 neo-proteins of random sequence as a negative set used to define the signal baseline in all experiments (see Experimental Procedures for details). In all cases, each complete protein was scanned with a tiling collection of 15-mer peptides overlapped by 14 residues, *i.e.* with an offset of one residue, to achieve maximum resolution. Next, we selected serum samples from Chagas Disease patients or healthy donors, with positive or negative serology for *T. cruzi*, respectively, using routine diagnostic methods, and assayed each chip successively with negative and positive pooled samples, therefore producing two fluorescence readouts. After signal processing and normalization (described below), the subtraction of the negative signal from the final readout produced an informative, disease-specific signal that was characterized further. Negative signal subtraction produced a significant improvement on the epitope mapping performance, see later. We then used the normalized signal for each addressable field in the chip to reconstruct the antibody binding profile for each protein.

A number of artifacts may distort the signal in microarray experiments. To correct for these random distortions, we applied a fast, robust, and simple smoothing procedure on these protein signal profiles. This procedure is based on the knowledge that most linear epitopes are 5–7 residues long (38). Because any two peptides in the array share a 14aa overlap, we expect that specific antibodies will bind several consecutive 15-mers. By using a smoothing procedure based on combined mean and median values in two sliding windows along the reconstructed protein profiles, we removed outliers and other noisy data points while still capturing the important signal patterns in the data. We also applied a global normalization procedure to the data to compare intensity values from different chip experiments and/or multiple readouts in successive assays with a single chip. This procedure allowed us to scale values of each individual chip, and each internal sector within a chip (see Experimental Procedures for details). Signal smoothing, signal normalization, and nonspecific signal subtraction all contributed with an increment in epitope mapping performance.

##### Performance of the HD-Chip Platform: Mapping of Known Chagas Disease Epitopes

We first benchmarked the ability of HD-Chips to fine map and identify a set of previously characterized B-cell epitopes associated with human *T. cruzi* chronic infections. As described above, each peptide chip was assayed sequentially, first with a pool of negative antibody samples (IgGs purified from five individual sera), followed by a pool of Chagas-positive antibody samples (IgGs purified from five individual *T. cruzi* positive sera). The benchmark consisted in correlating residue by residue the chip signal with the location of high confidence B-cell epitopes in nine validated antigens (shown in supplemental Table S3. An excellent epitope mapping performance was obtained, with a mean area under the ROC curve (AUC) for these antigens of 0.964 in the single best performing assay, and with a global average AUC of 0.912 ± 0.054, obtained by averaging the AUC measured for these antigens in all chip replicates (using 4 different sera pools, labeled A, B, C, and D, see Experimental Procedures). Fig. 1*A* shows examples of the antigenicity profiles obtained for three of the validated antigens together with the location of previously known B-cell epitopes and their corresponding AUC performance measures. Fig. 1*B*, summarizes the epitope mapping performance in all the 9 validated antigens. Reactivity data obtained from the negative samples (in magenta in Fig. 1) have zero epitope mapping capacity, with a mean AUC centered around 0.5, whereas data from the positive samples show an excellent performance with a mean AUC close to 1 (Fig. 1 in gray or green, signals before or after negative-signal subtraction, respectively). Epitope mapping performance was significantly improved by subtraction of negative signal. Antigens' AUC values were significantly incremented after negative signal subtraction, from an average AUC of 0.948 before subtraction to an average AUC of 0.972 after subtraction (*p* < 0.05, paired Wilcoxon signed rank, *n* = 9). supplemental Fig. S1 shows the antigenicity profiles of all known Chagas antigens with their validated epitopes, as measured with each biological sample (A, B, C, and D) and their average across all experiments.

##### High Technical Reproducibility Expedites Studies on the Diversity of B-Cell Responses Using HD-Chips

We next assessed the technical reproducibility of HD-Chips. For this, we analyzed the signal of identical peptides replicated at random locations on the chip (992 different peptide sequences, ranging from five to 25 copies each). These showed consistent intensities, with a mean coefficient of variation (*i.e.* standard deviation/mean) of 0.19 (± 0.05, in 8 replicates using four different biological samples). We then analyzed and compared the signal obtained from chips produced and assayed with the same biological samples (technical replicates). These showed a very high reproducibility, with peptide normalized values (n ∼199,000) across technical replicates showing a Pearson Coefficient Correlation (PCC) of 0.9 ± 0.032 (over the five different technical replicates described under “Experimental Procedures”, see Fig. 2*A*).

Given this high technical reproducibility, we proceeded to compare the antibody-binding signals across different biological samples. As expected, when comparing complete chip data assayed with purified IgGs from different sera pools, a much larger variability was observed. As shown in Fig. 2*B* for two pools, only a subset of peptides were reactive in both samples, and many peptides were reactive against one of the samples but not the other (with an overall correlation (PCC) of 0.558 ± 0.138, over the six different combinations of biological samples described under “Experimental Procedures”). When we considered the antibody-binding reactivity at the level of full-length proteins, by assigning to each protein a single score corresponding to the peptide with the highest signal within the protein sequence, a similar pattern of diversity was observed. Two sera pools shared on average 52% ± 10.6% of the positive antigens (Fig. 2*C*; serologically positive was defined above a threshold explained in the next section).

When comparing antibody specificities from different positive sera pools, it was expected that the pool-to-pool variability would decrease when increasing the size of those pools (*i.e.* effectively assaying a larger number of sera). Indeed, by adding signals from HD-Chips assayed with two sera pools into a larger virtual pool, two pools shared up to 70% of the positives antigens (Fig. 2*D*). Moreover, combination of data from different pools also increased the disease-specific signal allowing better separation of antigens from non-antigens. This can be seen in Fig. 2*D*, where positive controls (blue points) are clearly separated from the negative controls (black points). As shown in Fig. 2*C*, the relative signal for each antigen is not fully conserved across chip replicates assayed with separate biological samples. Every sample provided additional antigenicity information, and combining signal from two or more biological replicates led to an increased performance for mapping known epitopes. When considering pairs of chips assayed with different biological samples (using their averaged signal), we observed an increment of ∼6% in the AUCs (from a mean of 0.912 to a mean of 0.964). Moreover, the epitope mapping performance was further improved by increasing the number of biological replicates to three (averaging the signal from three assays with different pools of sera) to a mean AUC of 0.972. Adding a fourth biological replica, did not lead to further increases in the AUC value (data not shown). In contrast, technical replicates (averaging signal from two chips assayed with identical biological samples) produced only a small increment (0.6% AUC increment on average) in epitope mapping performance.

##### High Resolution Mapping of Reactive Epitopes

When analyzing the epitope mapping performance for previously described antigens, we noticed a unique case that displayed a highly antigenic region that did not agree perfectly with the previously described epitopes. This antigen, the Trypomastigote Small Surface Antigen (TSSA), is used for serological discrimination of *T. cruzi* lineages (39, 40). Di Noia *et al.* performed a preliminary epitope mapping of the TSSA protein identifying one major epitope (41-KPATGEAPSQ-50) along with two minor (weak signal and low prevalence) epitopes (30-TSSTPPSGTEN-41 and 36-SGTENKPATG-45) (39). This study was carried out with a limited number of short peptides of varying lengths (8–11 residues), and with a relatively large offset (five residues) for some key peptides. Here, we scanned the full-length of the TSSA protein at maximal resolution (one residue offset), detecting a single, broad antigenic region spanning residues 24 to 57, with a maximum signal located between residues 30 and 46 (see Fig. 3). So although the location of the identified central antigenic region agrees between the two studies, the location of the peak of the antigenic signal differs. To further investigate this discrepancy and further study the seroprevalence of individual responses to TSSA peptides, we re-assayed five overlapping peptides from this antigenic region in an ELISA format assay using 9 individual (not pooled) human sera samples. Data from the peptide chip and from the ELISA assays are provided in supplemental Table S1. The antigenicity profile of the TSSA antigen, as obtained from the HD-Chips is depicted in Fig. 3 along with the signal from the five individual peptides (P24–38, P30–44, P36–50, P42–56, and P48–62) assayed in ELISA format. The correlation of both seroprevalence (fraction of the nine donors that responded to each peptides) and seroreactivity (average ELISA signal of each the positive individual donors to each peptide) with the chip intensity values is evident, strongly suggesting that the dominant TSSA epitope is located in the region P30–46, and not in the region P41–50 as reported previously. This example shows the high accuracy of HD-Chips to map linear epitopes, which can be exploited to refine previous epitopes where mapping might be imprecise because of technological or cost issues resulting in poor mapping resolution (insufficient number of peptides, or low peptide overlap).

##### Discovery and Fine Mapping of Novel Antigenic Determinants

We next applied these HD-Chips to the identification of novel antigens and antigenic determinants (new candidate diagnostic markers). For this, we analyzed the seroreactivity of 175,566 distinct 15-mer peptides, covering 457 proteins at maximum resolution from the *T. cruzi* CL-Brener proteome. These peptides cover ∼3% of the ∼6.3 million 15-mer unique peptides of this proteome. Protein sets were defined as follows (for details see the section on “Experimental Procedures”): *Group 1*: 50 proteins randomly selected from the proteome (serologically uncharacterized); *Group 2*: 99 proteins prioritized by a multi-feature integrative bioinformatics strategy (25) (serologically uncharacterized); *Group 3*: 232 serologically uncharacterized members of the MASP family of surface proteins (41); *Group 4*: 68 proteins with previous evidence of seroreactivity in *T. cruzi* infected humans (both with and without previously mapped epitopes). As mentioned (see “Experimental Procedures”), we also included 54 neo-proteins of random sequence (*Group 5*) to measure the background distribution of antibody-binding signal in the chips, and 8 additional parasite proteins not belonging to any of the other groups. Peptides included in this HD-Chagas-Chip design were derived from proteins in all these groups by taking all possible 15-mer subsequences.

To establish a reactivity threshold to classify proteins and peptides as ‘positive’ (reactive) or ‘negative’ (not reactive), we analyzed the sensitivity for each protein group (fraction of identified proteins) as a function of decreasing chip-signal score. In this analysis, each protein was assigned a single score corresponding to the peptide with the highest signal within the protein sequence. The analysis included 8 replicates for each protein sequence (one from each of the eight-chip assays performed). The signal from each chip was processed as described earlier to reduce noise and allow for standardization between chips, and the final signal reported is the average over the 8 replicates.

**Fig. 4.**
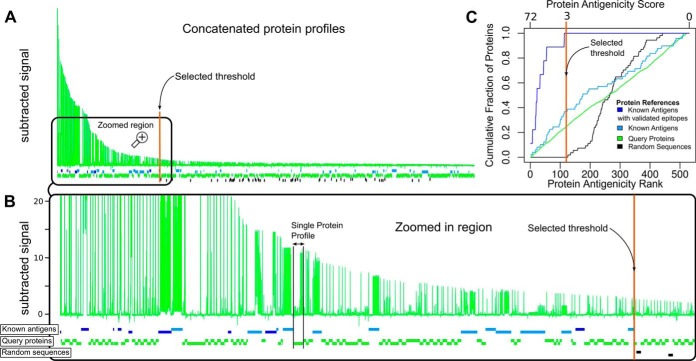
**Global visualization of antigen and epitope discovery.** *A*, Concatenation of all protein profiles (similar to those in Fig. 1) ordered from left to right according to the peptide with the highest signal in each protein. The *x* axis shows the residue position for all concatenated proteins (full length including their antigenic and non-reactive regions), and the *y* axis represents the averaged, normalized and subtracted reactivity values (Chagas-specific signal). *B,* Enlarged region showing the antigenicity profiles of proteins that lie to the left of the selected threshold value of 3 (colored vertical line). Horizontal boxes intercalated below the concatenated profiles represent individual proteins. Blue boxes represent antigens with previously validated linear epitopes. Cyan boxes are proteins with previous serology evidence (Protein Group 4) but without known linear epitopes. Black boxes correspond to random sequences (Group 5). Green boxes represent serologically uncharacterized (‘query’) *T. cruzi* proteins (Groups 1, 2 and 3). *C*, Enrichment of different groups of proteins along the ranking. Bottom *x*-axis: antigenicity rank, with the most antigenic protein (according to the highest scoring peptide) at the left. Top *x*-axis: protein antigenicity score. In the *y*-axis, the cumulated fraction of proteins at each ranking position, for the different protein group.

Using these data, we generated an antigenicity profile for each protein (like the ones depicted in Fig. 1). Next, we sorted from high to low these profiles on overall antigenicity score (signal from the highest scoring peptide contained within the given protein). The result of this analysis is shown in Fig. 4 (panels *A* and *B*). From this figure, separation of the positive controls (the 9 antigens with high-confidence epitopes) from the negative controls (random neo-proteins) is evident, whereas the serologically uncharacterized proteins were distributed along the full reactivity range. Using a reactivity threshold of 3 (Fig. 4*C*) we can identify all positive controls (100% sensitivity) and no negative controls, corresponding to a specificity and predicted positive value (PPV) of 100% (equivalent to 0% false positives). Applying this cut-off to the complete data set, we detected 2031 Chagas-specific positive peptides in 122 distinct proteins.

Using this threshold, around 1% of all peptides were hence found positive with substantial positivity rate differences across protein groups. Group 4 had the highest positivity rate with ∼2.62% of its peptides being positive, followed by Group 3 of MASP family proteins with 1.21% of positive peptides, followed by Group 2 containing peptides from prioritized proteins with a rate of 0.84%; and finally Group 1 (randomly selected proteins) with a 0.13% rate. Noteworthy, our integrative multi-feature prioritization strategy (25) produced a ∼sixfold increment in the peptide reactivity rate compared with selecting proteins at random (group 2 *versus* group 1). Detailed reactivity rates for each protein group are summarized in Table I, and protein profiles for all positive proteins are shown in supplemental Fig. S2.

**Table I:** Chagas-specific positivity rates in different peptide sets. The table shows the total number of distinct peptides assayed in each group; the number of positive peptides (above cutoff of 3) in the averaged signal (combining data from eight peptide-chip assays); the average number of positive peptides in a single experiment (single chip/single sample above cut-off of 7); and the number of peptides that were positive in at least 1 out of the 4 sera samples (pools) tested. Percentages are given in parentheses. Group 5 of 24,000 randomly generated peptides (negative control) had no positives in any case

Protein Set	Description	No. of Peptides	Positive Peptides	
			Average signal in all chips	In single chip
Group 1	Proteins picked randomly from the proteome	38,664	50 (0.13%)	23.5 ± 17.2
Group 2	Proteins ranked with a bioinformatics method	37,773	317 (0.84%)	133.25 ± 38.54
Group 3	Proteins from MASP family	65,808	797 (1.21%)	399.75 ± 219.3
Group 4	Proteins with previous seroreactivity evidence	32,372	848 (2.62%)	484.75 ± 76.3

Next, to further delineate antigenic portions of the analyzed proteins we defined any group of consecutive reactive peptides located in a specific region of an antigen as an ‘antigenic region’ (see “Experimental Procedures”). These regions are defined by peaks in the antibody-binding signal observed from the arrays and contain one or more B-cell epitopes. The 2031 positive peptides detected were mapped to 187 distinct antigenic regions. After removal of peptides from known *T. cruzi* antigens, the remaining 1164 peptides (121 unique regions, which map to 97 novel *T. cruzi* antigens), represent new peptidic markers for Chagas Disease, for which this report represents their first serological characterization. Furthermore, from the 24 positive proteins with prior serologic evidence (Group 4), we detected new antigenic regions (not reported previously) in 10 of them (shown in supplemental Table S2). Protein reactivity rates for each protein group are summarized in Table II. Briefly, 35% of proteins in Group 4 were Positive, followed by a 30% of Group 3 (MASPs), followed by a 21% of prioritized protein (Group 2) and a 10% of randomly picked proteins (Group 1).

**Table II:** Chagas-specific positivity rates in different protein sets. The table shows the total number of distinct proteins assayed; the number of proteins with any specific signal peak above the specified cutoff considering the averaged signal (combining data from eight peptide-chip assays); the average number of positive proteins in a single experiment (single chip\sample); and the number of proteins that displayed a positive signal (above cutoff) in any of the four sera samples tested. Percentages are given in parentheses. Group 5 of randomly generated 54 neo proteins (negative controls) had no positives in any case

Protein Set	Description	Number	Positive Proteins	
			Average signal in all chips	In a single chip
Group 1	Proteins picked randomly from the proteome	50	5 (10%)	3 ± 2.1
Group 2	Proteins ranked with a bioinformatics method	99	21 (21.21%)	10 ± 1.4
Group 3	Proteins from MASP family	232	70 (30.17%)	46.25 ± 11.6
Group 4	Proteins with previous seroreactivity evidence	68	24 (35.29%)	16.5 ± 2.9

In summary, our study is providing detailed information on the linear antigenic determinants for both novel antigens, and for a subset of antigens that have not been characterized in detail until now. A table with all antigenic regions along with their source protein, localization, sequence and reactivity data is available as supplemental Table S2.

It is worth mentioning that prior to this work, the number of known *T. cruzi* antigens recognized from human infections was ∼68, and the number of those with mapped epitopes were 20 (see supplemental Table S3). Therefore, despite the fact that our HD-Chip contained a still low representation of the *T. cruzi* proteome (∼3% of the proteome was displayed in our design), we were able to increase the coverage of known antigens for this pathogen more than twofold, and more than 10-fold in the case of finely mapped antigenic regions, essentially providing the majority of currently known epitopes for this disease.

## DISCUSSION

This study provides so far the largest number of fine antibody specificities simultaneously measured for a human infection, with more than a 10-fold increment in throughput when compared with previous screenings using peptide microarrays (7, 13, 42, 43). With only 20 μg of purified immunoglobulin directly obtained from human clinical samples, these high-density peptide microarrays allowed the recognition of specificities against ∼180,000 distinct pathogen-specific 15-mer peptides. Moreover, a sequential incubation protocol allowed subtraction of signal from healthy/non-infected human samples to obtain infection-specific signal in each microarray slide.

We benchmarked the precision of our measurements using previously known antigens with mapped B-cell linear epitopes. These experiments showed that HD-Chips have an excellent epitope mapping performance, with AUCs values >0.96 in a single microarray and an average AUC of 0.972 after combining data from multiple samples. Further analysis showed that the deviation from a perfect AUC of 1 was only because of single-residue differences at the boundaries of the previously reported B-cell epitopes, particularly in the case of antigens with multiple repetitive epitopes. An independent validation in ELISA format of the TSSA antigen profile further supported the mapping precision of HD-Chips. In fact, we showed in this case that the major linear epitope within this antigen is located between residues 30 and 46 and not between residues 41 to 50 as detected using a less accurate technique (39).

The set of 15-mer peptides derived from randomly generated protein sequences was essential to define a nonspecific antibody-binding baseline distribution, and to normalize the data, allowing the subtraction of signal from incubations with negative samples, and bringing the data from different microarray replicates into a common scale. These data was also used to guide the selection of a reasonable threshold for classification of reactive peptides. Using this threshold, more than 2000 peptides, *i.e.* ∼1% of all screened 15-mer peptides, resulted seropositive in *T. cruzi* infected human sera pools and negative in the non-infected sera pools, with virtually no false positives (with the reactivity of all 24,000 randomly generated peptides below the cut-off). Although this threshold was defined based on the comparison against the reactivity of random peptides, it is already well established that both combinatorial libraries in phages (1), or random peptides in high-density arrays (43, 44) can detect significant and reproducible binding by mimicking natural epitopes. Therefore, although we chose a very conservative threshold to identify the most reactive peptides, lowering this threshold can certainly provide additional Chagas-specific biomarkers.

In our benchmark, epitope mapping performance was enhanced by subtracting reactivity signals measured in healthy donor samples, *i.e.* not related to the infection of interest, and perhaps caused by exposure of patients to other infectious agents or antigens with cross-reacting epitopes. Moreover, with this experimental setup the two samples were compared on the same physical spots, hence reducing the number of required chips by half and suppressing the variability associated with peptide synthesis and slide manipulation.

Based on the presented evidence it is clear that these HD-Chips are highly suitable for exhaustive mapping of the specificities of B-cell responses against human pathogens. Interestingly, antibody-binding to specific pathogen protein regions was also detected when using sera pools from healthy individuals (see for example the N-terminal region of the Ribosomal protein L19 in Fig. 1*A*, additional cases are also evident in supplemental Fig. S2). These antigenic determinants may be shared with other pathogens to which the donor has been exposed, or may represent cross-reactive determinants to other antigens (perhaps from vaccination antigens). In any case, the high-resolution scanning of antibody-binding across the full length of proteins is able to precisely map these regions, providing essential information for improving the specificity of recombinant antigens used in diagnostic applications.

In this context, it is interesting to compare the overall rate of antigen discovery provided by peptide and whole-protein arrays. Previous studies have profiled humoral immune responses in infected humans compared with healthy individuals for several pathogens using protein microarrays (45). Some of the largest studies were performed for *Mycobacterium tuberculosis* (∼4000 proteins, representing almost the full proteome; with a 10% of seroreactive proteins) (8), *Leptospira interrogans* (∼3300 proteins, representing almost full proteome; with ∼5% of seroreactive proteins) (46), *Brucella melitensis* (∼3000 proteins, representing almost full proteome, with a ∼4% of seroreactive proteins) (47), and *Plasmodium falciparum* (∼1200 proteins or ∼23% of its proteome, with ∼13% of seroreactive proteins) (9). In our experiments we obtained a ∼1% global peptide positivity rate (∼2,031 positive out of 175,566 unique *T. cruzi* derived 15-mer peptides) when averaging signal from all HD-Chip experiments. This rate, goes down to ∼0.5% if we consider a non-redundant set of proteins with no prior serological characterization. In terms of full-length proteins, this corresponds to a ∼15% of the parasite proteins being targeted by the humoral immune response in infected subjects. Despite the fact that peptide microarrays are limited to identify linear epitopes, the estimated proportion of seroreactive proteins observed here for Chagas Disease is in line and even above those described in the aforementioned screenings using whole protein microarrays in other pathogens.

Therefore, we believe that high-density peptide microarrays offer an excellent platform for whole proteome screenings. In scenarios where the peptide space to screen is still too large, a combination of high-coverage whole-protein arrays followed by a detailed epitope scanning using high-density peptide chips could provide an excellent strategy to analyze antibody responses against pathogens. In this strategy, protein arrays would streamline the process of antigen identification, and HD peptide chips would precisely map the antigenic determinants in these antigens.

Besides the obvious practical impact of these large scale antigen screenings on the development of improved serodiagnostics and vaccines, unbiased and exhaustive studies of humoral immune response specificities in human infectious diseases are needed for a better understanding of pathogen immunogenicity and immunodominance, and to improve current antigenicity prediction tools (48, 49). In our study, we successfully detected all antigenic determinants in high-confidence antigens with mapped B-cell epitopes. We were also able to discover and map the locations of epitopes for previously described antigens that lacked detailed B-cell epitope mappings. However, we failed to detect linear antigenic determinants for some of the proteins with previous serological evidence. Among other likely explanations, these antigens may contain only discontinuous, or low prevalence epitopes. Low prevalence of epitopes may be explained by the genetic heterogeneity of infecting parasites (50). Future studies using diverse serum samples (*e.g.* from diverse geographic origins, or from patients displaying different clinical manifestations of the disease) will help to answer this. We also note that the only epitopes that are readily mimicked by synthetic peptides are continuous epitopes. Discontinuous epitopes, which are made up of residues from separate stretches of the antigen polypeptide chain, can only be identified by x-ray crystallography of antigen-antibody complexes. However, we believe this limitation is largely balanced by the throughput and flexibility offered by high-density peptide microarrays.

For many of these screenings seeking to identify antigens or defined antigenic determinants, a number of bioinformatic strategies based on defined protein features have been applied to narrow down the set of candidates displayed in the arrays (25, 51–53). We have previously developed one such strategy to prioritize *T. cruzi* proteins and peptides for use in a spotted peptide microarray (25). In this work we found a significant ∼6 fold increment in the number of seroreactive peptides in this ranked set when compared with a random pick, thus supporting the utility of such approaches.

In these experiments, we used a HD-Chip design were we simultaneously measured antibody-binding to >200,000 addressable spots in each slide. However, larger capacities (currently up to two million addressable spots) are possible by using a single mirror per peptide field. In this case, the larger capacity comes at the price of smaller fields and inherent larger measurement error. However, even using larger peptides fields, the screened protein space can be increased significantly by decreasing the sequence overlap between adjacent peptides in a protein sequence. The high signal correlation observed from peptides that are contiguous in the protein sequence suggested that it could be possible to further reduce the number of peptides required to completely scan each protein. In *a posteriori* computational experiments in which we re-analyzed the data simulating different peptide overlaps, we observed no significant epitope mapping performance loss down to an overlap of 12 residues between two 15-mers (*i.e.* a 3 residue shift when scanning a protein sequence) (not shown). This means that we should be able to achieve a ∼threefold increase in the protein search space using the same number of peptides per slide. Considering a large proteome such as that of *T. cruzi,* 33 HD-Chips would be required to screen the ∼6 million 15-mers in which this proteome can be broken down (200K peptide fields per chip, scanning proteins at maximal resolution). However, by scanning proteins with a 12-residue overlap and pushing the microarray density up to 500K peptides per slide, the complete proteome could be covered with only four HD-Chips, without sacrificing significant sensitivity.

In conclusion, by taking advantage of next-generation peptide arrays, we show that by screening ∼3% a large eukaryotic proteome we discovered and finely mapped more than 120 new antigenic determinants, providing essentially most of the linear B-cell epitopes currently known for this infectious disease. Our results show that it is now feasible to increase the pace of biomarker discovery for infectious diseases, and to further increase the scale and detail in the study of B-cell immune responses against human infectious diseases.

## Supplementary Material

Supplemental Data

## Footnotes

^1^ The abbreviations used are:

HD-Chips: high-density peptide chips
ROC: Receiver Operating Characteristic
AUC: Area under the ROC curve
PPV: Positive-Predictive Value
PCC: Pearson product-moment Correlation Coefficient.
