# Supplementary material for: Towards High-throughput Immunomics for Infectious Diseases: Use of Next-generation Peptide Microarrays for Rapid Discovery and Mapping of Antigenic Determinants
Source: Mol Cell Proteomics. 2015 Jul;14(7):1871–84. doi: 10.1074/mcp.M114.045906 (PMC4587317; doi:10.1074/mcp.M114.045906)
Supplement: Supplemental Data [file supp_14_7_1871__index.html]

Towards high-throughput immunomics for infectious diseases: use of next-generation peptide microarrays for rapid discovery and mapping of antigenic determinants — Towards High-throughput Immunomics for Infectious Diseases: Use of Next-generation Peptide Microarrays for Rapid Discovery and Mapping of Antigenic Determinants — Towards Chagas Disease Immunomics with HD Peptide Arrays — Supplemental Data 

# Towards High-throughput Immunomics for Infectious Diseases: Use of Next-generation Peptide Microarrays for Rapid Discovery and Mapping of Antigenic Determinants

## Supplemental Data

**Files in this Data Supplement:**

- Supplemental Figure 3 - Boxplots of normalized data. The plots in the figure show the distribution of intensity values after normalization of readouts from the incubation with negative sample (top), cumulative readouts after incubation with negative followed by positive sample (middle) and positive, disease-specific values (after subtraction of negative signal, bottom). Boxplots correspond to each microarray (from left to right: A1, A2, A3, B1, B2, C1, C2 and D1). Figures on the right side show the same boxplots but zooming in the bulk of the data.
- Supplemental Figure 1 - Supplementary Figure 1. Epitope mapping performance. The figure shows antigenicity profile plots for all antigens used for epitope mapping performance assessment. In these plots, the antibody-binding signal for each protein was reconstructed based on the array data, as explained in the text. The plots also show the location of previously known epitopes (blue marks at the bottom of the plot frame). After each antibody-binding profile, we provide an assessment of epitope mapping performance (ROC curves) for each antigen. The zip file contains 5 PDFs, one for each sample (sera pools A to D) and one displaying the averaged signal from all samples.
- Supplemental Figure 2 - Supplementary Figure 2. Antigenicity Profiles of Positive Proteins. The figure displays plots of normalized reactivity values along sequences for all proteins with at least one peptide above the selected cut-off (see main text). The zip file contains two PDF files: proteinProfilesPositiveProteins.Average.pdf containing antigenicity profiles showing the averaged signal for all chips and samples and the range of reactivity values corresponding to the 4 sera pools A, B, C and D; proteinProfilesPositiveProteins.IndividualSamples.pdf containing antigenicity profiles showing the antibody-binding signal obtained from each sera pool in a separate color.
- Supplemental Table 1 - Supplementary Table 1. ELISA validation of the antibody-binding profile of the TSSA antigen. Sheet 1 shows the complete results from the ELISA assays performed on different GST-fusions containing TSSA-derived peptides (shown in Supplementary Table 5). Sheet 2 shows normalized intensity values of TSSA (locus tag Tc00.1047053507511.81) from peptide microarrays of: 1) negative sample, 2) cumulated negative + positive sample and 3) positive (negative subtracted) for the 8 replicates (sample A replicate 1, sample A replicate 2, etc.), average values per sample (A.avg, B.avg, C.avg), global average (ABCD.avg) and prevalence (proportion of positive samples).
- Supplemental Table 2 - Supplementary Table 2. Antigenic Regions (Excel Spreadsheet). Table listing antigenic regions, their positions in the proteins, and the recorded antibody-binding signal across different biological samples. The spreadsheet file contains 3 sheets: 1) AllPositiveProteins listing data for all positive proteins (ie, all proteins having at least one positive peptide); 2) unique15mers listing data for all positive proteins where their most reactive peptide is different from the other proteins' most reactive peptides (i.e., identical regions located in different proteins are collapsed); and 3) unique7mer listing data for all positive proteins where all 7-mers within their most reactive peptide are different from all 7-mers within the other proteins' most reactive peptides (i.e., no 7-mers are shared between regions). See Experimental Procedures for more details and columns descriptions (Excel File).
- Supplemental Table 3 - Supplementary Table 3. Chagas Antigens included in the High-Density Chagas-Chip (Excel Spreadsheet). The table lists T. cruzi proteins with previous serological evidence in infected humans.
- Supplemental Table 4 - Supplementary Table 4. Protein Sequences included in the HD-Chip array (Excel Spreadsheet). Accession numbers and protein sequences of the proteins included in the array. The file lists T. cruzi proteins, as well as neo-proteins (proteins of random sequence).
- Supplemental Table 5 - Supplementary Table 5. Oligonucleotides and GST-fusion peptides used in this study. These were used to generate the different forms of the TSSA antigen for validation in an ELISA format assay (results in Supplementary Table 1).
