# Supplementary material for: Towards High-throughput Immunomics for Infectious Diseases: Use of Next-generation Peptide Microarrays for Rapid Discovery and Mapping of Antigenic Determinants
Source: Mol Cell Proteomics. 2015 Jul;14(7):1871–84. doi: 10.1074/mcp.M114.045906 (PMC4587317; doi:10.1074/mcp.M114.045906)
Supplement: Supplemental Data [file supp_M114.045906_mcp.M114.045906-3.pdf]

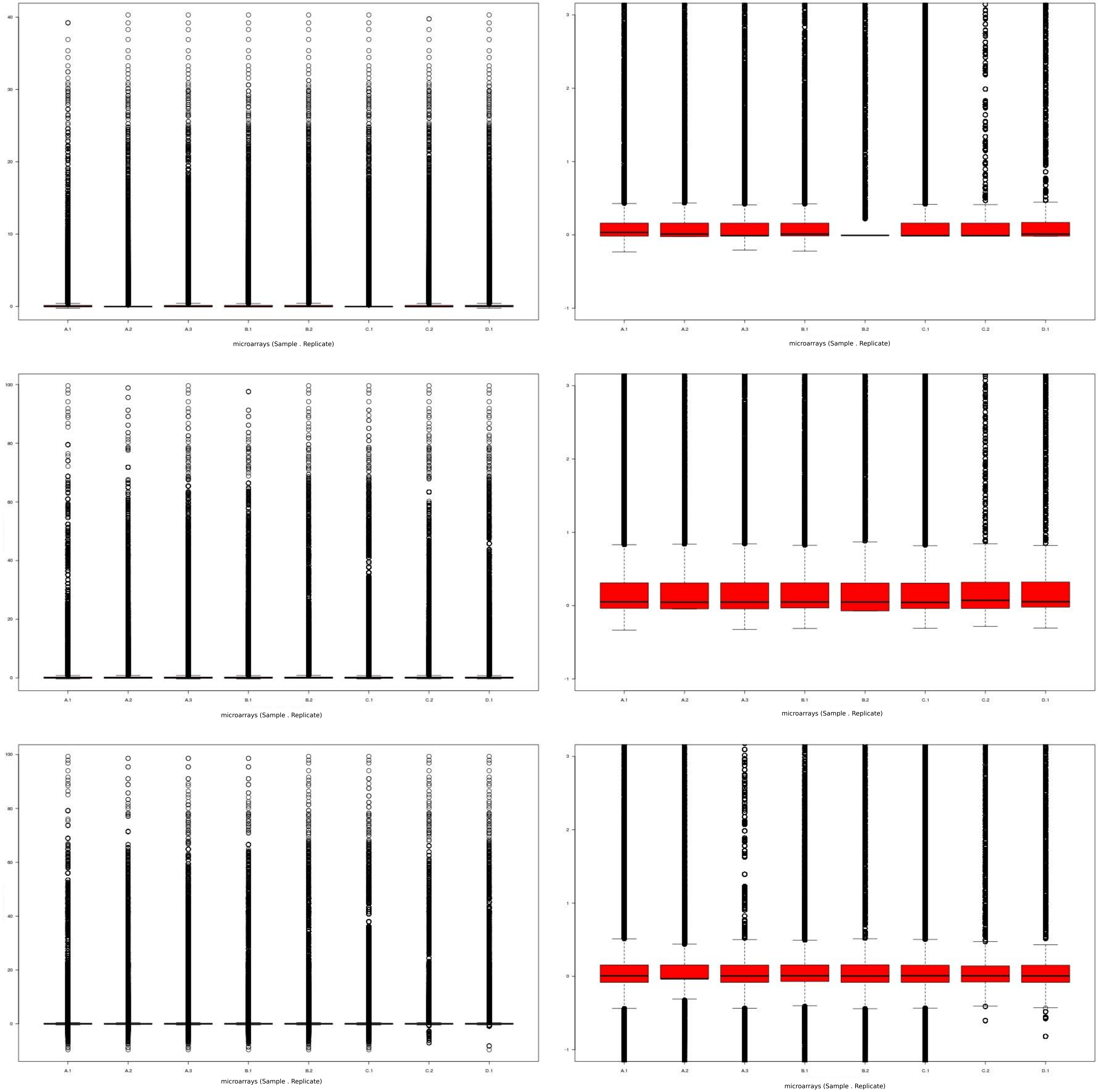


**Boxplots of normalized data.** The plots in the figure show the distribution of intensity values after normalization of readouts from the incubation with negative sample (top), cumulative readouts after incubation with negative followed by positive sample (middle) and positive, disease-specific values (after subtraction of negative signal, bottom). Boxplots correspond to each microarray (from left to right: A1, A2, A3, B1, B2, C1, C2 and D1). Figures on the right side show the same boxplots but zooming in the bulk of the data.
