# Supplementary material for: Towards High-throughput Immunomics for Infectious Diseases: Use of Next-generation Peptide Microarrays for Rapid Discovery and Mapping of Antigenic Determinants
Source: Mol Cell Proteomics. 2015 Jul;14(7):1871–84. doi: 10.1074/mcp.M114.045906 (PMC4587317; doi:10.1074/mcp.M114.045906)
Supplement: Supplemental Data [file supp_M114.045906_mcp.M114.045906-1.zip › Supplementary Figure 1- Epitope mapping performance/EpitopePerformance.SampleD.pdf]

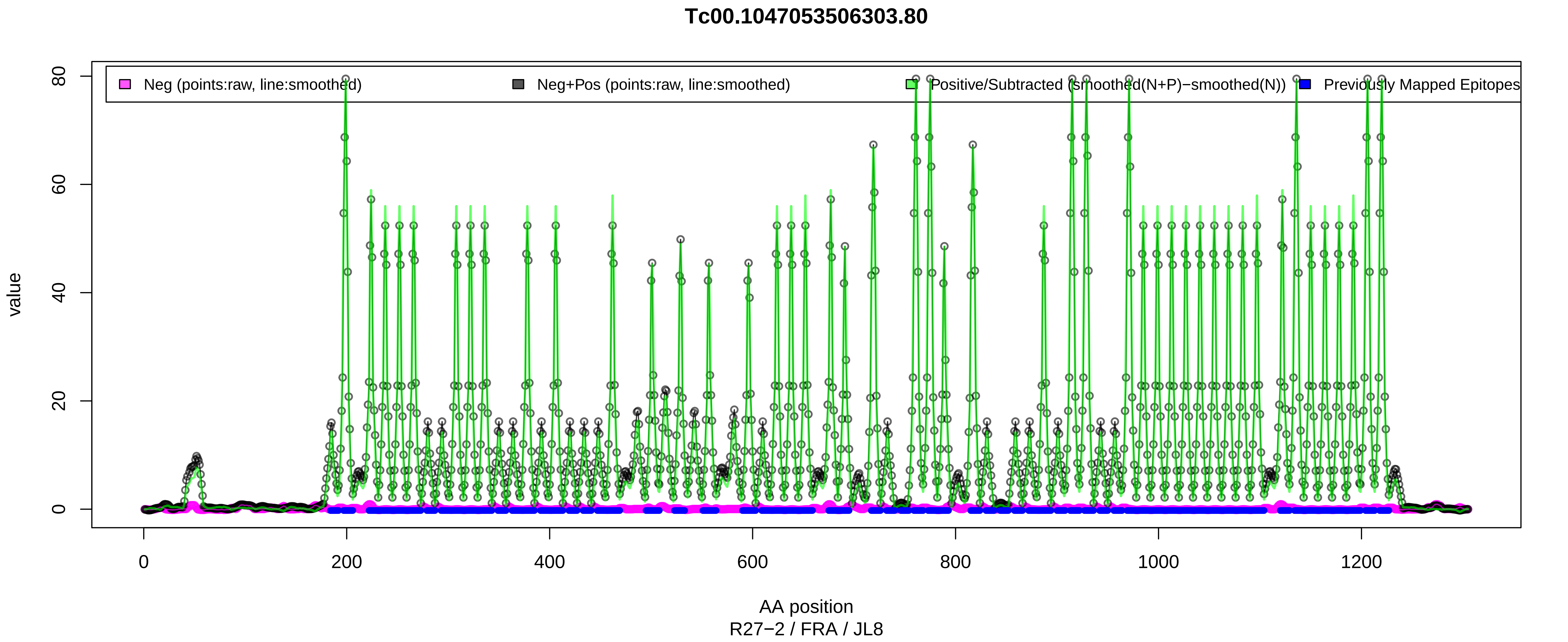
Tc00.1047053506303.80
Neg (points:raw, line:smoothed)
Neg+Pos (points:raw, line:smoothed)
Positive/Subtracted (smoothed(N+P)−smoothed(N))
Previously Mapped Epitopes
value
0
20
40
60
80
0
200
400
600
800
1000
1200
AA position
R27−2 / FRA / JL8

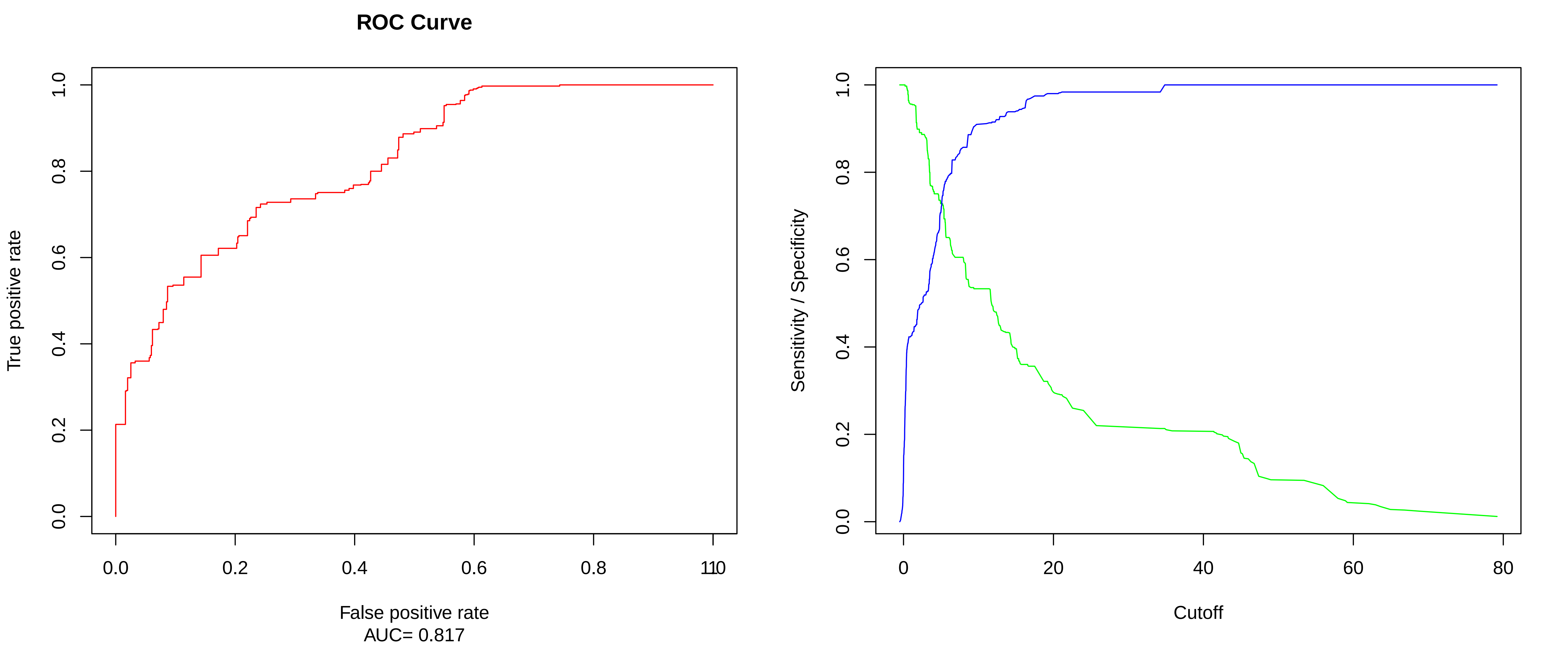
ROC Curve
True positive rate
False positive rate
AUC= 0.817
0.0
0.2
0.4
0.6
0.8
1.0
Sensitivity / Specificity
Cutoff
0
20
40
60
80

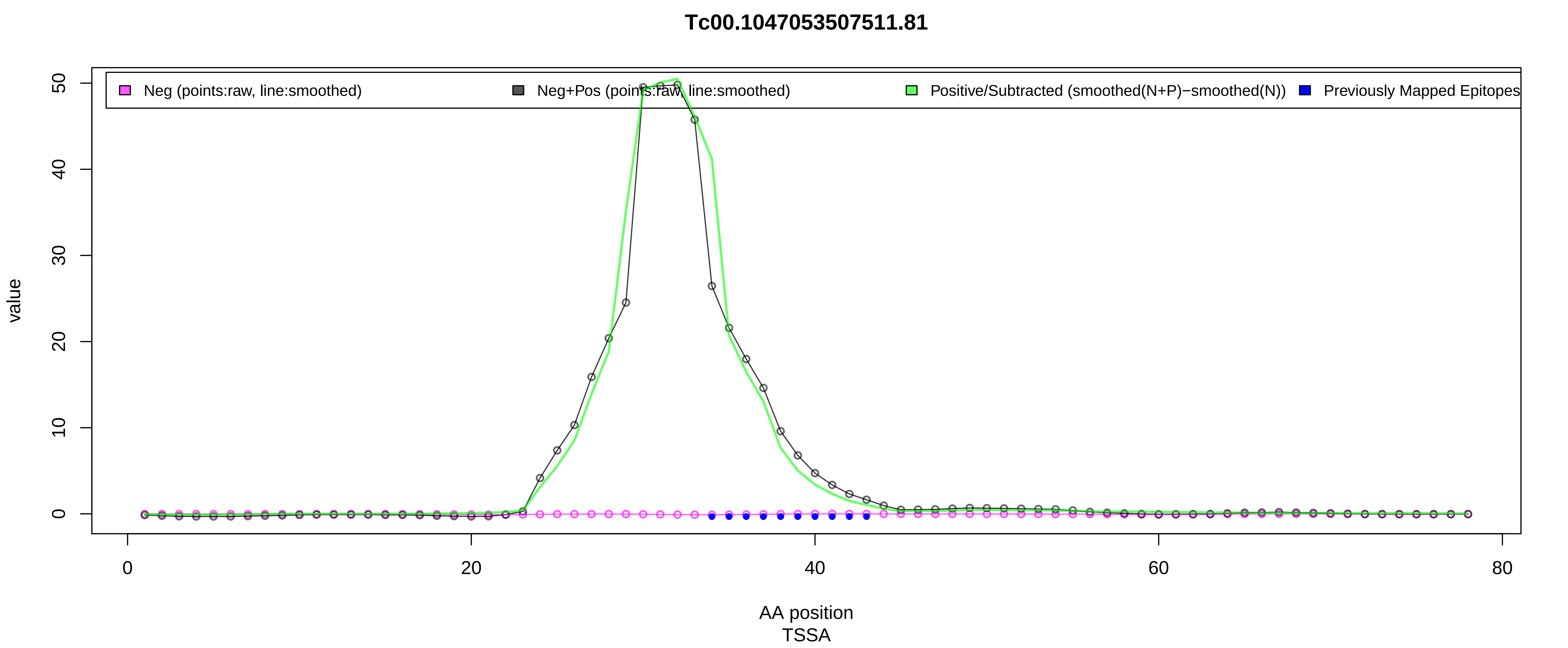

Tc00.1047053507511.81
Neg (points:raw, line:smoothed)
Neg+Pos (points:raw, line:smoothed)
Positive/Subtracted (smoothed(N+P)−smoothed(N))
Previously Mapped Epitopes
value
0
10
20
30
40
50
0
20
40
60
80
AA position
TSSA

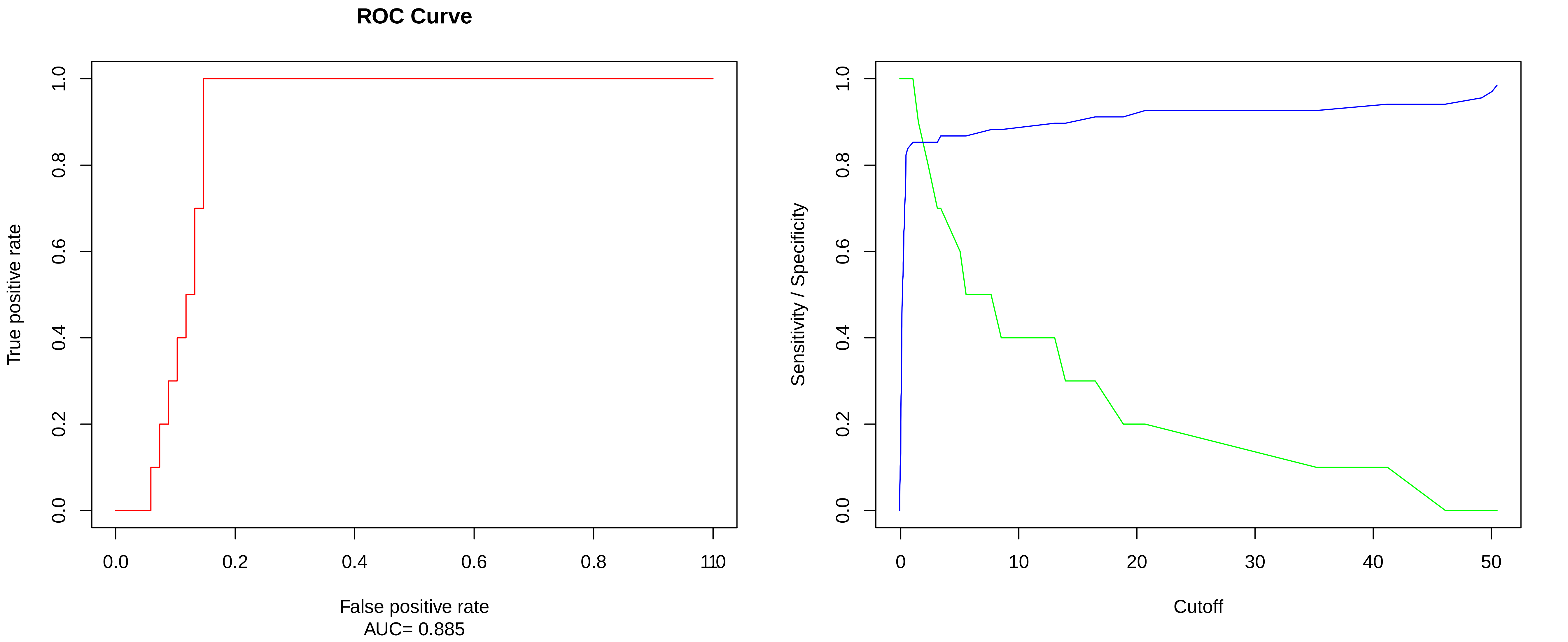

ROC Curve
True positive rate
False positive rate
AUC= 0.885
0.0
0.2
0.4
0.6
0.8
1.0
Sensitivity / Specificity
Cutoff
0
10
20
30
40
50

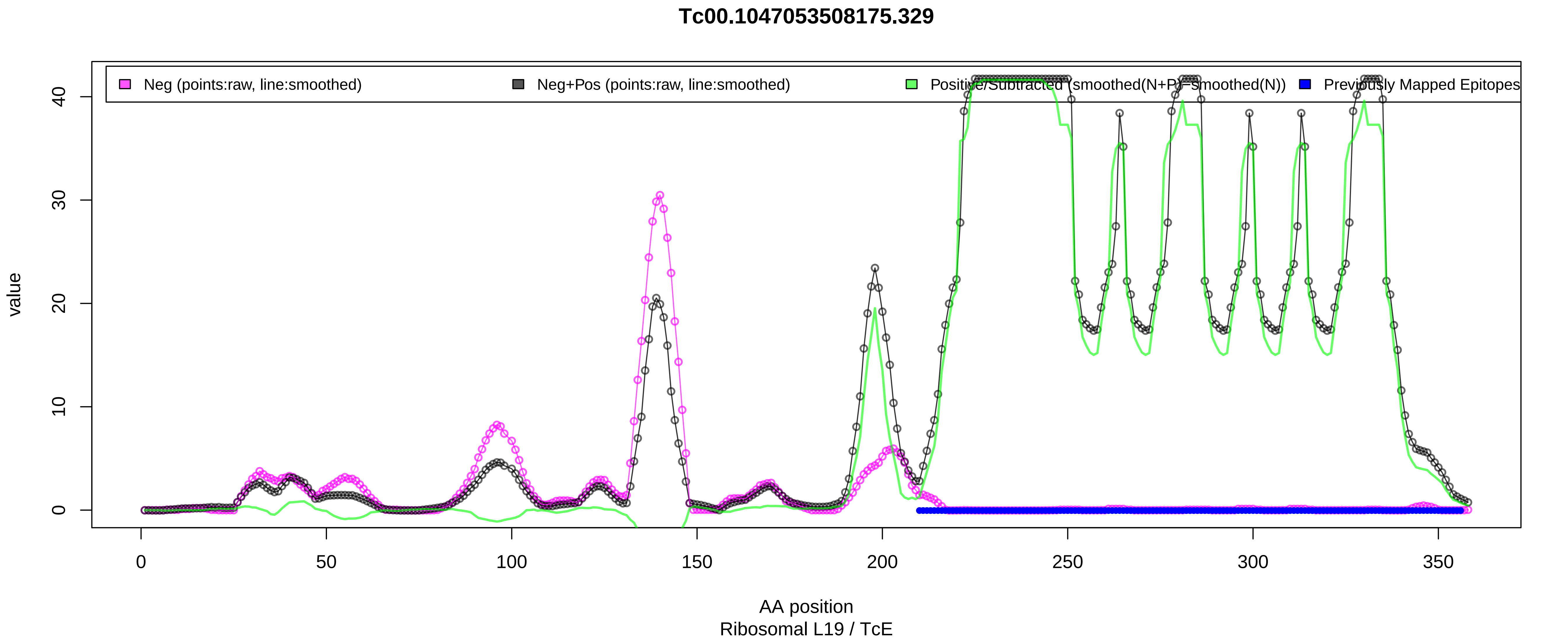
Tc00.1047053508175.329
Neg (points:raw, line:smoothed)
Neg+Pos (points:raw, line:smoothed)
Positive/Subtracted (smoothed(N+P)-smoothed(N))
Previously Mapped Epitopes
value
0
10
20
30
40
0
50
100
150
200
250
300
350
AA position
Ribosomal L19 / TcE

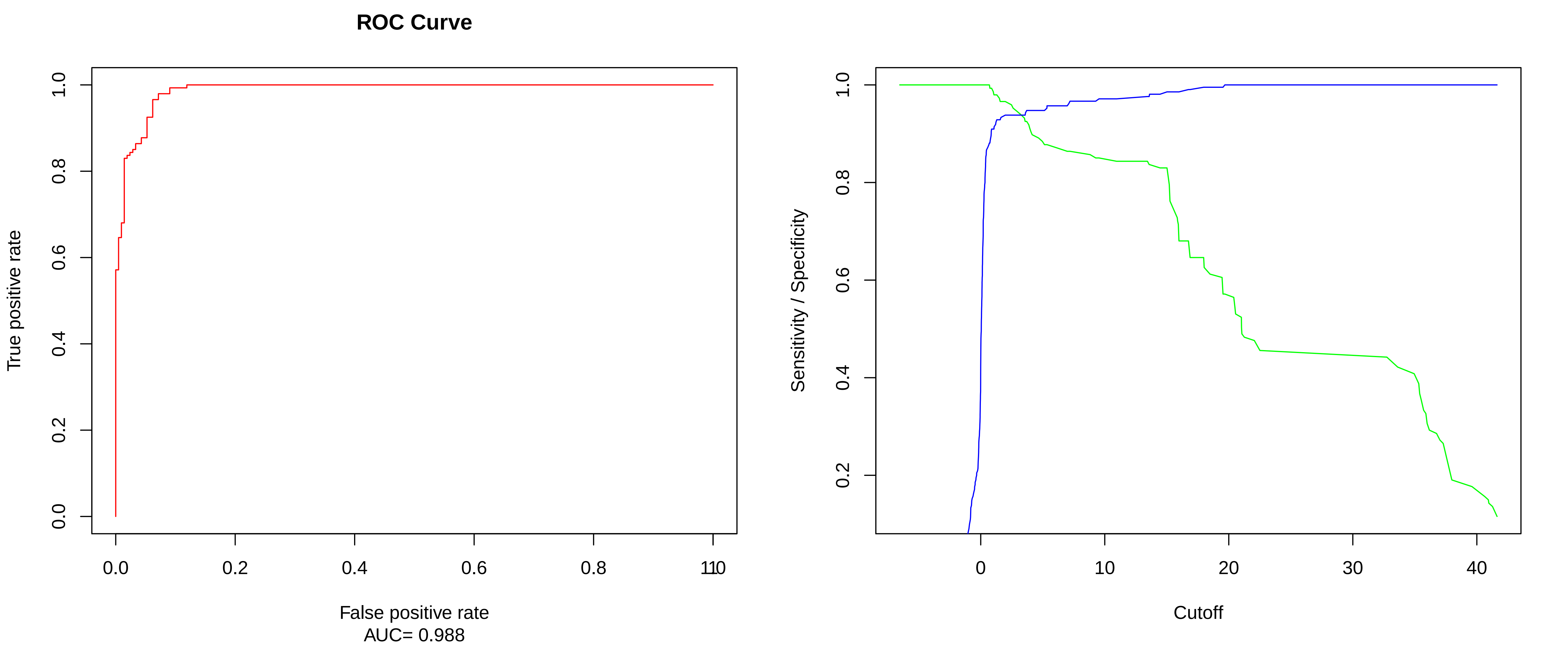
ROC Curve
True positive rate
0.0
0.2
0.4
0.6
0.8
1.0
False positive rate
AUC= 0.988
Sensitivity / Specificity
Cutoff
0
10
20
30
40

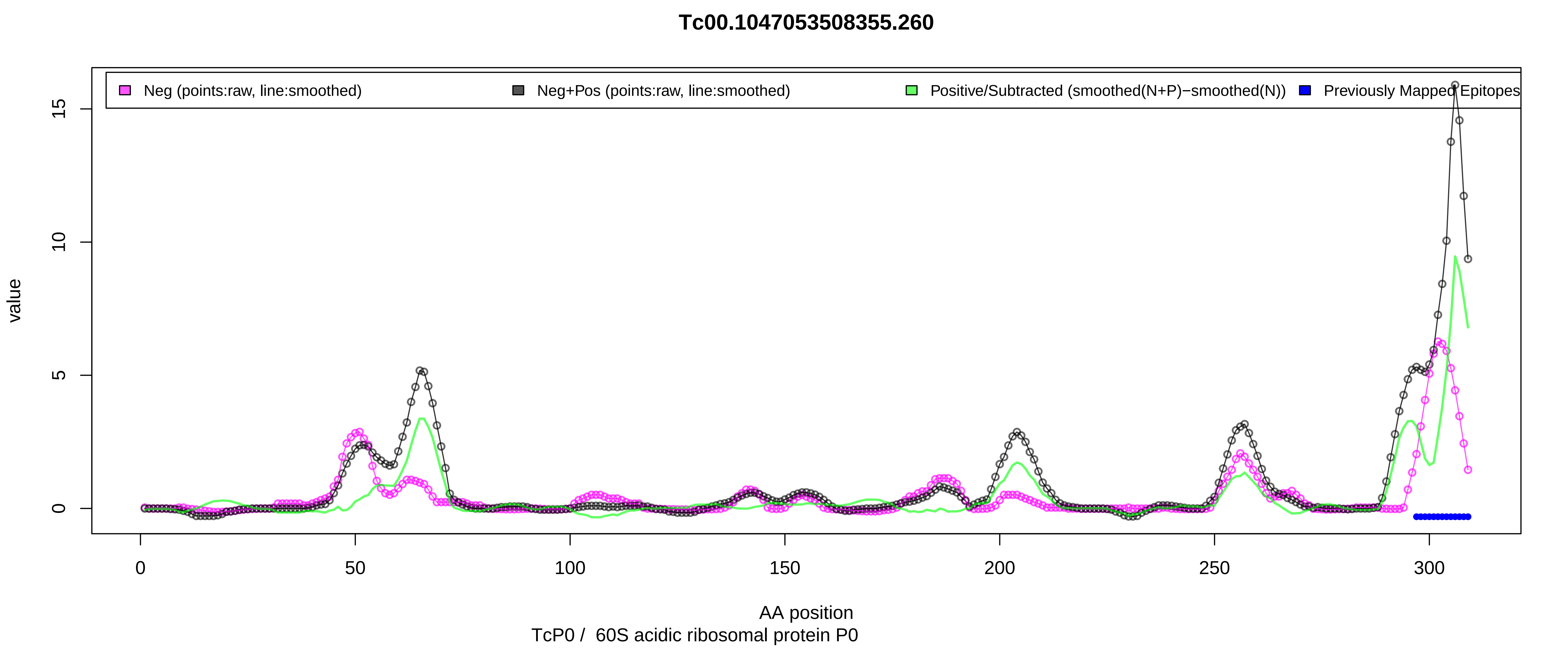
Tc00.1047053508355.260
Neg (points:raw, line:smoothed)
Neg+Pos (points:raw, line:smoothed)
Positive/Subtracted (smoothed(N+P)−smoothed(N))
Previously Mapped Epitopes
value
0
5
10
15
0
50
100
150
200
250
300
AA position
TcP0 / 60S acidic ribosomal protein P0

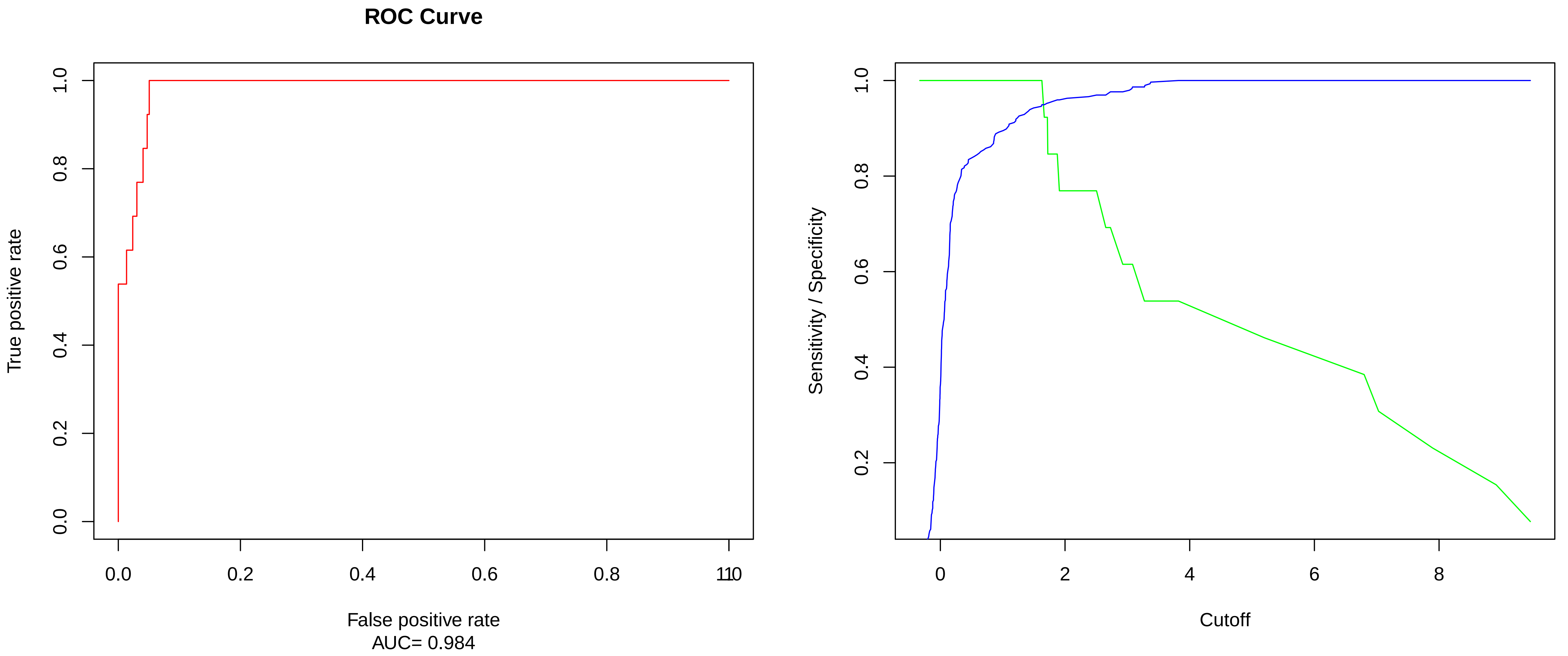
ROC Curve
True positive rate
False positive rate
AUC= 0.984
0.0
0.2
0.4
0.6
0.8
1.0
Sensitivity / Specificity
Cutoff
0
2
4
6
8

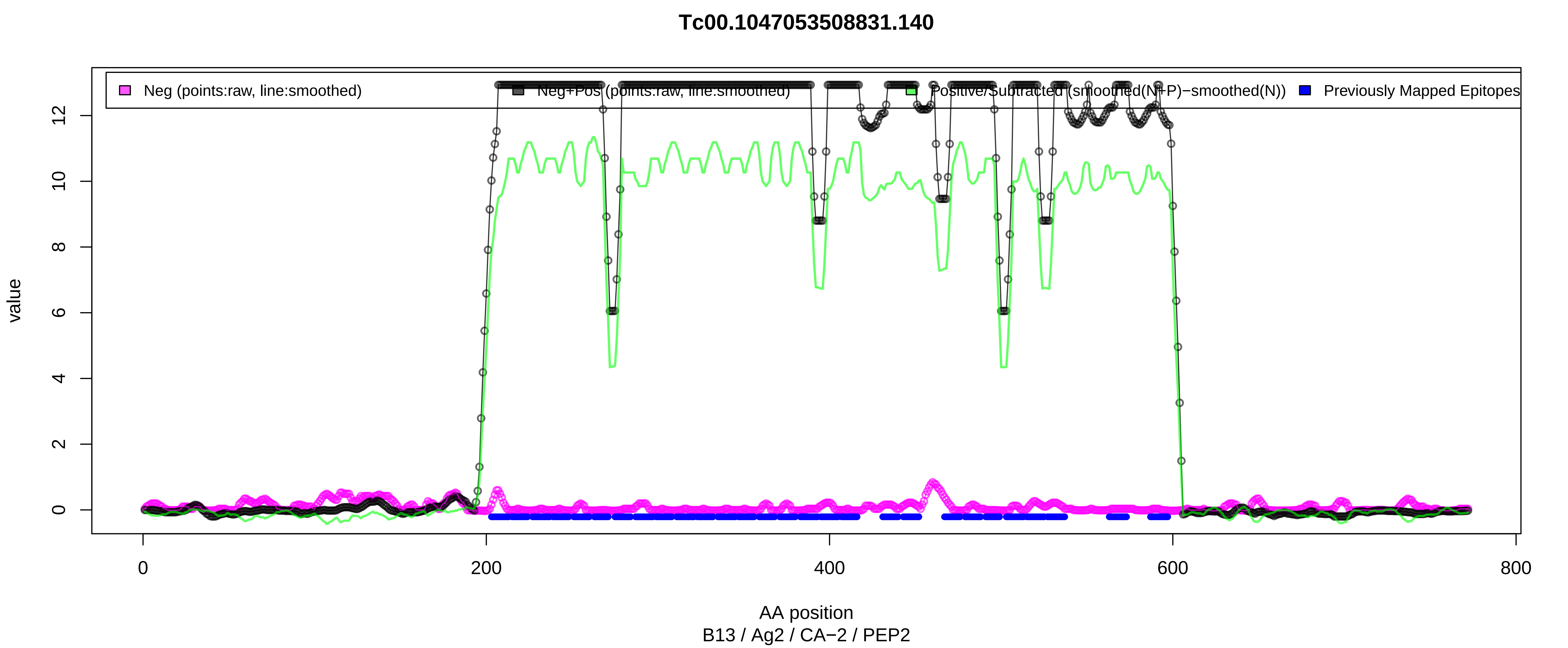

Tc00.1047053508831.140
Neg (points:raw, line:smoothed)
Neg+Pos (points:raw, line:smoothed)
Positive/Subtracted (smoothed(N+P)−smoothed(N))
Previously Mapped Epitopes
value
0
2
4
6
8
10
12
0
200
400
600
800
AA position
B13 / Ag2 / CA−2 / PEP2

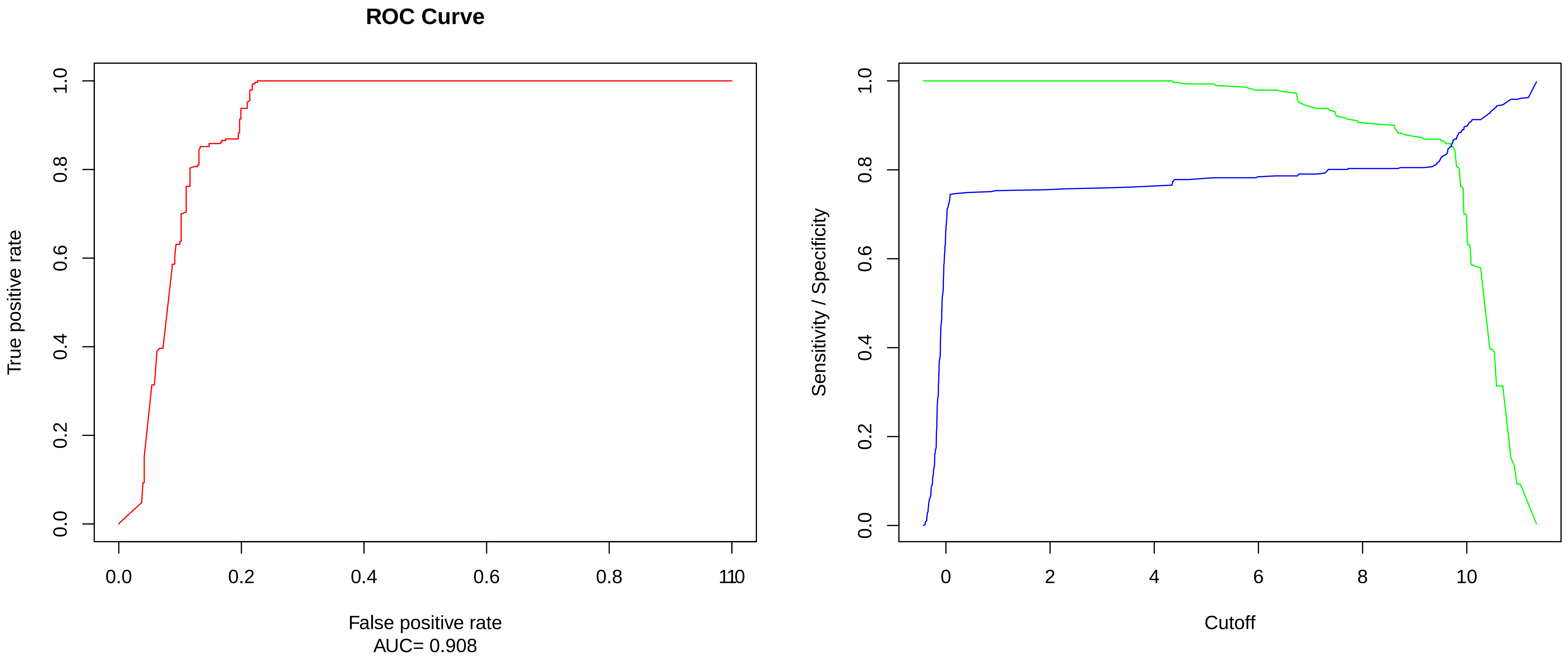
ROC Curve
True positive rate
0.0
0.2
0.4
0.6
0.8
1.0
0.0
0.2
0.4
0.6
0.8
1.0
False positive rate
AUC= 0.908
Sensitivity / Specificity
0.0
0.2
0.4
0.6
0.8
1.0
0
2
4
6
8
10
Cutoff

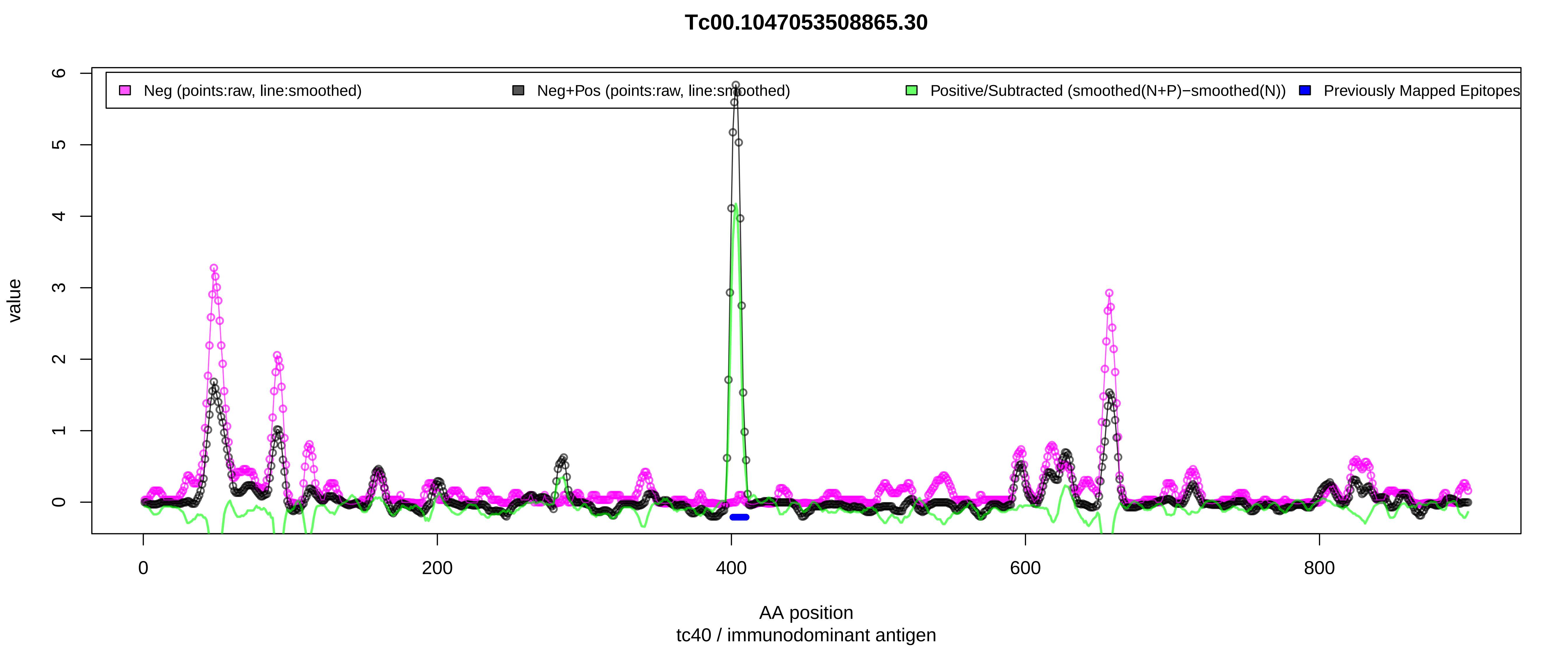

Tc00.1047053508865.30
Neg (points:raw, line:smoothed)
Neg+Pos (points:raw, line:smoothed)
Positive/Subtracted (smoothed(N+P)−smoothed(N))
Previously Mapped Epitopes
value
0
1
2
3
4
5
6
0
200
400
600
800
AA position
tc40 / immunodominant antigen

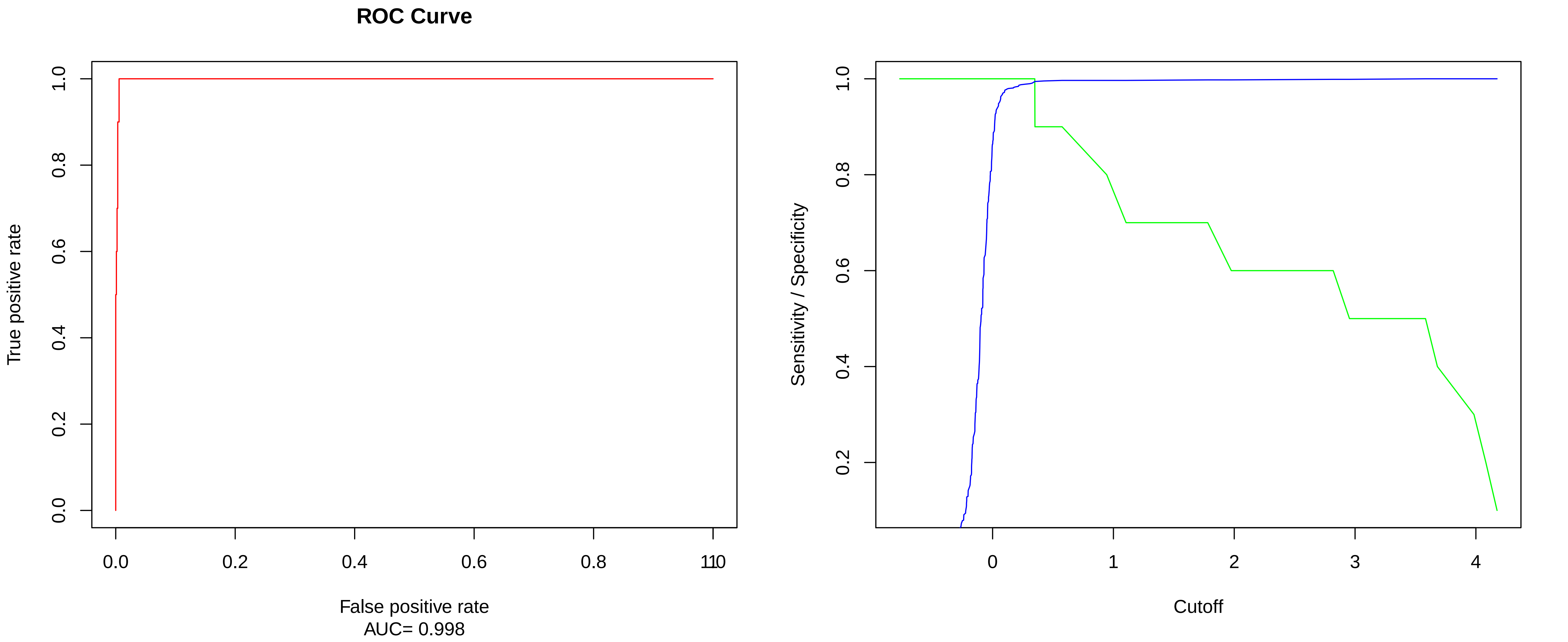
ROC Curve
True positive rate
0.0
0.2
0.4
0.6
0.8
1.0
False positive rate
AUC= 0.998
Sensitivity / Specificity
Cutoff
0
1
2
3
4

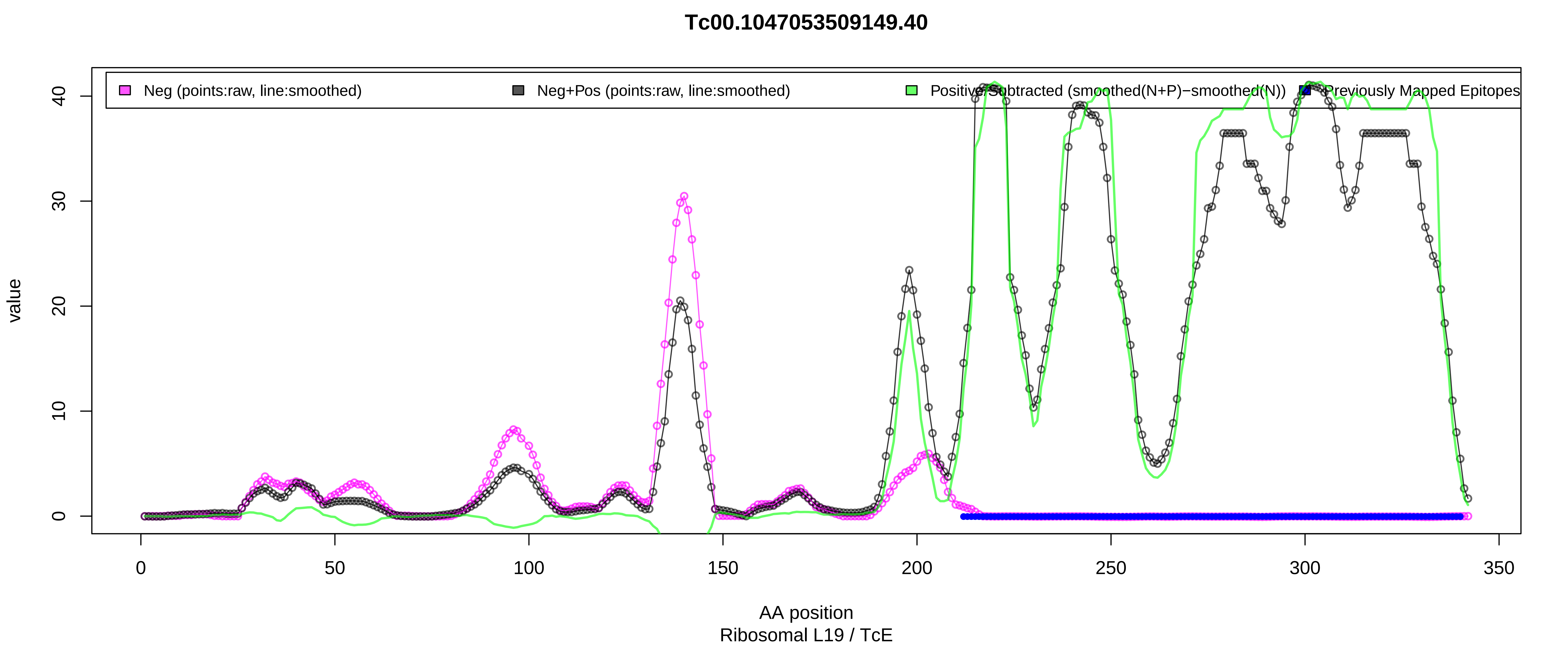

Tc00.1047053509149.40
Neg (points:raw, line:smoothed)
Neg+Pos (points:raw, line:smoothed)
Positive Subtracted (smoothed(N+P)−smoothed(N))
Previously Mapped Epitopes
value
0
10
20
30
40
0
50
100
150
200
250
300
350
AA position
Ribosomal L19 / TcE

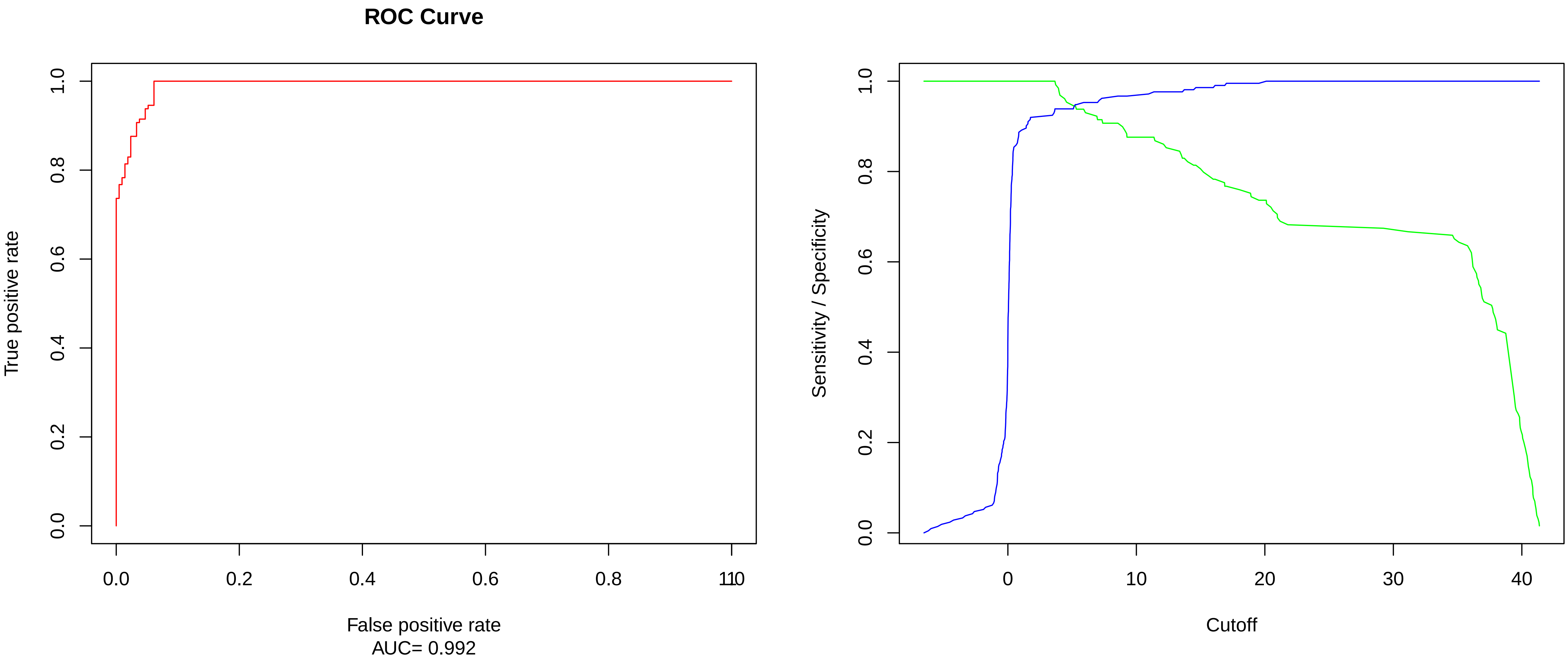
ROC Curve
True positive rate
False positive rate
AUC= 0.992
0.0
0.2
0.4
0.6
0.8
1.0
Sensitivity / Specificity
Cutoff
0
10
20
30
40

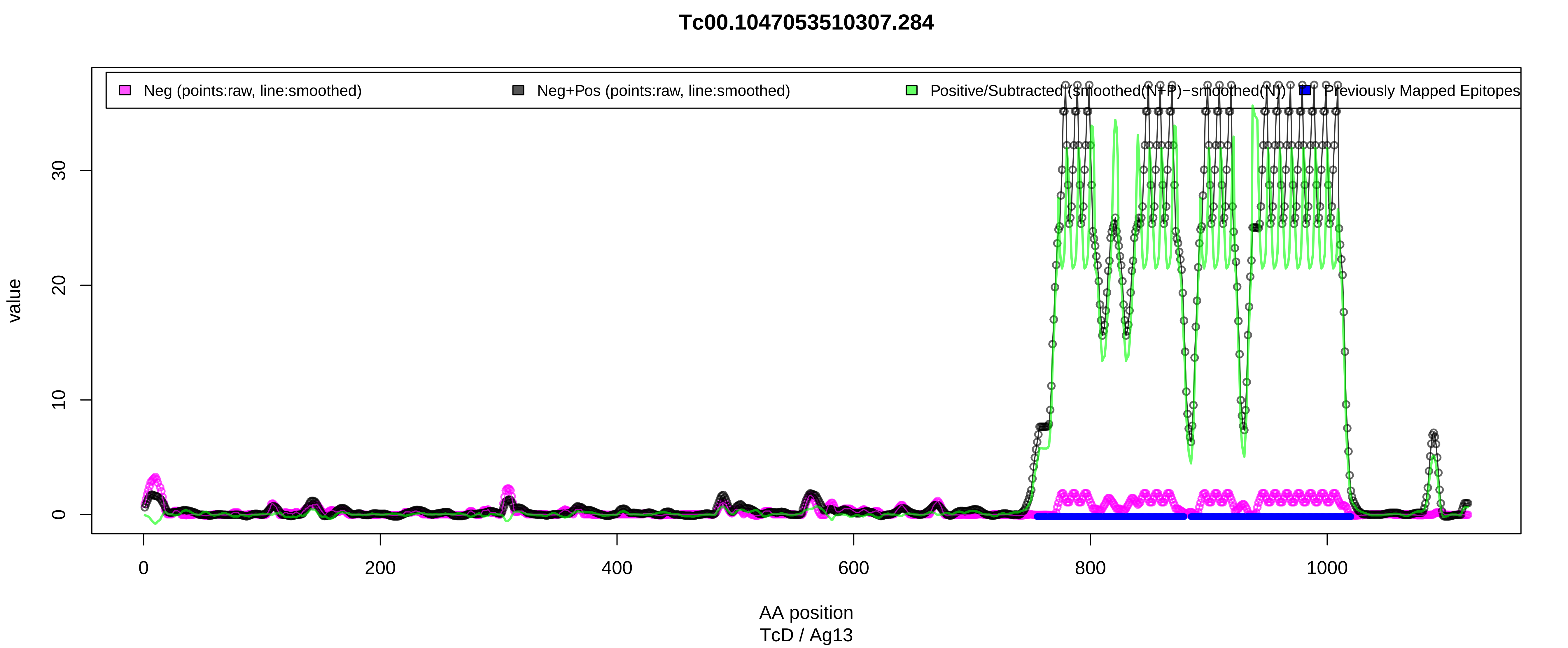

Tc00.1047053510307.284
Neg (points:raw, line:smoothed)
Neg+Pos (points:raw, line:smoothed)
Positive/Subtracted (smoothed(N+P)−smoothed(N))
Previously Mapped Epitopes
value
0
10
20
30
0
200
400
600
800
1000
AA position
TcD / Ag13

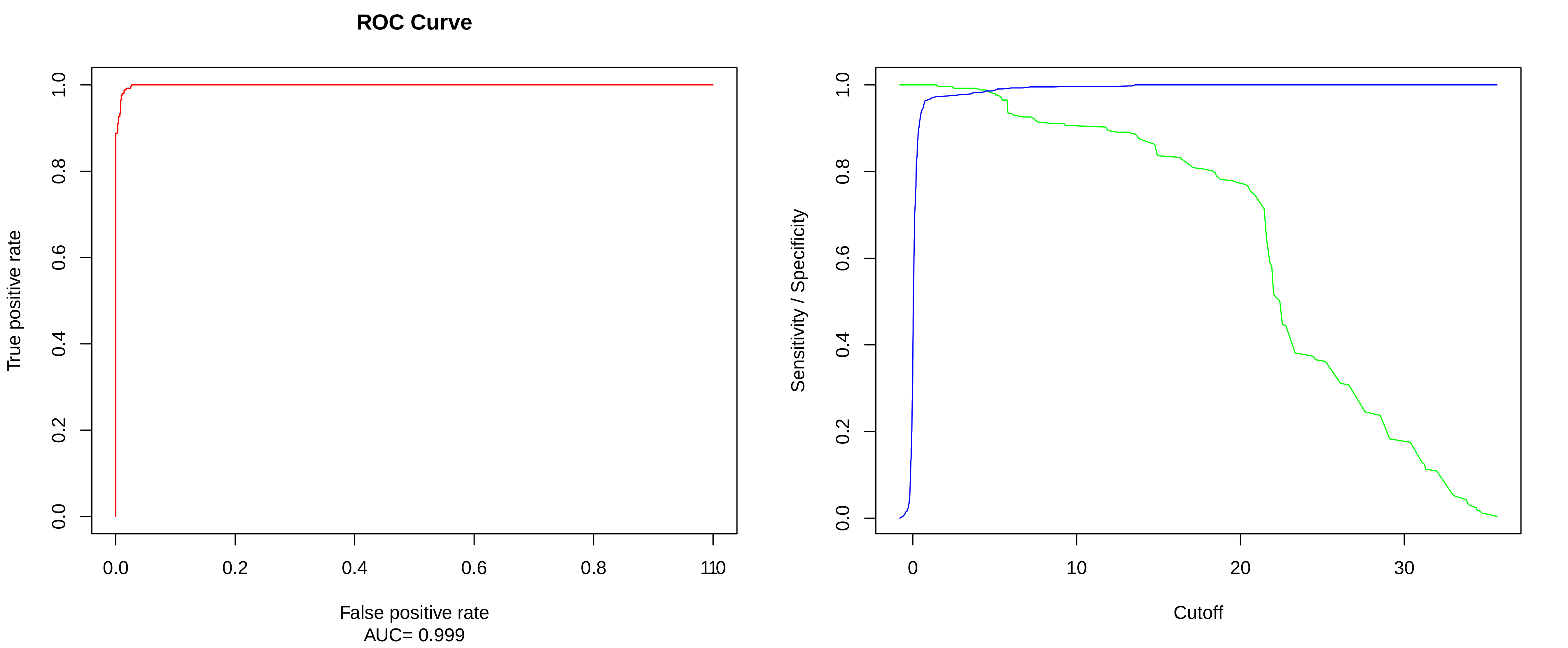
ROC Curve
True positive rate
0.0
0.2
0.4
0.6
0.8
1.0
False positive rate
AUC= 0.999
Sensitivity / Specificity
0
10
20
30
Cutoff

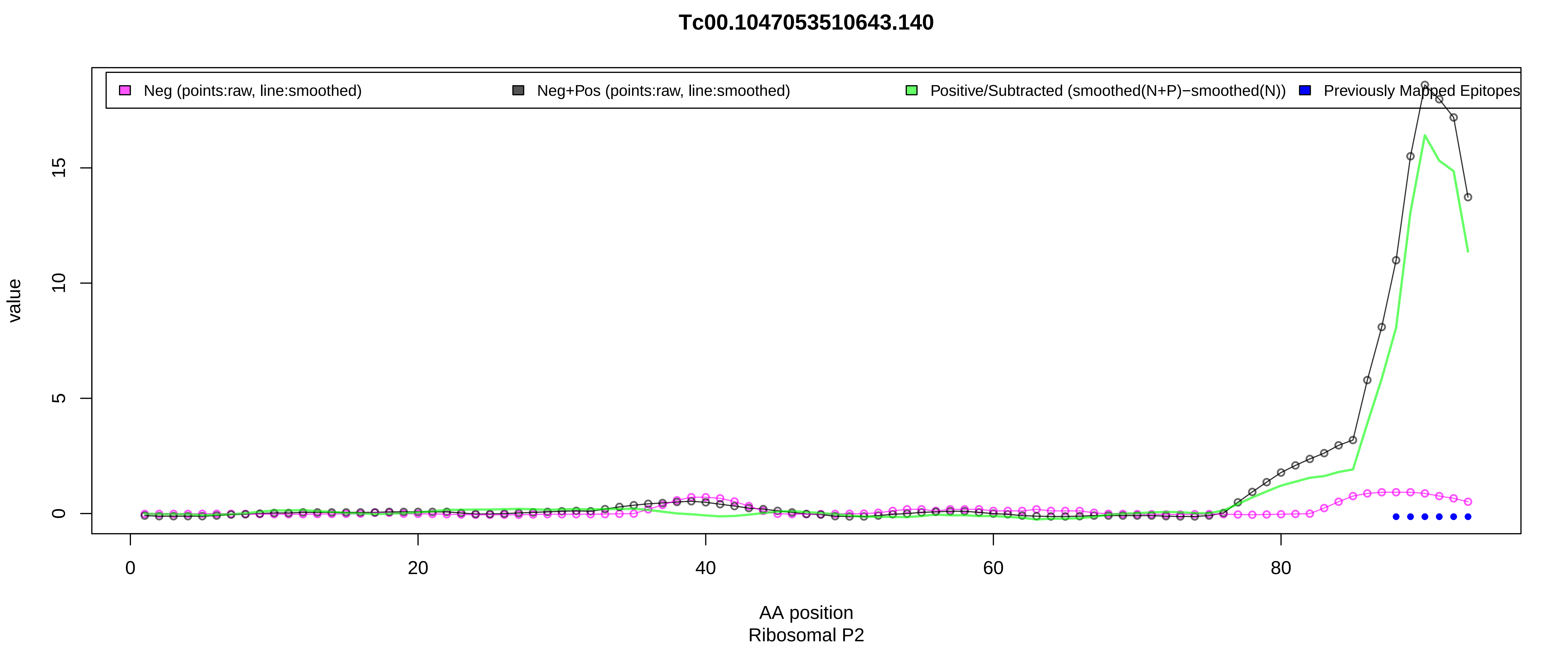

Tc00.1047053510643.140
Neg (points:raw, line:smoothed)
Neg+Pos (points:raw, line:smoothed)
Positive/Subtracted (smoothed(N+P)−smoothed(N))
Previously Mapped Epitopes
value
0
5
10
15
0
20
40
60
80
AA position
Ribosomal P2

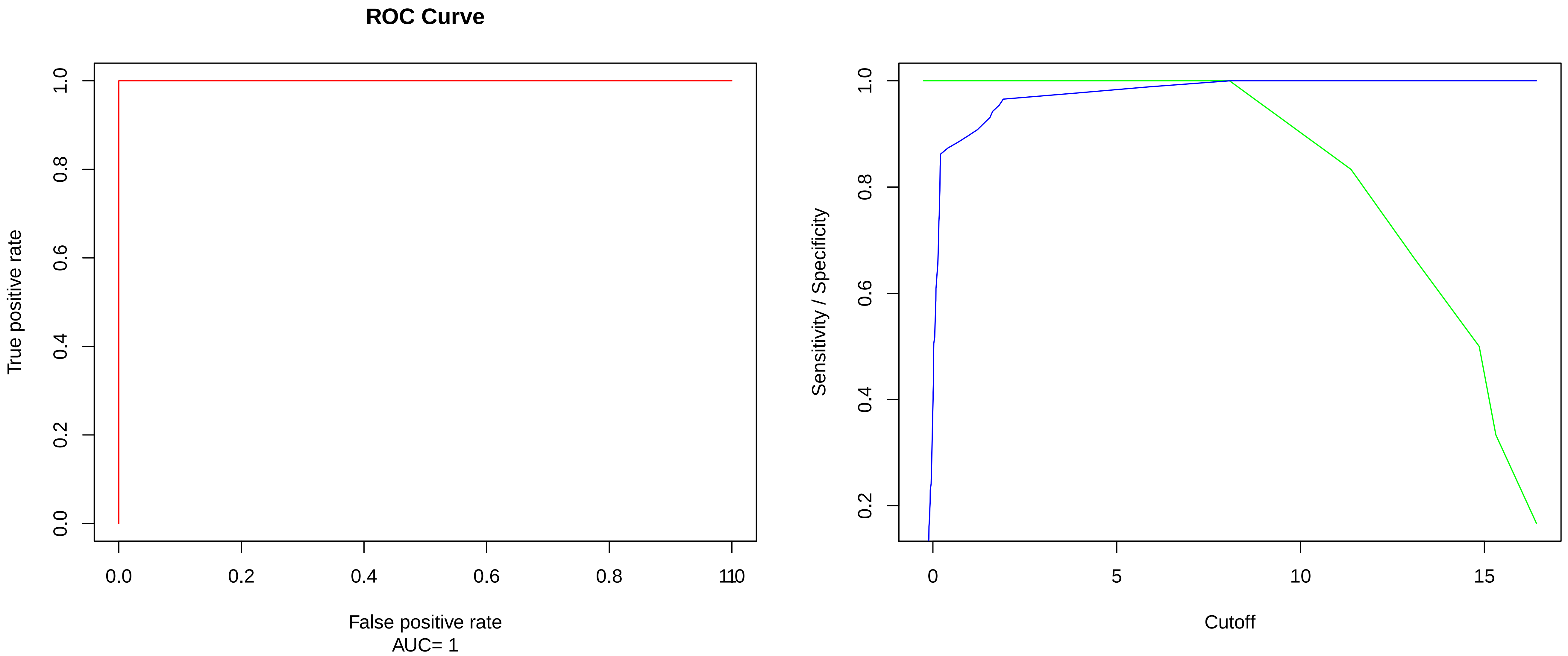
ROC Curve
True positive rate
False positive rate
AUC= 1
Sensitivity / Specificity
Cutoff

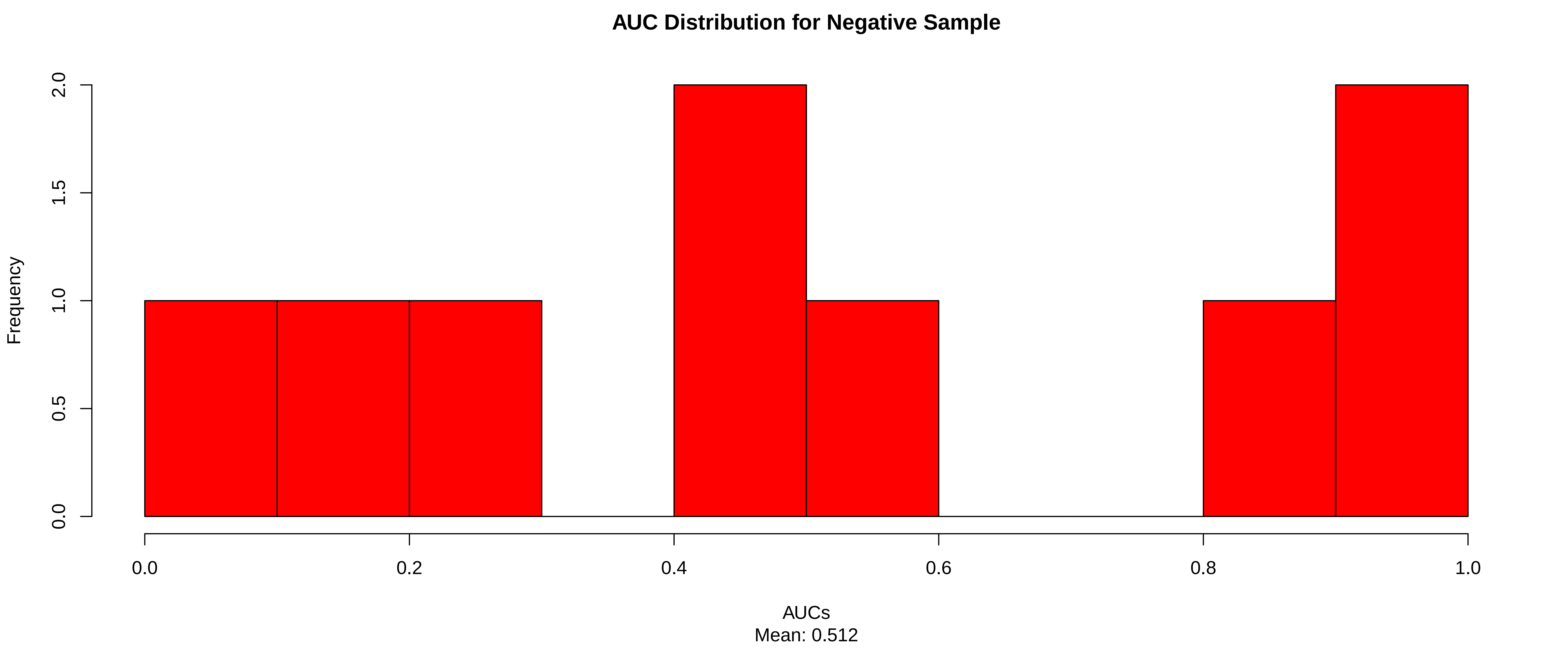
AUC Distribution for Negative Sample
Frequency
0.0
0.5
1.0
1.5
2.0
0.0
0.2
0.4
0.6
0.8
1.0
AUCs
Mean: 0.512

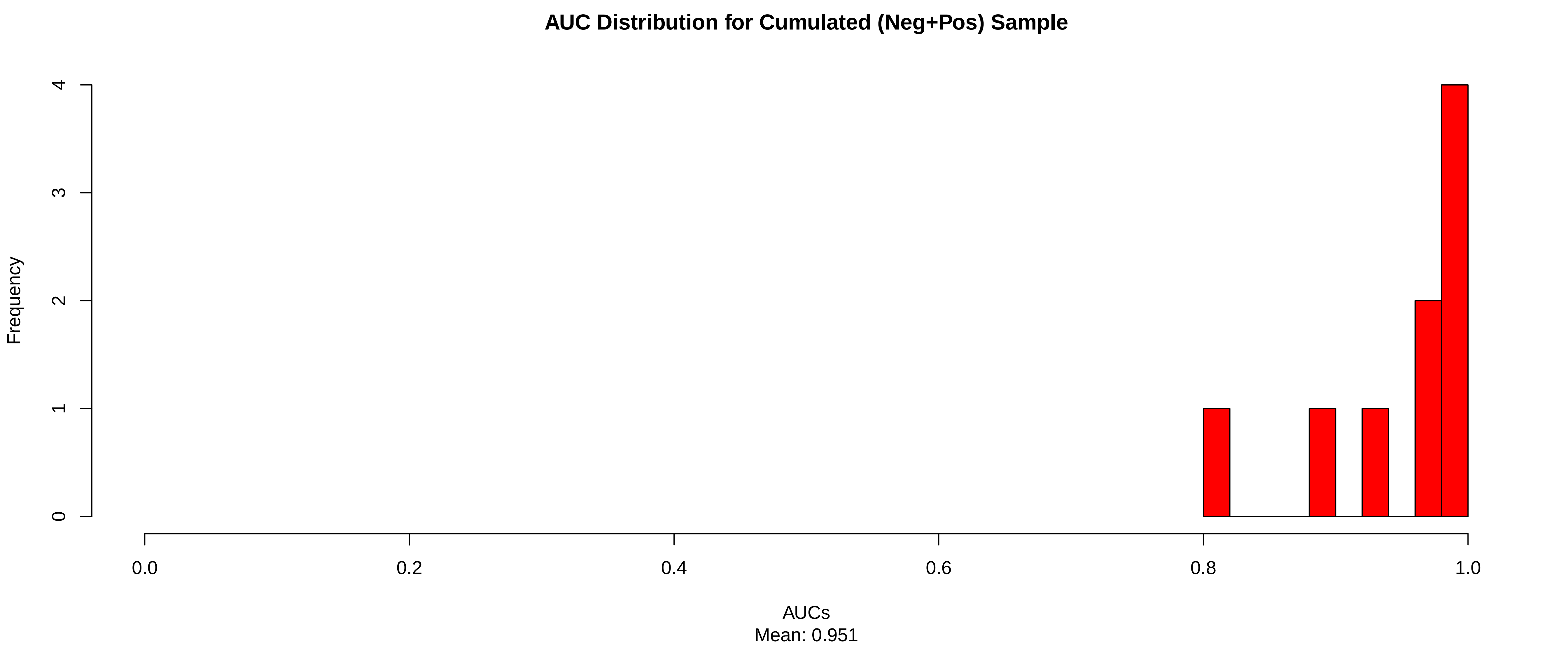
AUC Distribution for Cumulated (Neg+Pos) Sample
Frequency
0
1
2
3
4
0.0
0.2
0.4
0.6
0.8
1.0
AUCs
Mean: 0.951

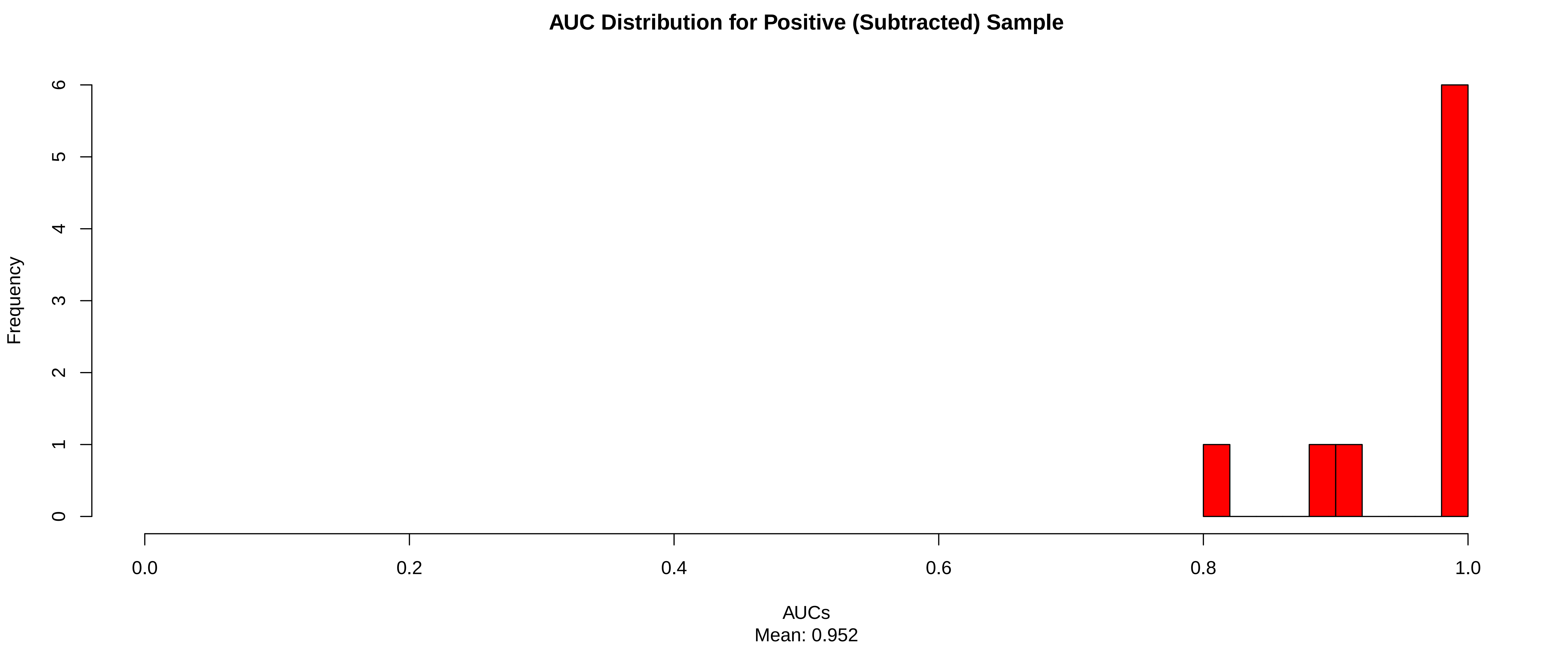
AUC Distribution for Positive (Subtracted) Sample
Frequency
0
1
2
3
4
5
6
0.0
0.2
0.4
0.6
0.8
1.0
AUCs
Mean: 0.952
