# Supplementary material for: Towards High-throughput Immunomics for Infectious Diseases: Use of Next-generation Peptide Microarrays for Rapid Discovery and Mapping of Antigenic Determinants
Source: Mol Cell Proteomics. 2015 Jul;14(7):1871–84. doi: 10.1074/mcp.M114.045906 (PMC4587317; doi:10.1074/mcp.M114.045906)
Supplement: Supplemental Data [file supp_M114.045906_mcp.M114.045906-1.zip › Supplementary Figure 1- Epitope mapping performance/Legend for Supplementary Figure 1.docx]

**Supplementary Figure 1. Epitope mapping performance.** The figure shows antigenicity profile plots for all antigens used for epitope mapping performance assessment. In these plots, the antibody-binding signal for each protein was reconstructed based on the array data, as explained in the text. The plots also show the location of previously known epitopes (blue marks at the bottom of the plot frame). After each antibody-binding profile, we provide an assessment of epitope mapping performance (ROC curves) for each antigen. The zip file contains 5 PDFs, one for each sample (sera pools A to D) and one displaying the averaged signal from all samples.
