# Supplementary material for: Towards High-throughput Immunomics for Infectious Diseases: Use of Next-generation Peptide Microarrays for Rapid Discovery and Mapping of Antigenic Determinants
Source: Mol Cell Proteomics. 2015 Jul;14(7):1871–84. doi: 10.1074/mcp.M114.045906 (PMC4587317; doi:10.1074/mcp.M114.045906)
Supplement: Supplemental Data [file supp_M114.045906_mcp.M114.045906-2.zip › Supplementary Figure 2 - Antigenicity Profiles of Positive Proteins/proteinProfilesPositiveProteins.Average.pdf]

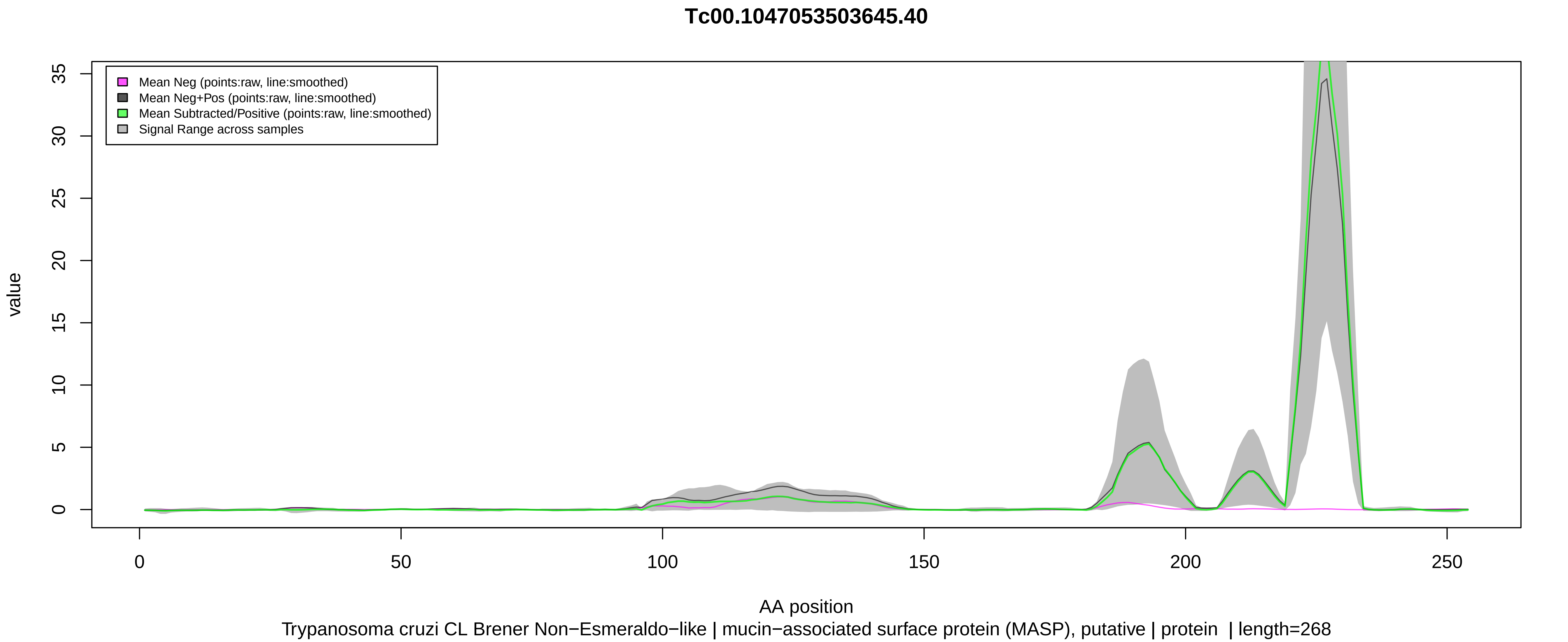

Tc00.1047053503645.40
Mean Neg (points:raw, line:smoothed)
Mean Neg+Pos (points:raw, line:smoothed)
Mean Subtracted/Positive (points:raw, line:smoothed)
Signal Range across samples
value
0
5
10
15
20
25
30
35
0
50
100
150
200
250
AA position
Trypanosoma cruzi CL Brener Non−Esmeraldo−like | mucin−associated surface protein (MASP), putative | protein | length=268

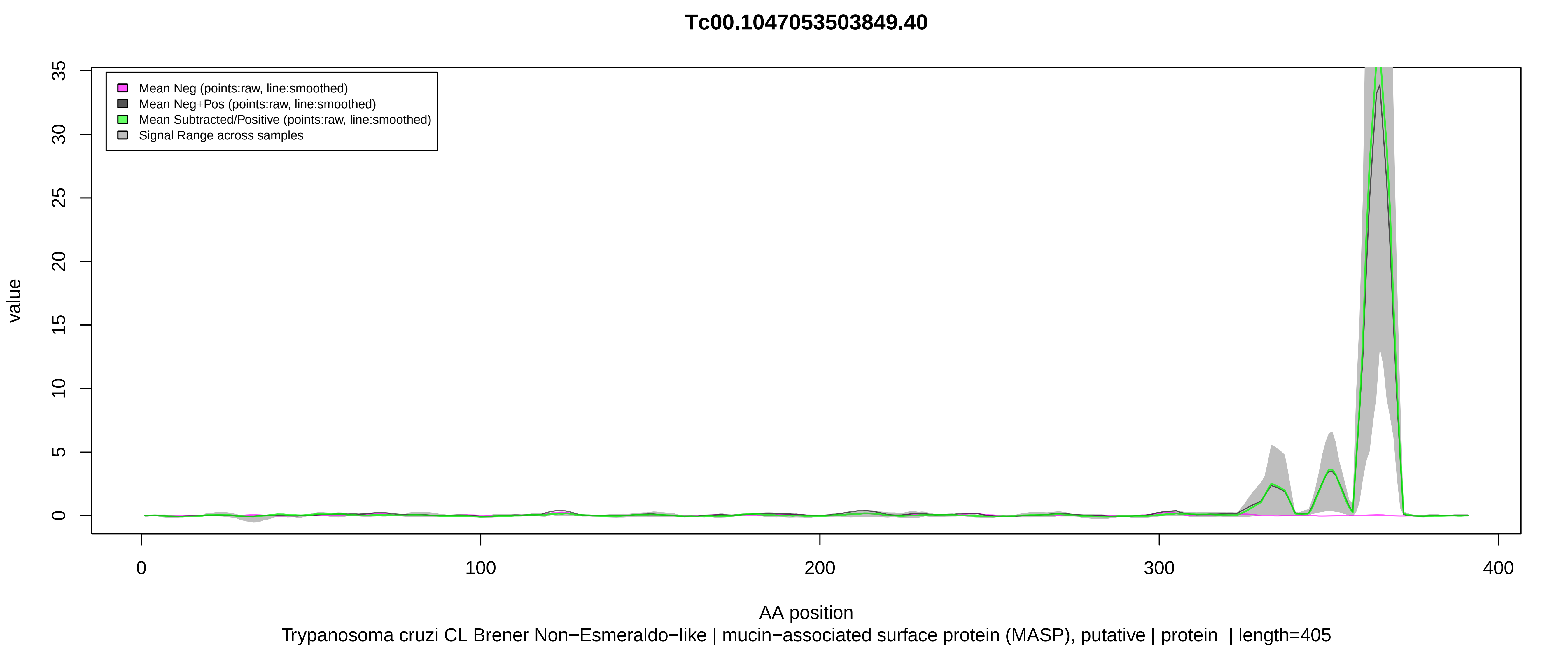

Tc00.1047053503849.40
Mean Neg (points:raw, line:smoothed)
Mean Neg+Pos (points:raw, line:smoothed)
Mean Subtracted/Positive (points:raw, line:smoothed)
Signal Range across samples
value
0
5
10
15
20
25
30
35
0
100
200
300
400
AA position
Trypanosoma cruzi CL Brener Non−Esmeraldo−like | mucin−associated surface protein (MASP), putative | protein | length=405

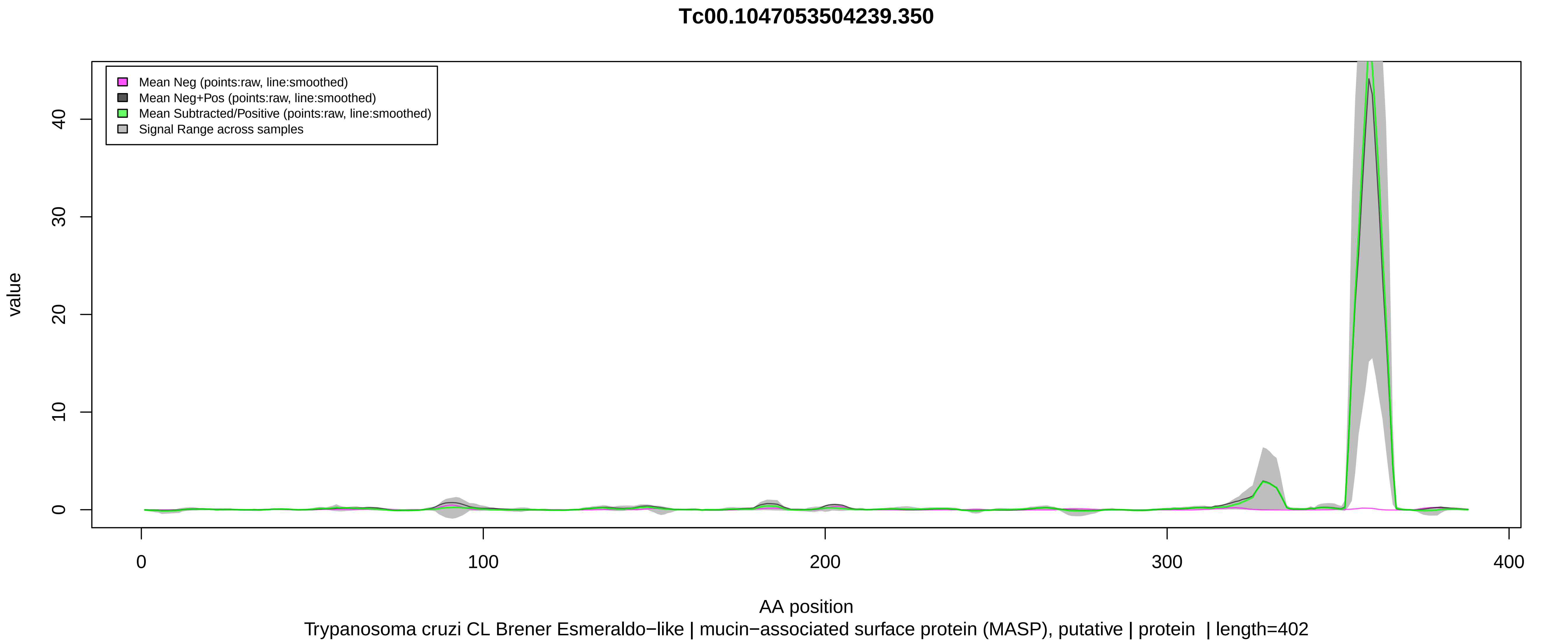

Tc00.1047053504239.350
Mean Neg (points:raw, line:smoothed)
Mean Neg+Pos (points:raw, line:smoothed)
Mean Subtracted/Positive (points:raw, line:smoothed)
Signal Range across samples
value
0
10
20
30
40
0
100
200
300
400
AA position
Trypanosoma cruzi CL Brener Esmeraldo−like | mucin−associated surface protein (MASP), putative | protein | length=402

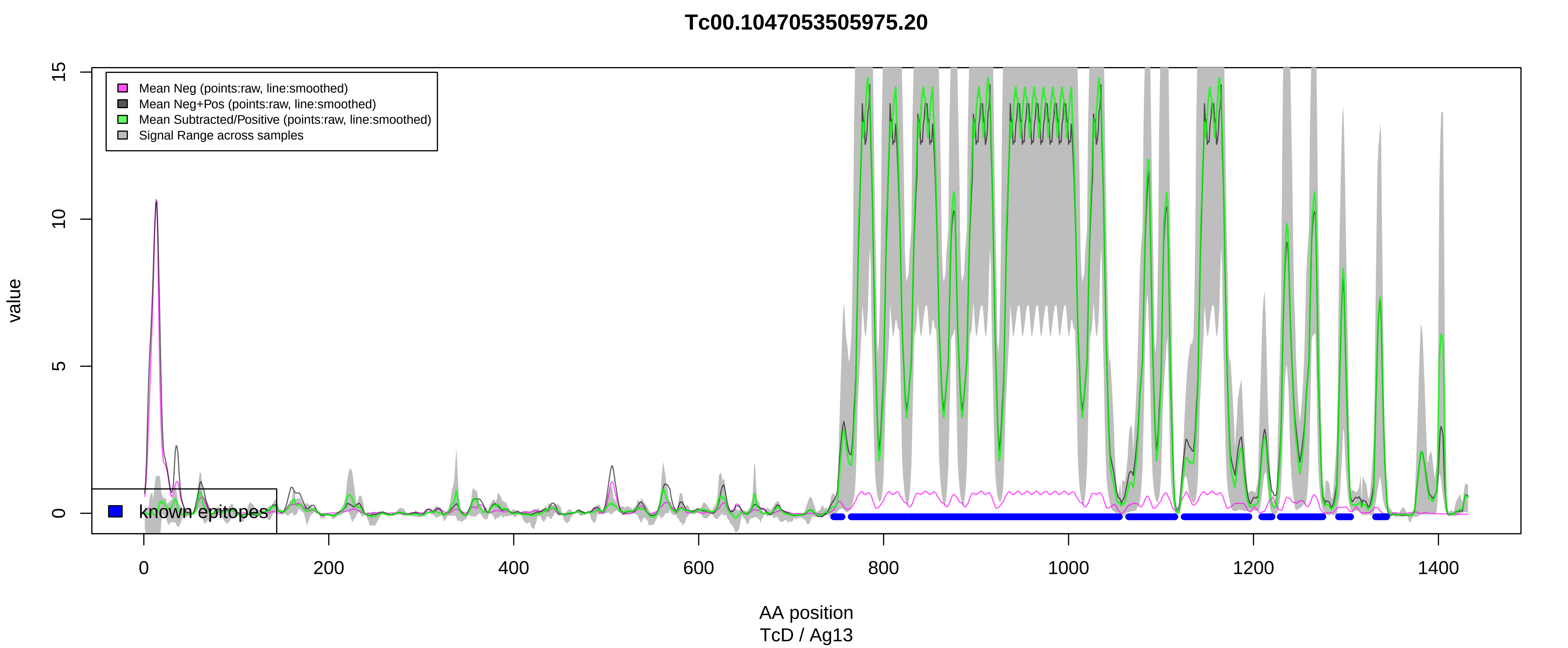

Tc00.1047053505975.20
Mean Neg (points:raw, line:smoothed)
Mean Neg+Pos (points:raw, line:smoothed)
Mean Subtracted/Positive (points:raw, line:smoothed)
Signal Range across samples
known epitopes
value
0
5
10
15
0
200
400
600
800
1000
1200
1400
AA position
TcD / Ag13

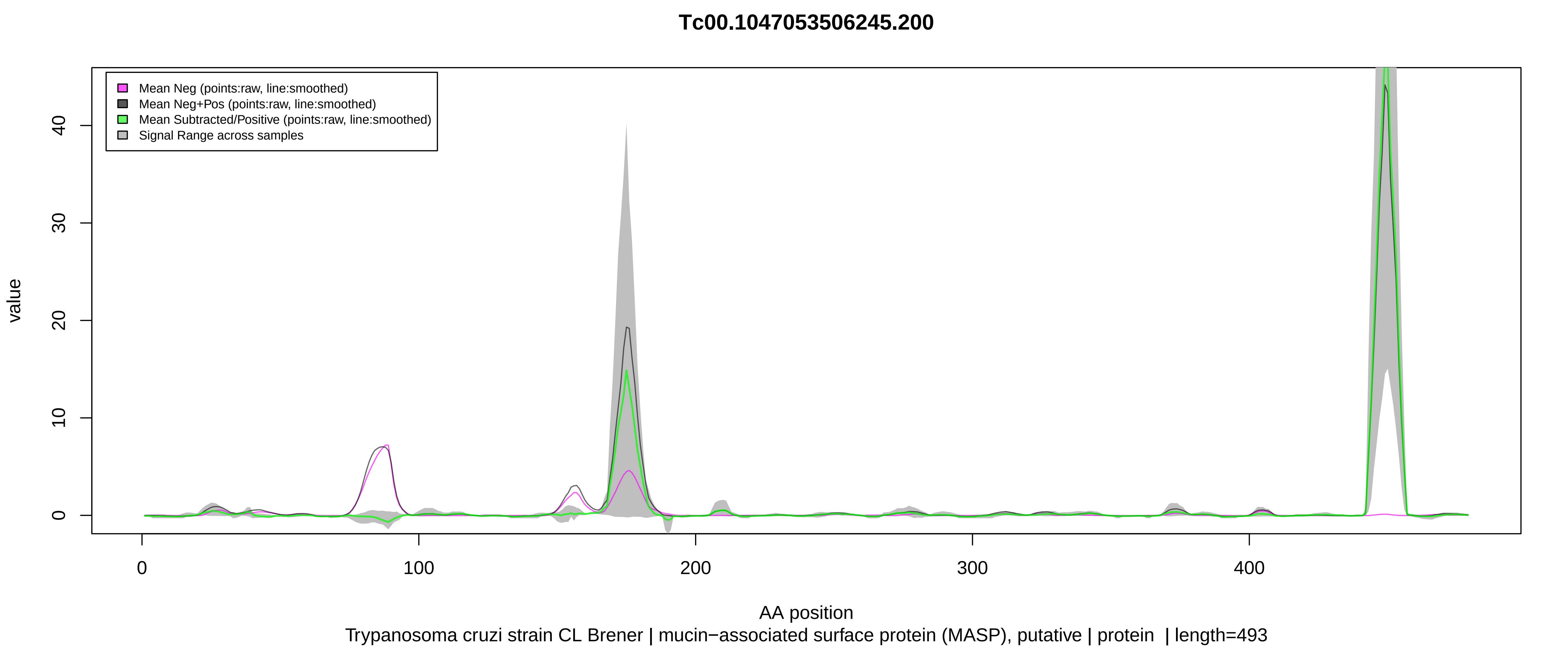

Tc00.1047053506245.200
Mean Neg (points:raw, line:smoothed)
Mean Neg+Pos (points:raw, line:smoothed)
Mean Subtracted/Positive (points:raw, line:smoothed)
Signal Range across samples
value
0
10
20
30
40
0
100
200
300
400
AA position
Trypanosoma cruzi strain CL Brener | mucin−associated surface protein (MASP), putative | protein | length=493

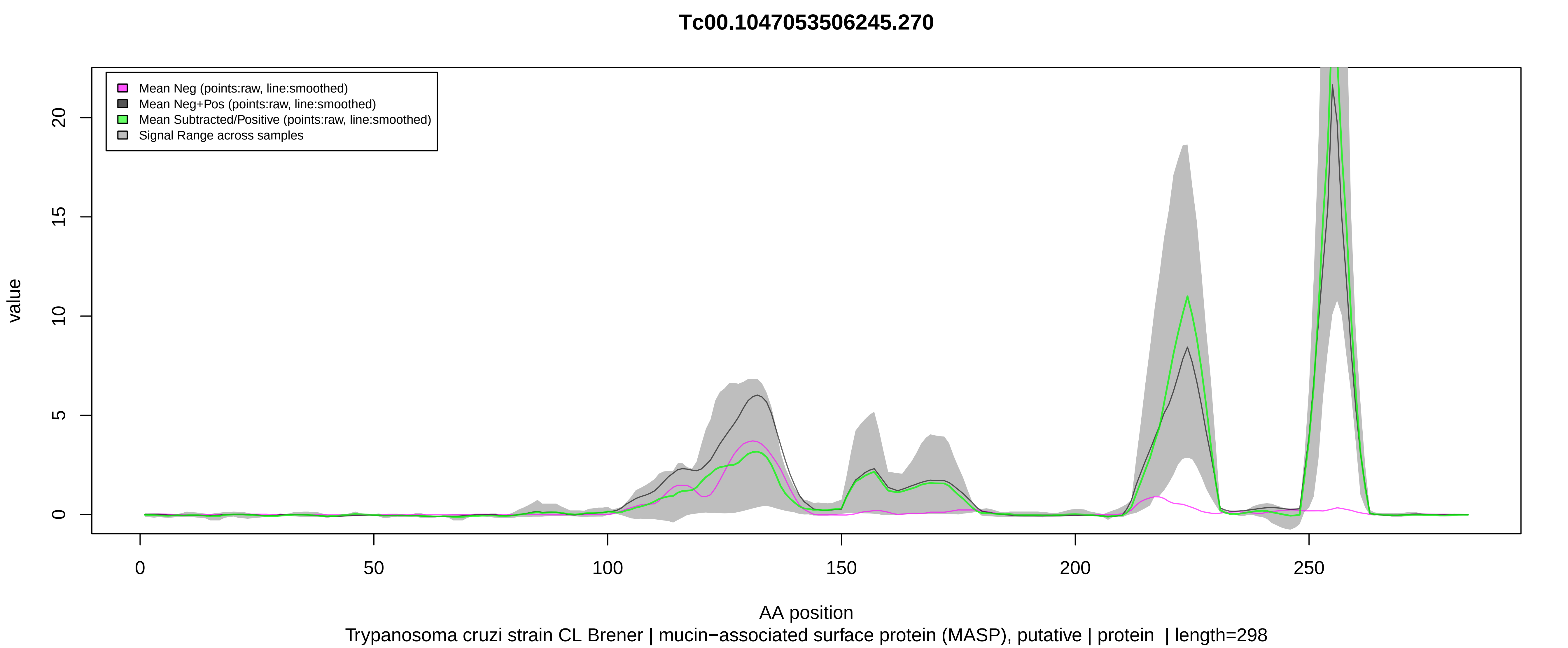
Tc00.1047053506245.270
Mean Neg (points:raw, line:smoothed)
Mean Neg+Pos (points:raw, line:smoothed)
Mean Subtracted/Positive (points:raw, line:smoothed)
Signal Range across samples
value
0
5
10
15
20
0
50
100
150
200
250
AA position
Trypanosoma cruzi strain CL Brener | mucin−associated surface protein (MASP), putative | protein | length=298

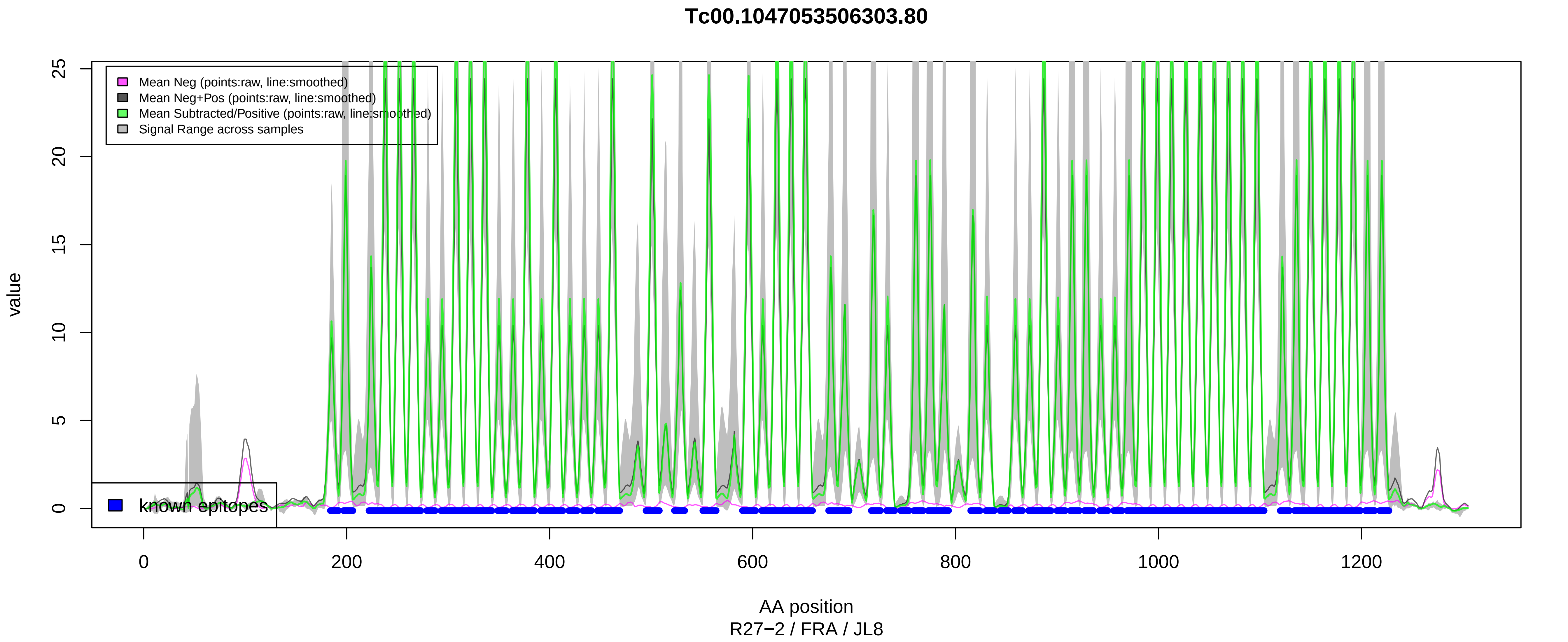

Tc00.1047053506303.80
Mean Neg (points:raw, line:smoothed)
Mean Neg+Pos (points:raw, line:smoothed)
Mean Subtracted/Positive (points:raw, line:smoothed)
Signal Range across samples
known epitopes
value
0
5
10
15
20
25
0
200
400
600
800
1000
1200
AA position
R27−2 / FRA / JL8

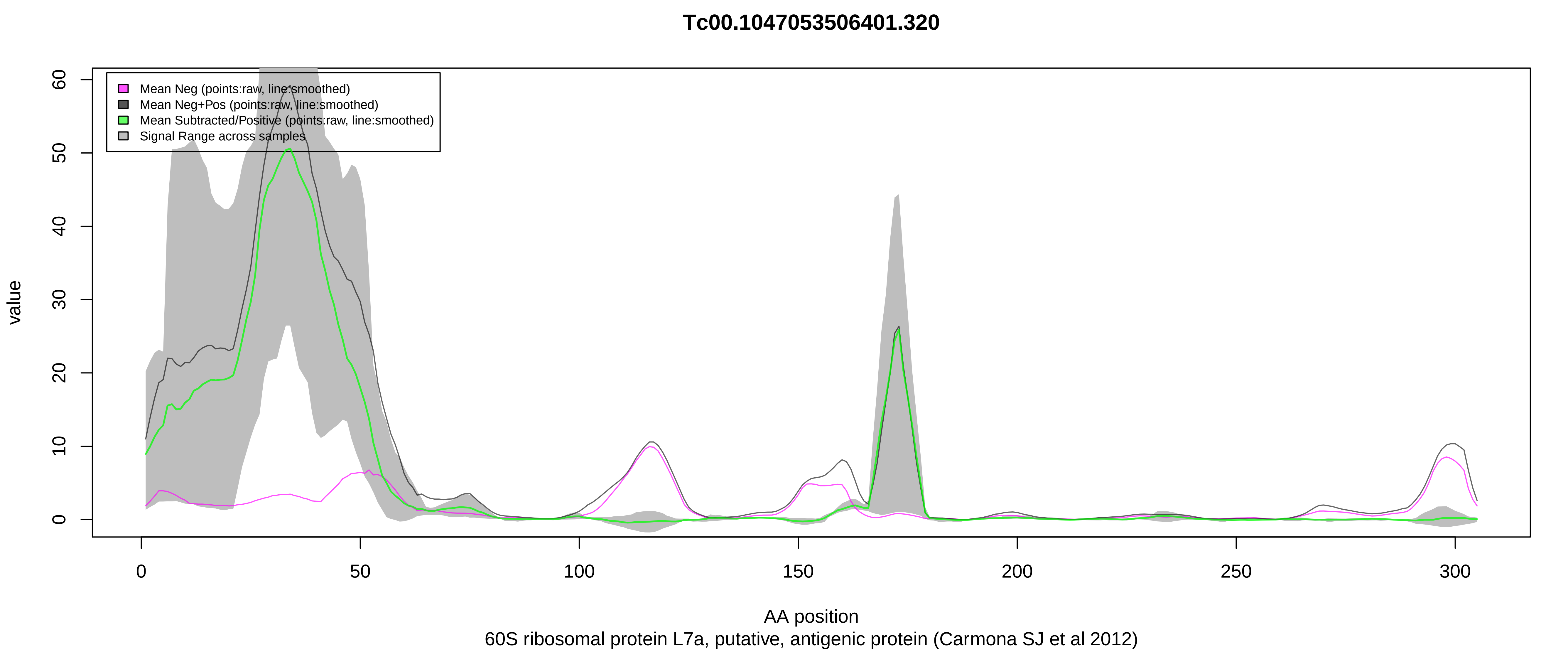
Tc00.1047053506401.320
Mean Neg (points:raw, line:smoothed)
Mean Neg+Pos (points:raw, line:smoothed)
Mean Subtracted/Positive (points:raw, line:smoothed)
Signal Range across samples
value
0
10
20
30
40
50
60
0
50
100
150
200
250
300
AA position
60S ribosomal protein L7a, putative, antigenic protein (Carmona SJ et al 2012)

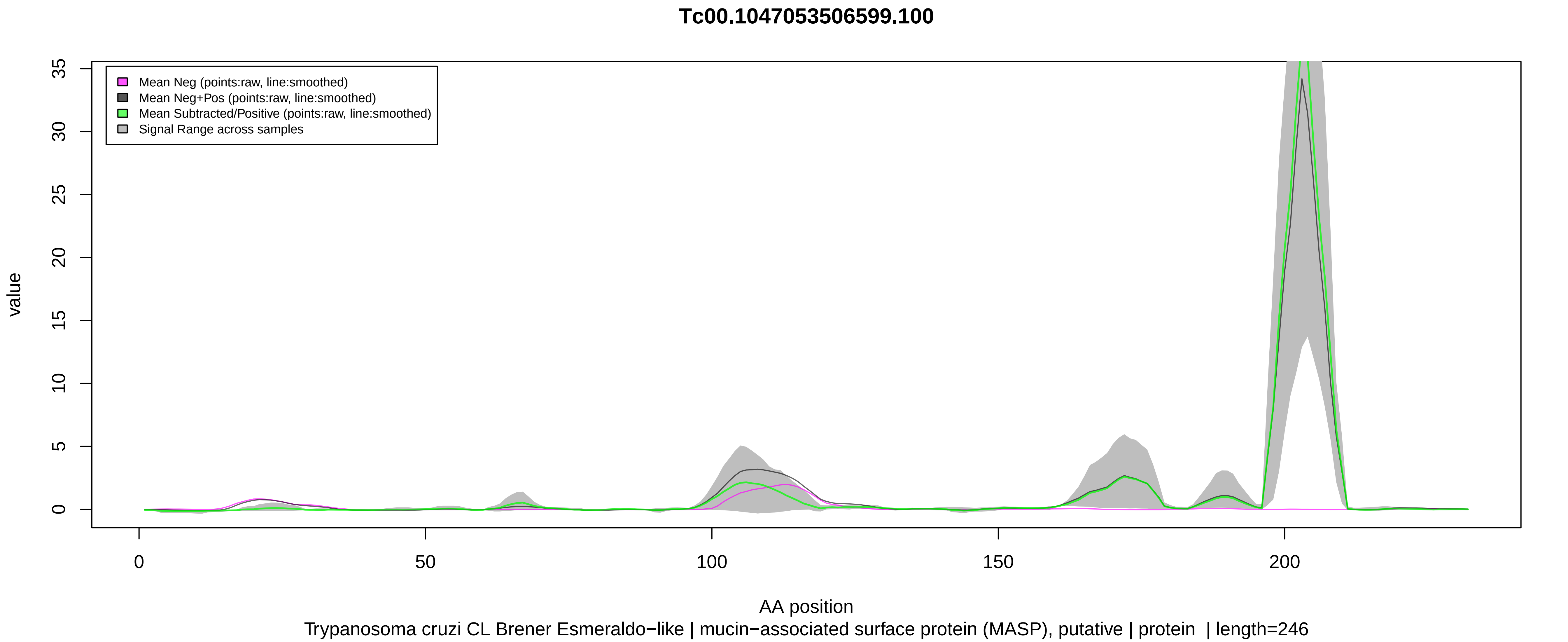

Tc00.1047053506599.100
Mean Neg (points:raw, line:smoothed)
Mean Neg+Pos (points:raw, line:smoothed)
Mean Subtracted/Positive (points:raw, line:smoothed)
Signal Range across samples
value
0
5
10
15
20
25
30
35
0
50
100
150
200
AA position
Trypanosoma cruzi CL Brener Esmeraldo−like | mucin−associated surface protein (MASP), putative | protein | length=246

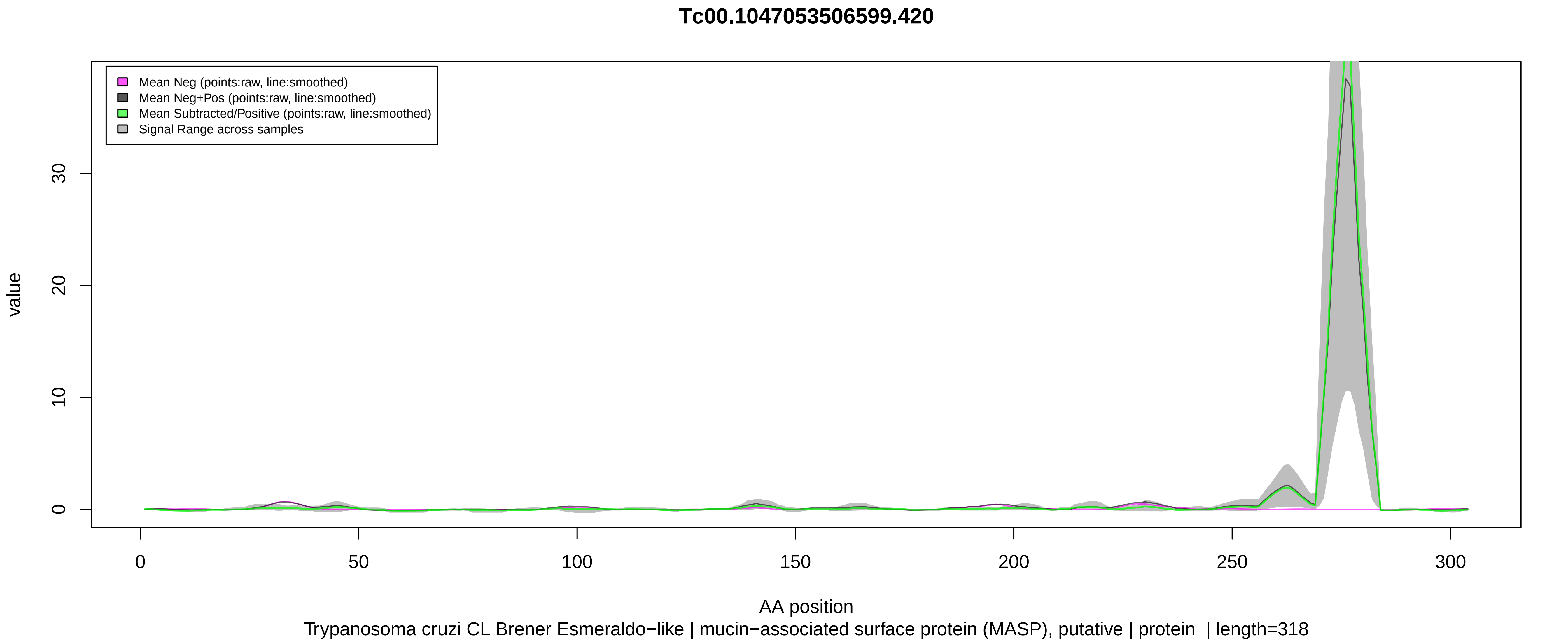

Tc00.1047053506599.420
Mean Neg (points:raw, line:smoothed)
Mean Neg+Pos (points:raw, line:smoothed)
Mean Subtracted/Positive (points:raw, line:smoothed)
Signal Range across samples
value
0
10
20
30
0
50
100
150
200
250
300
AA position
Trypanosoma cruzi CL Brener Esmeraldo−like | mucin−associated surface protein (MASP), putative | protein | length=318

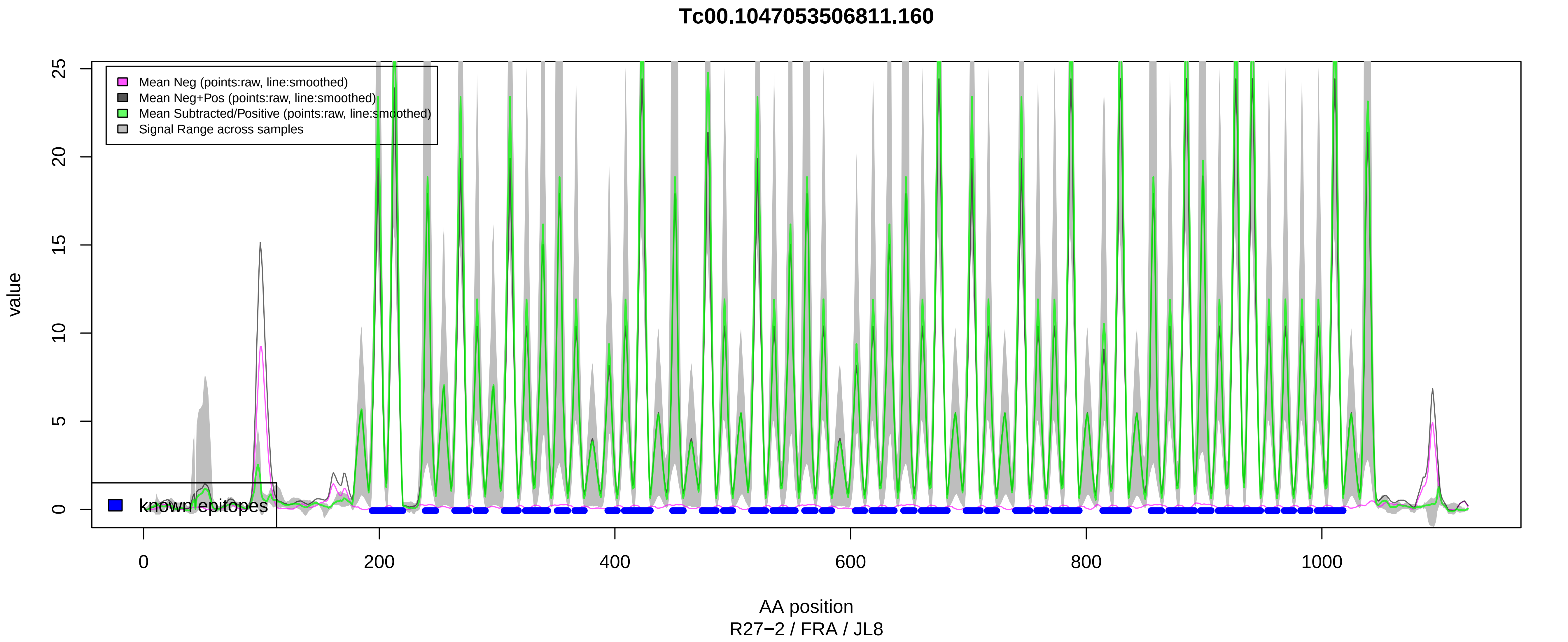

Tc00.1047053506811.160
Mean Neg (points:raw, line:smoothed)
Mean Neg+Pos (points:raw, line:smoothed)
Mean Subtracted/Positive (points:raw, line:smoothed)
Signal Range across samples
known epitopes
value
0
5
10
15
20
25
0
200
400
600
800
1000
AA position
R27−2 / FRA / JL8

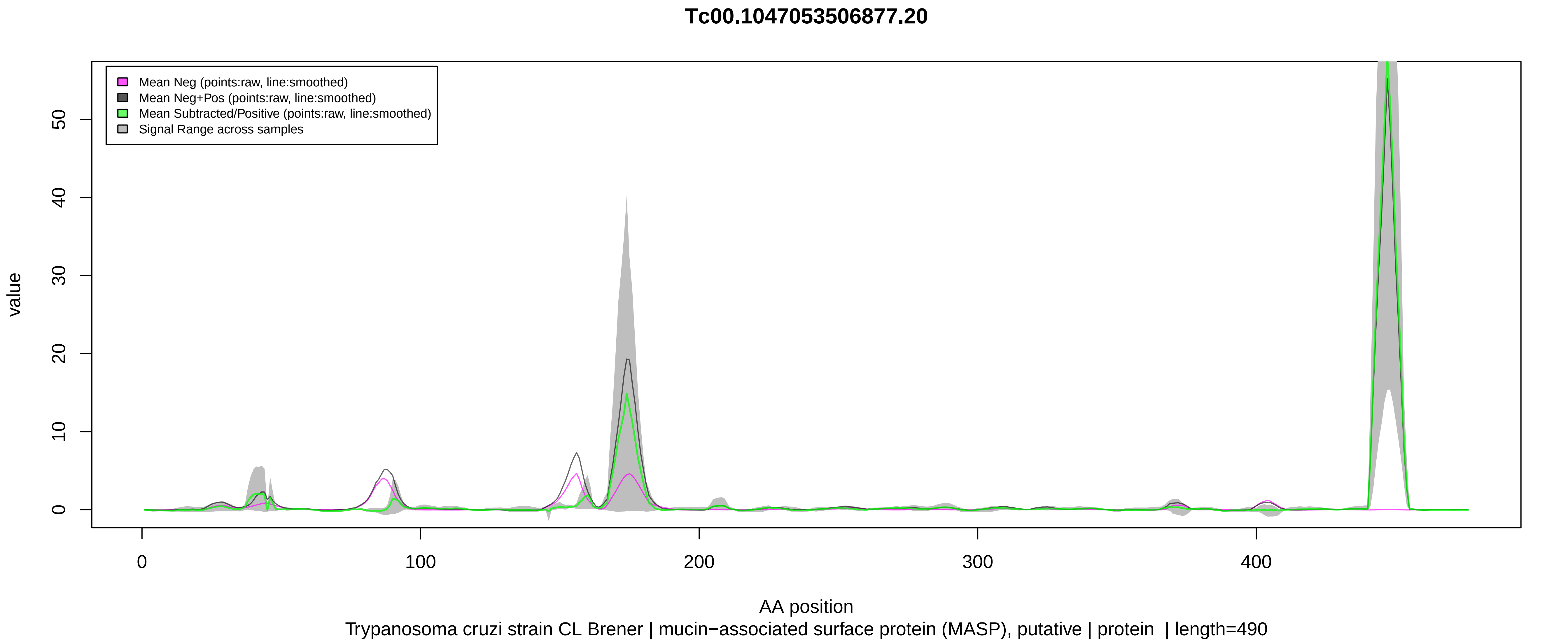

Tc00.1047053506877.20
Mean Neg (points:raw, line:smoothed)
Mean Neg+Pos (points:raw, line:smoothed)
Mean Subtracted/Positive (points:raw, line:smoothed)
Signal Range across samples
value
0
10
20
30
40
50
0
100
200
300
400
AA position
Trypanosoma cruzi strain CL Brener | mucin−associated surface protein (MASP), putative | protein | length=490

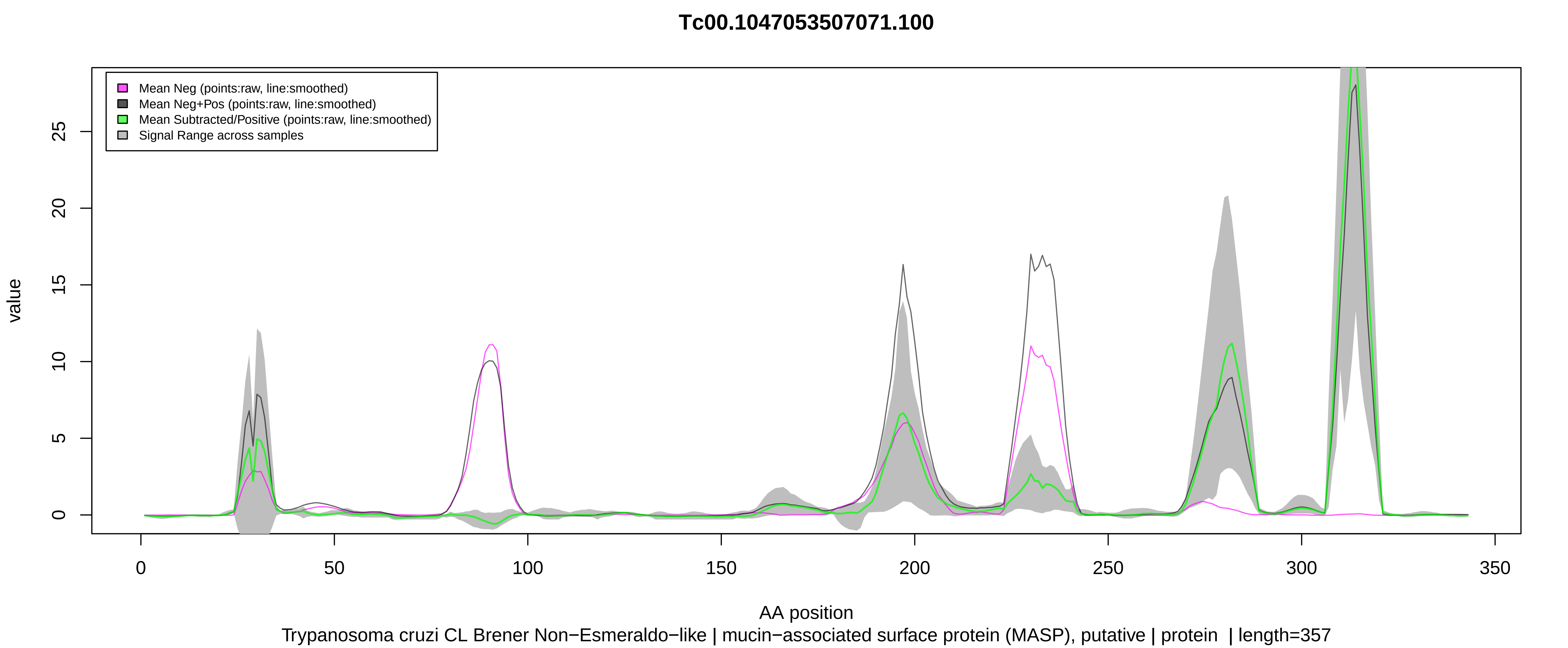
Tc00.1047053507071.100
Mean Neg (points:raw, line:smoothed)
Mean Neg+Pos (points:raw, line:smoothed)
Mean Subtracted/Positive (points:raw, line:smoothed)
Signal Range across samples
value
0
5
10
15
20
25
0
50
100
150
200
250
300
350
AA position
Trypanosoma cruzi CL Brener Non−Esmeraldo−like | mucin−associated surface protein (MASP), putative | protein | length=357

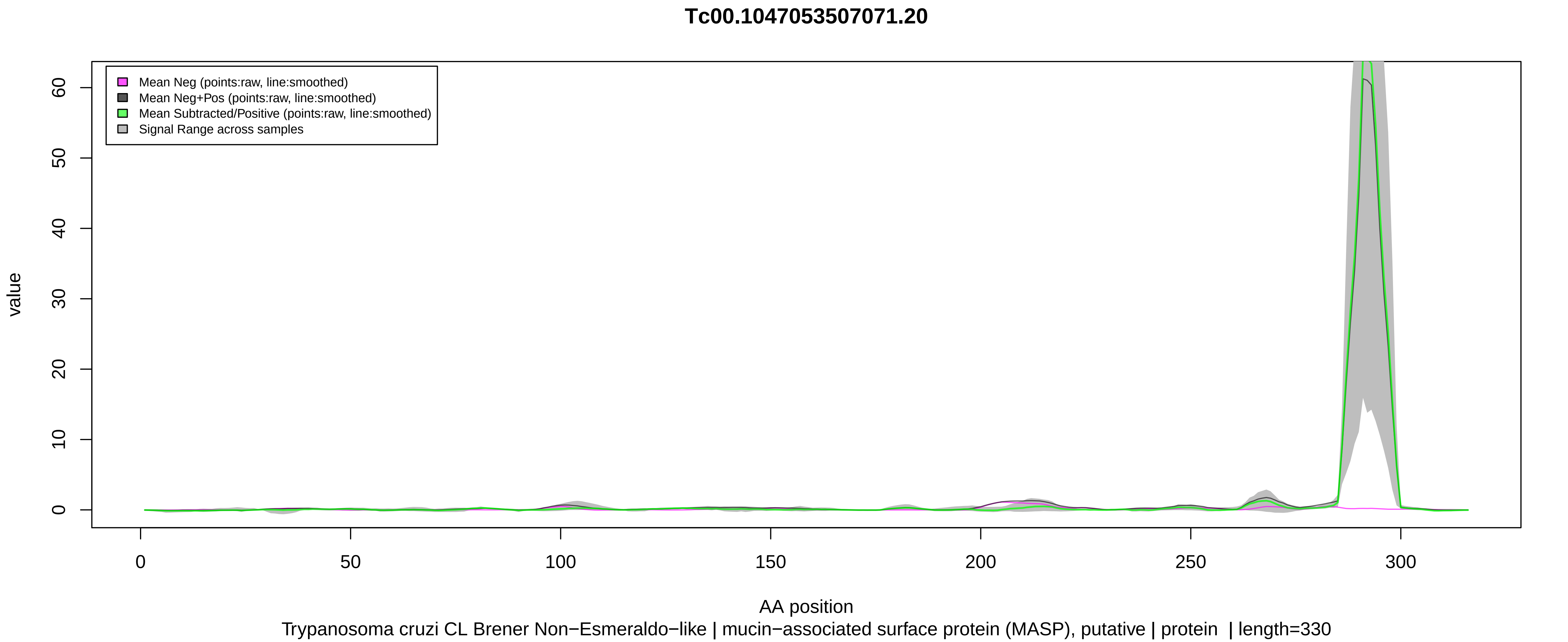

Tc00.1047053507071.20
Mean Neg (points:raw, line:smoothed)
Mean Neg+Pos (points:raw, line:smoothed)
Mean Subtracted/Positive (points:raw, line:smoothed)
Signal Range across samples
value
0
10
20
30
40
50
60
0
50
100
150
200
250
300
AA position
Trypanosoma cruzi CL Brener Non−Esmeraldo−like | mucin−associated surface protein (MASP), putative | protein | length=330

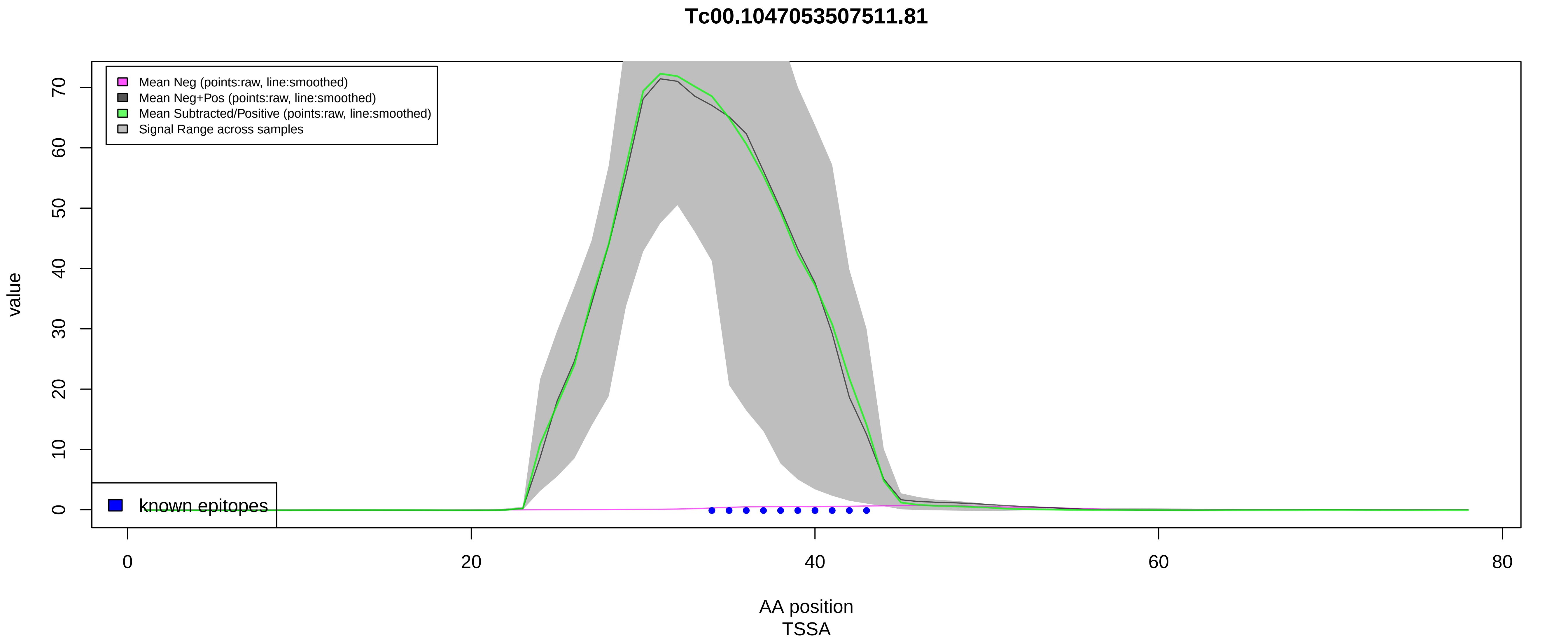

Tc00.1047053507511.81
Mean Neg (points:raw, line:smoothed)
Mean Neg+Pos (points:raw, line:smoothed)
Mean Subtracted/Positive (points:raw, line:smoothed)
Signal Range across samples
known epitopes
value
0
10
20
30
40
50
60
70
0
20
40
60
80
AA position
TSSA

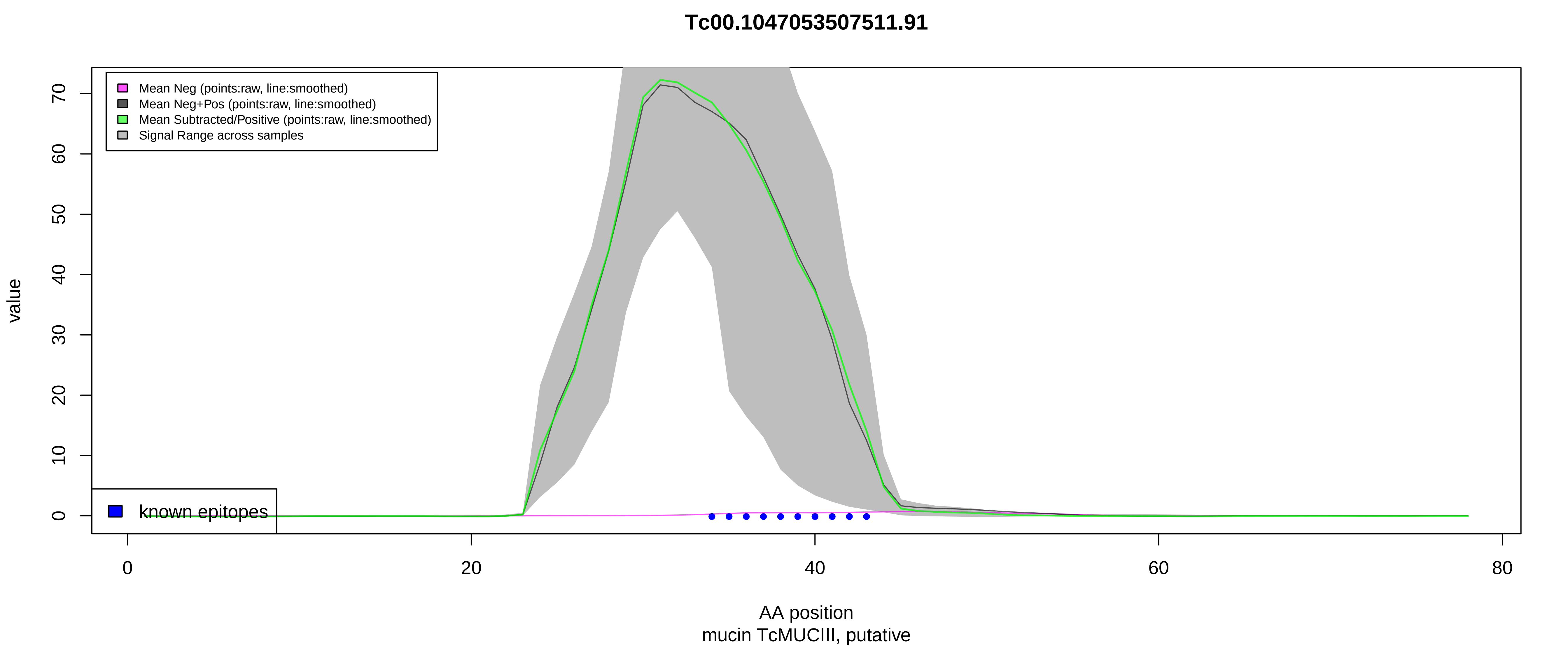
Tc00.1047053507511.91
Mean Neg (points:raw, line:smoothed)
Mean Neg+Pos (points:raw, line:smoothed)
Mean Subtracted/Positive (points:raw, line:smoothed)
Signal Range across samples
known epitopes
value
0
10
20
30
40
50
60
70
0
20
40
60
80
AA position
mucin TcMUCIII, putative

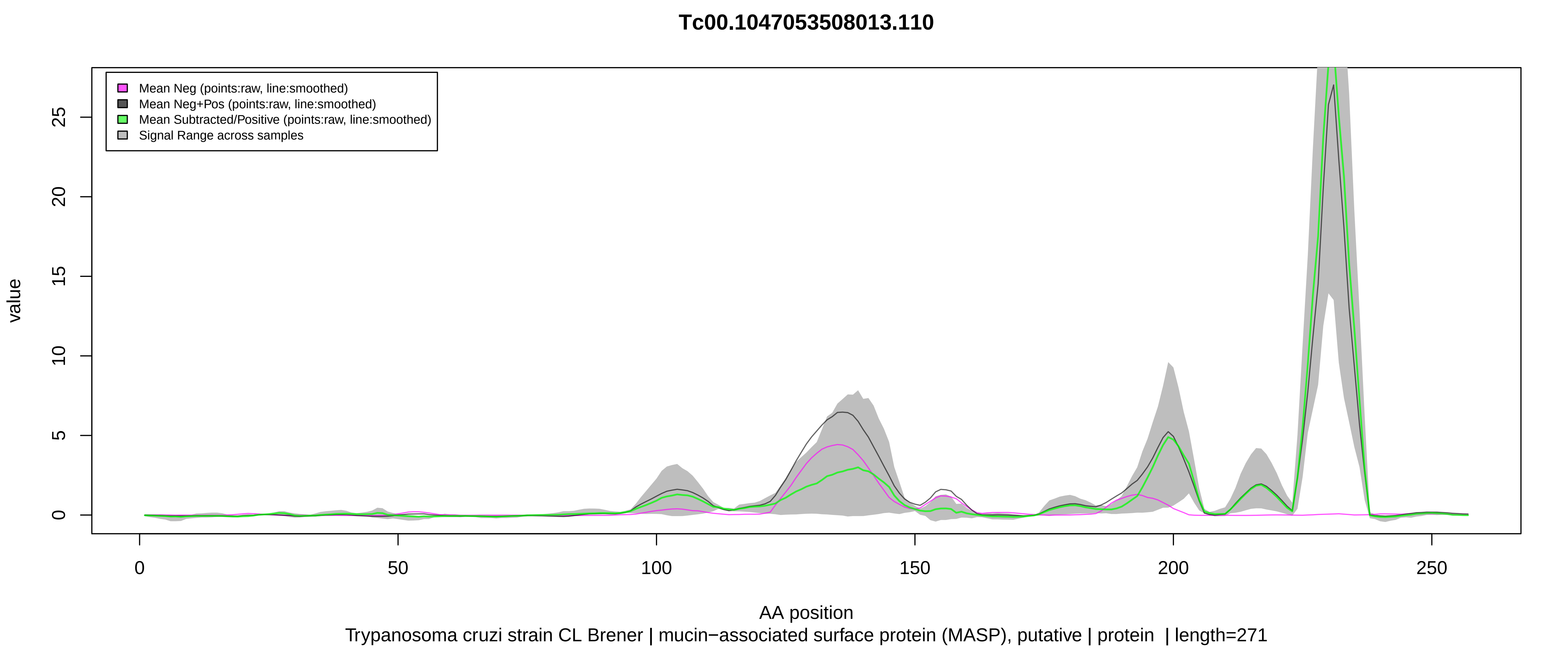
Tc00.1047053508013.110
Mean Neg (points:raw, line:smoothed)
Mean Neg+Pos (points:raw, line:smoothed)
Mean Subtracted/Positive (points:raw, line:smoothed)
Signal Range across samples
value
0
5
10
15
20
25
0
50
100
150
200
250
AA position
Trypanosoma cruzi strain CL Brener | mucin−associated surface protein (MASP), putative | protein | length=271

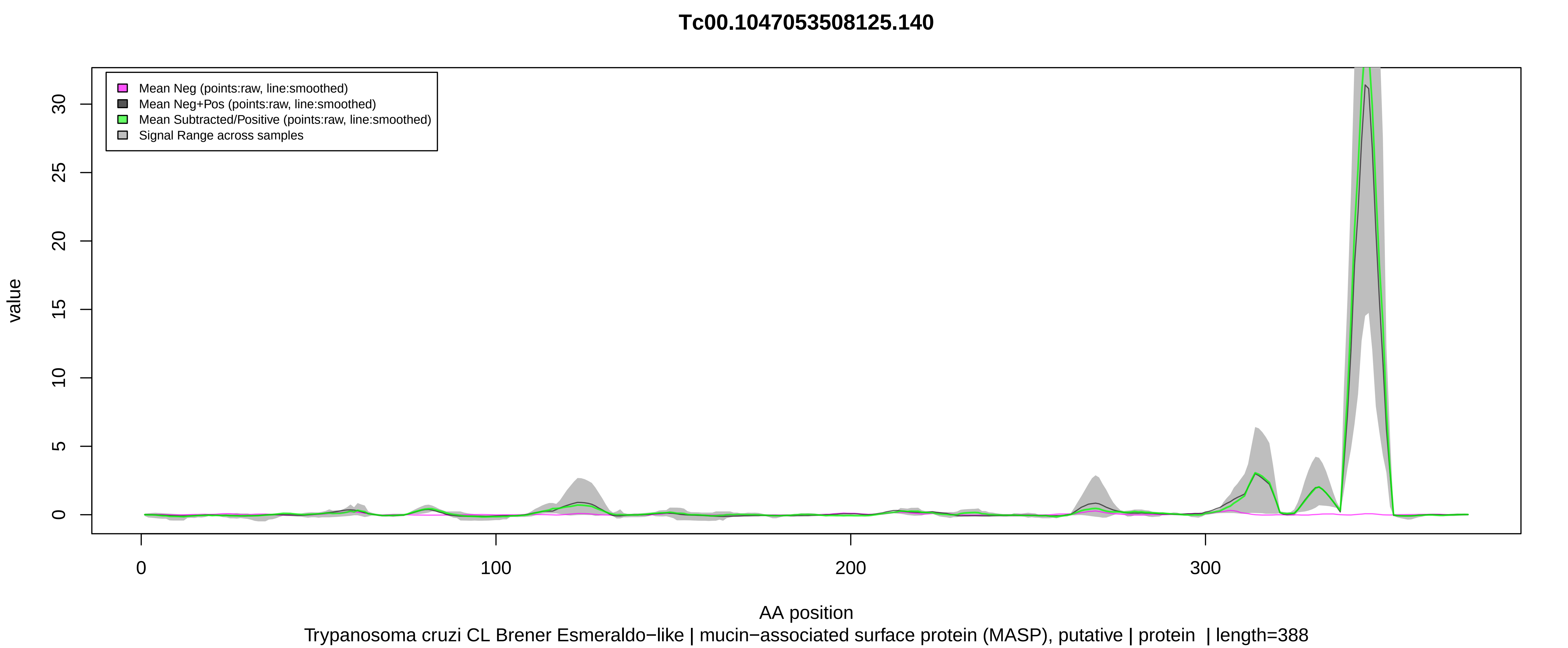

Tc00.1047053508125.140
Mean Neg (points:raw, line:smoothed)
Mean Neg+Pos (points:raw, line:smoothed)
Mean Subtracted/Positive (points:raw, line:smoothed)
Signal Range across samples
value
0
5
10
15
20
25
30
0
100
200
300
AA position
Trypanosoma cruzi CL Brener Esmeraldo−like | mucin−associated surface protein (MASP), putative | protein | length=388

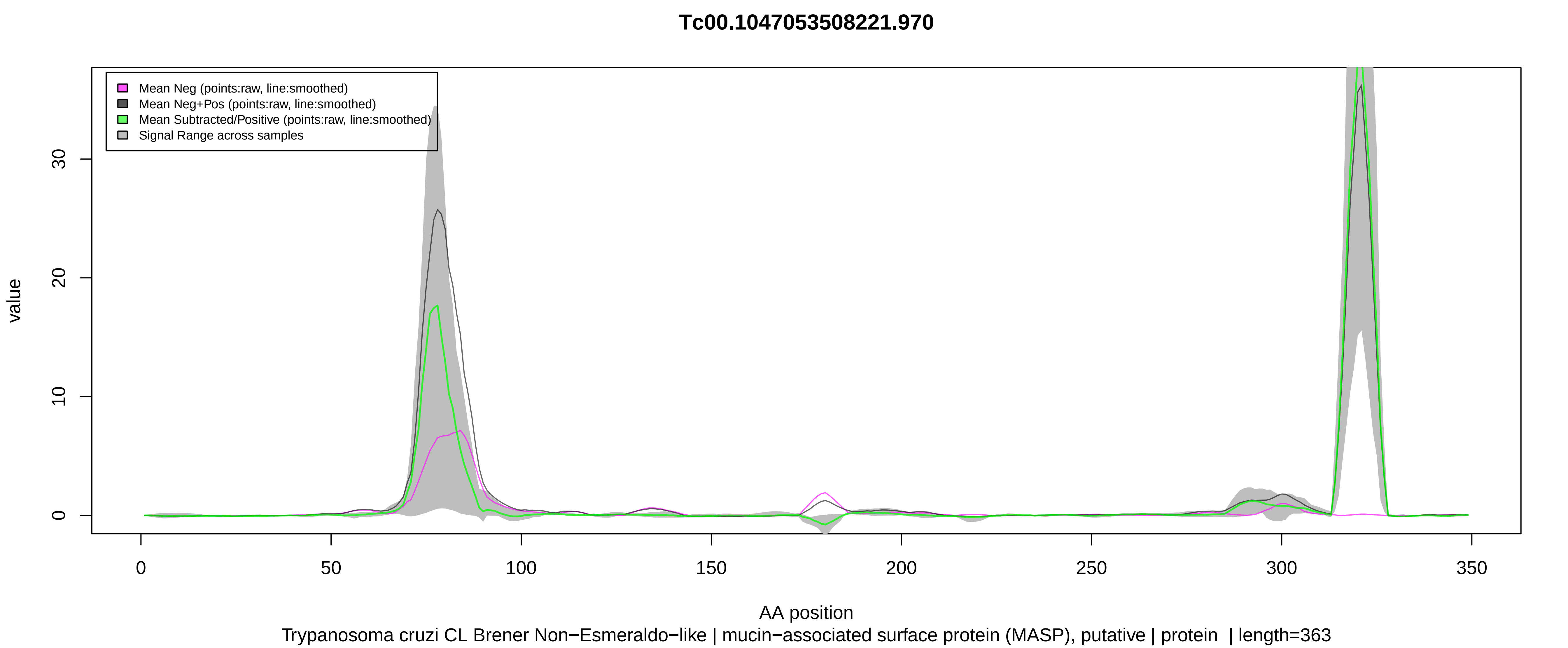

Tc00.1047053508221.970
Mean Neg (points:raw, line:smoothed)
Mean Neg+Pos (points:raw, line:smoothed)
Mean Subtracted/Positive (points:raw, line:smoothed)
Signal Range across samples
value
0
10
20
30
0
50
100
150
200
250
300
350
AA position
Trypanosoma cruzi CL Brener Non−Esmeraldo−like | mucin−associated surface protein (MASP), putative | protein | length=363

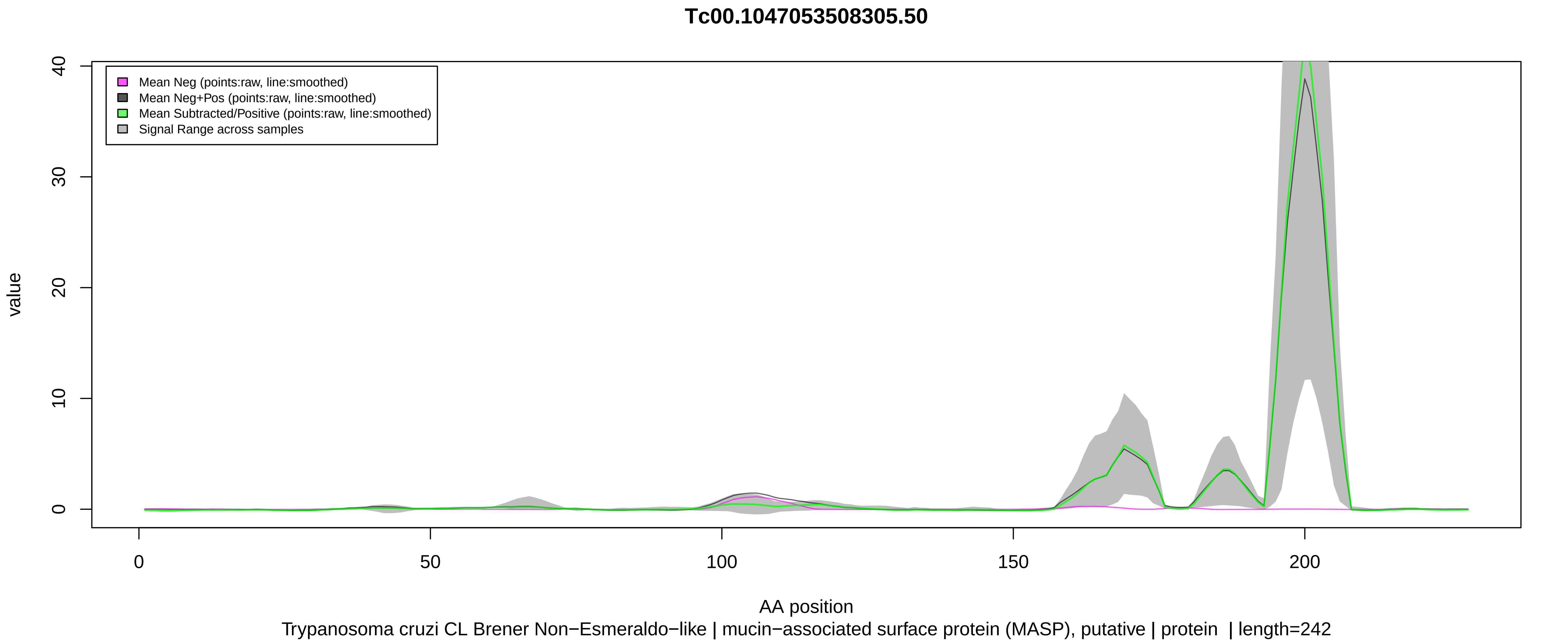

Tc00.1047053508305.50
Mean Neg (points:raw, line:smoothed)
Mean Neg+Pos (points:raw, line:smoothed)
Mean Subtracted/Positive (points:raw, line:smoothed)
Signal Range across samples
value
0
10
20
30
40
0
50
100
150
200
AA position
Trypanosoma cruzi CL Brener Non−Esmeraldo−like | mucin−associated surface protein (MASP), putative | protein | length=242

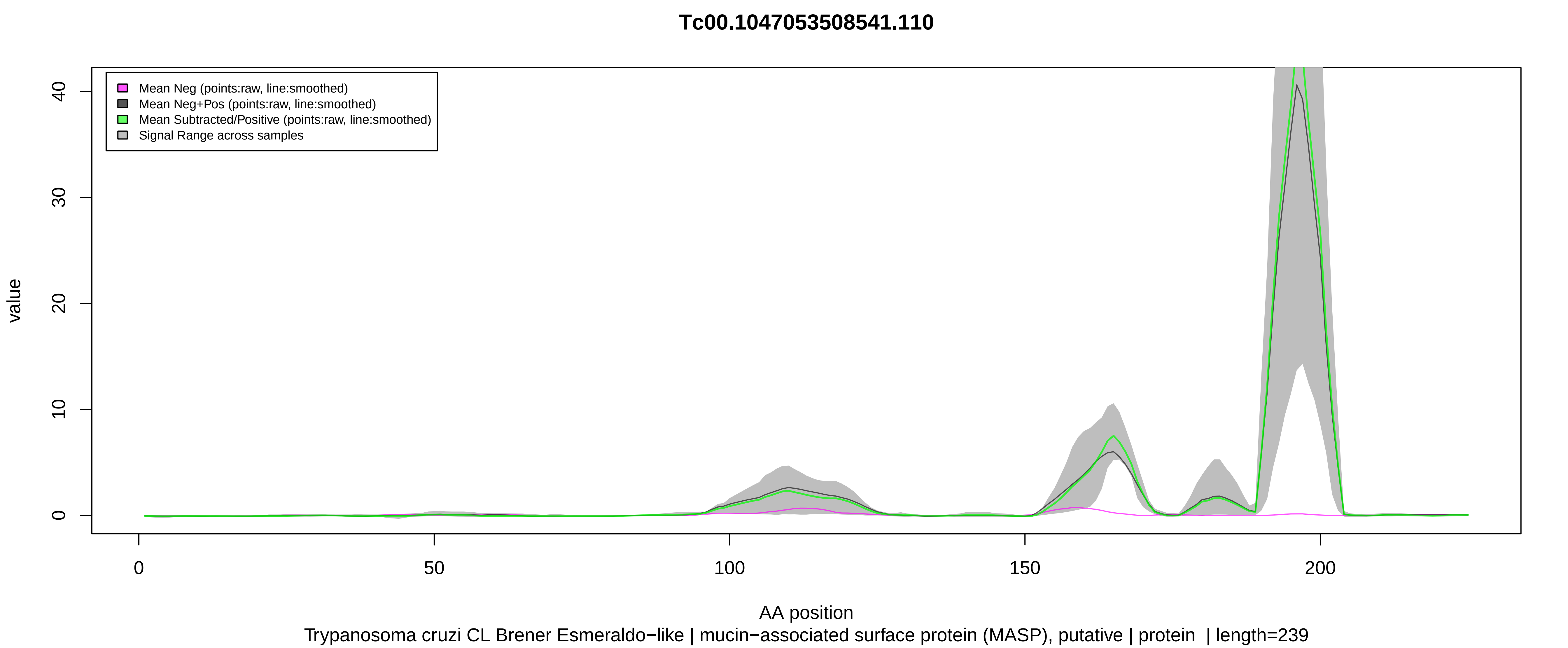

Tc00.1047053508541.110
Mean Neg (points:raw, line:smoothed)
Mean Neg+Pos (points:raw, line:smoothed)
Mean Subtracted/Positive (points:raw, line:smoothed)
Signal Range across samples
value
0
10
20
30
40
0
50
100
150
200
AA position
Trypanosoma cruzi CL Brener Esmeraldo−like | mucin−associated surface protein (MASP), putative | protein | length=239

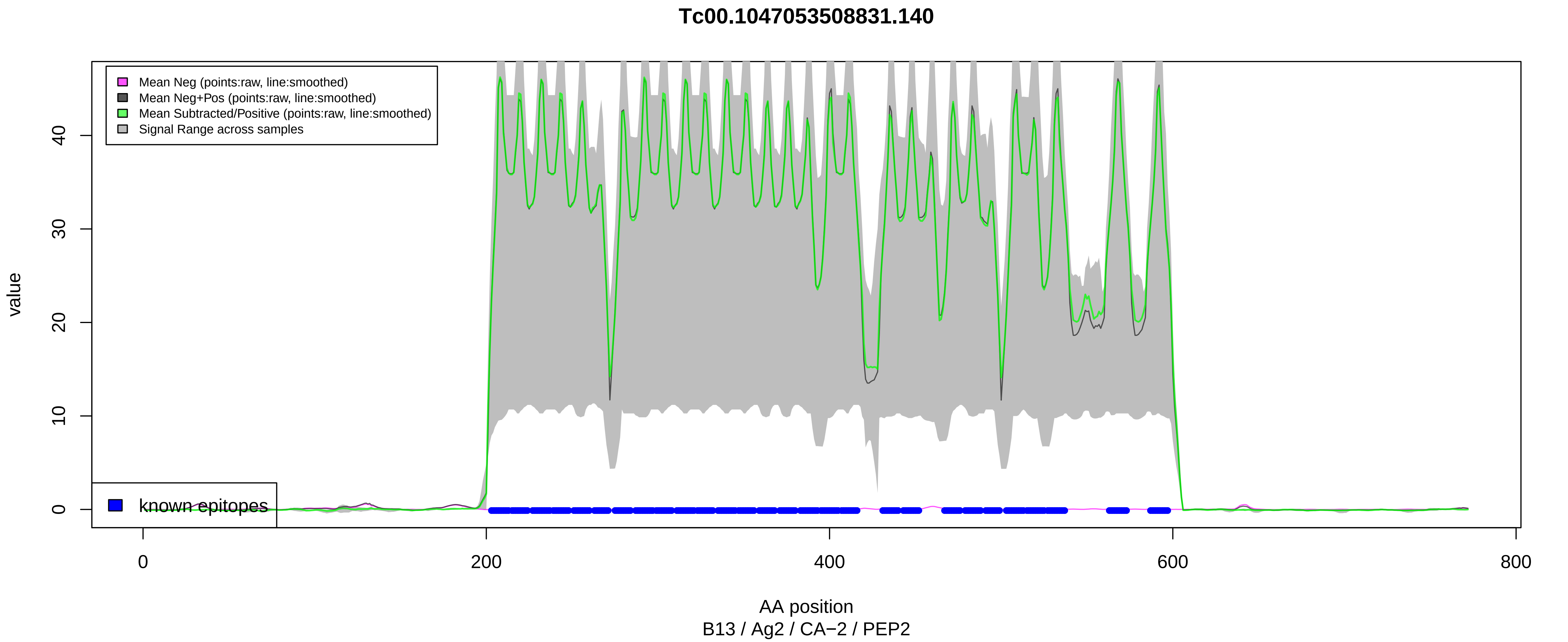
Tc00.1047053508831.140
Mean Neg (points:raw, line:smoothed)
Mean Neg+Pos (points:raw, line:smoothed)
Mean Subtracted/Positive (points:raw, line:smoothed)
Signal Range across samples
known epitopes
value
0
10
20
30
40
0
200
400
600
800
AA position
B13 / Ag2 / CA−2 / PEP2

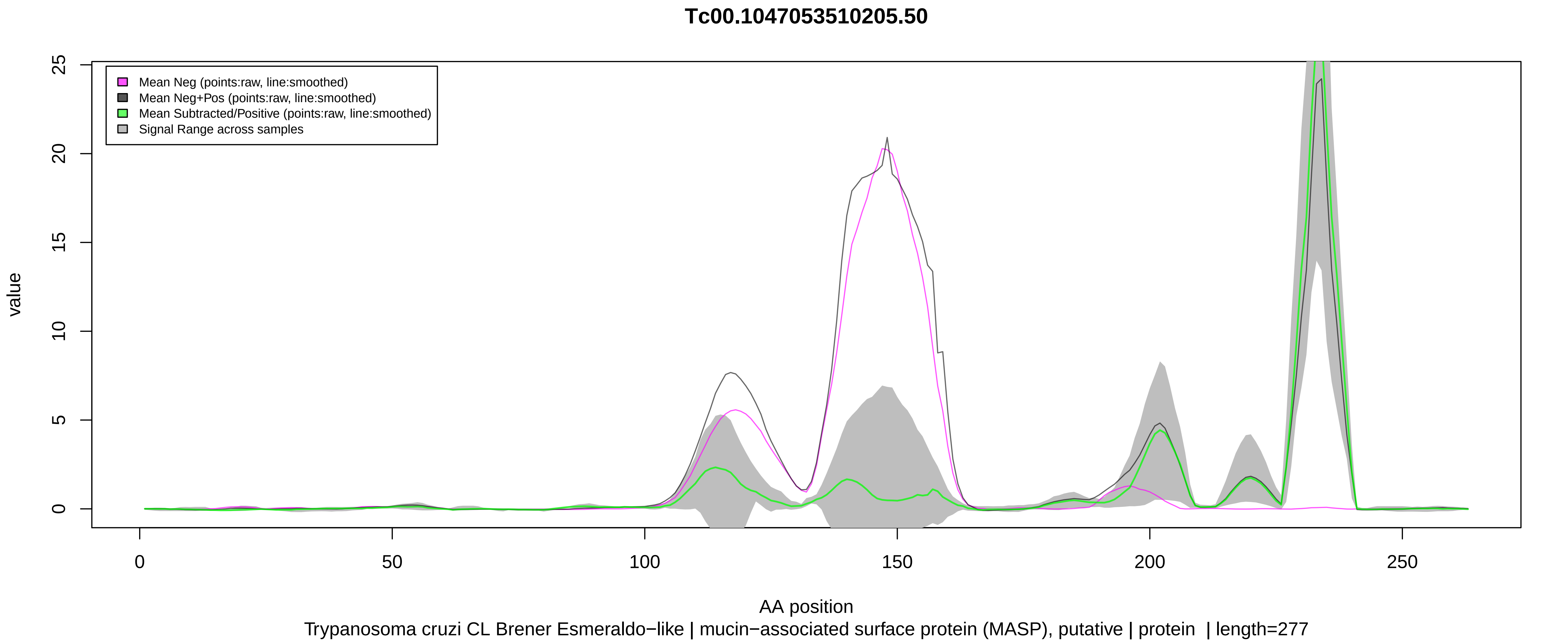
Tc00.1047053510205.50
Mean Neg (points:raw, line:smoothed)
Mean Neg+Pos (points:raw, line:smoothed)
Mean Subtracted/Positive (points:raw, line:smoothed)
Signal Range across samples
value
0
5
10
15
20
25
0
50
100
150
200
250
AA position
Trypanosoma cruzi CL Brener Esmeraldo−like | mucin−associated surface protein (MASP), putative | protein | length=277

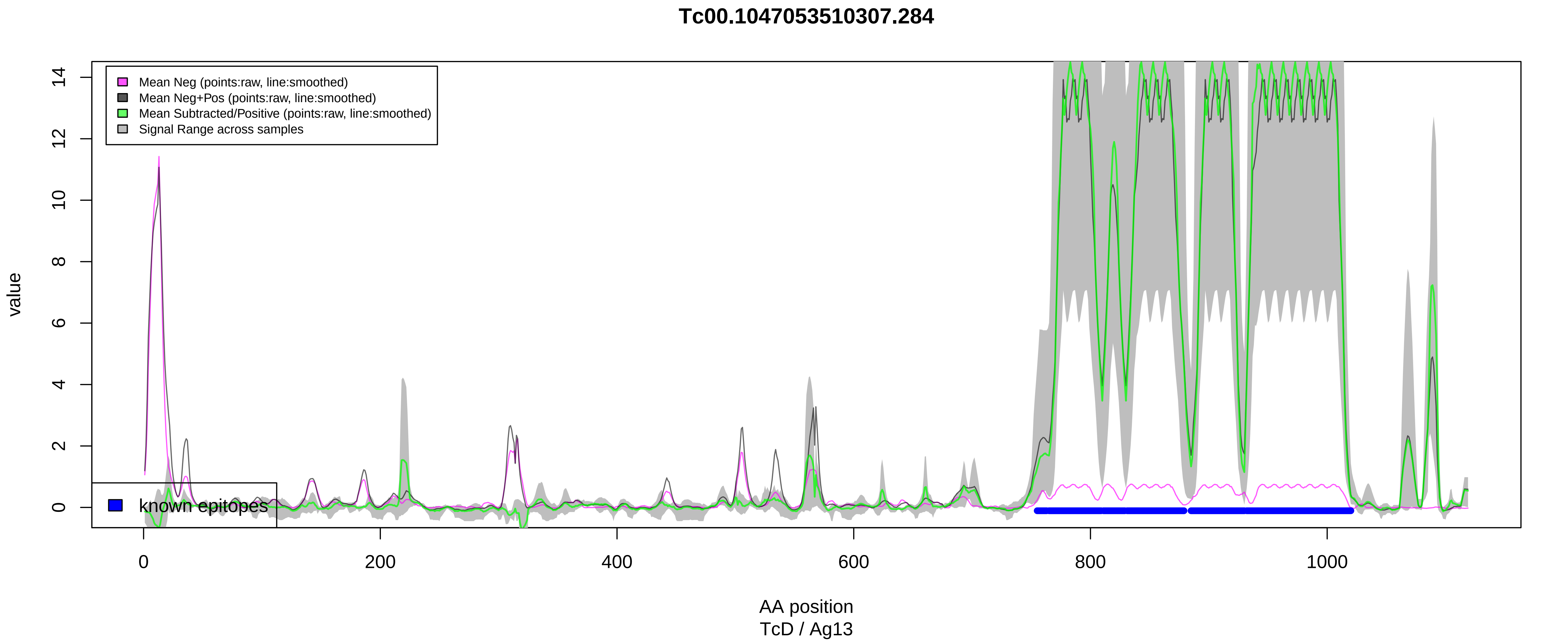

Tc00.1047053510307.284
Mean Neg (points:raw, line:smoothed)
Mean Neg+Pos (points:raw, line:smoothed)
Mean Subtracted/Positive (points:raw, line:smoothed)
Signal Range across samples
known epitopes
value
0
2
4
6
8
10
12
14
0
200
400
600
800
1000
AA position
TcD / Ag13

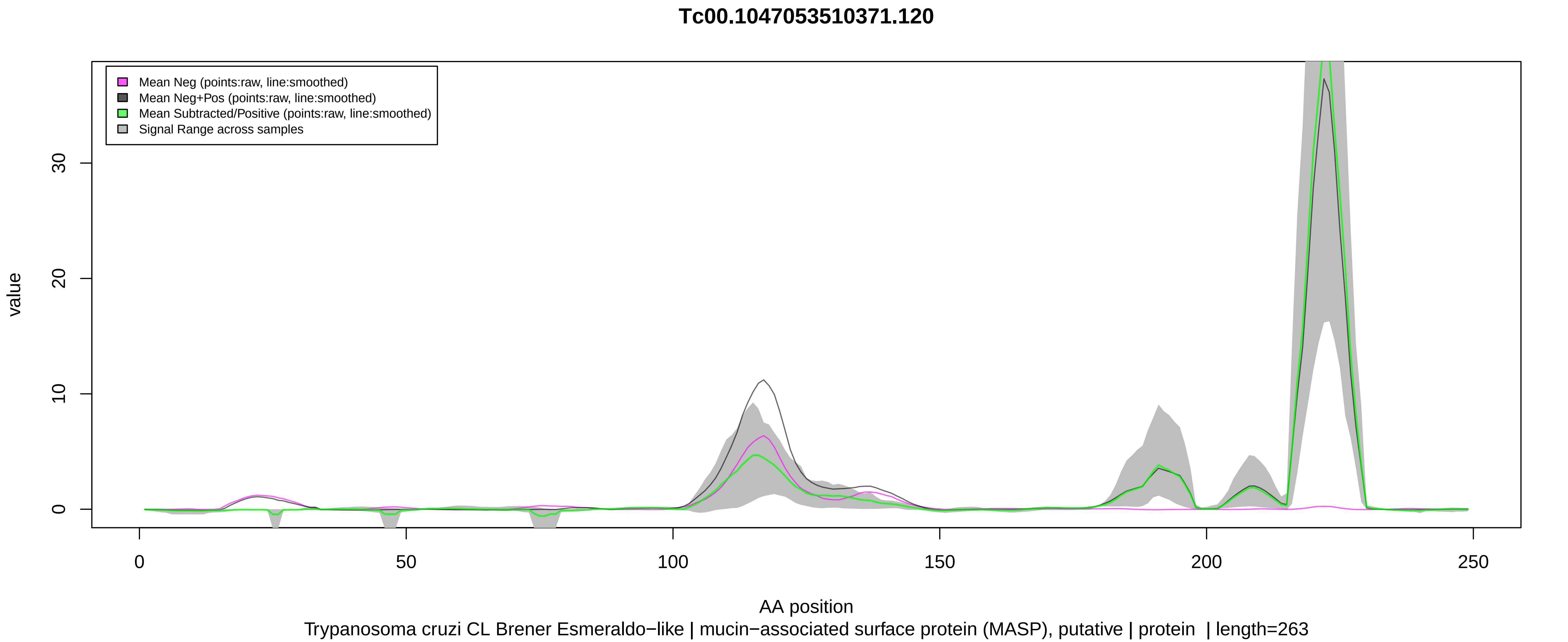

Tc00.1047053510371.120
Mean Neg (points:raw, line:smoothed)
Mean Neg+Pos (points:raw, line:smoothed)
Mean Subtracted/Positive (points:raw, line:smoothed)
Signal Range across samples
value
0
10
20
30
0
50
100
150
200
250
AA position
Trypanosoma cruzi CL Brener Esmeraldo−like | mucin−associated surface protein (MASP), putative | protein | length=263

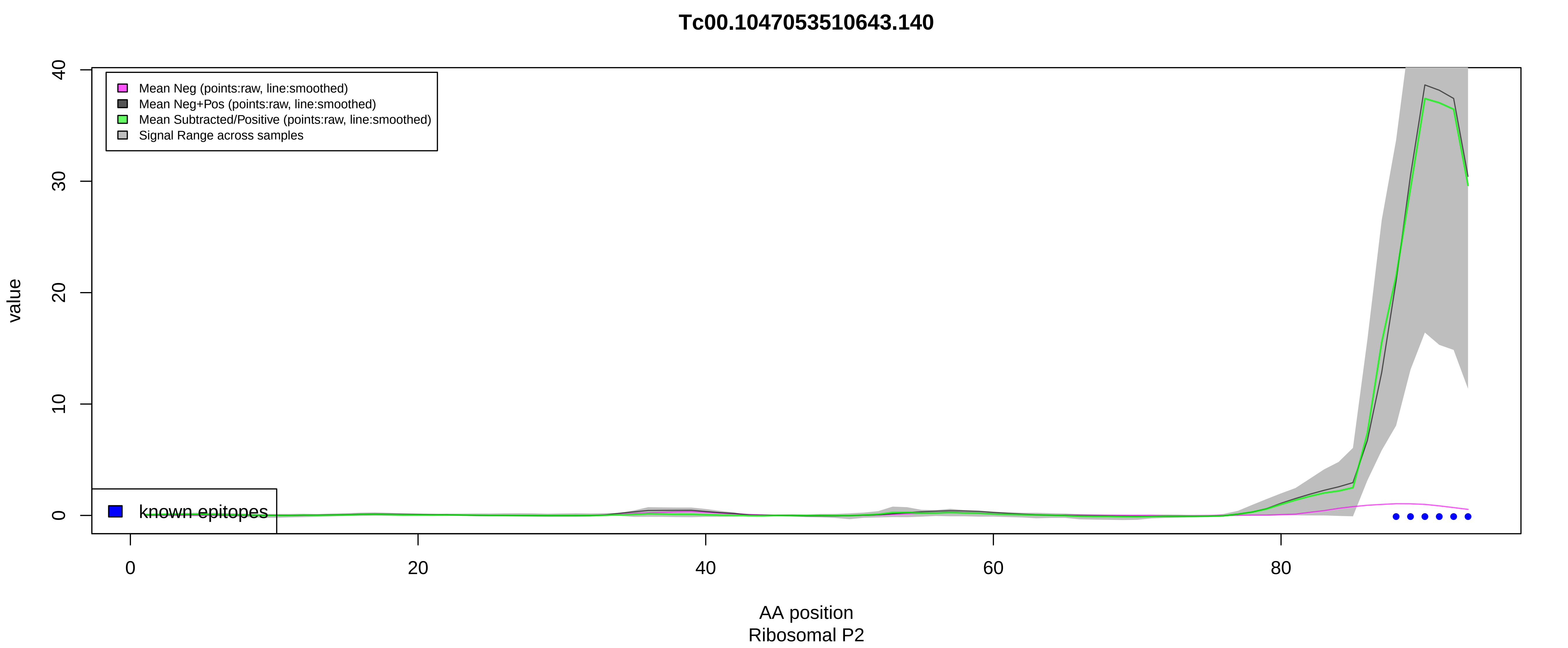

Tc00.1047053510643.140
Mean Neg (points:raw, line:smoothed)
Mean Neg+Pos (points:raw, line:smoothed)
Mean Subtracted/Positive (points:raw, line:smoothed)
Signal Range across samples
known epitopes
value
0
10
20
30
40
0
20
40
60
80
AA position
Ribosomal P2

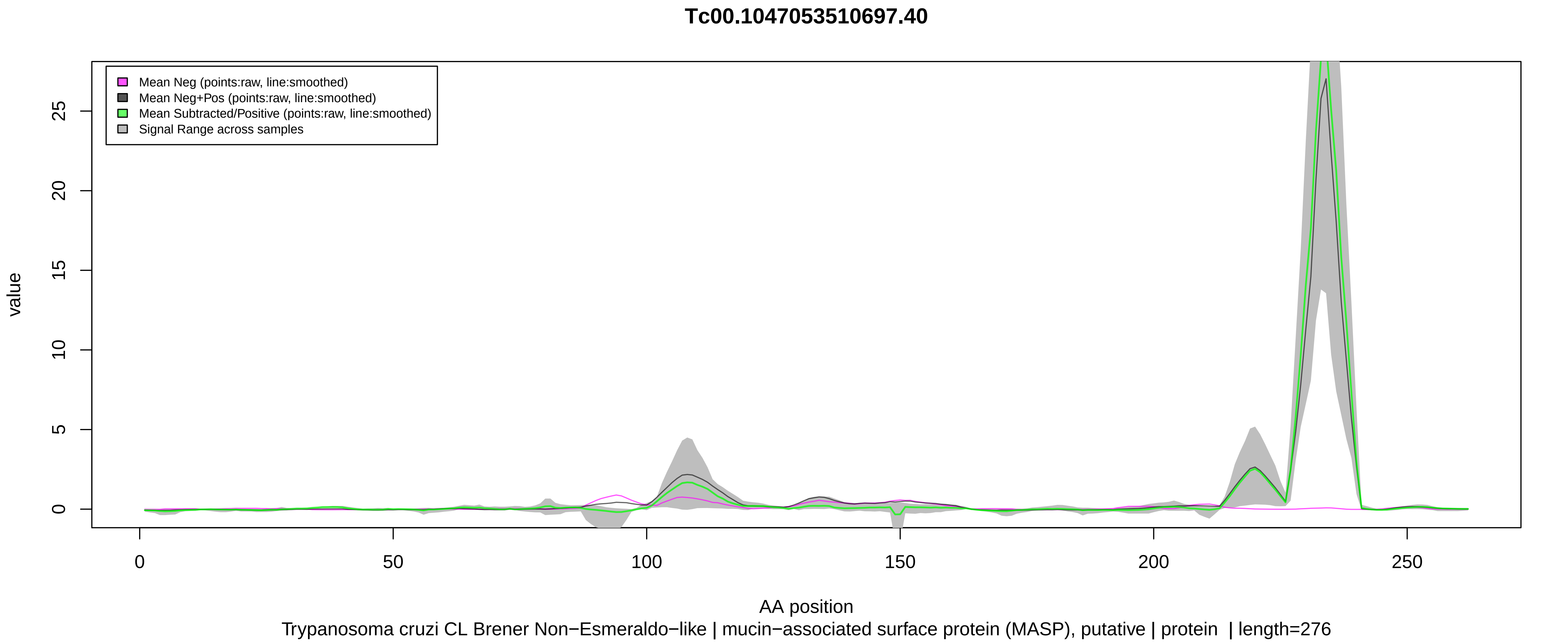

Tc00.1047053510697.40
Mean Neg (points:raw, line:smoothed)
Mean Neg+Pos (points:raw, line:smoothed)
Mean Subtracted/Positive (points:raw, line:smoothed)
Signal Range across samples
value
0
5
10
15
20
25
0
50
100
150
200
250
AA position
Trypanosoma cruzi CL Brener Non−Esmeraldo−like | mucin−associated surface protein (MASP), putative | protein | length=276

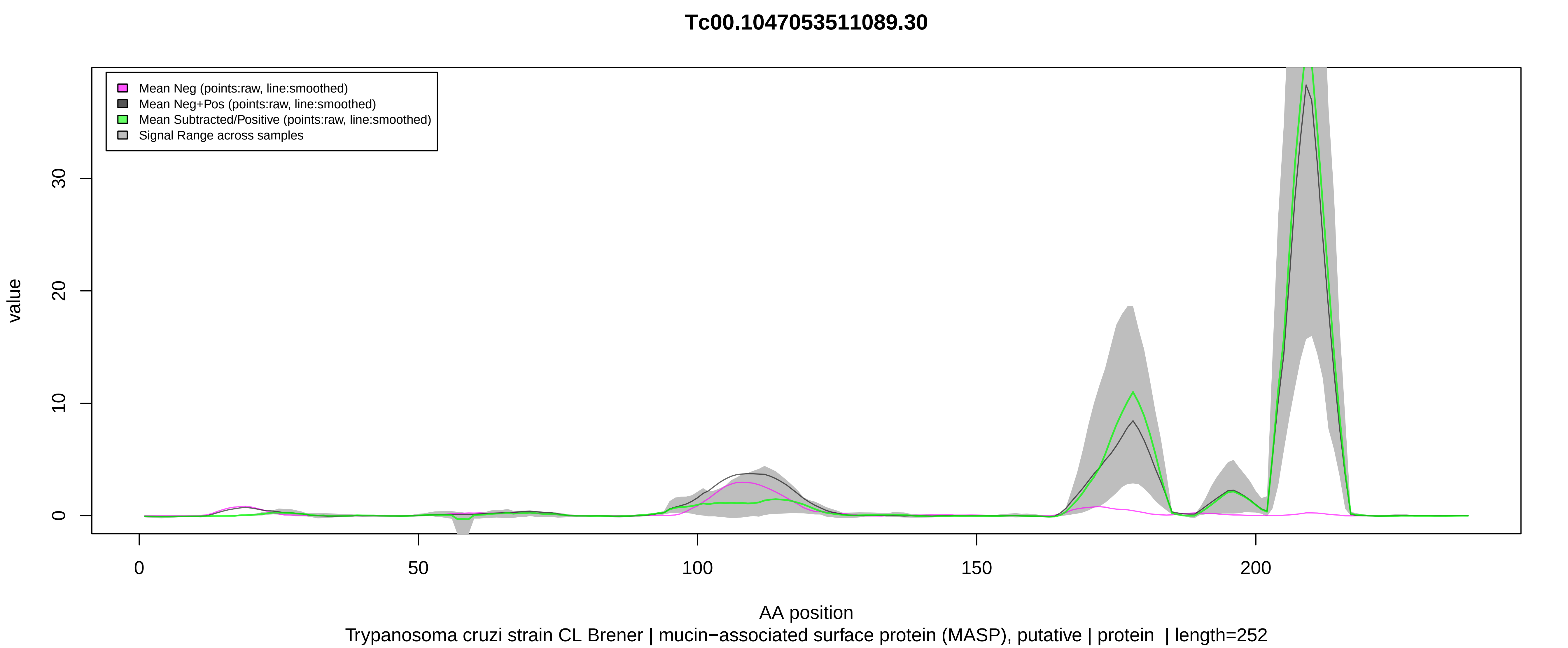
Tc00.1047053511089.30
Mean Neg (points:raw, line:smoothed)
Mean Neg+Pos (points:raw, line:smoothed)
Mean Subtracted/Positive (points:raw, line:smoothed)
Signal Range across samples
value
0
10
20
30
0
50
100
150
200
AA position
Trypanosoma cruzi strain CL Brener | mucin−associated surface protein (MASP), putative | protein | length=252

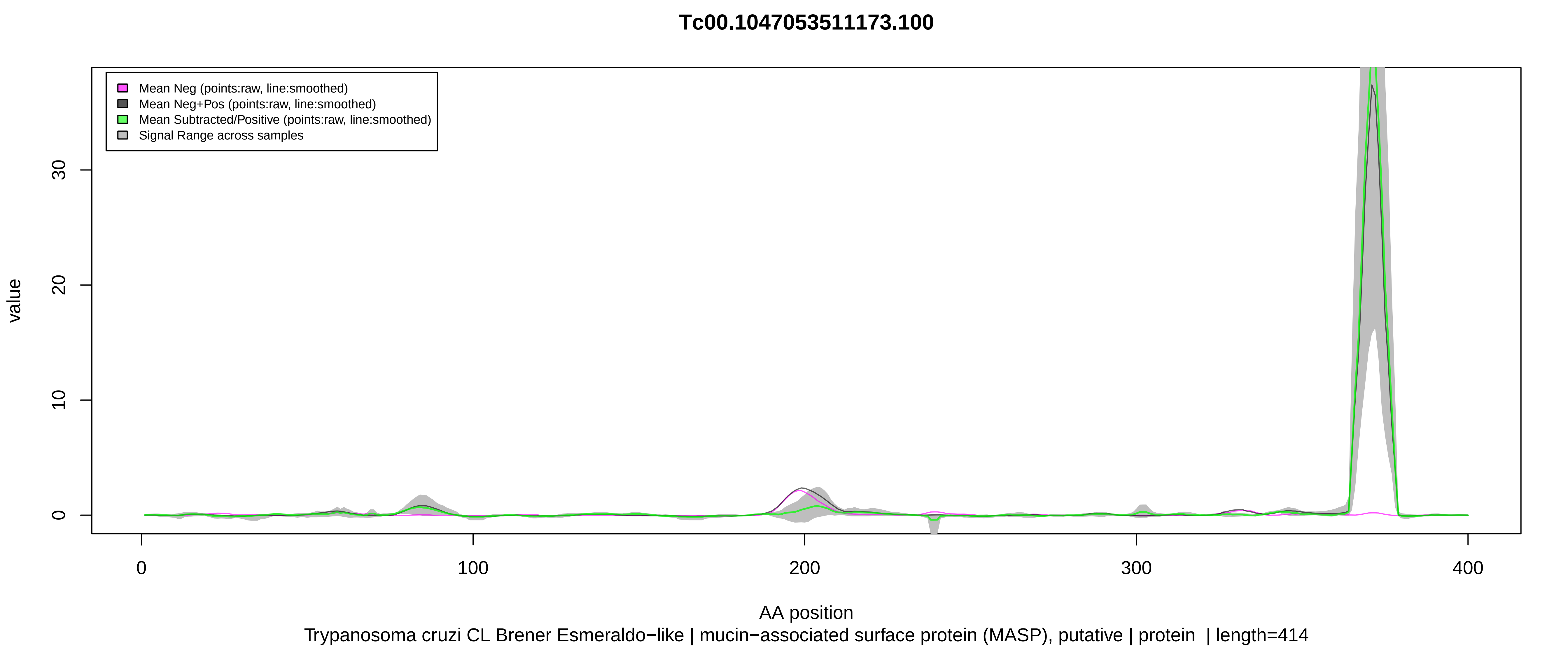

Tc00.1047053511173.100
Mean Neg (points:raw, line:smoothed)
Mean Neg+Pos (points:raw, line:smoothed)
Mean Subtracted/Positive (points:raw, line:smoothed)
Signal Range across samples
value
0
10
20
30
0
100
200
300
400
AA position
Trypanosoma cruzi CL Brener Esmeraldo−like | mucin−associated surface protein (MASP), putative | protein | length=414

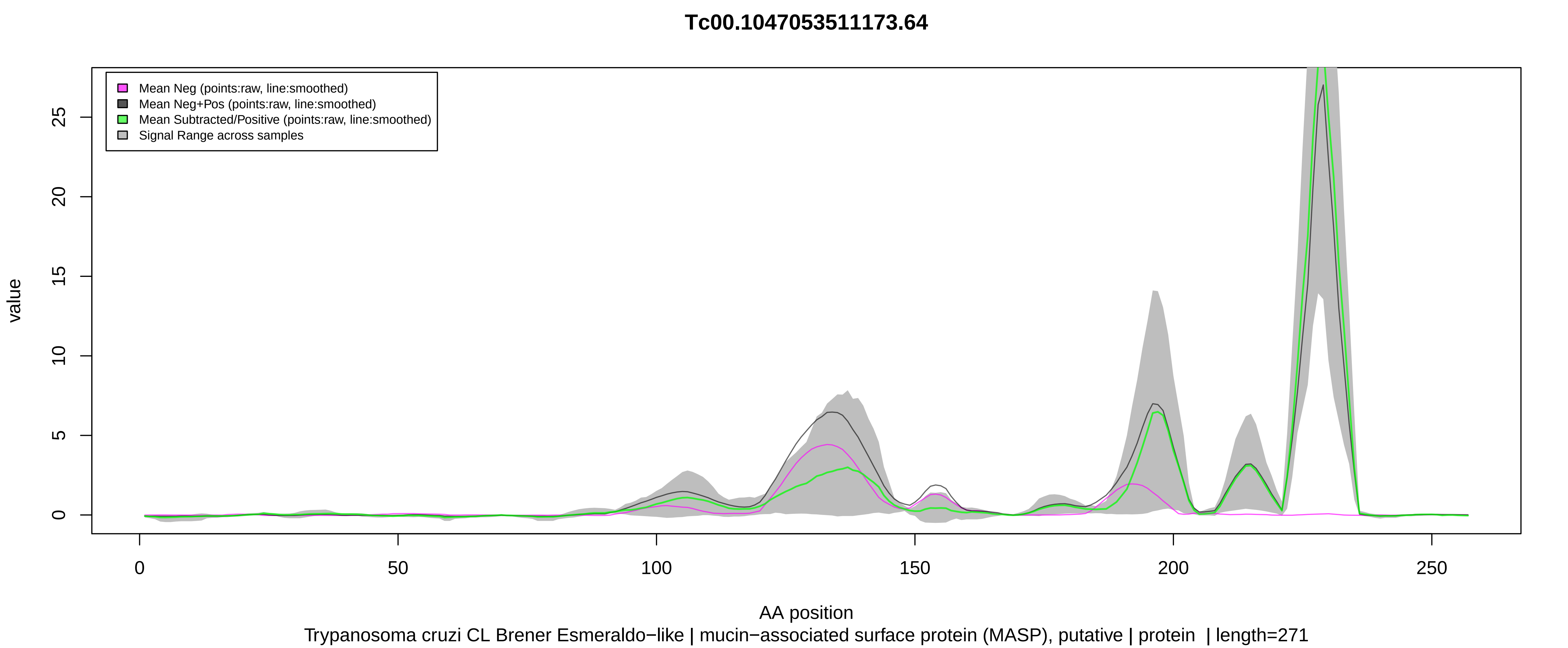
Tc00.1047053511173.64
Mean Neg (points:raw, line:smoothed)
Mean Neg+Pos (points:raw, line:smoothed)
Mean Subtracted/Positive (points:raw, line:smoothed)
Signal Range across samples
value
0
5
10
15
20
25
0
50
100
150
200
250
AA position
Trypanosoma cruzi CL Brener Esmeraldo−like | mucin−associated surface protein (MASP), putative | protein | length=271

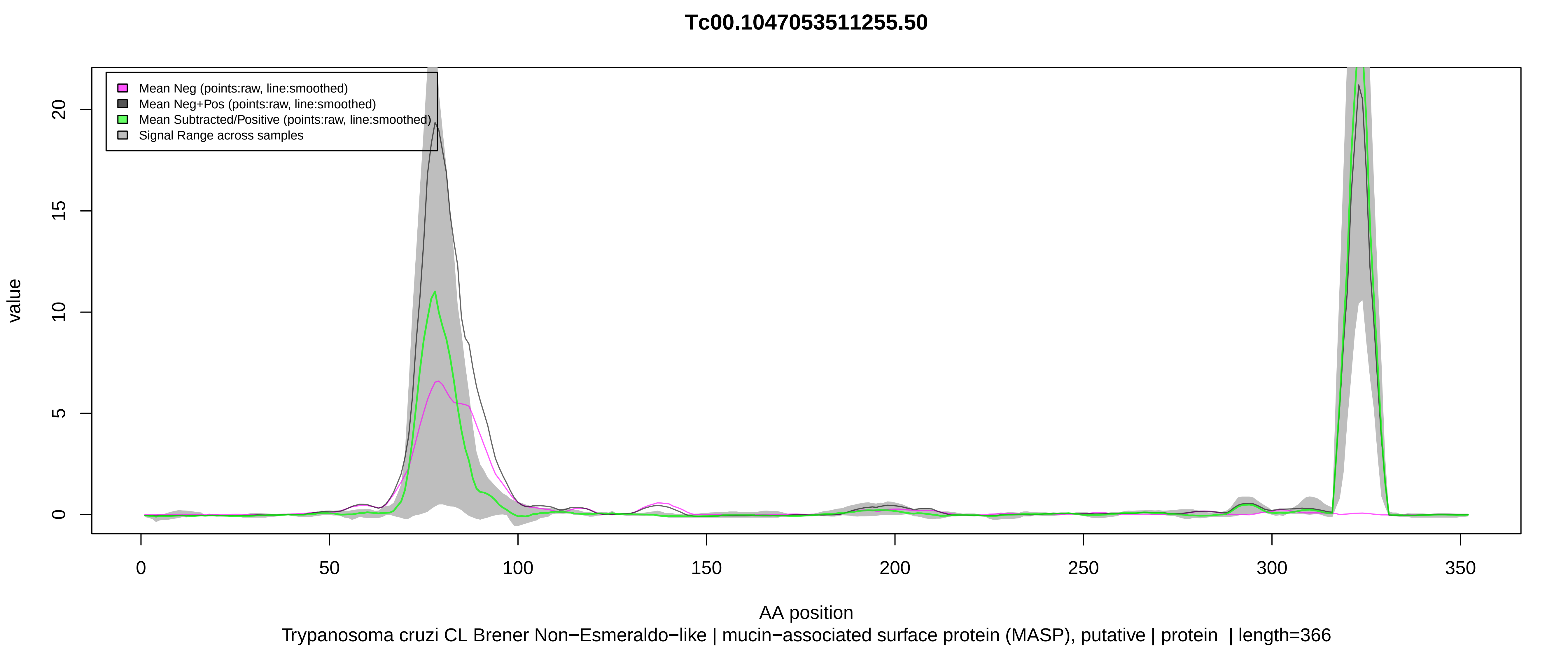
Tc00.1047053511255.50
Mean Neg (points:raw, line:smoothed)
Mean Neg+Pos (points:raw, line:smoothed)
Mean Subtracted/Positive (points:raw, line:smoothed)
Signal Range across samples
value
0
5
10
15
20
0
50
100
150
200
250
300
350
AA position
Trypanosoma cruzi CL Brener Non−Esmeraldo−like | mucin−associated surface protein (MASP), putative | protein | length=366

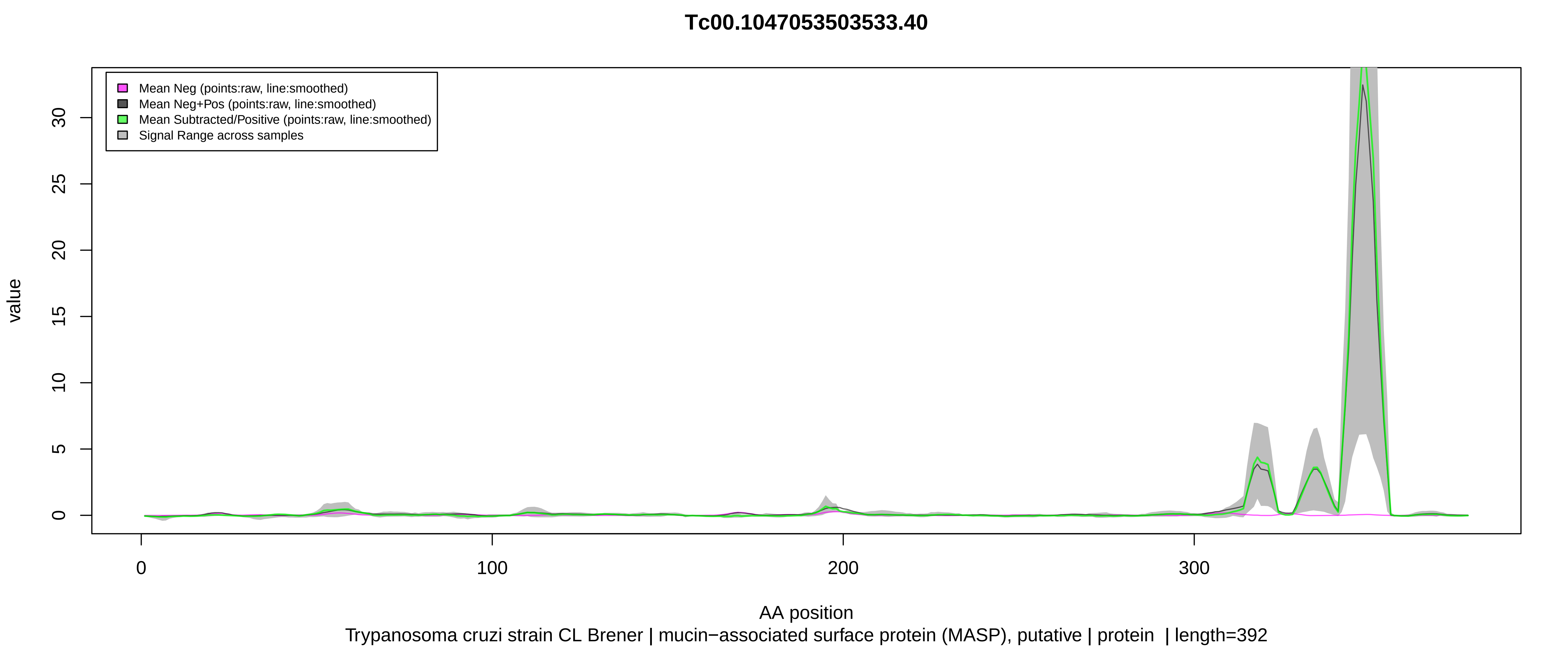

Tc00.1047053503533.40
Mean Neg (points:raw, line:smoothed)
Mean Neg+Pos (points:raw, line:smoothed)
Mean Subtracted/Positive (points:raw, line:smoothed)
Signal Range across samples
value
0
5
10
15
20
25
30
0
100
200
300
AA position
Trypanosoma cruzi strain CL Brener | mucin−associated surface protein (MASP), putative | protein | length=392

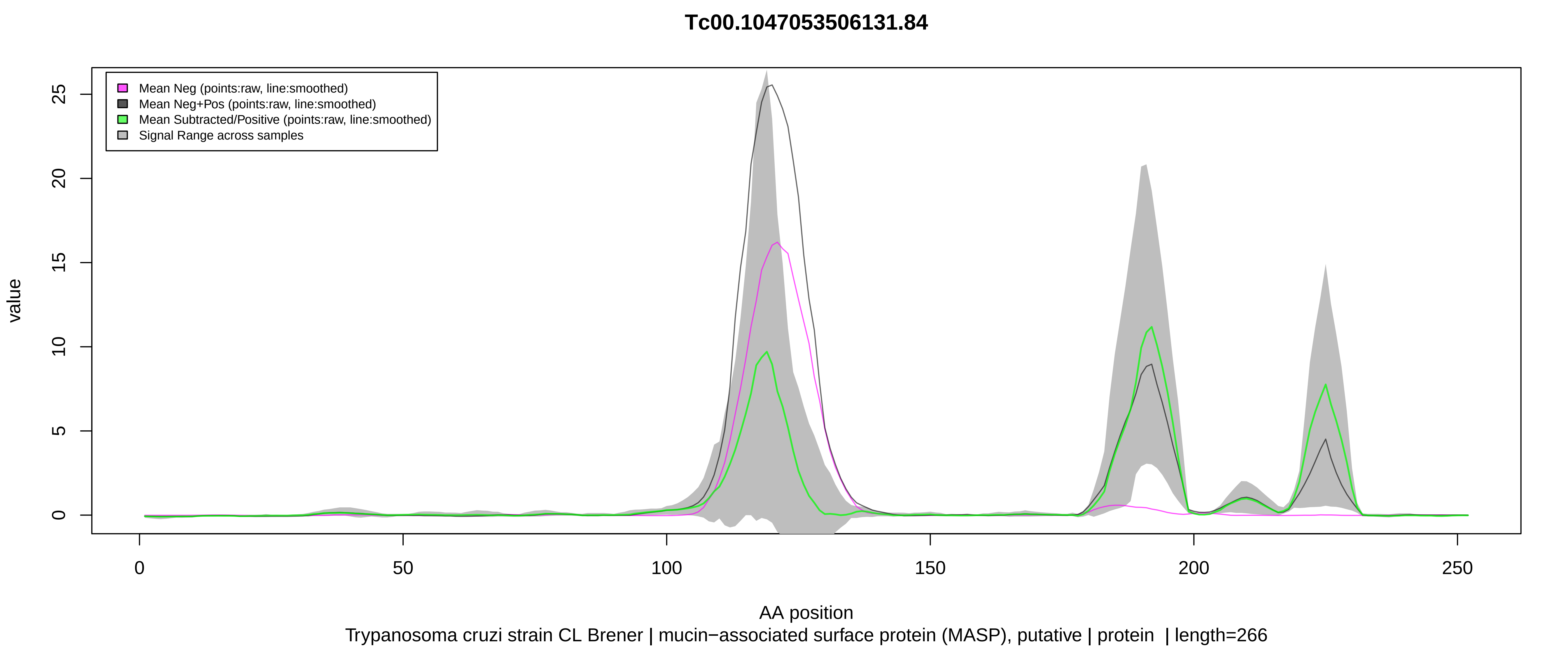
Tc00.1047053506131.84
Mean Neg (points:raw, line:smoothed)
Mean Neg+Pos (points:raw, line:smoothed)
Mean Subtracted/Positive (points:raw, line:smoothed)
Signal Range across samples
value
0
5
10
15
20
25
0
50
100
150
200
250
AA position
Trypanosoma cruzi strain CL Brener | mucin−associated surface protein (MASP), putative | protein | length=266

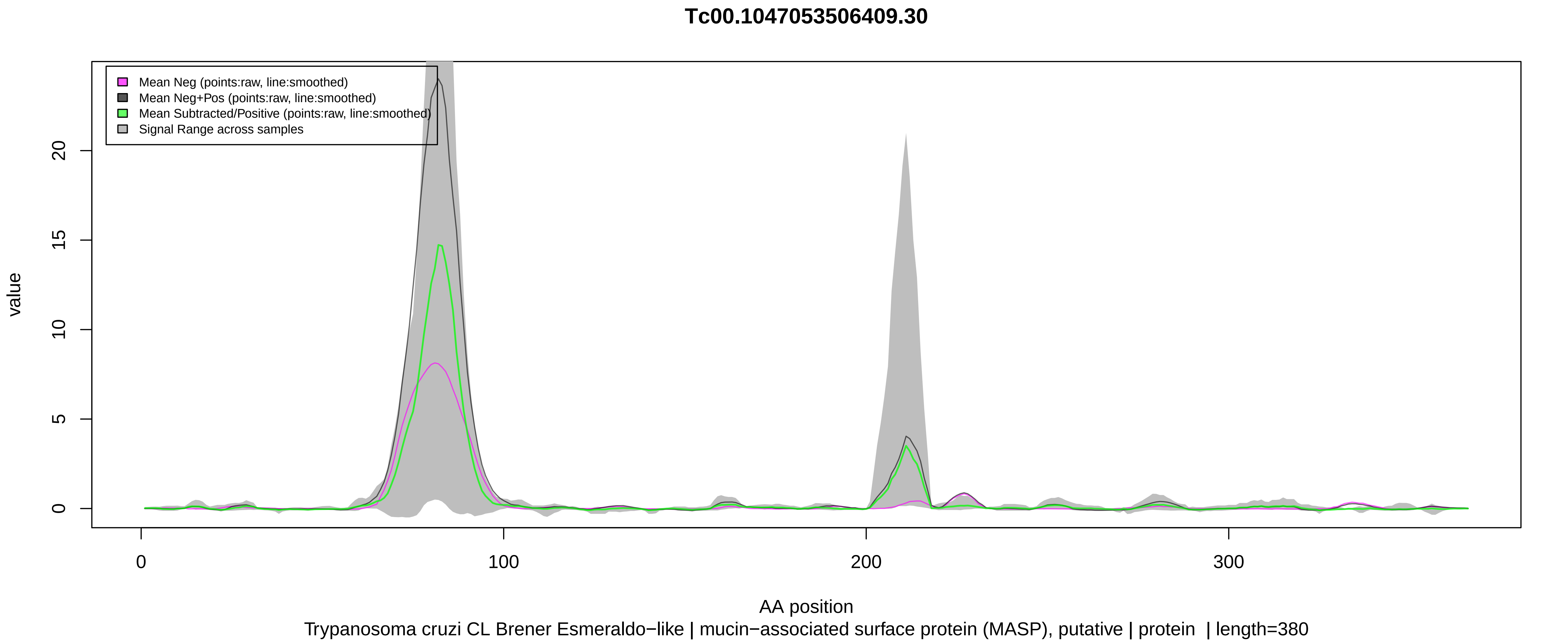

Tc00.1047053506409.30
Mean Neg (points:raw, line:smoothed)
Mean Neg+Pos (points:raw, line:smoothed)
Mean Subtracted/Positive (points:raw, line:smoothed)
Signal Range across samples
value
0
5
10
15
20
0
100
200
300
AA position
Trypanosoma cruzi CL Brener Esmeraldo−like | mucin−associated surface protein (MASP), putative | protein | length=380

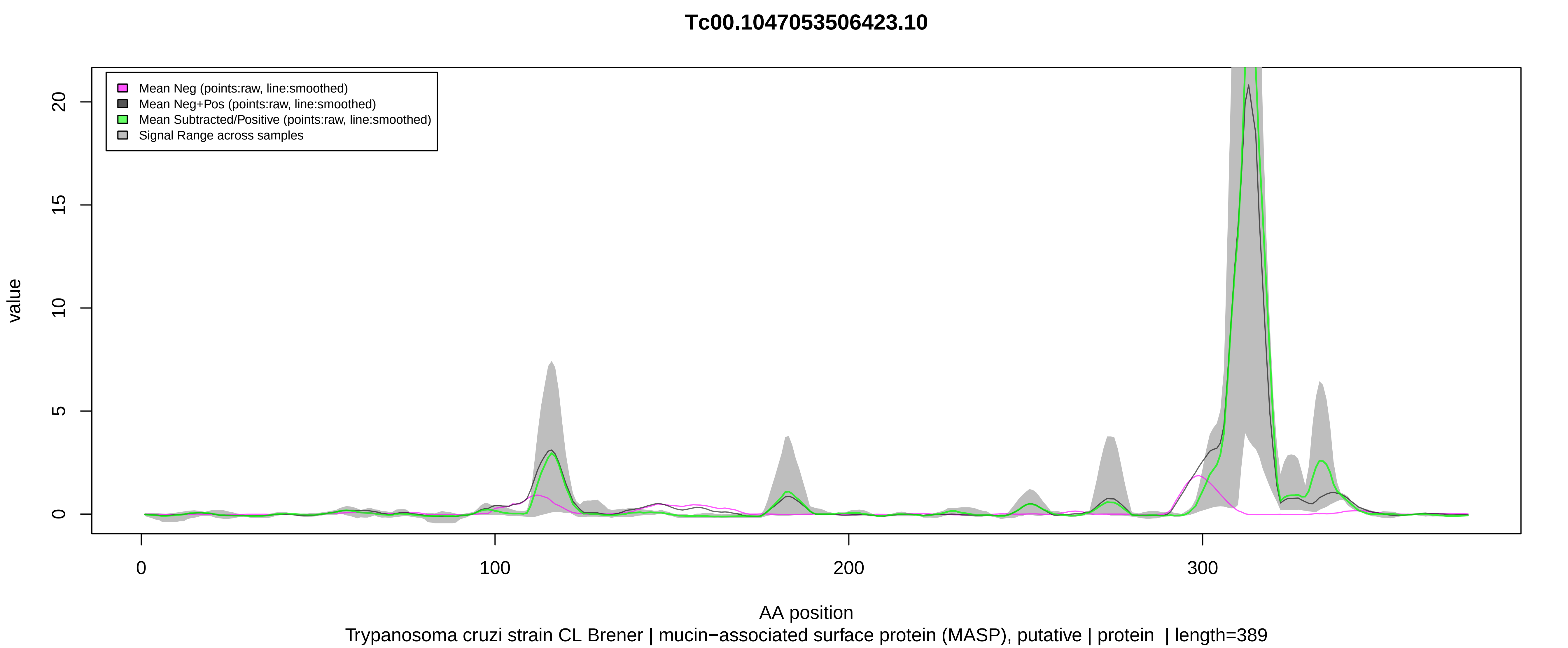
Tc00.1047053506423.10
Mean Neg (points:raw, line:smoothed)
Mean Neg+Pos (points:raw, line:smoothed)
Mean Subtracted/Positive (points:raw, line:smoothed)
Signal Range across samples
value
0
5
10
15
20
0
100
200
300
AA position
Trypanosoma cruzi strain CL Brener | mucin−associated surface protein (MASP), putative | protein | length=389

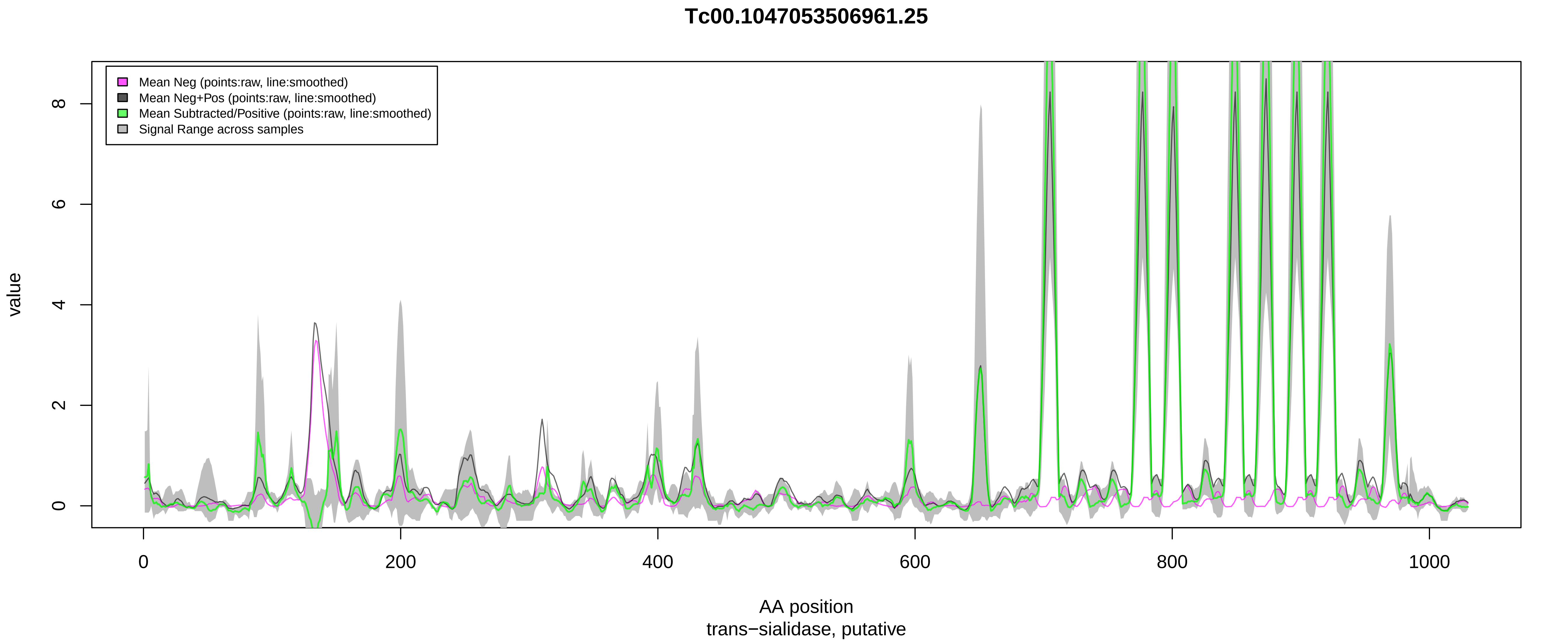

Tc00.1047053506961.25
Mean Neg (points:raw, line:smoothed)
Mean Neg+Pos (points:raw, line:smoothed)
Mean Subtracted/Positive (points:raw, line:smoothed)
Signal Range across samples
value
0
2
4
6
8
0
200
400
600
800
1000
AA position
trans−sialidase, putative

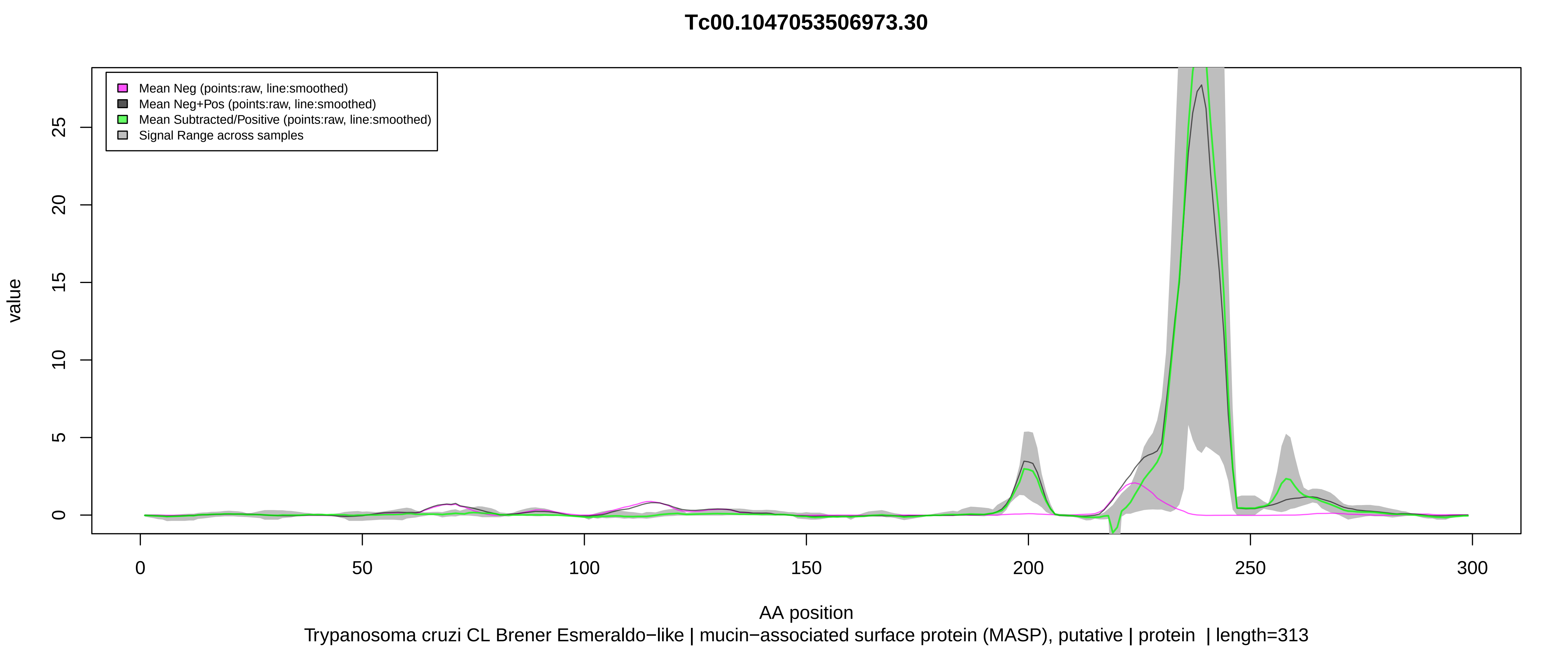

Tc00.1047053506973.30
Mean Neg (points:raw, line:smoothed)
Mean Neg+Pos (points:raw, line:smoothed)
Mean Subtracted/Positive (points:raw, line:smoothed)
Signal Range across samples
value
0
5
10
15
20
25
0
50
100
150
200
250
300
AA position
Trypanosoma cruzi CL Brener Esmeraldo−like | mucin−associated surface protein (MASP), putative | protein | length=313

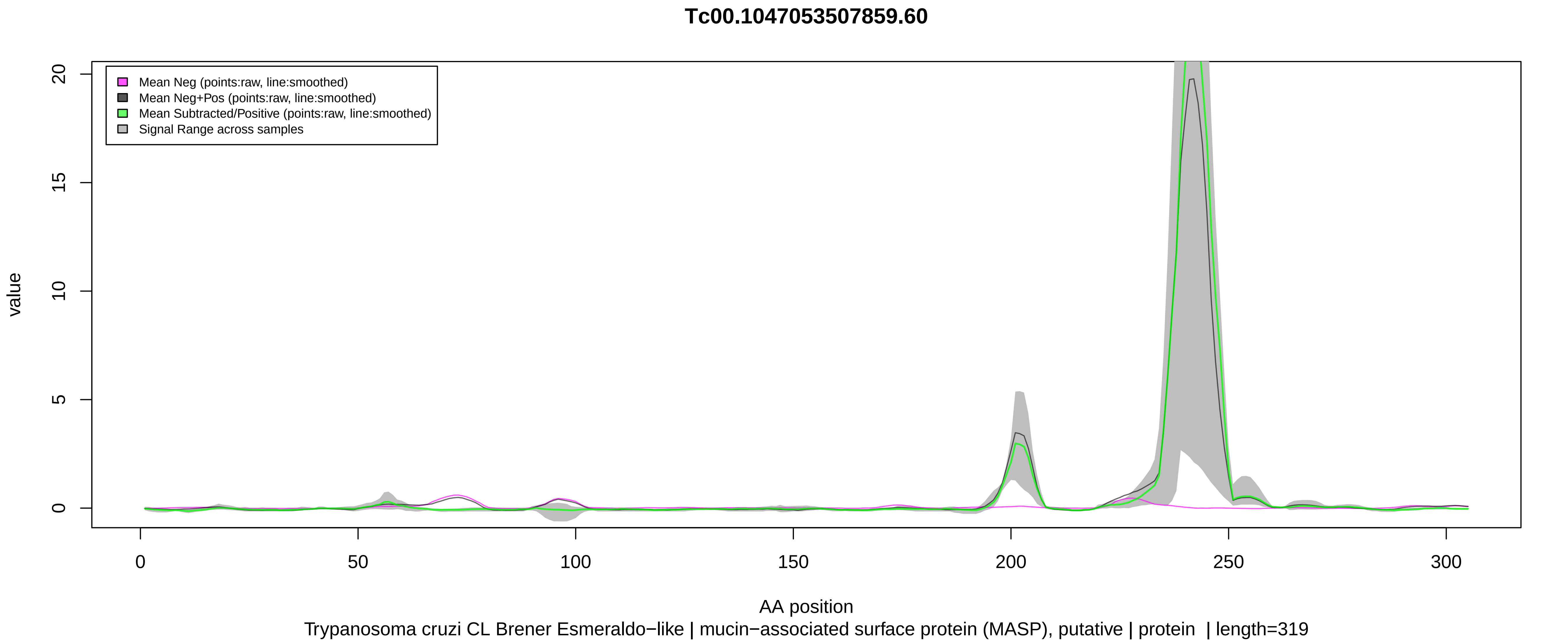
Tc00.1047053507859.60
Mean Neg (points:raw, line:smoothed)
Mean Neg+Pos (points:raw, line:smoothed)
Mean Subtracted/Positive (points:raw, line:smoothed)
Signal Range across samples
value
0
5
10
15
20
0
50
100
150
200
250
300
AA position
Trypanosoma cruzi CL Brener Esmeraldo−like | mucin−associated surface protein (MASP), putative | protein | length=319

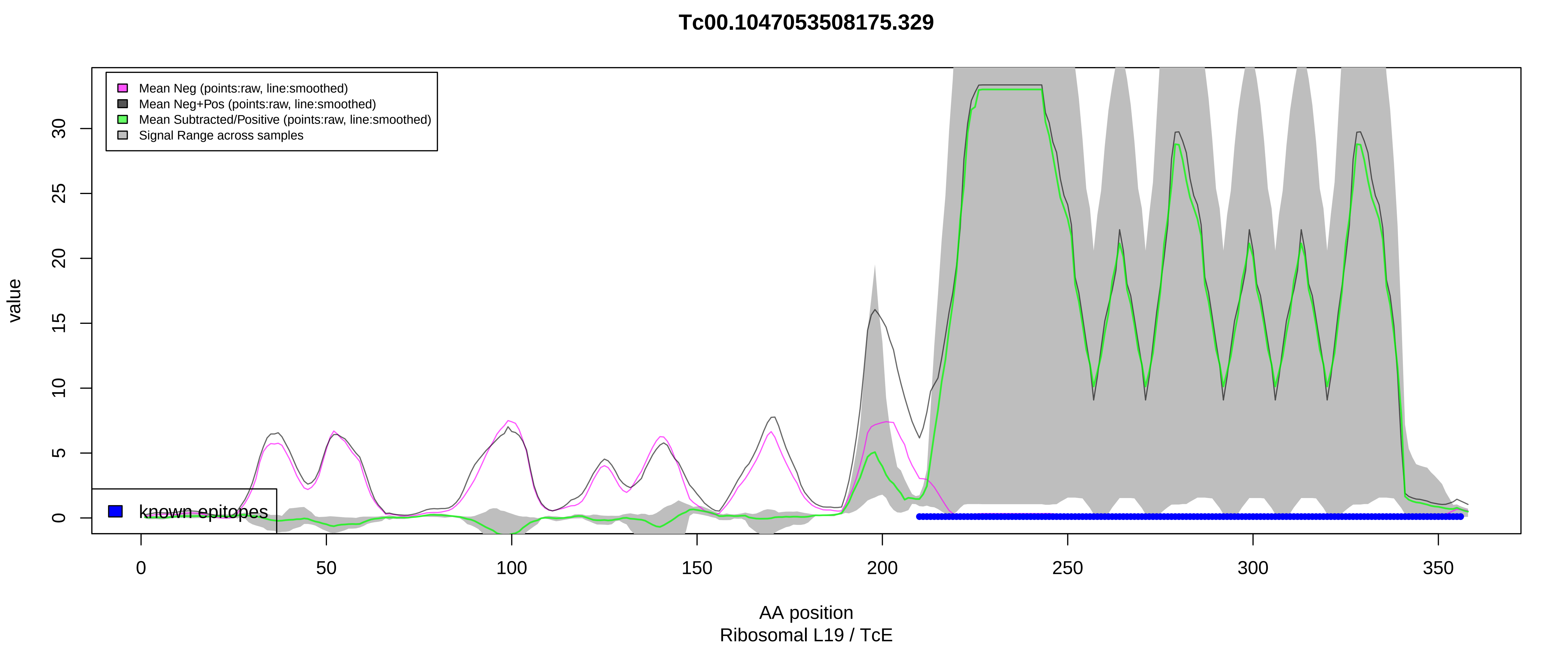

Tc00.1047053508175.329
Mean Neg (points:raw, line:smoothed)
Mean Neg+Pos (points:raw, line:smoothed)
Mean Subtracted/Positive (points:raw, line:smoothed)
Signal Range across samples
known epitopes
value
0
5
10
15
20
25
30
0
50
100
150
200
250
300
350
AA position
Ribosomal L19 / TcE

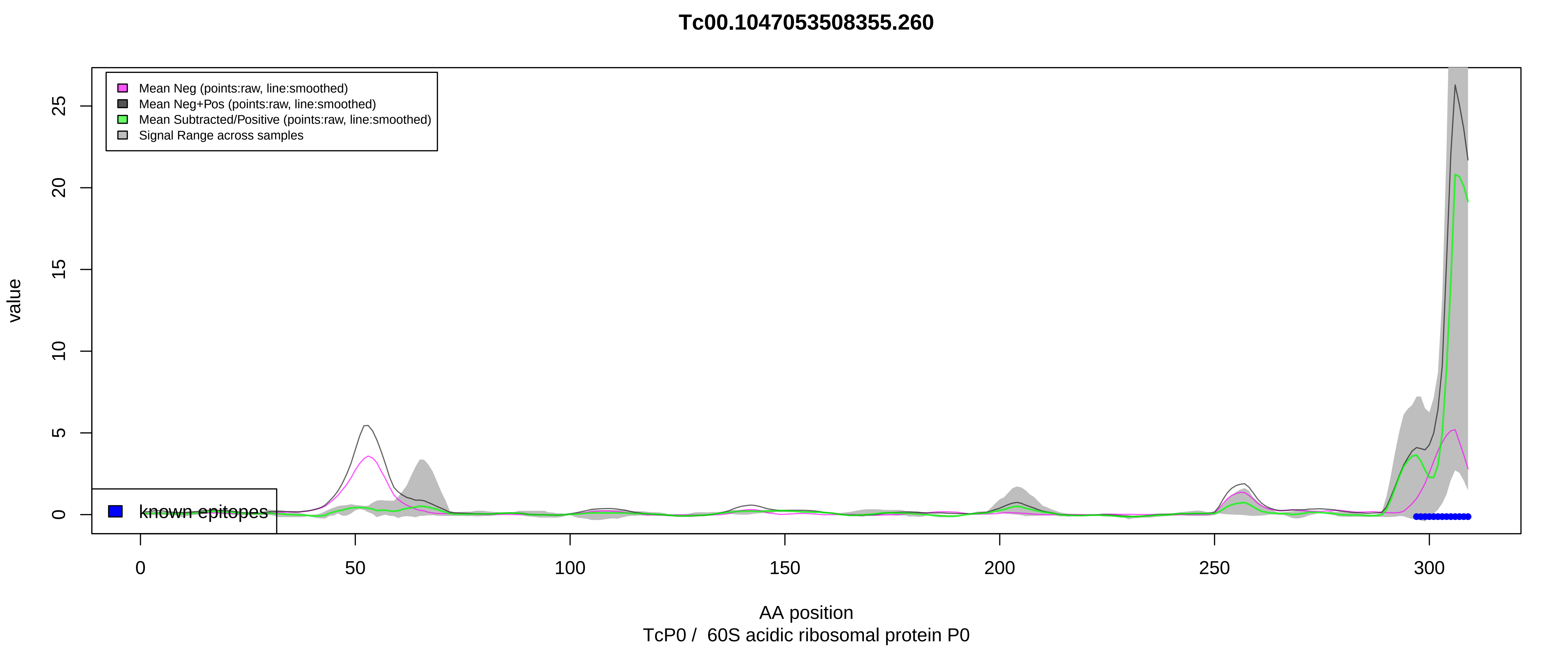

Tc00.1047053508355.260
Mean Neg (points:raw, line:smoothed)
Mean Neg+Pos (points:raw, line:smoothed)
Mean Subtracted/Positive (points:raw, line:smoothed)
Signal Range across samples
known epitopes
value
0
5
10
15
20
25
0
50
100
150
200
250
300
AA position
TcP0 / 60S acidic ribosomal protein P0

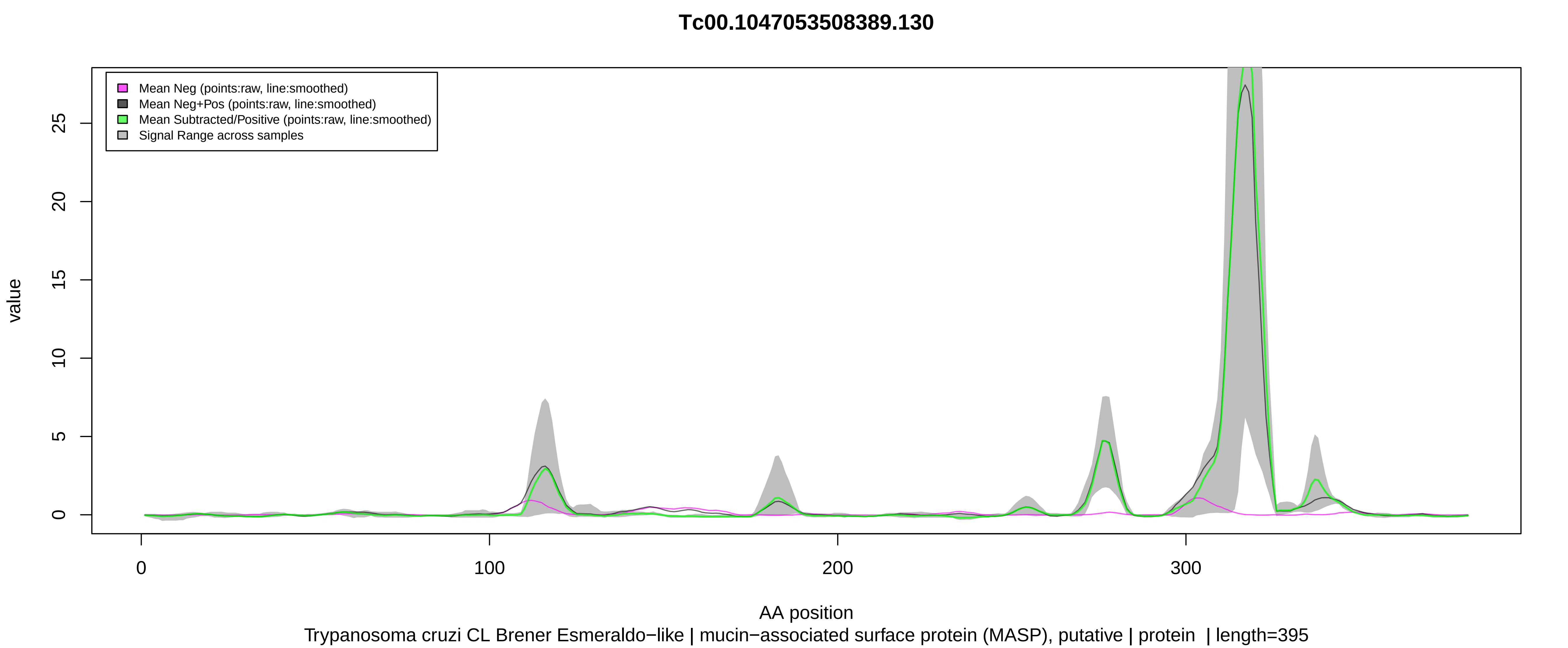

Tc00.1047053508389.130
Mean Neg (points:raw, line:smoothed)
Mean Neg+Pos (points:raw, line:smoothed)
Mean Subtracted/Positive (points:raw, line:smoothed)
Signal Range across samples
value
0
5
10
15
20
25
0
100
200
300
AA position
Trypanosoma cruzi CL Brener Esmeraldo−like | mucin−associated surface protein (MASP), putative | protein | length=395

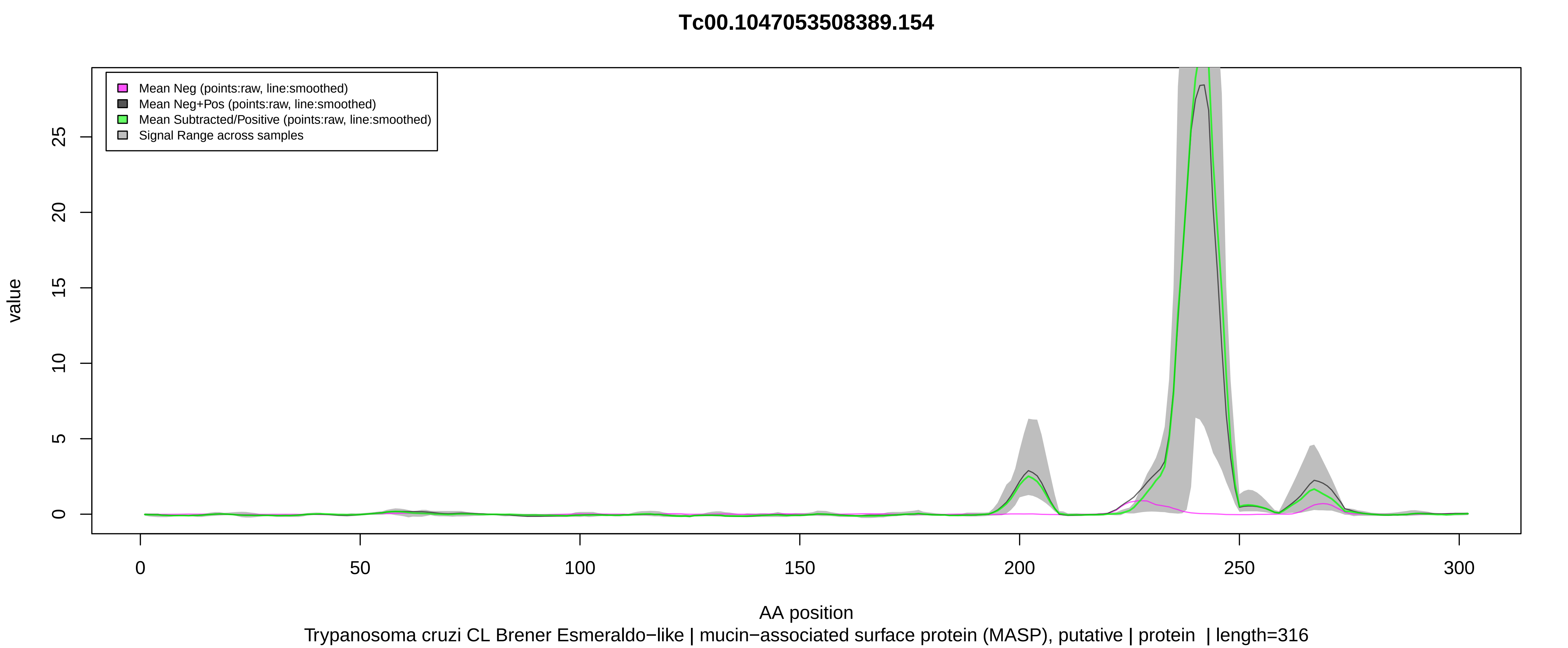

Tc00.1047053508389.154
Mean Neg (points:raw, line:smoothed)
Mean Neg+Pos (points:raw, line:smoothed)
Mean Subtracted/Positive (points:raw, line:smoothed)
Signal Range across samples
value
0
5
10
15
20
25
0
50
100
150
200
250
300
AA position
Trypanosoma cruzi CL Brener Esmeraldo−like | mucin−associated surface protein (MASP), putative | protein | length=316

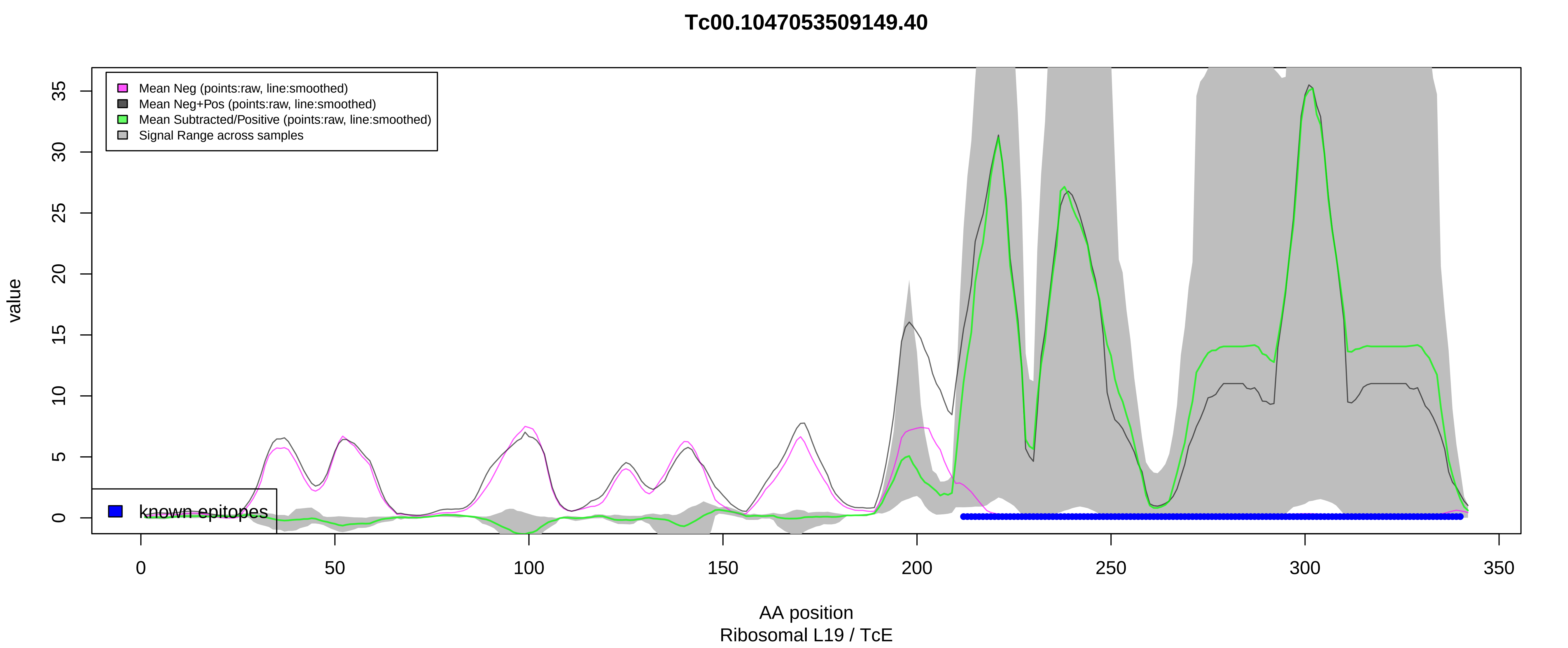

Tc00.1047053509149.40
Mean Neg (points:raw, line:smoothed)
Mean Neg+Pos (points:raw, line:smoothed)
Mean Subtracted/Positive (points:raw, line:smoothed)
Signal Range across samples
known epitopes
value
0
5
10
15
20
25
30
35
0
50
100
150
200
250
300
350
AA position
Ribosomal L19 / TcE

Tc00.1047053509151.140

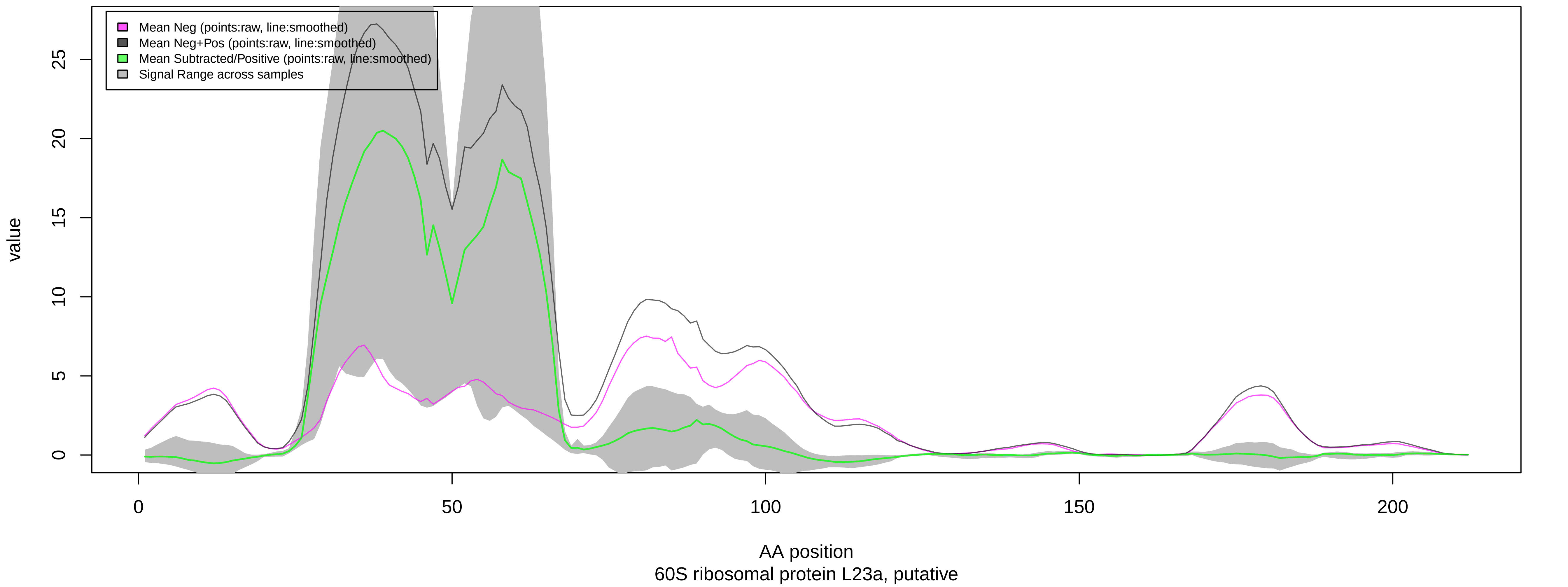

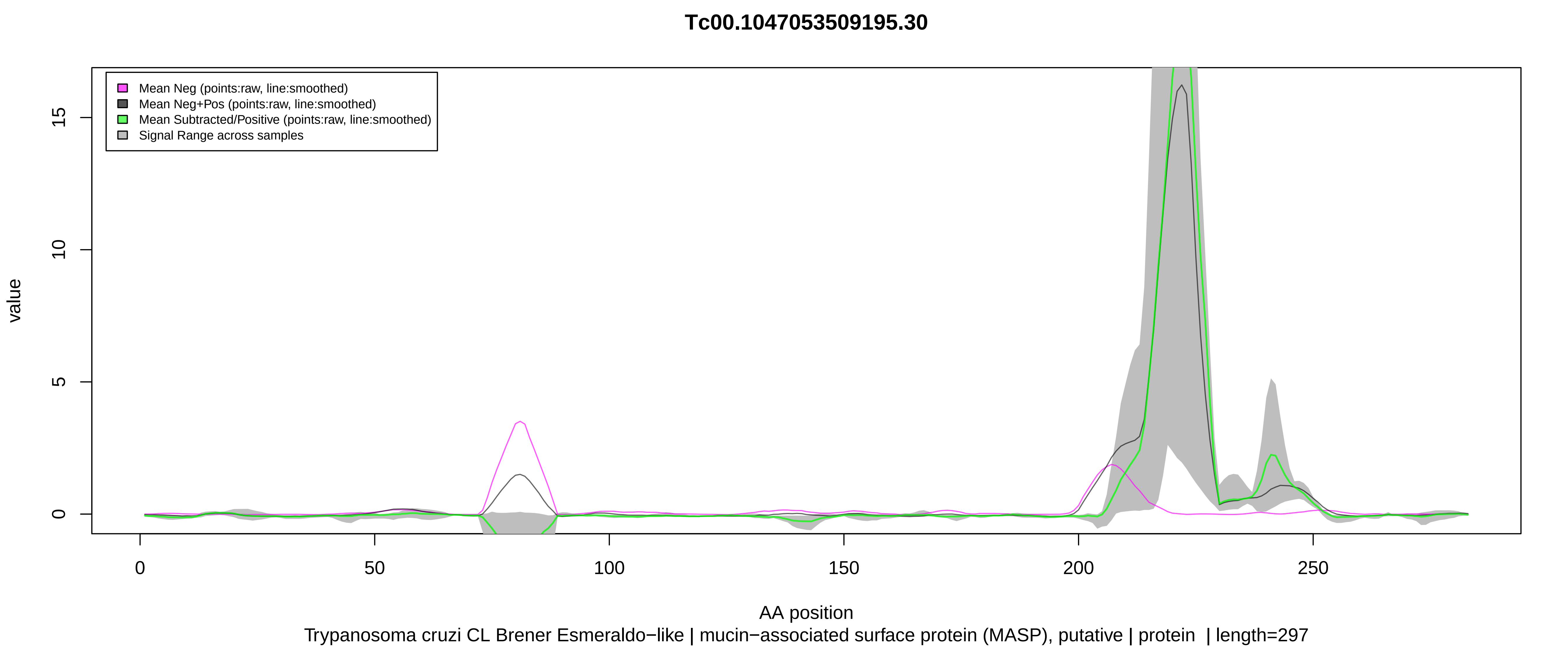
Tc00.1047053509195.30
Mean Neg (points:raw, line:smoothed)
Mean Neg+Pos (points:raw, line:smoothed)
Mean Subtracted/Positive (points:raw, line:smoothed)
Signal Range across samples
value
0
5
10
15
0
50
100
150
200
250
AA position
Trypanosoma cruzi CL Brener Esmeraldo−like | mucin−associated surface protein (MASP), putative | protein | length=297

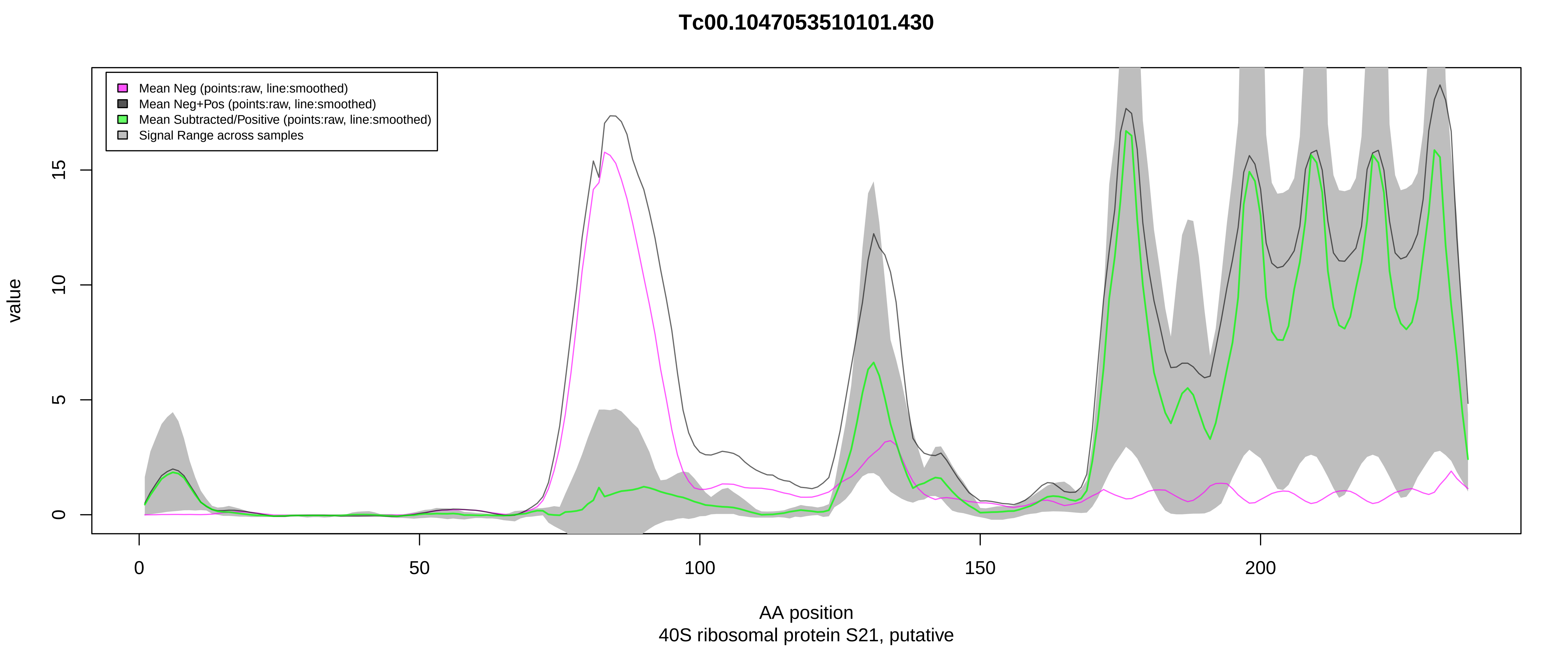

Tc00.1047053510101.430
Mean Neg (points:raw, line:smoothed)
Mean Neg+Pos (points:raw, line:smoothed)
Mean Subtracted/Positive (points:raw, line:smoothed)
Signal Range across samples
value
0
5
10
15
0
50
100
150
200
AA position
40S ribosomal protein S21, putative

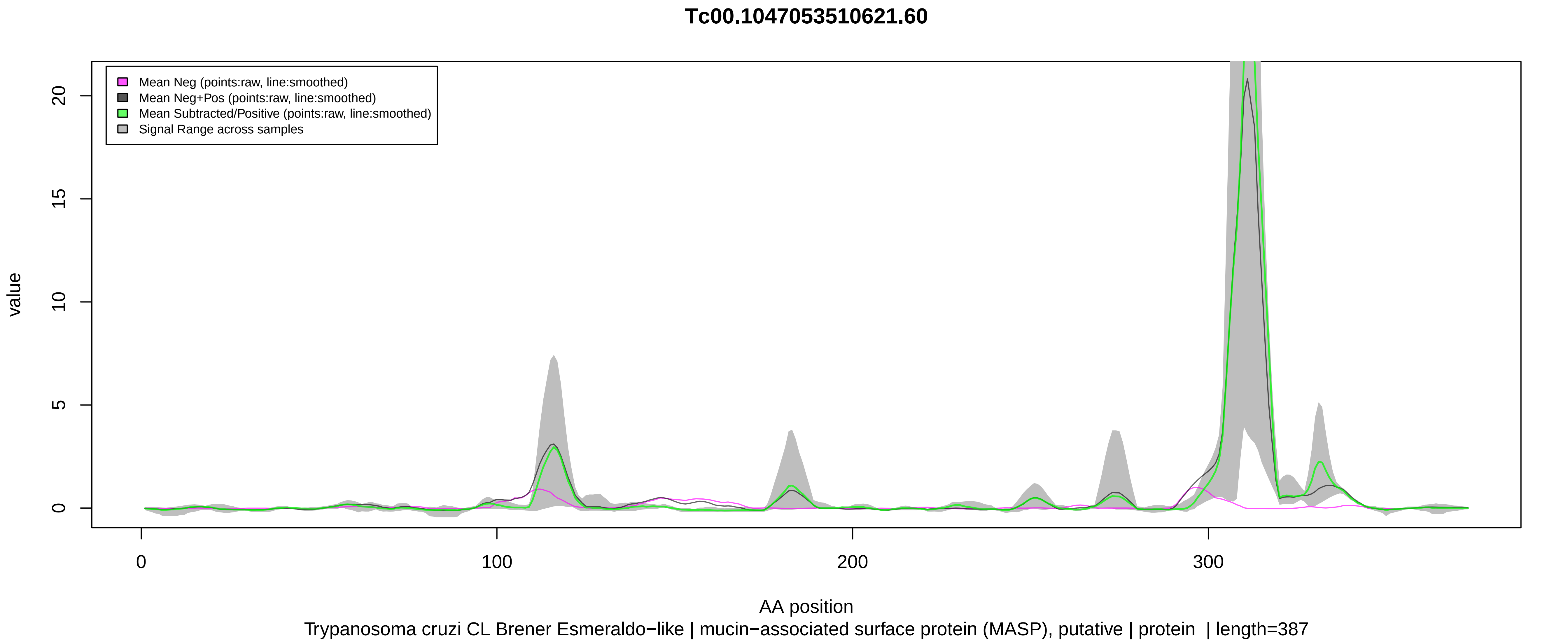
Tc00.1047053510621.60
Mean Neg (points:raw, line:smoothed)
Mean Neg+Pos (points:raw, line:smoothed)
Mean Subtracted/Positive (points:raw, line:smoothed)
Signal Range across samples
value
0
5
10
15
20
0
100
200
300
AA position
Trypanosoma cruzi CL Brener Esmeraldo−like | mucin−associated surface protein (MASP), putative | protein | length=387

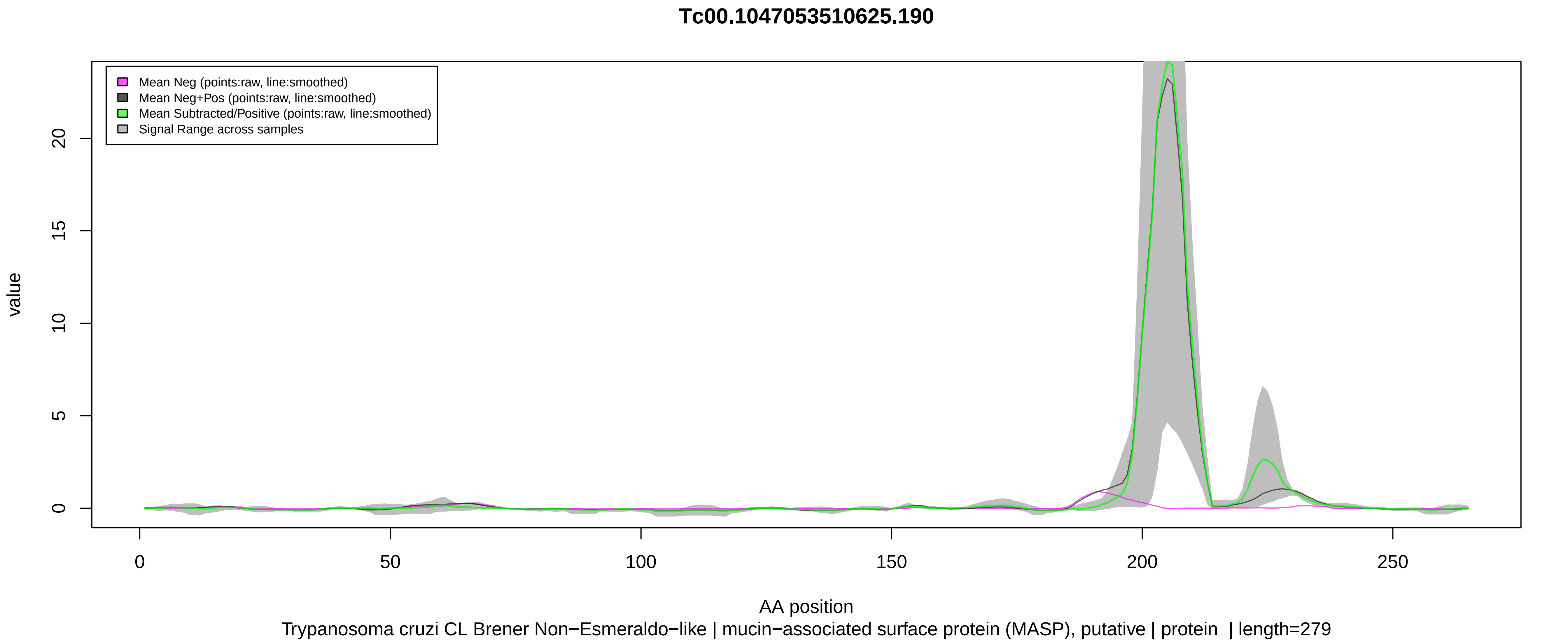
Tc00.1047053510625.190
Mean Neg (points:raw, line:smoothed)
Mean Neg+Pos (points:raw, line:smoothed)
Mean Subtracted/Positive (points:raw, line:smoothed)
Signal Range across samples
value
0
5
10
15
20
0
50
100
150
200
250
AA position
Trypanosoma cruzi CL Brener Non−Esmeraldo−like | mucin−associated surface protein (MASP), putative | protein | length=279

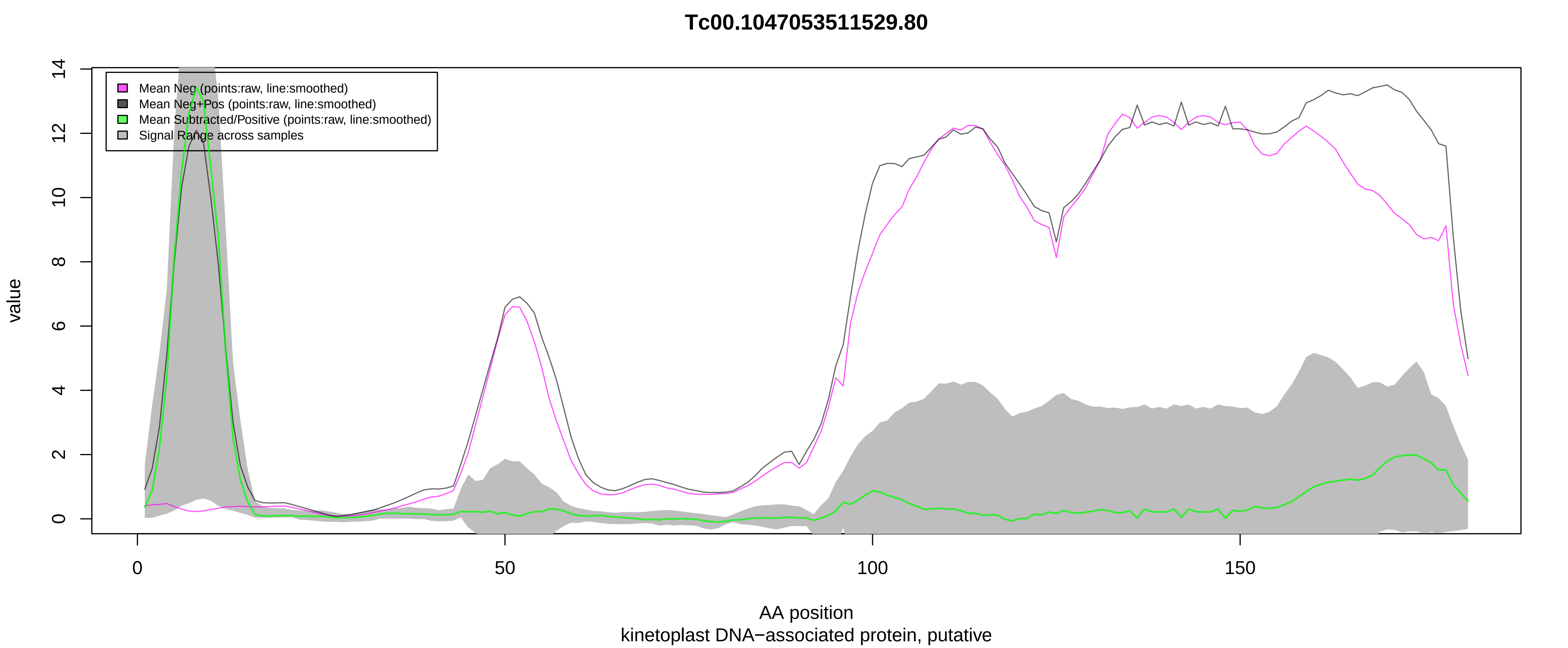

Tc00.1047053511529.80
Mean Neg (points:raw, line:smoothed)
Mean Neg+Pos (points:raw, line:smoothed)
Mean Subtracted/Positive (points:raw, line:smoothed)
Signal Range across samples
value
0
2
4
6
8
10
12
14
0
50
100
150
AA position
kinetoplast DNA−associated protein, putative

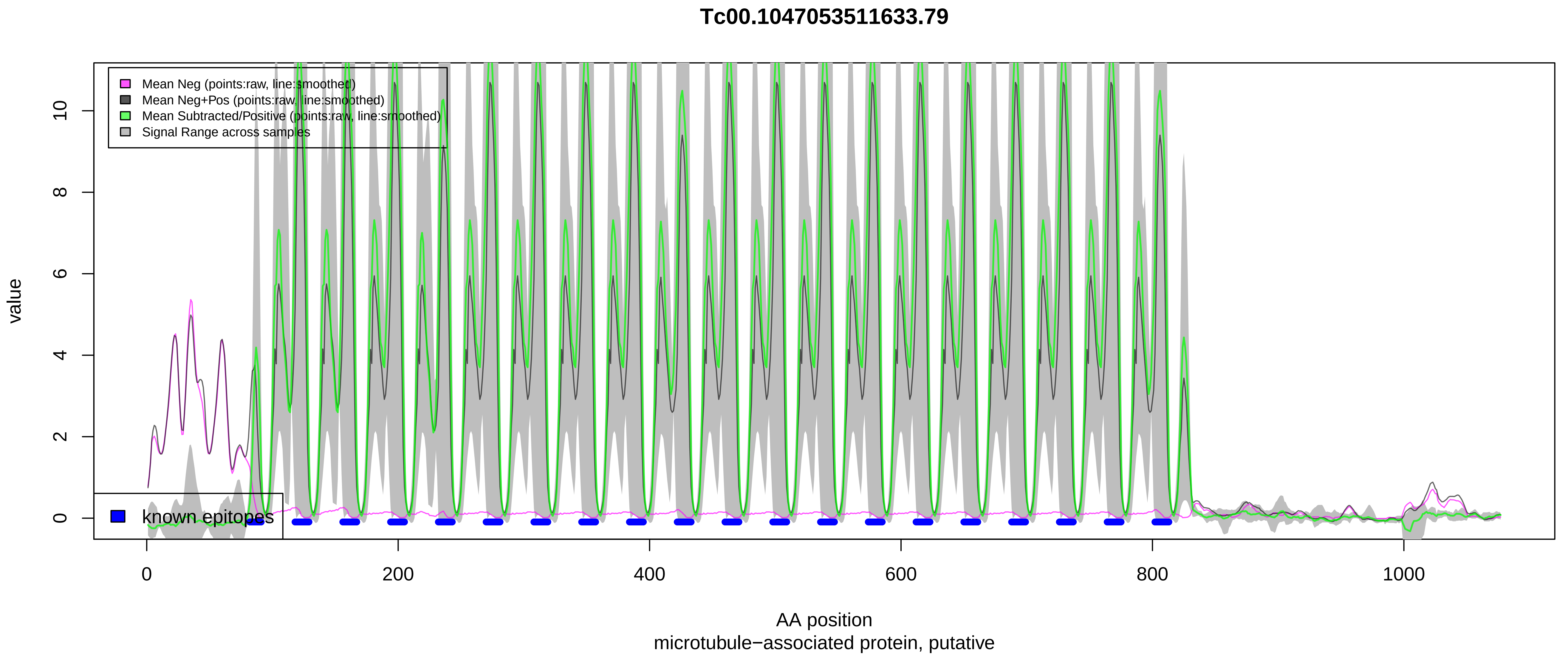
Tc00.1047053511633.79
Mean Neg (points:raw, line:smoothed)
Mean Neg+Pos (points:raw, line:smoothed)
Mean Subtracted/Positive (points:raw, line:smoothed)
Signal Range across samples
known epitopes
value
0
2
4
6
8
10
0
200
400
600
800
1000
AA position
microtubule−associated protein, putative

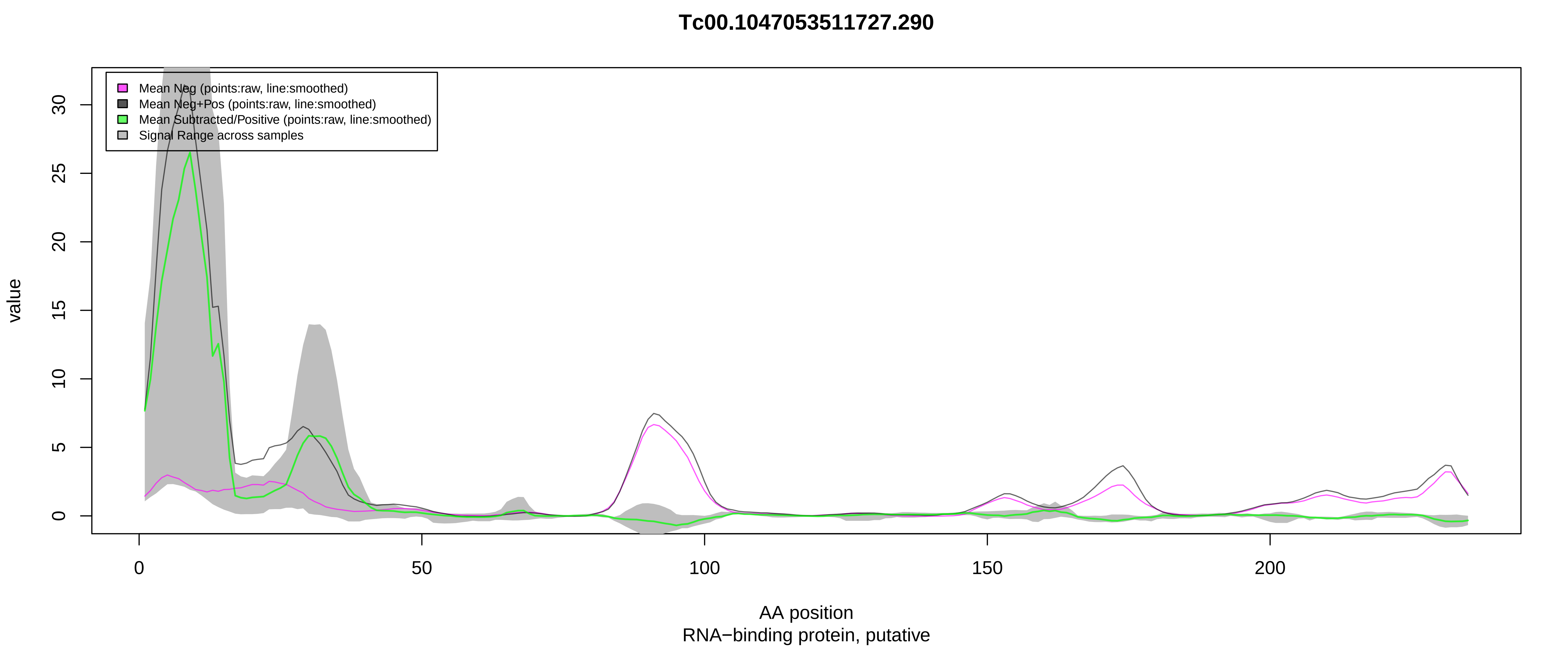
Tc00.1047053511727.290
Mean Neg (points:raw, line:smoothed)
Mean Neg+Pos (points:raw, line:smoothed)
Mean Subtracted/Positive (points:raw, line:smoothed)
Signal Range across samples
value
0
5
10
15
20
25
30
0
50
100
150
200
AA position
RNA−binding protein, putative

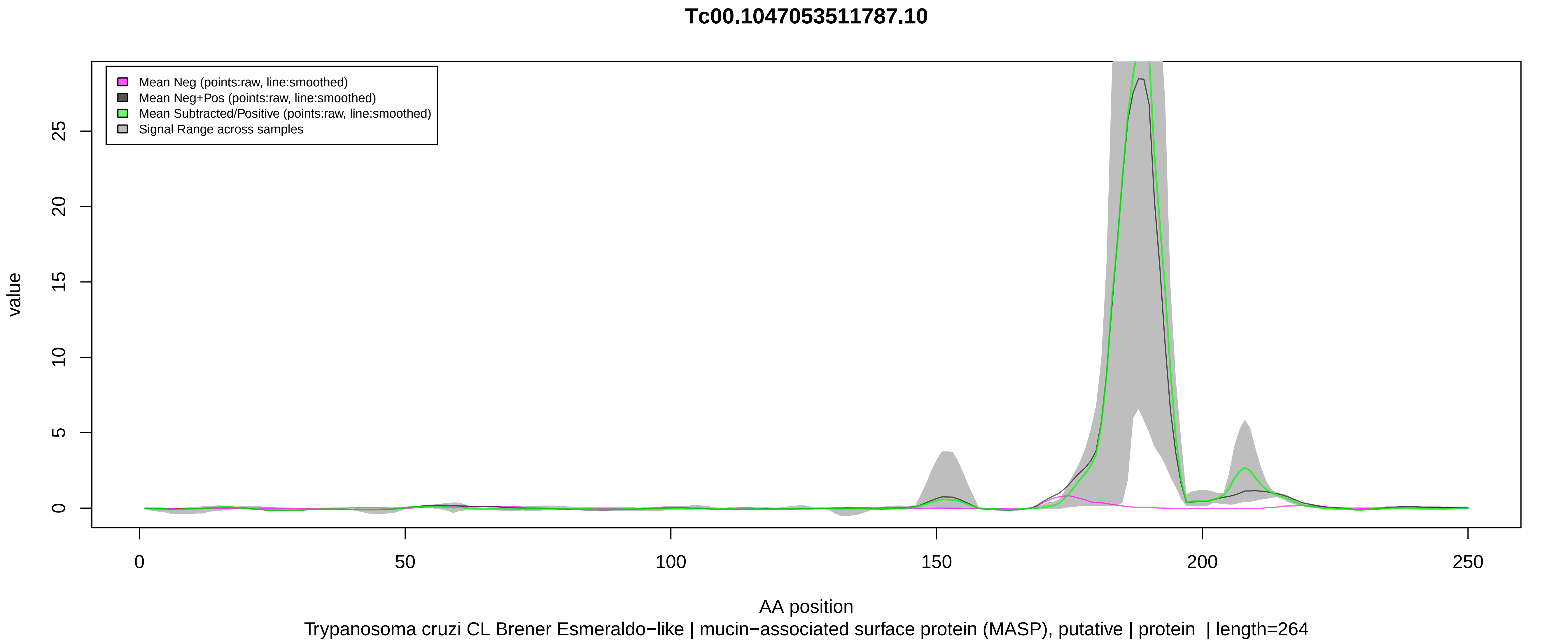

Tc00.1047053511787.10
Mean Neg (points:raw, line:smoothed)
Mean Neg+Pos (points:raw, line:smoothed)
Mean Subtracted/Positive (points:raw, line:smoothed)
Signal Range across samples
value
0
5
10
15
20
25
0
50
100
150
200
250
AA position
Trypanosoma cruzi CL Brener Esmeraldo−like | mucin−associated surface protein (MASP), putative | protein | length=264

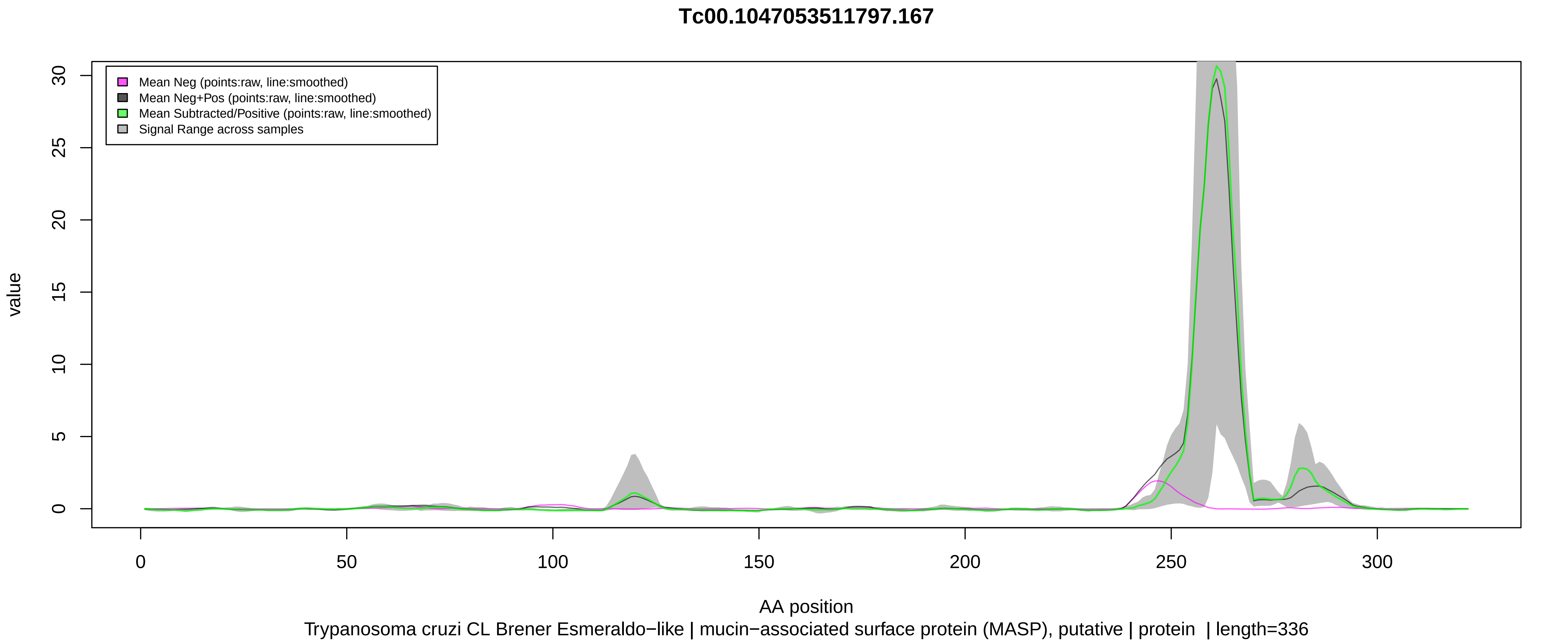

Tc00.1047053511797.167
Mean Neg (points:raw, line:smoothed)
Mean Neg+Pos (points:raw, line:smoothed)
Mean Subtracted/Positive (points:raw, line:smoothed)
Signal Range across samples
value
0
5
10
15
20
25
30
0
50
100
150
200
250
300
AA position
Trypanosoma cruzi CL Brener Esmeraldo−like | mucin−associated surface protein (MASP), putative | protein | length=336

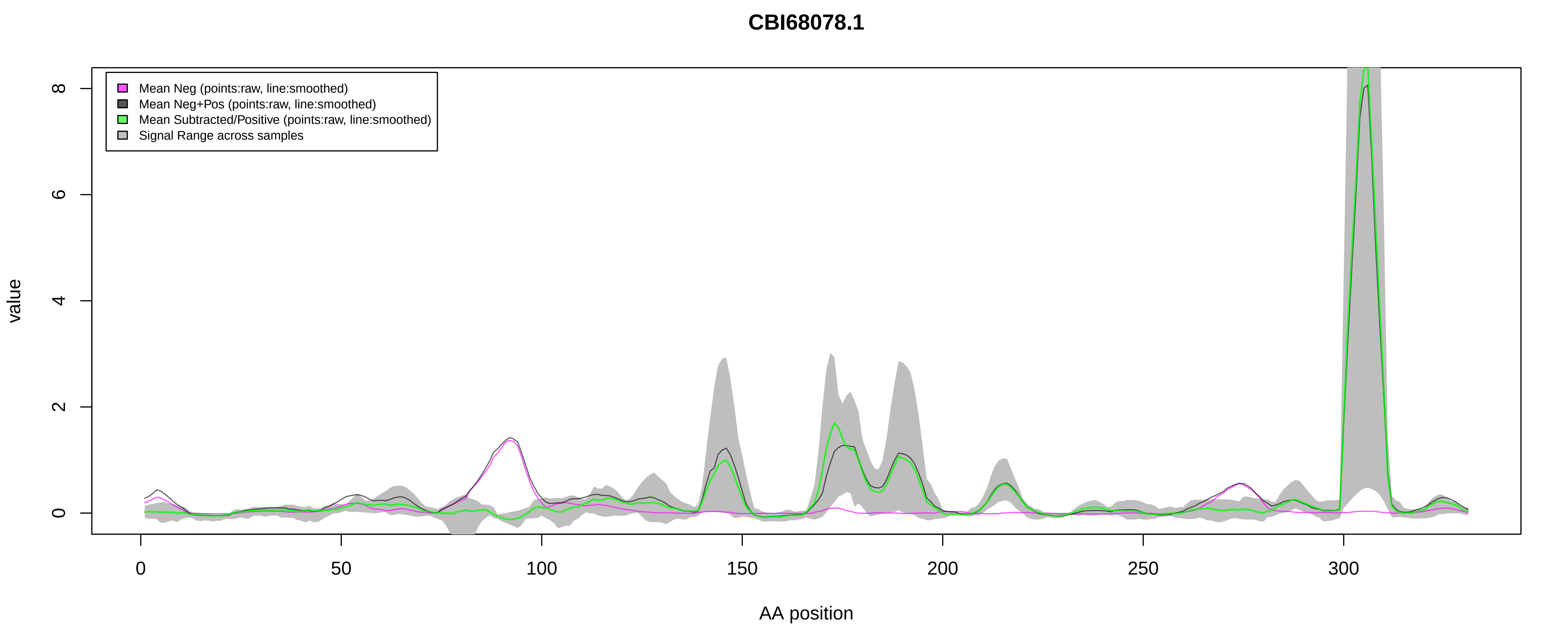

CBI68078.1
Mean Neg (points:raw, line:smoothed)
Mean Neg+Pos (points:raw, line:smoothed)
Mean Subtracted/Positive (points:raw, line:smoothed)
Signal Range across samples
value
0
2
4
6
8
0
50
100
150
200
250
300
AA position

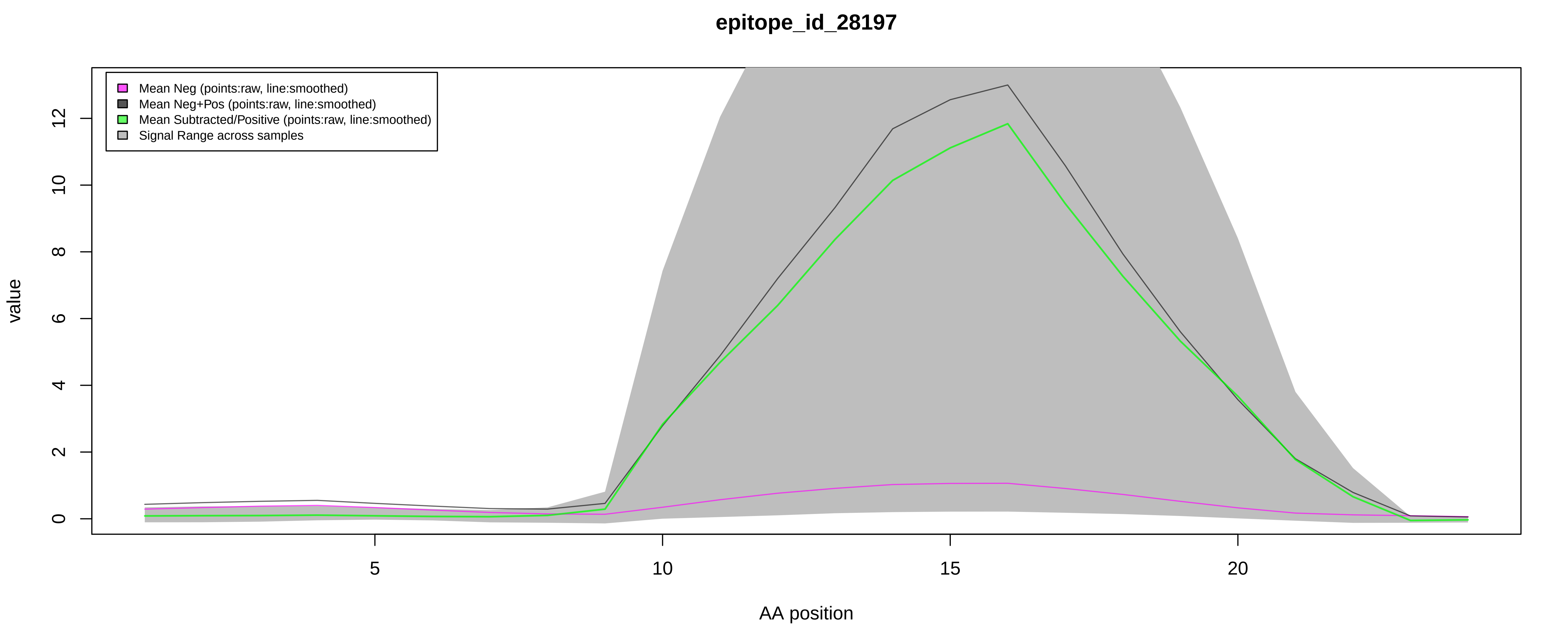

epitope_id_28197
Mean Neg (points:raw, line:smoothed)
Mean Neg+Pos (points:raw, line:smoothed)
Mean Subtracted/Positive (points:raw, line:smoothed)
Signal Range across samples
value
0
2
4
6
8
10
12
AA position
5
10
15
20

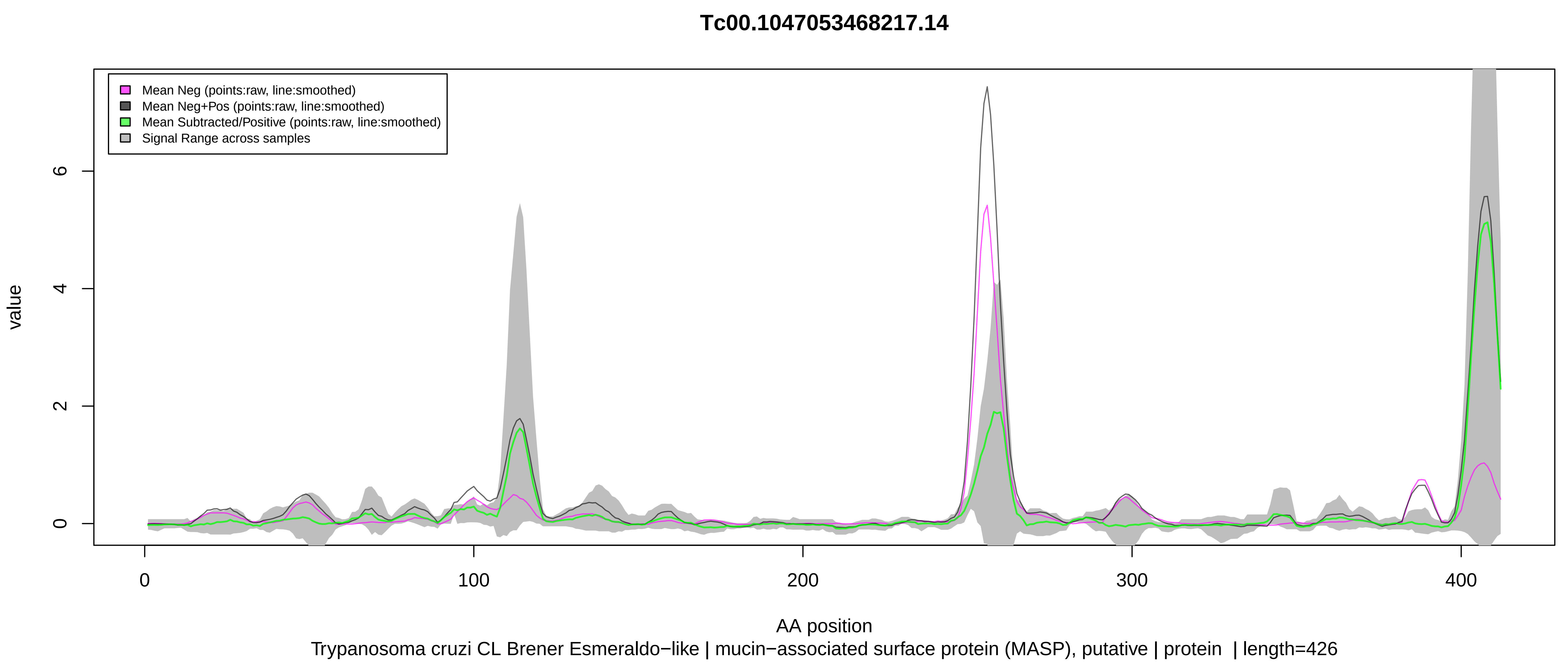
Tc00.1047053468217.14
Mean Neg (points:raw, line:smoothed)
Mean Neg+Pos (points:raw, line:smoothed)
Mean Subtracted/Positive (points:raw, line:smoothed)
Signal Range across samples
value
0
2
4
6
0
100
200
300
400
AA position
Trypanosoma cruzi CL Brener Esmeraldo−like | mucin−associated surface protein (MASP), putative | protein | length=426

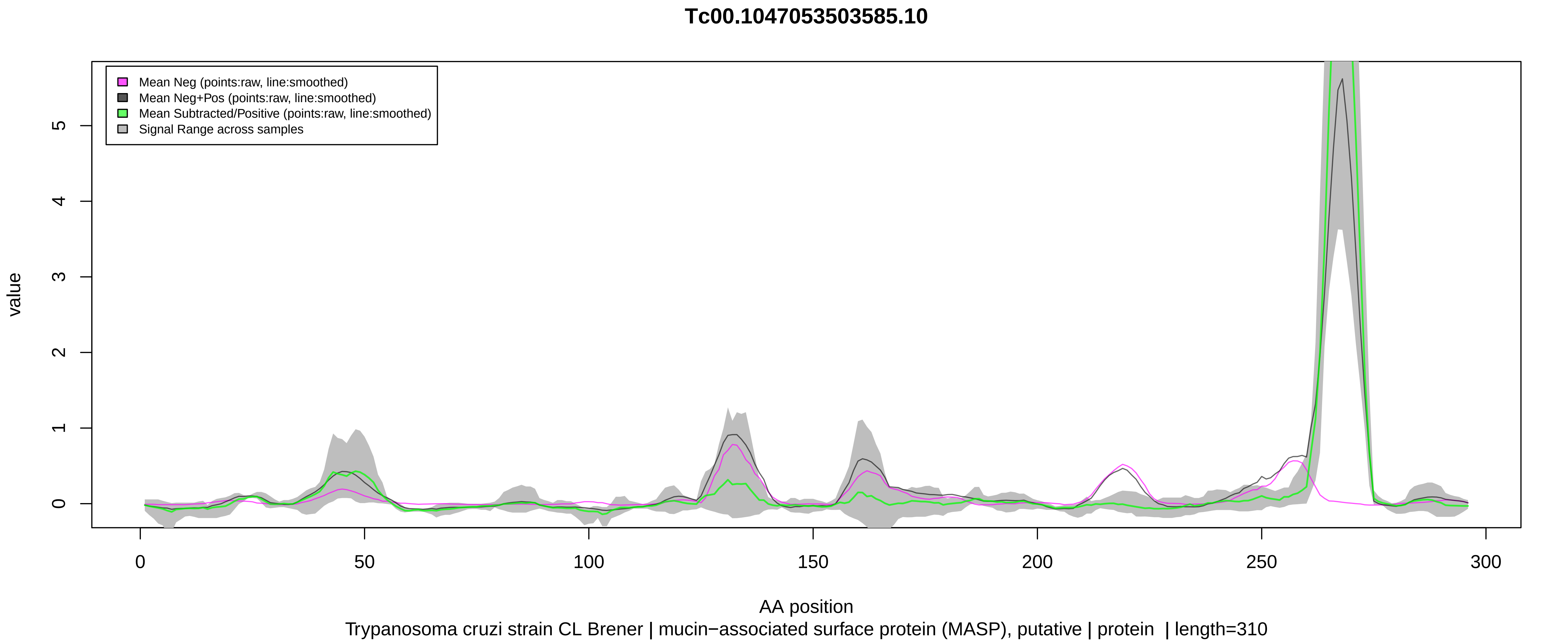
Tc00.1047053503585.10
Mean Neg (points:raw, line:smoothed)
Mean Neg+Pos (points:raw, line:smoothed)
Mean Subtracted/Positive (points:raw, line:smoothed)
Signal Range across samples
value
0
1
2
3
4
5
0
50
100
150
200
250
300
AA position
Trypanosoma cruzi strain CL Brener | mucin−associated surface protein (MASP), putative | protein | length=310

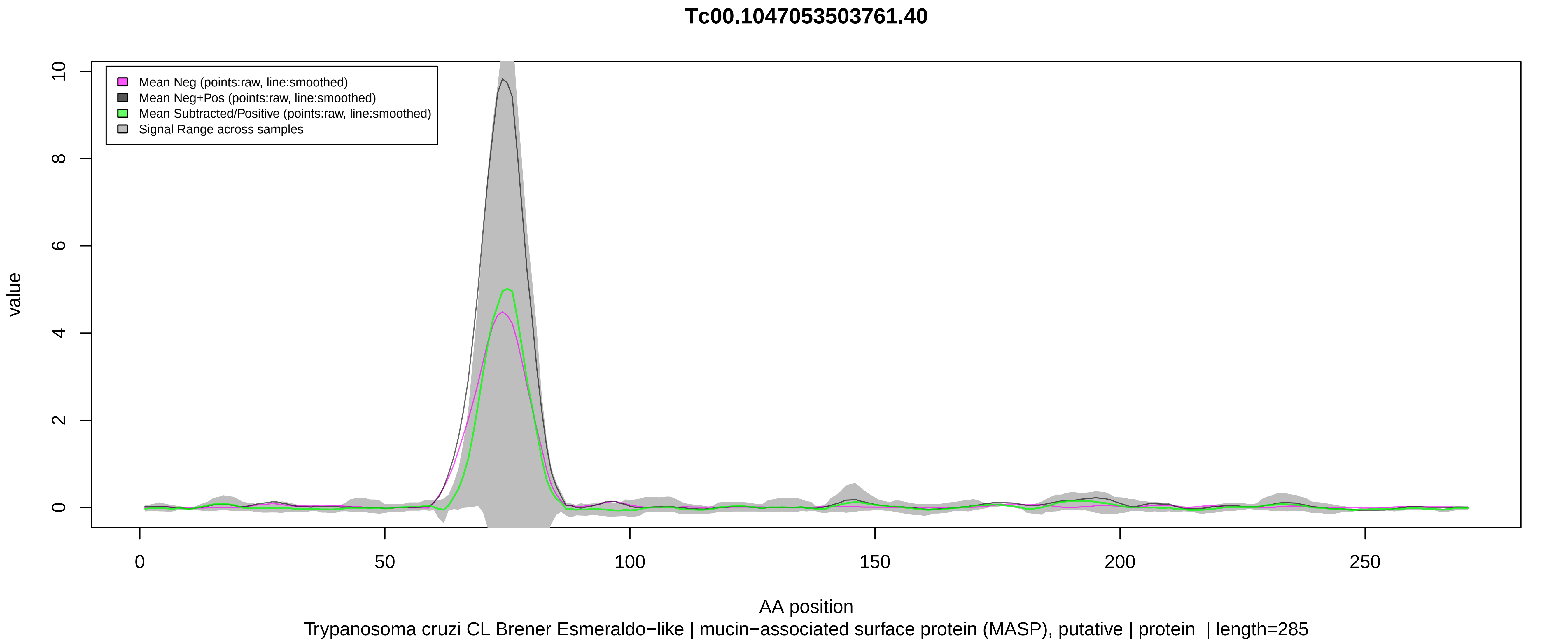

Tc00.1047053503761.40
Mean Neg (points:raw, line:smoothed)
Mean Neg+Pos (points:raw, line:smoothed)
Mean Subtracted/Positive (points:raw, line:smoothed)
Signal Range across samples
value
0
2
4
6
8
10
0
50
100
150
200
250
AA position
Trypanosoma cruzi CL Brener Esmeraldo−like | mucin−associated surface protein (MASP), putative | protein | length=285

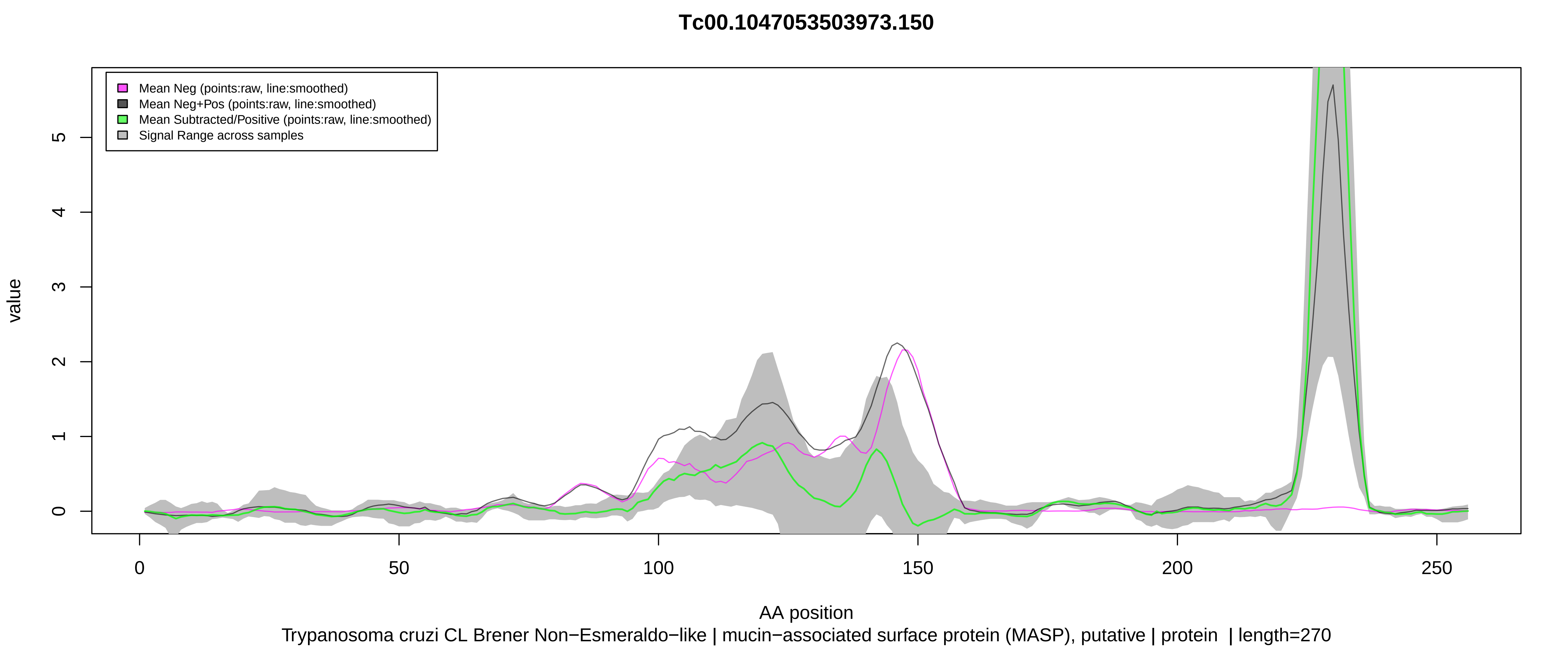

Tc00.1047053503973.150
Mean Neg (points:raw, line:smoothed)
Mean Neg+Pos (points:raw, line:smoothed)
Mean Subtracted/Positive (points:raw, line:smoothed)
Signal Range across samples
value
0
1
2
3
4
5
0
50
100
150
200
250
AA position
Trypanosoma cruzi CL Brener Non−Esmeraldo−like | mucin−associated surface protein (MASP), putative | protein | length=270

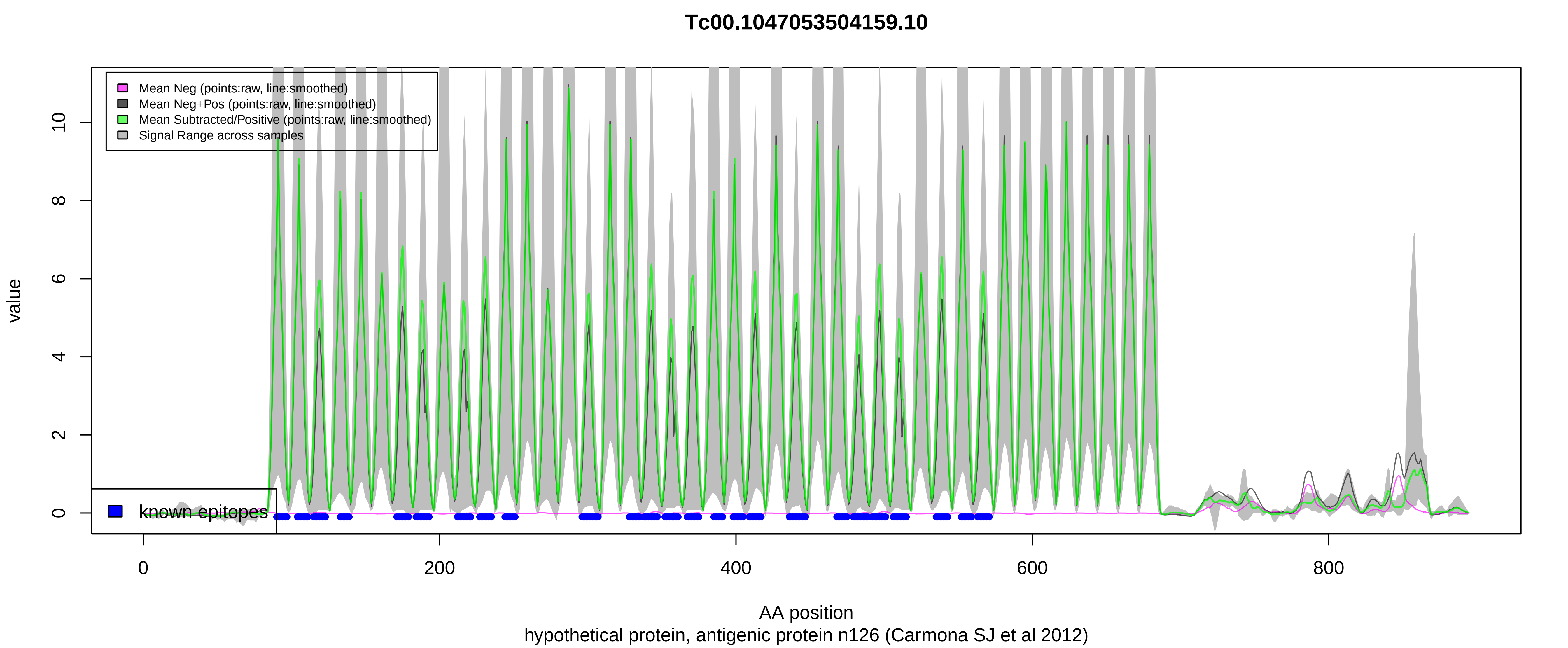
Tc00.1047053504159.10
Mean Neg (points:raw, line:smoothed)
Mean Neg+Pos (points:raw, line:smoothed)
Mean Subtracted/Positive (points:raw, line:smoothed)
Signal Range across samples
known epitopes
value
0
2
4
6
8
10
0
200
400
600
800
AA position
hypothetical protein, antigenic protein n126 (Carmona SJ et al 2012)

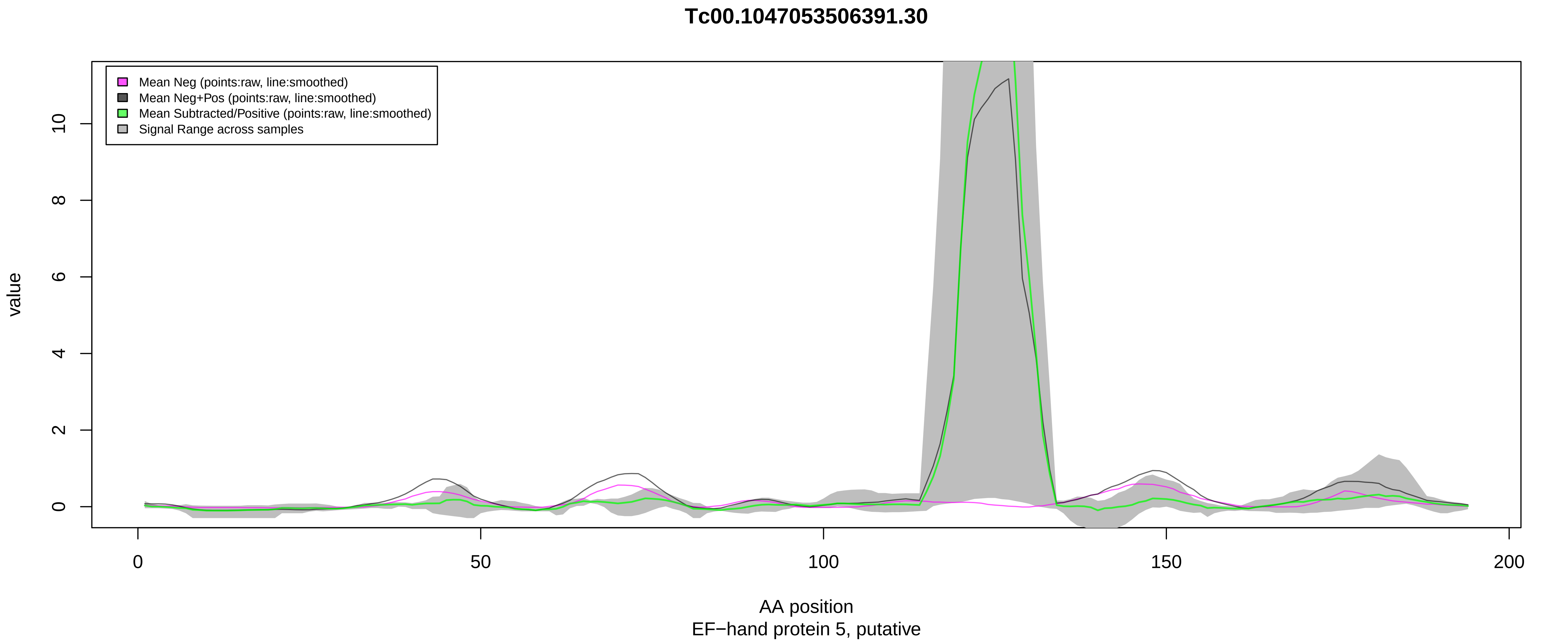

Tc00.1047053506391.30
Mean Neg (points:raw, line:smoothed)
Mean Neg+Pos (points:raw, line:smoothed)
Mean Subtracted/Positive (points:raw, line:smoothed)
Signal Range across samples
value
0
2
4
6
8
10
0
50
100
150
200
AA position
EF−hand protein 5, putative

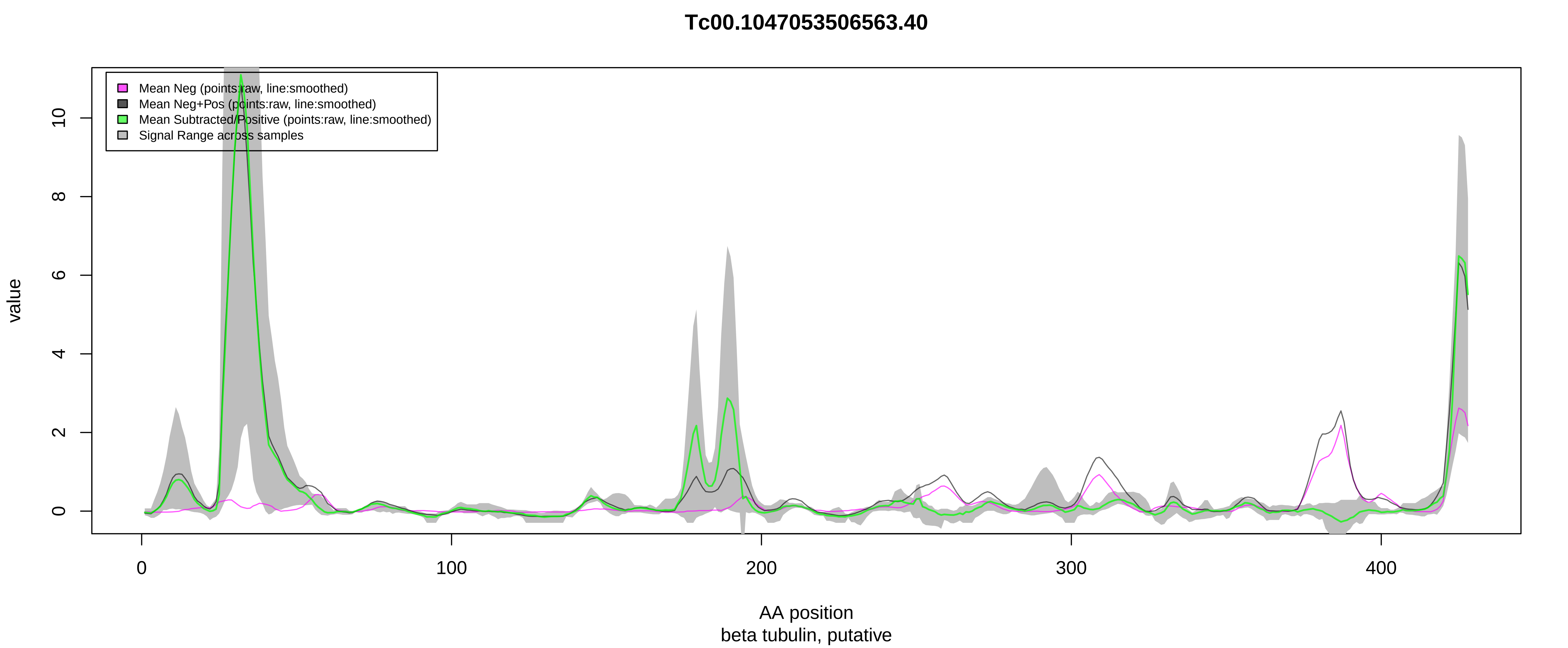
Tc00.1047053506563.40
Mean Neg (points:raw, line:smoothed)
Mean Neg+Pos (points:raw, line:smoothed)
Mean Subtracted/Positive (points:raw, line:smoothed)
Signal Range across samples
value
0
2
4
6
8
10
0
100
200
300
400
AA position
beta tubulin, putative

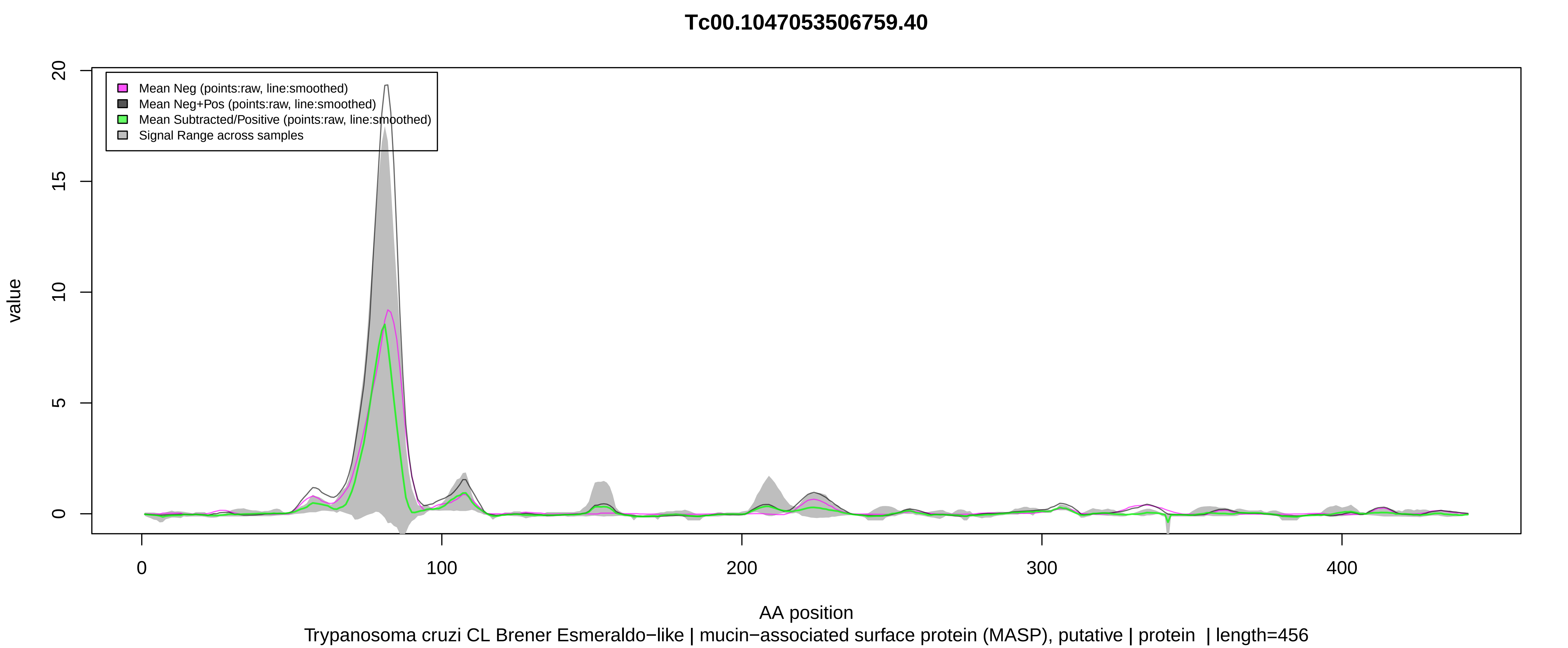

Tc00.1047053506759.40
Mean Neg (points:raw, line:smoothed)
Mean Neg+Pos (points:raw, line:smoothed)
Mean Subtracted/Positive (points:raw, line:smoothed)
Signal Range across samples
value
0
5
10
15
20
0
100
200
300
400
AA position
Trypanosoma cruzi CL Brener Esmeraldo−like | mucin−associated surface protein (MASP), putative | protein | length=456

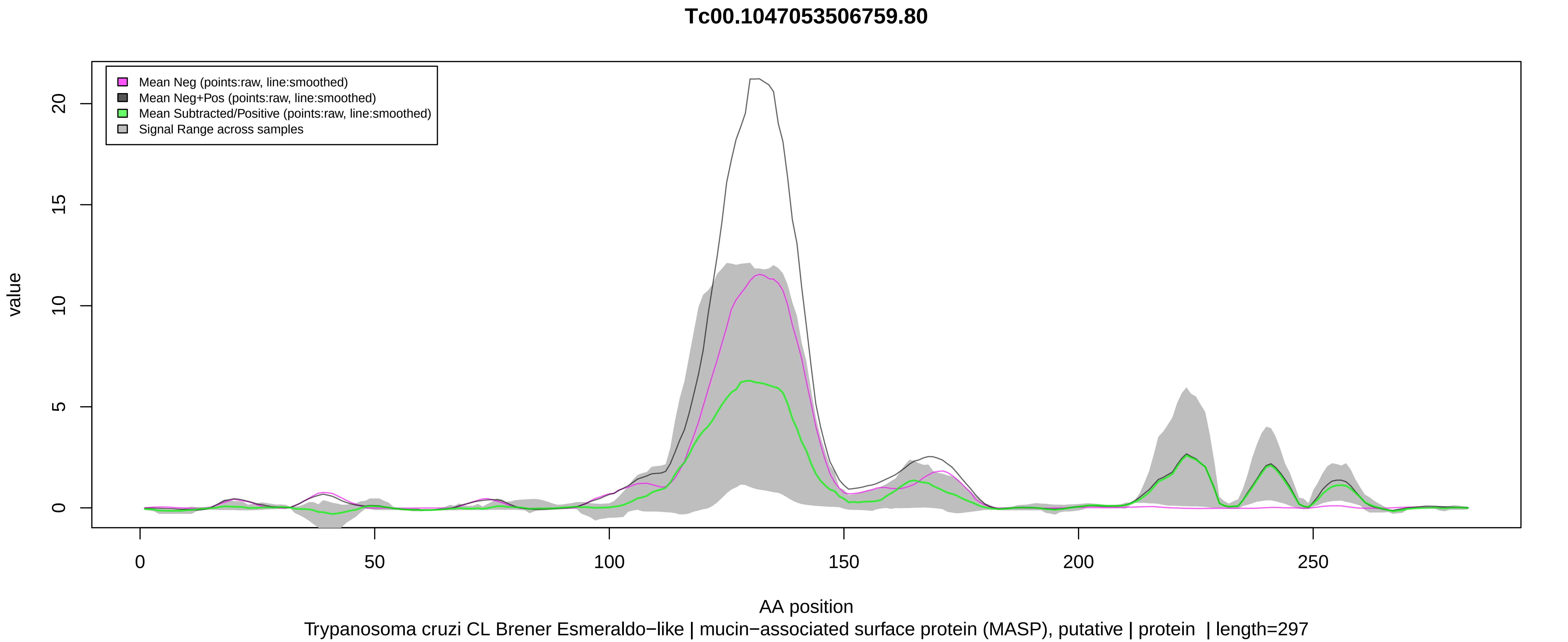
Tc00.1047053506759.80
Mean Neg (points:raw, line:smoothed)
Mean Neg+Pos (points:raw, line:smoothed)
Mean Subtracted/Positive (points:raw, line:smoothed)
Signal Range across samples
value
0
5
10
15
20
0
50
100
150
200
250
AA position
Trypanosoma cruzi CL Brener Esmeraldo−like | mucin−associated surface protein (MASP), putative | protein | length=297

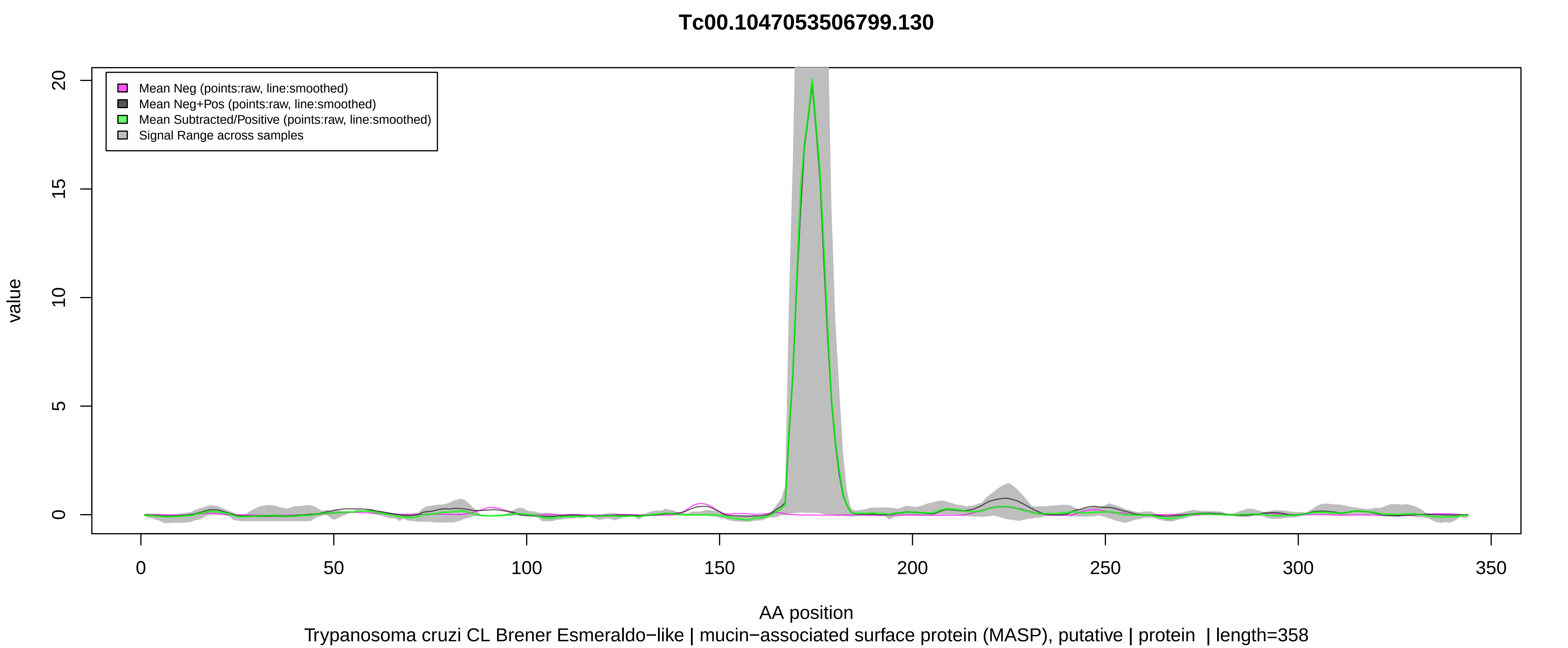

Tc00.1047053506799.130
Mean Neg (points:raw, line:smoothed)
Mean Neg+Pos (points:raw, line:smoothed)
Mean Subtracted/Positive (points:raw, line:smoothed)
Signal Range across samples
value
0
5
10
15
20
0
50
100
150
200
250
300
350
AA position
Trypanosoma cruzi CL Brener Esmeraldo−like | mucin−associated surface protein (MASP), putative | protein | length=358

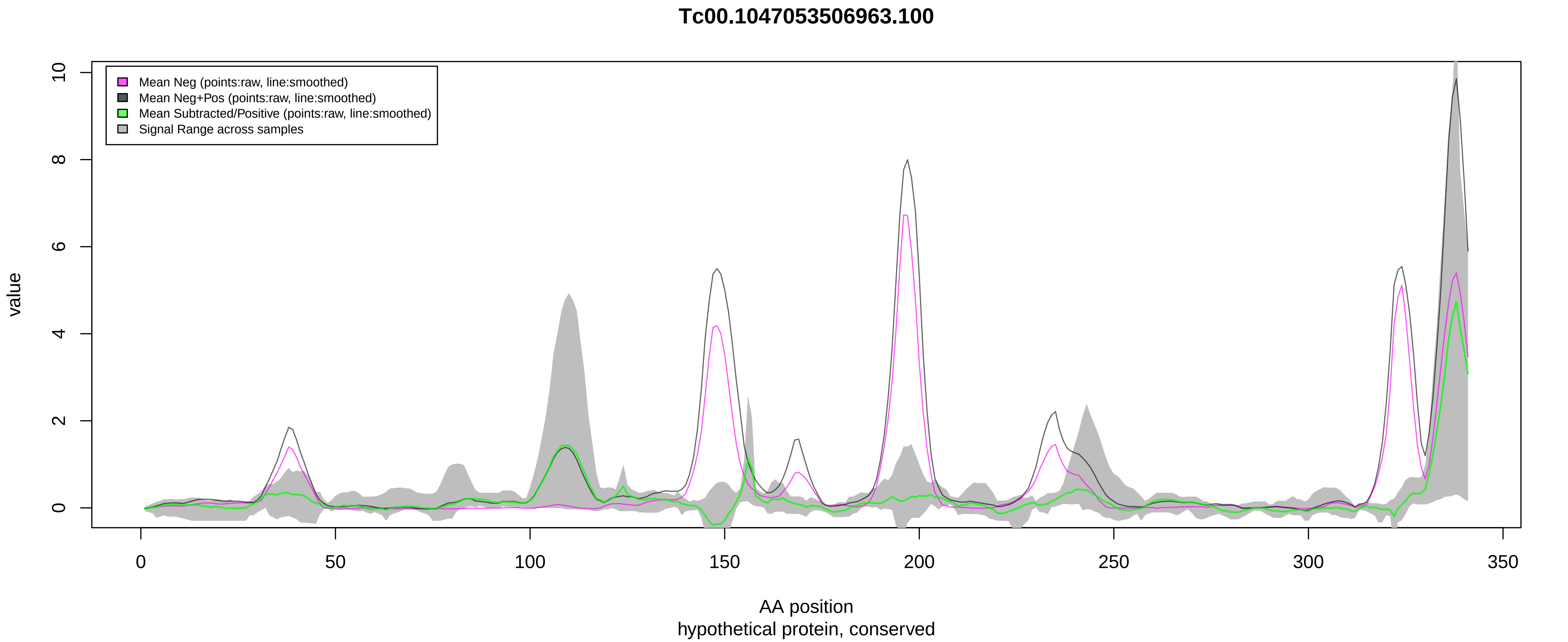

Tc00.1047053506963.100
Mean Neg (points:raw, line:smoothed)
Mean Neg+Pos (points:raw, line:smoothed)
Mean Subtracted/Positive (points:raw, line:smoothed)
Signal Range across samples
value
0
2
4
6
8
10
0
50
100
150
200
250
300
350
AA position
hypothetical protein, conserved

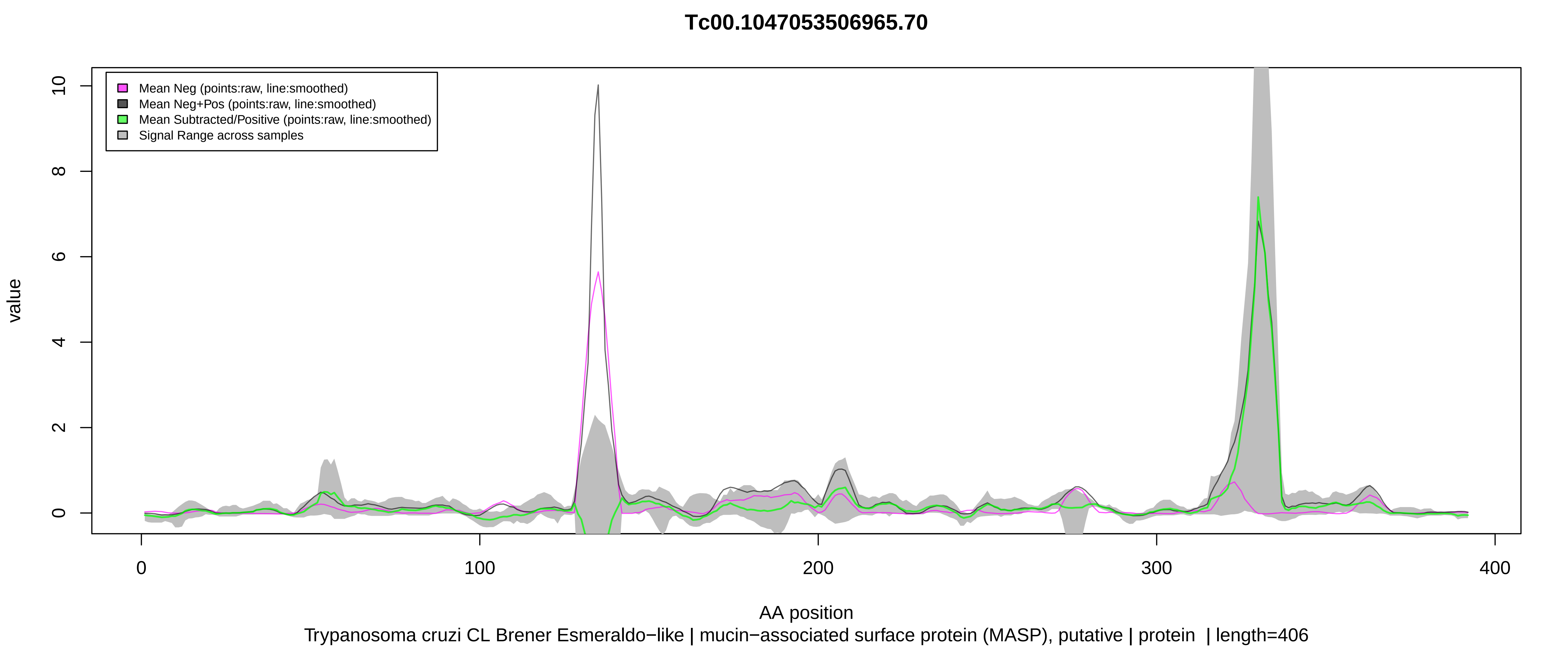
Tc00.1047053506965.70
Mean Neg (points:raw, line:smoothed)
Mean Neg+Pos (points:raw, line:smoothed)
Mean Subtracted/Positive (points:raw, line:smoothed)
Signal Range across samples
value
0
2
4
6
8
10
0
100
200
300
400
AA position
Trypanosoma cruzi CL Brener Esmeraldo−like | mucin−associated surface protein (MASP), putative | protein | length=406

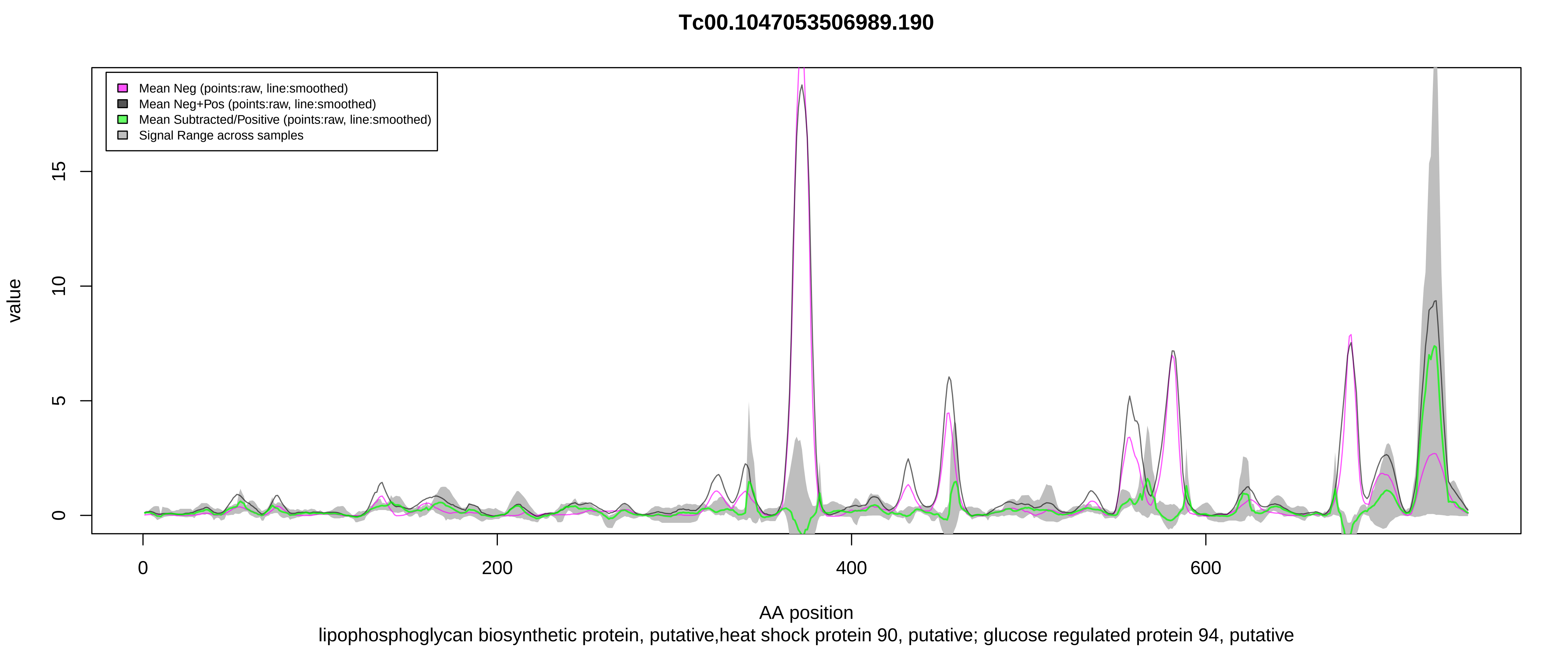
Tc00.1047053506989.190
Mean Neg (points:raw, line:smoothed)
Mean Neg+Pos (points:raw, line:smoothed)
Mean Subtracted/Positive (points:raw, line:smoothed)
Signal Range across samples
value
0
5
10
15
0
200
400
600
AA position
lipophosphoglycan biosynthetic protein, putative,heat shock protein 90, putative; glucose regulated protein 94, putative

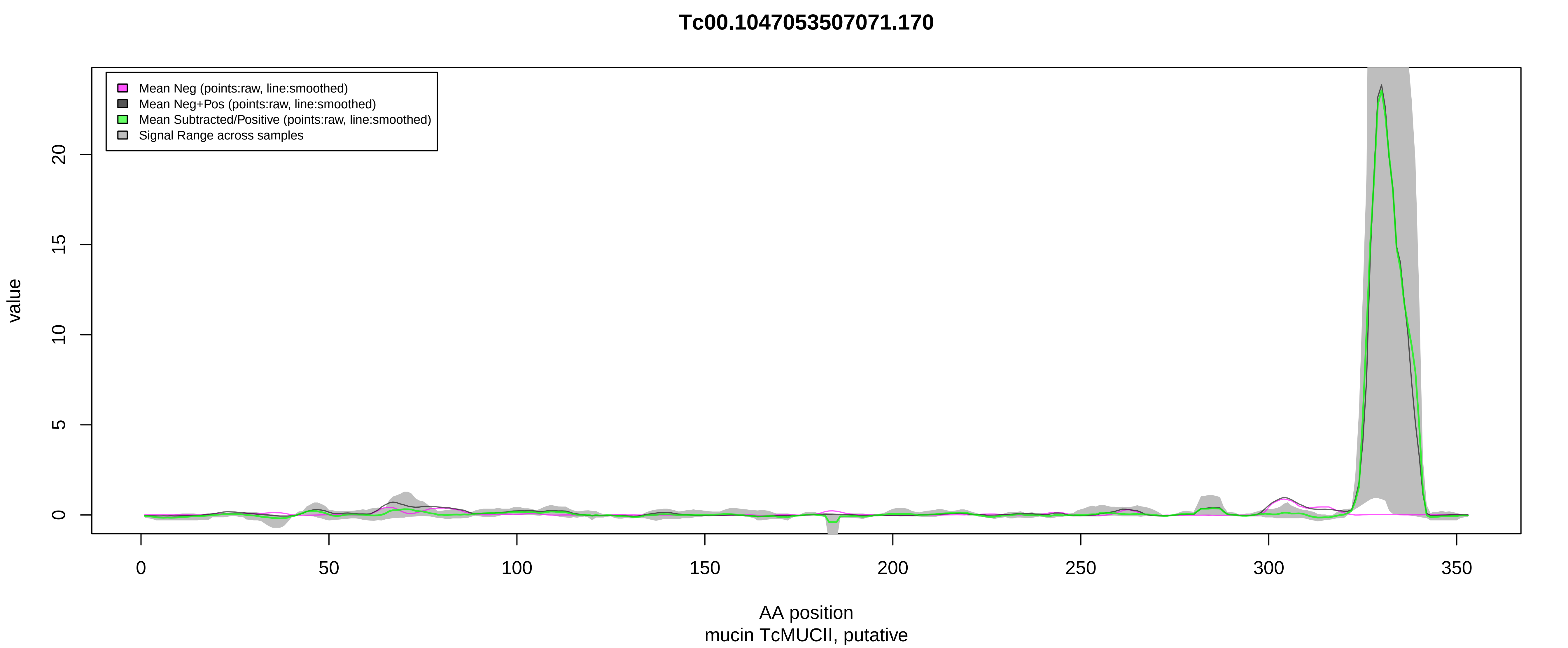
Tc00.1047053507071.170
Mean Neg (points:raw, line:smoothed)
Mean Neg+Pos (points:raw, line:smoothed)
Mean Subtracted/Positive (points:raw, line:smoothed)
Signal Range across samples
value
0
5
10
15
20
0
50
100
150
200
250
300
350
AA position
mucin TcMUCII, putative

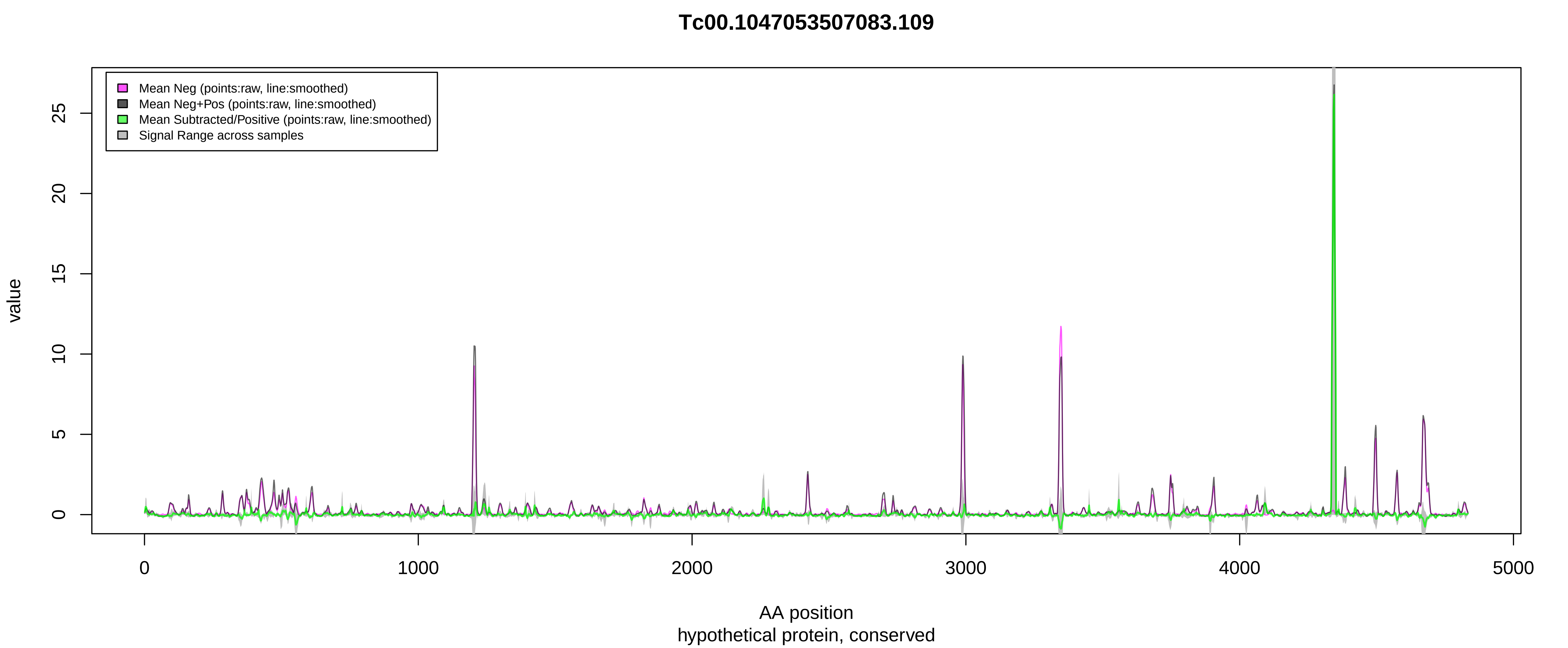
Tc00.1047053507083.109
Mean Neg (points:raw, line:smoothed)
Mean Neg+Pos (points:raw, line:smoothed)
Mean Subtracted/Positive (points:raw, line:smoothed)
Signal Range across samples
value
0
5
10
15
20
25
0
1000
2000
3000
4000
5000
AA position
hypothetical protein, conserved

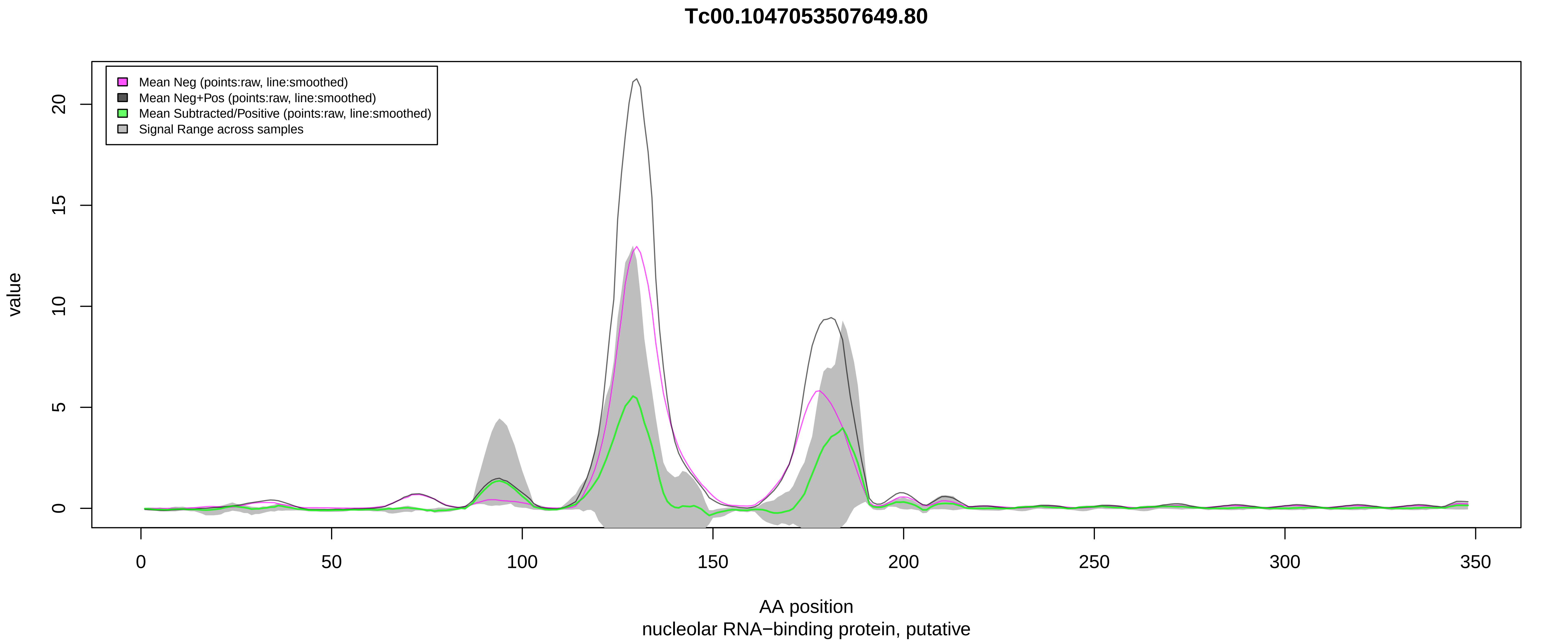
Tc00.1047053507649.80
Mean Neg (points:raw, line:smoothed)
Mean Neg+Pos (points:raw, line:smoothed)
Mean Subtracted/Positive (points:raw, line:smoothed)
Signal Range across samples
value
0
5
10
15
20
0
50
100
150
200
250
300
350
AA position
nucleolar RNA−binding protein, putative

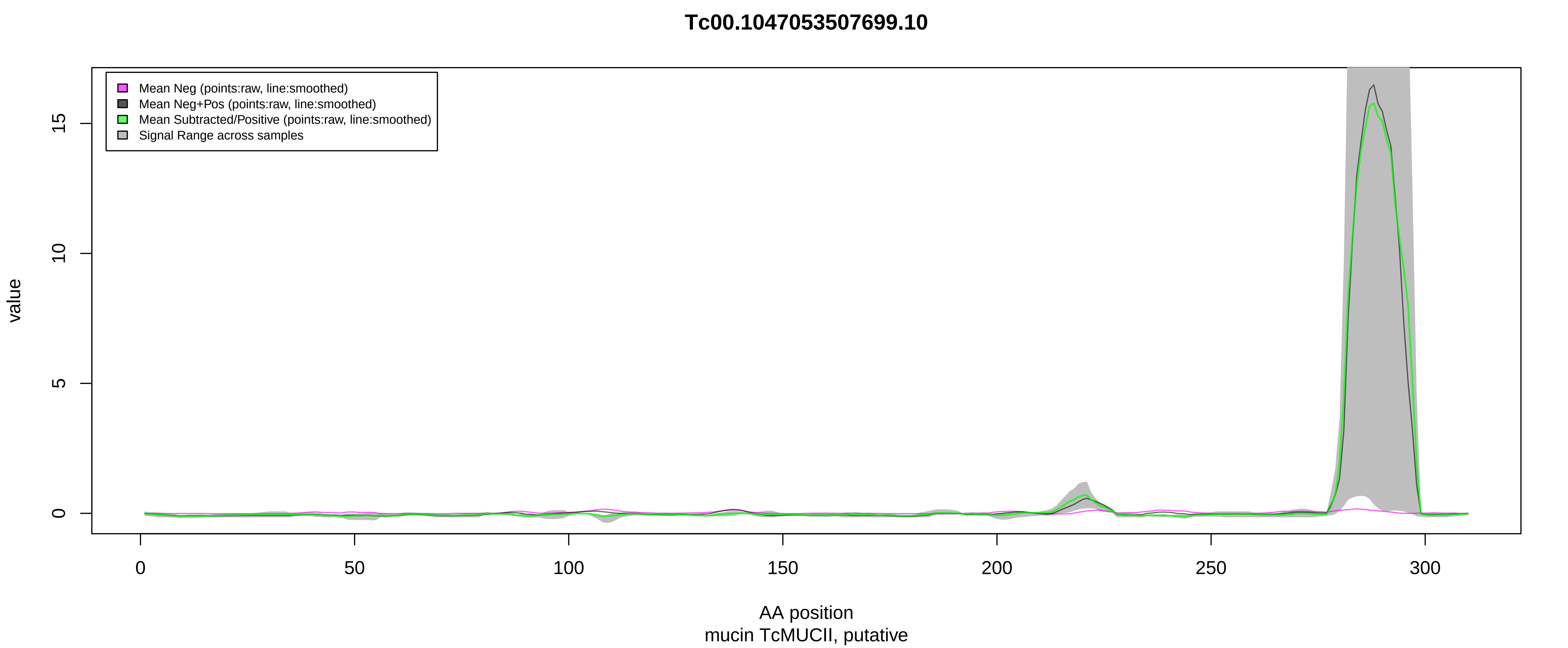

Tc00.1047053507699.10
Mean Neg (points:raw, line:smoothed)
Mean Neg+Pos (points:raw, line:smoothed)
Mean Subtracted/Positive (points:raw, line:smoothed)
Signal Range across samples
value
0
5
10
15
0
50
100
150
200
250
300
AA position
mucin TcMUCII, putative

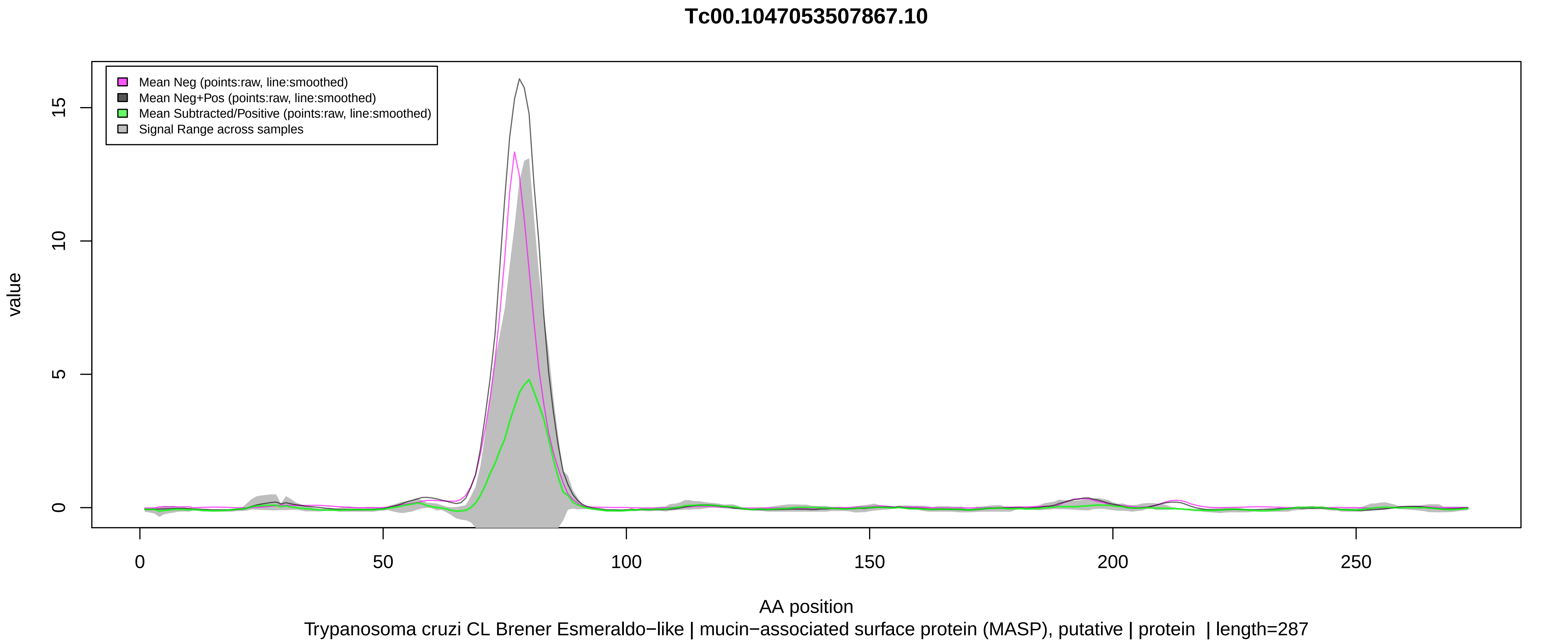

Tc00.1047053507867.10
Mean Neg (points:raw, line:smoothed)
Mean Neg+Pos (points:raw, line:smoothed)
Mean Subtracted/Positive (points:raw, line:smoothed)
Signal Range across samples
value
0
5
10
15
0
50
100
150
200
250
AA position
Trypanosoma cruzi CL Brener Esmeraldo−like | mucin−associated surface protein (MASP), putative | protein | length=287

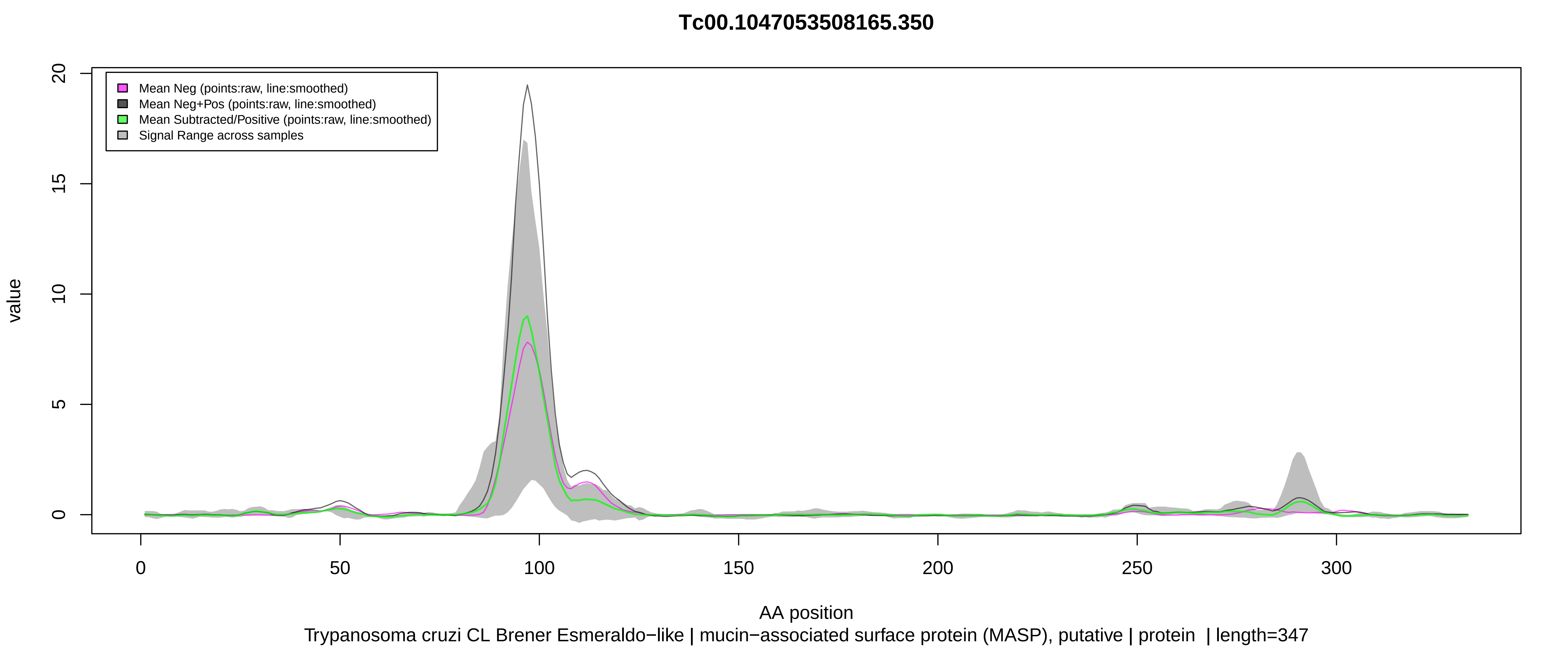
Tc00.1047053508165.350
Mean Neg (points:raw, line:smoothed)
Mean Neg+Pos (points:raw, line:smoothed)
Mean Subtracted/Positive (points:raw, line:smoothed)
Signal Range across samples
value
0
5
10
15
20
0
50
100
150
200
250
300
AA position
Trypanosoma cruzi CL Brener Esmeraldo−like | mucin−associated surface protein (MASP), putative | protein | length=347

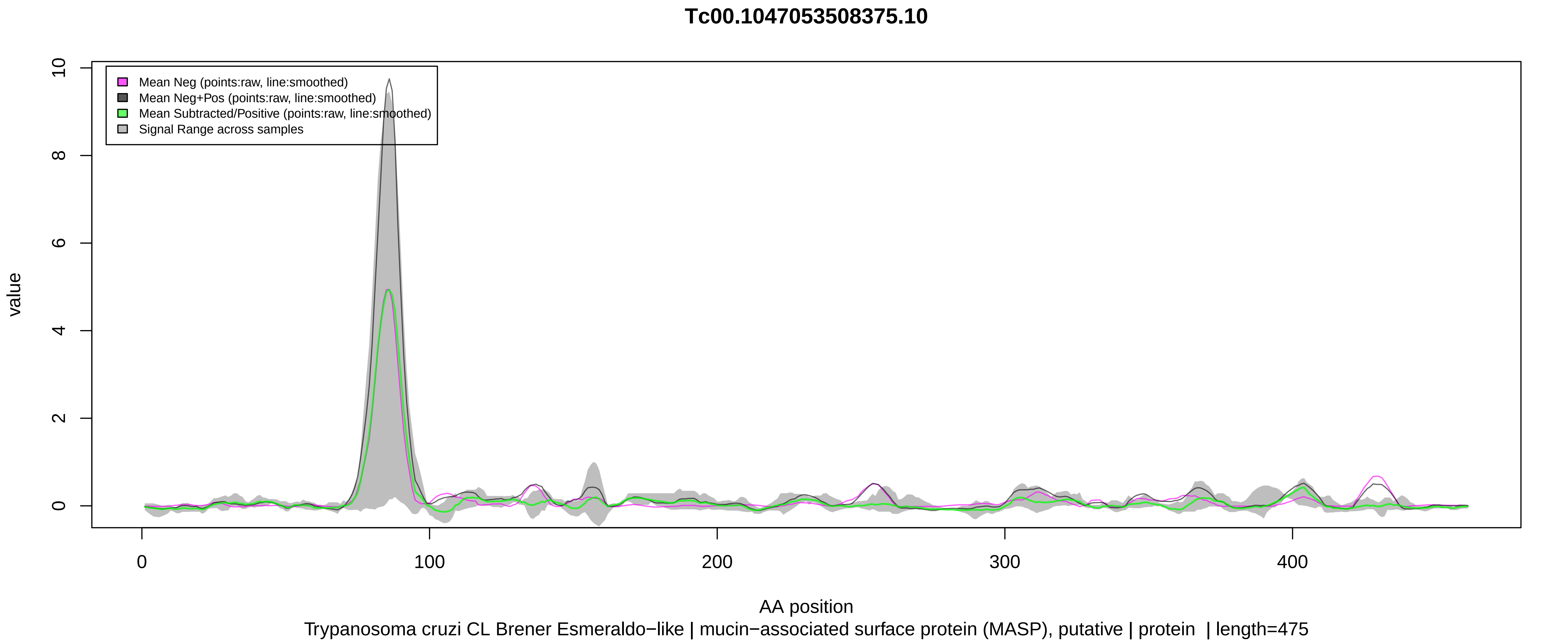

Tc00.1047053508375.10
Mean Neg (points:raw, line:smoothed)
Mean Neg+Pos (points:raw, line:smoothed)
Mean Subtracted/Positive (points:raw, line:smoothed)
Signal Range across samples
value
0
2
4
6
8
10
0
100
200
300
400
AA position
Trypanosoma cruzi CL Brener Esmeraldo−like | mucin−associated surface protein (MASP), putative | protein | length=475

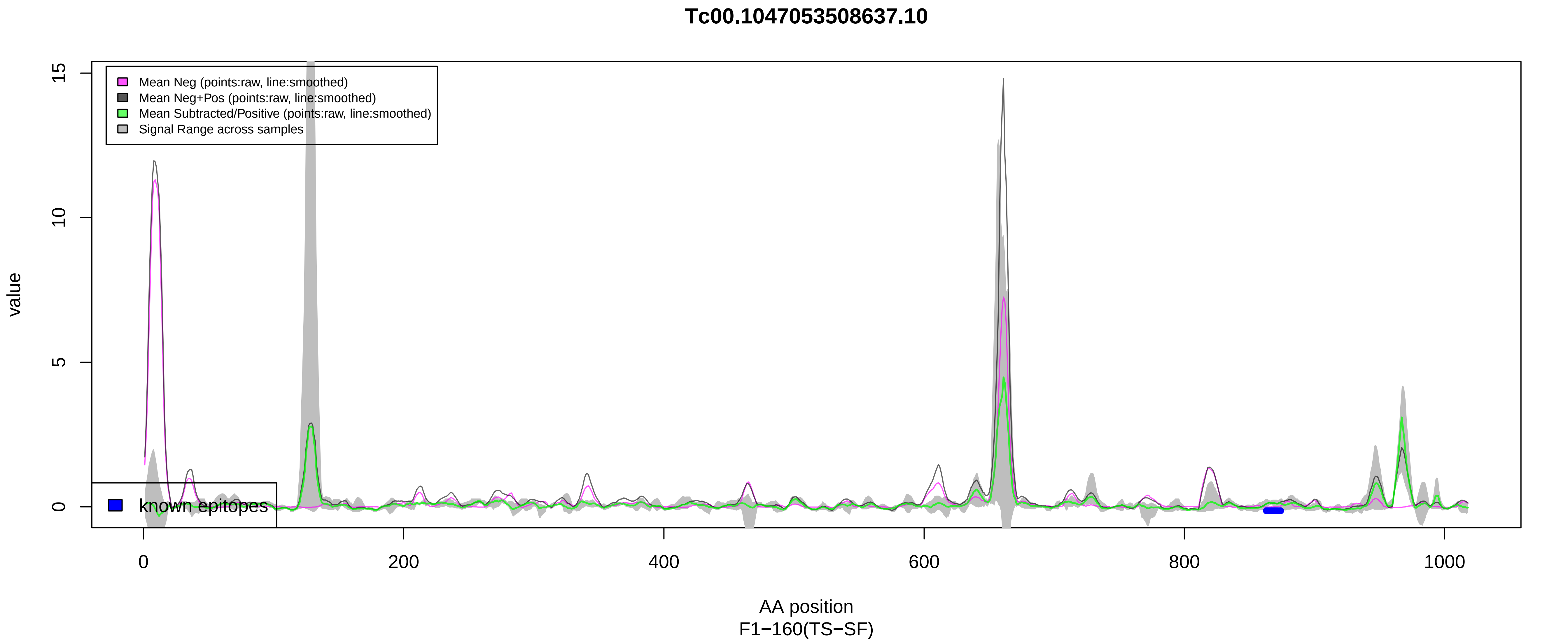
Tc00.1047053508637.10
Mean Neg (points:raw, line:smoothed)
Mean Neg+Pos (points:raw, line:smoothed)
Mean Subtracted/Positive (points:raw, line:smoothed)
Signal Range across samples
known epitopes
value
0
5
10
15
0
200
400
600
800
1000
AA position
F1−160(TS−SF)

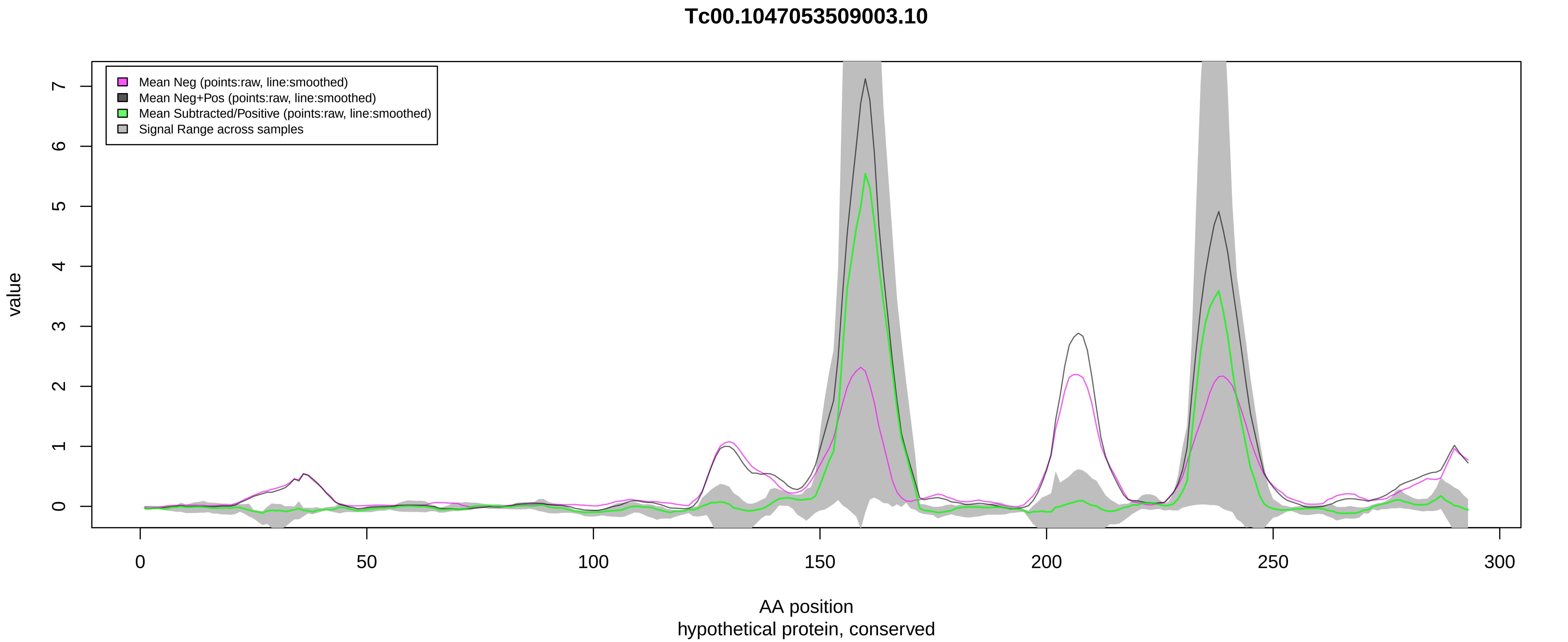

Tc00.1047053509003.10
Mean Neg (points:raw, line:smoothed)
Mean Neg+Pos (points:raw, line:smoothed)
Mean Subtracted/Positive (points:raw, line:smoothed)
Signal Range across samples
value
0
1
2
3
4
5
6
7
0
50
100
150
200
250
300
AA position
hypothetical protein, conserved

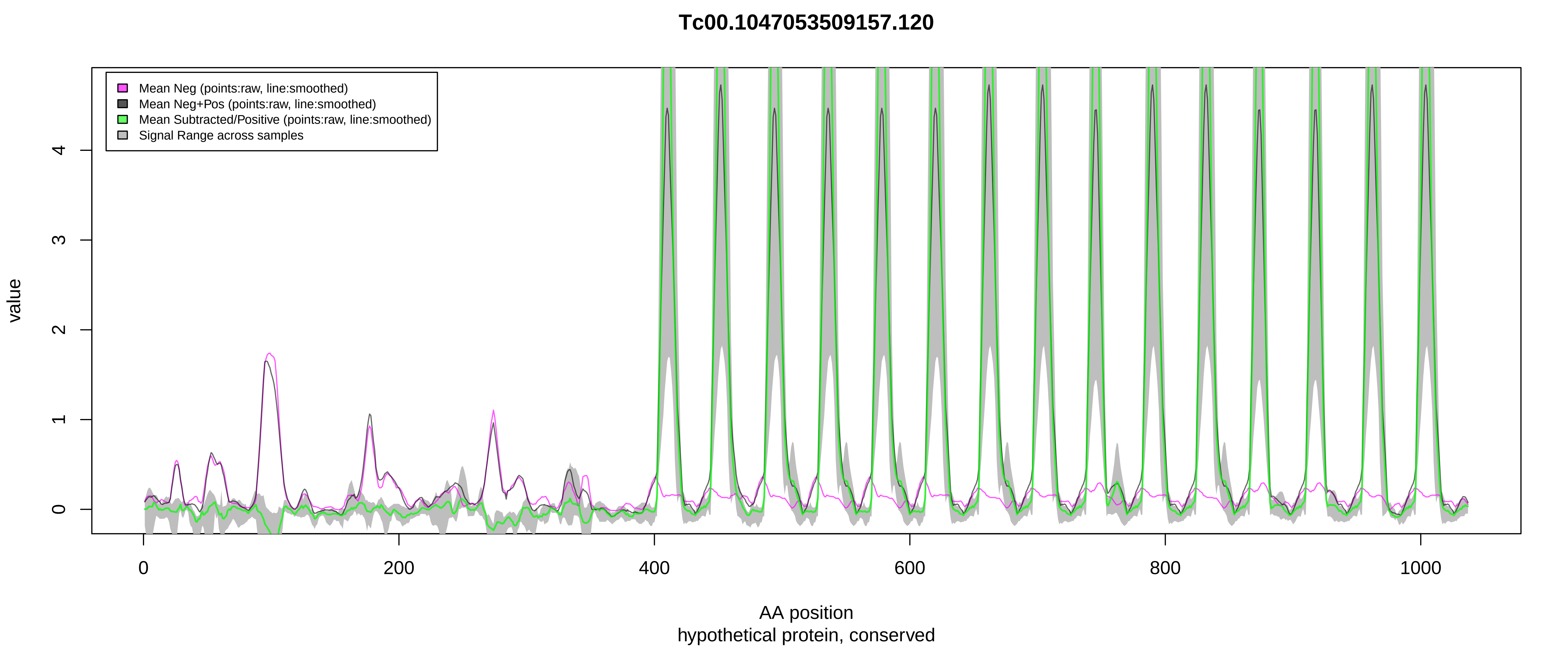

Tc00.1047053509157.120
Mean Neg (points:raw, line:smoothed)
Mean Neg+Pos (points:raw, line:smoothed)
Mean Subtracted/Positive (points:raw, line:smoothed)
Signal Range across samples
value
0
1
2
3
4
0
200
400
600
800
1000
AA position
hypothetical protein, conserved

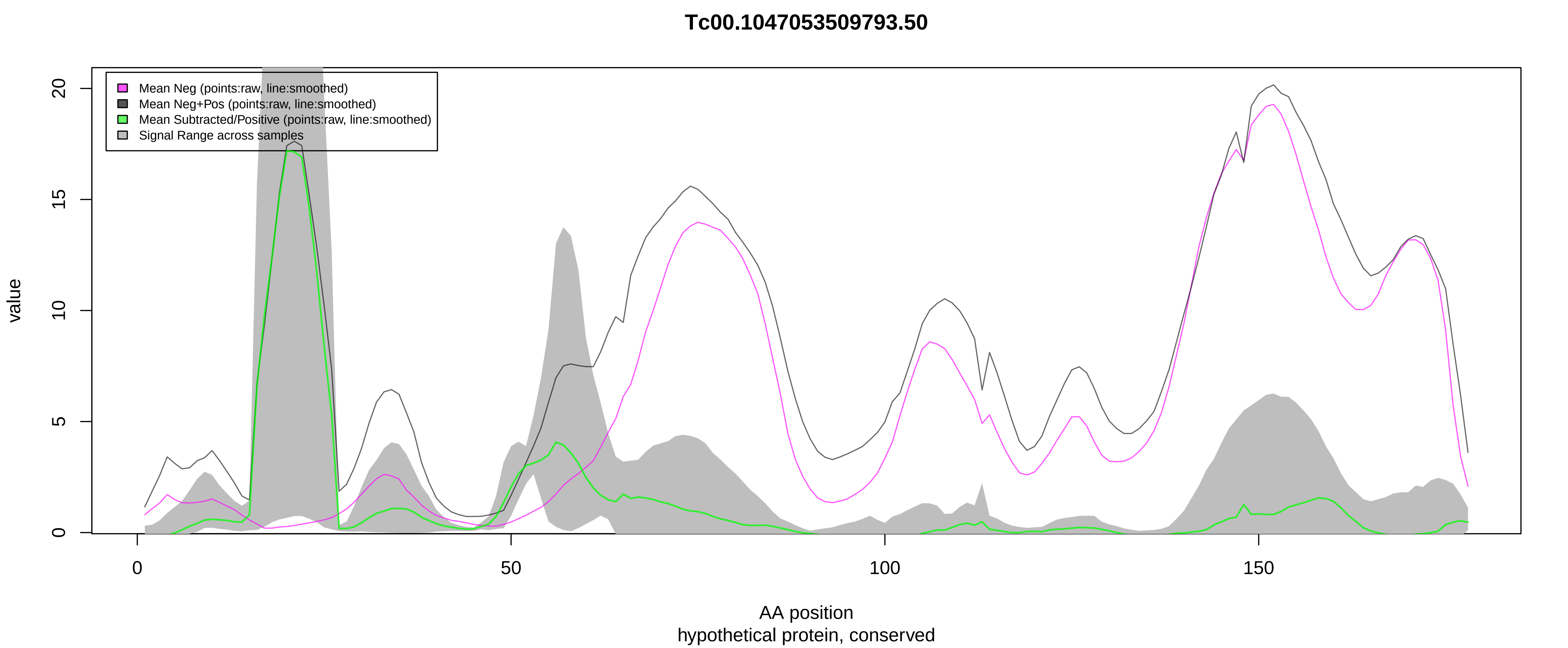
Tc00.1047053509793.50
Mean Neg (points:raw, line:smoothed)
Mean Neg+Pos (points:raw, line:smoothed)
Mean Subtracted/Positive (points:raw, line:smoothed)
Signal Range across samples
value
0
5
10
15
20
0
50
100
150
AA position
hypothetical protein, conserved

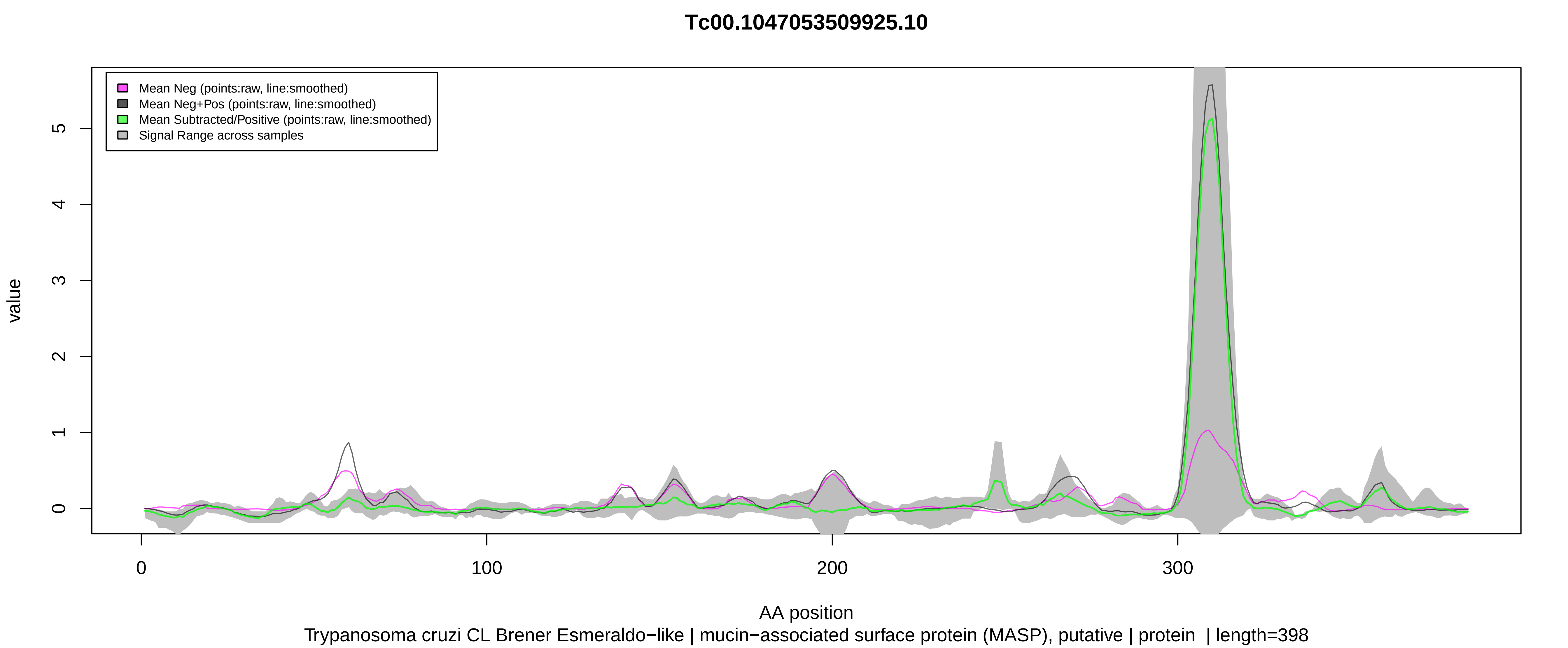

Tc00.1047053509925.10
Mean Neg (points:raw, line:smoothed)
Mean Neg+Pos (points:raw, line:smoothed)
Mean Subtracted/Positive (points:raw, line:smoothed)
Signal Range across samples
value
0
1
2
3
4
5
0
100
200
300
AA position
Trypanosoma cruzi CL Brener Esmeraldo−like | mucin−associated surface protein (MASP), putative | protein | length=398

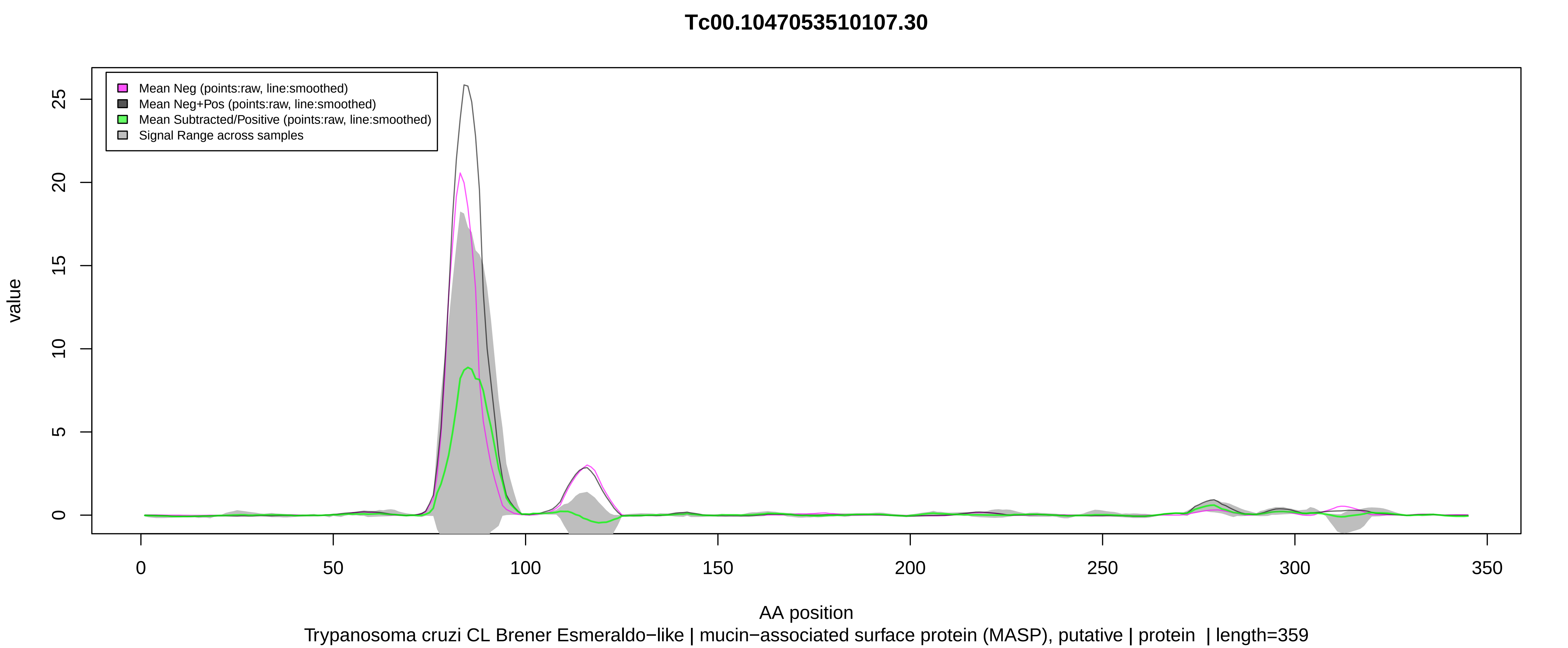

Tc00.1047053510107.30
Mean Neg (points:raw, line:smoothed)
Mean Neg+Pos (points:raw, line:smoothed)
Mean Subtracted/Positive (points:raw, line:smoothed)
Signal Range across samples
value
0
5
10
15
20
25
0
50
100
150
200
250
300
350
AA position
Trypanosoma cruzi CL Brener Esmeraldo−like | mucin−associated surface protein (MASP), putative | protein | length=359

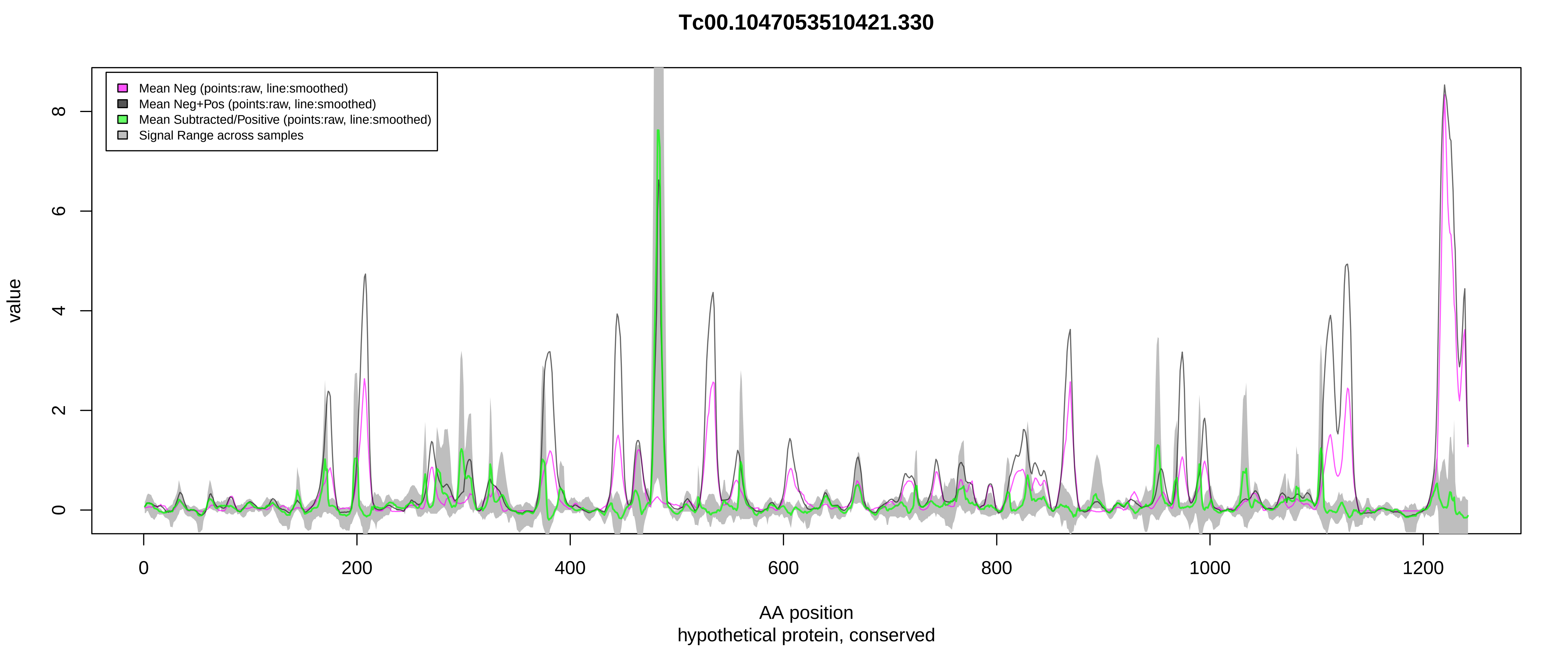

Tc00.1047053510421.330
Mean Neg (points:raw, line:smoothed)
Mean Neg+Pos (points:raw, line:smoothed)
Mean Subtracted/Positive (points:raw, line:smoothed)
Signal Range across samples
value
0
2
4
6
8
0
200
400
600
800
1000
1200
AA position
hypothetical protein, conserved

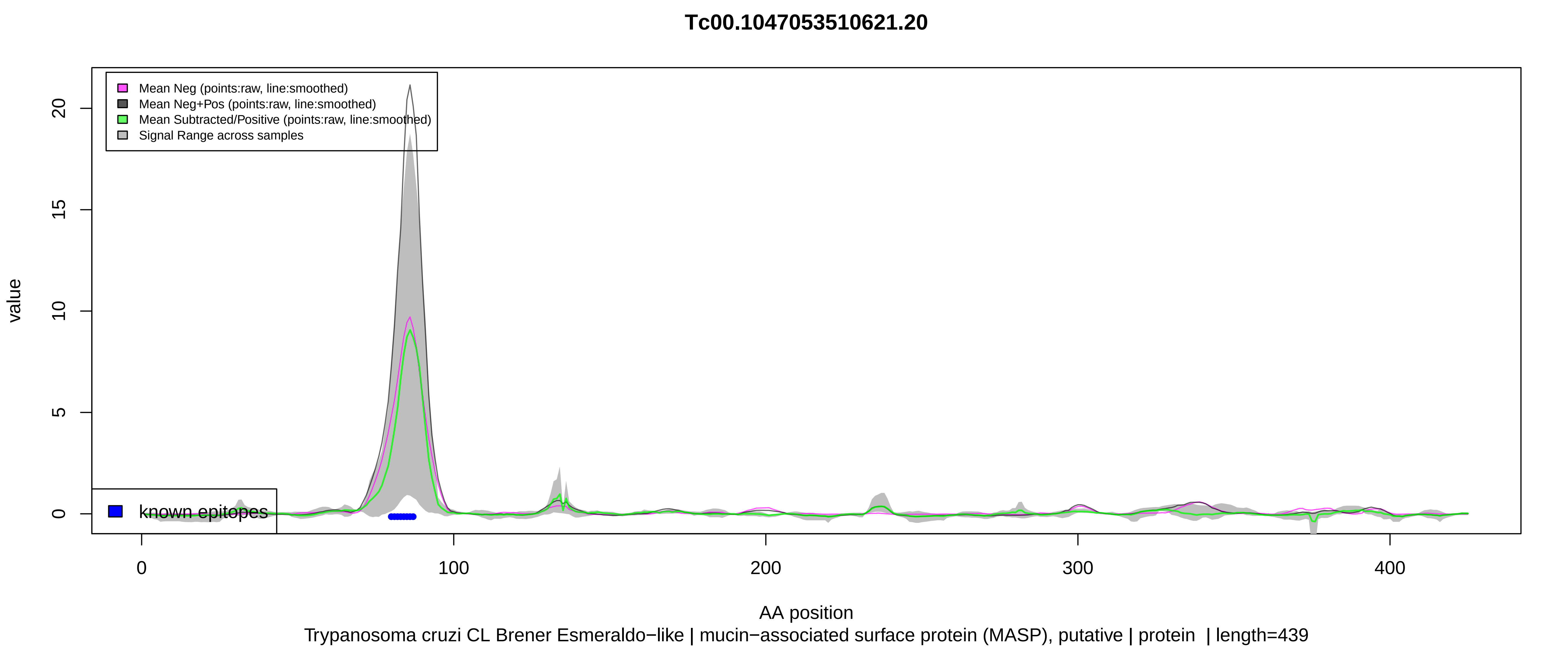

Tc00.1047053510621.20
Mean Neg (points:raw, line:smoothed)
Mean Neg+Pos (points:raw, line:smoothed)
Mean Subtracted/Positive (points:raw, line:smoothed)
Signal Range across samples
known epitopes
value
0
5
10
15
20
0
100
200
300
400
AA position
Trypanosoma cruzi CL Brener Esmeraldo−like | mucin−associated surface protein (MASP), putative | protein | length=439

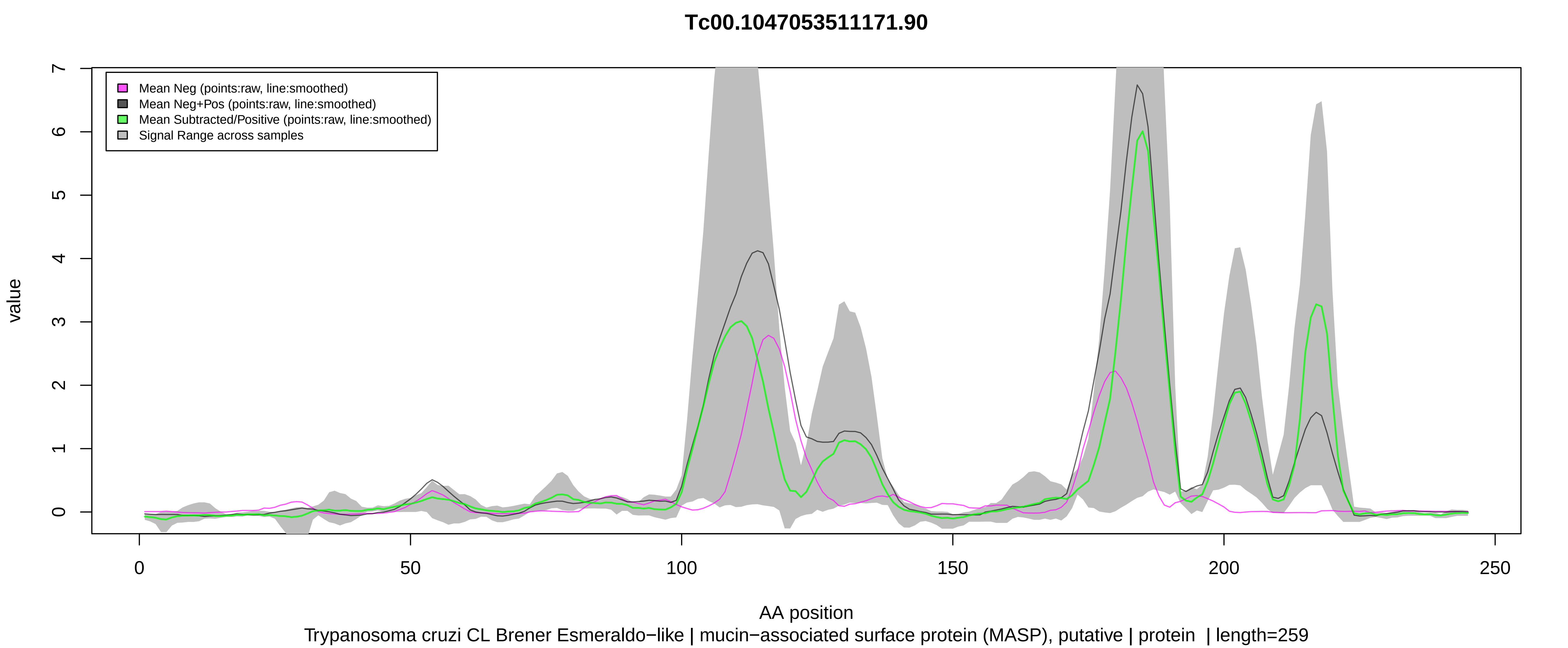
Tc00.1047053511171.90
Mean Neg (points:raw, line:smoothed)
Mean Neg+Pos (points:raw, line:smoothed)
Mean Subtracted/Positive (points:raw, line:smoothed)
Signal Range across samples
value
0
1
2
3
4
5
6
7
0
50
100
150
200
250
AA position
Trypanosoma cruzi CL Brener Esmeraldo−like | mucin−associated surface protein (MASP), putative | protein | length=259

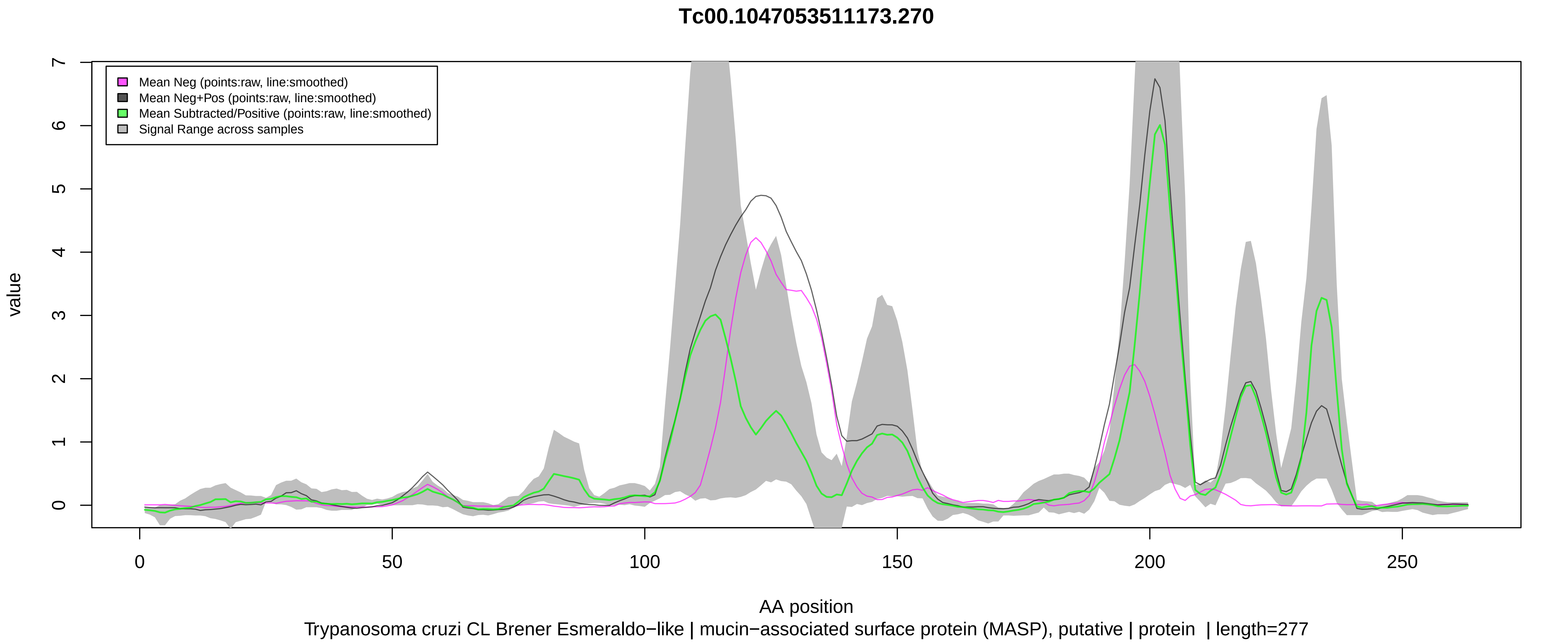

Tc00.1047053511173.270
Mean Neg (points:raw, line:smoothed)
Mean Neg+Pos (points:raw, line:smoothed)
Mean Subtracted/Positive (points:raw, line:smoothed)
Signal Range across samples
value
0
1
2
3
4
5
6
7
0
50
100
150
200
250
AA position
Trypanosoma cruzi CL Brener Esmeraldo−like | mucin−associated surface protein (MASP), putative | protein | length=277

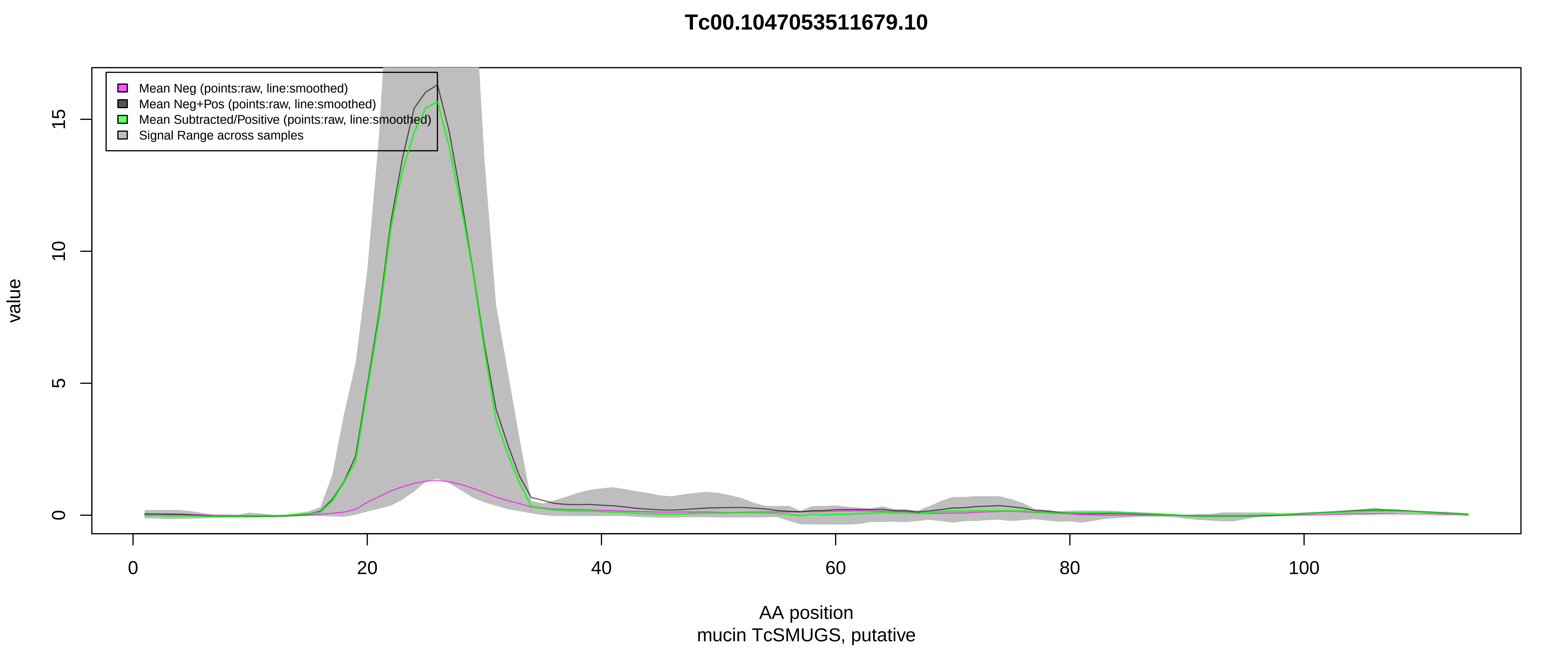
Tc00.1047053511679.10
Mean Neg (points:raw, line:smoothed)
Mean Neg+Pos (points:raw, line:smoothed)
Mean Subtracted/Positive (points:raw, line:smoothed)
Signal Range across samples
value
0
5
10
15
0
20
40
60
80
100
AA position
mucin TcSMUGS, putative

Tc00.1047053503665.30

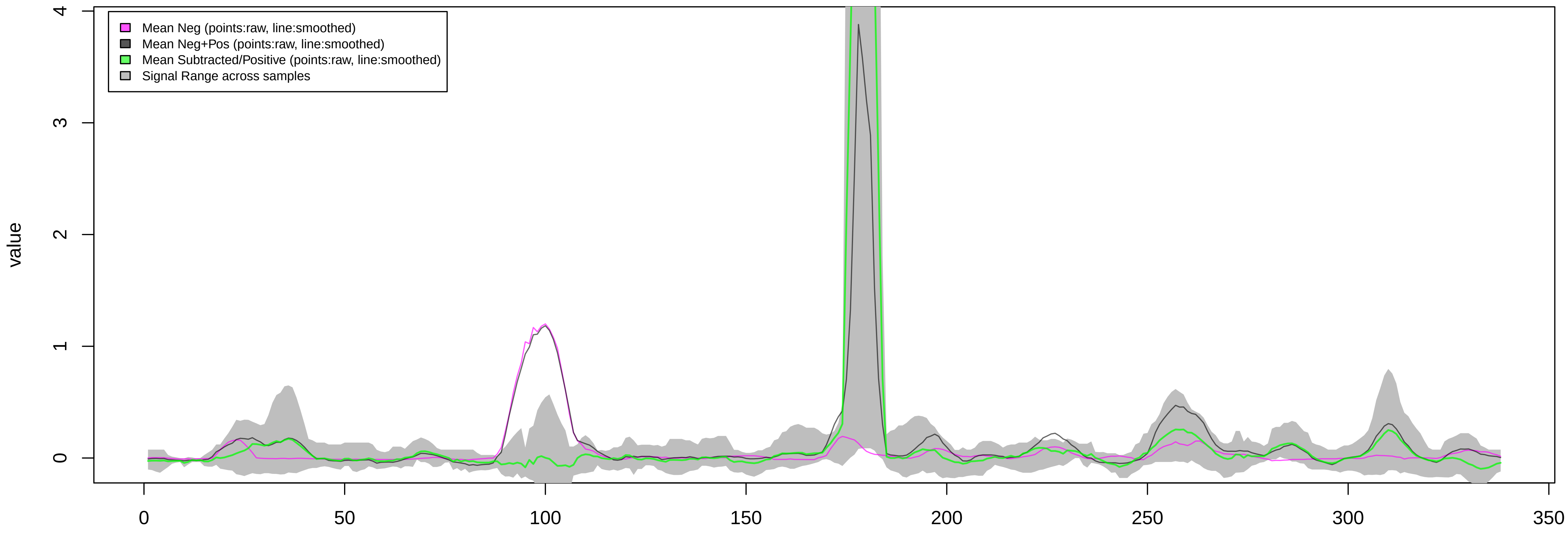

Trypanosoma cruzi CL Brener Esmeraldo−like | mucin−associated surface protein (MASP), putative | protein | length=352

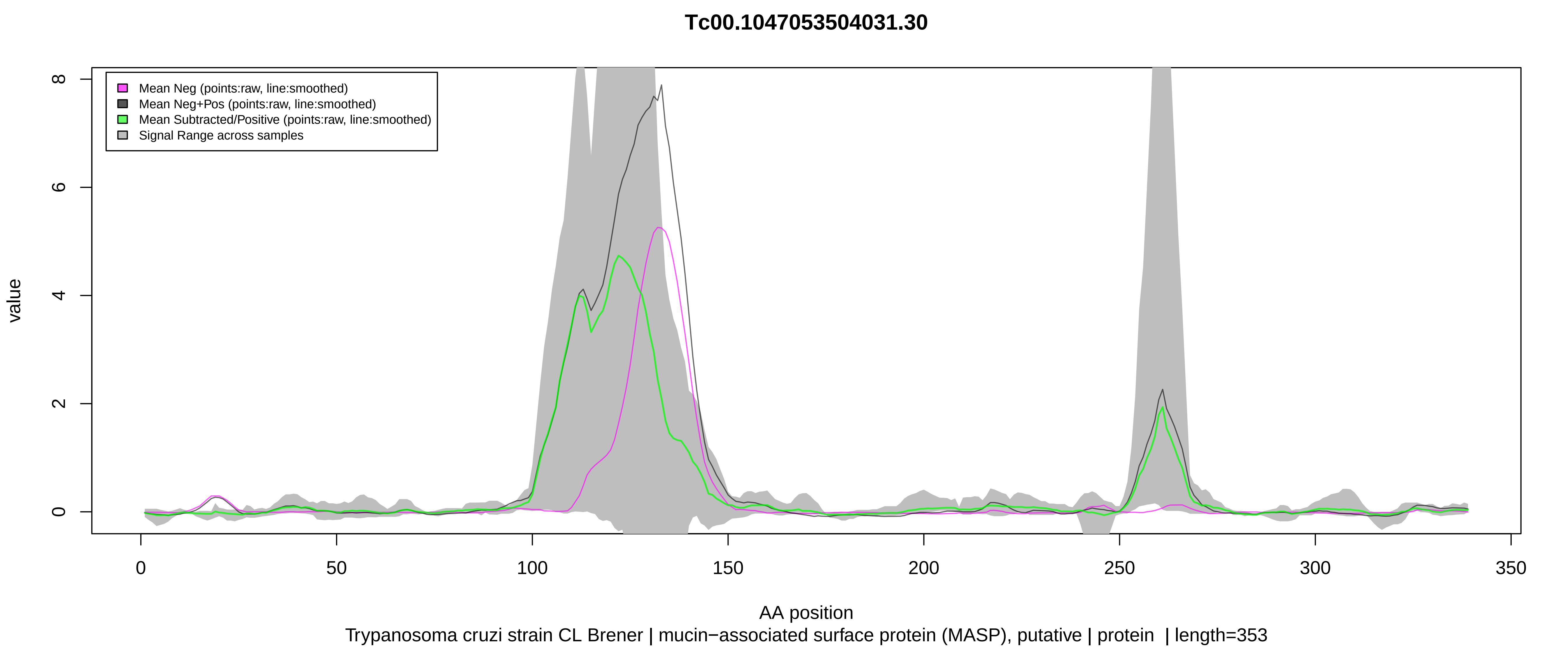

Tc00.1047053504031.30
Mean Neg (points:raw, line:smoothed)
Mean Neg+Pos (points:raw, line:smoothed)
Mean Subtracted/Positive (points:raw, line:smoothed)
Signal Range across samples
value
0
2
4
6
8
0
50
100
150
200
250
300
350
AA position
Trypanosoma cruzi strain CL Brener | mucin−associated surface protein (MASP), putative | protein | length=353

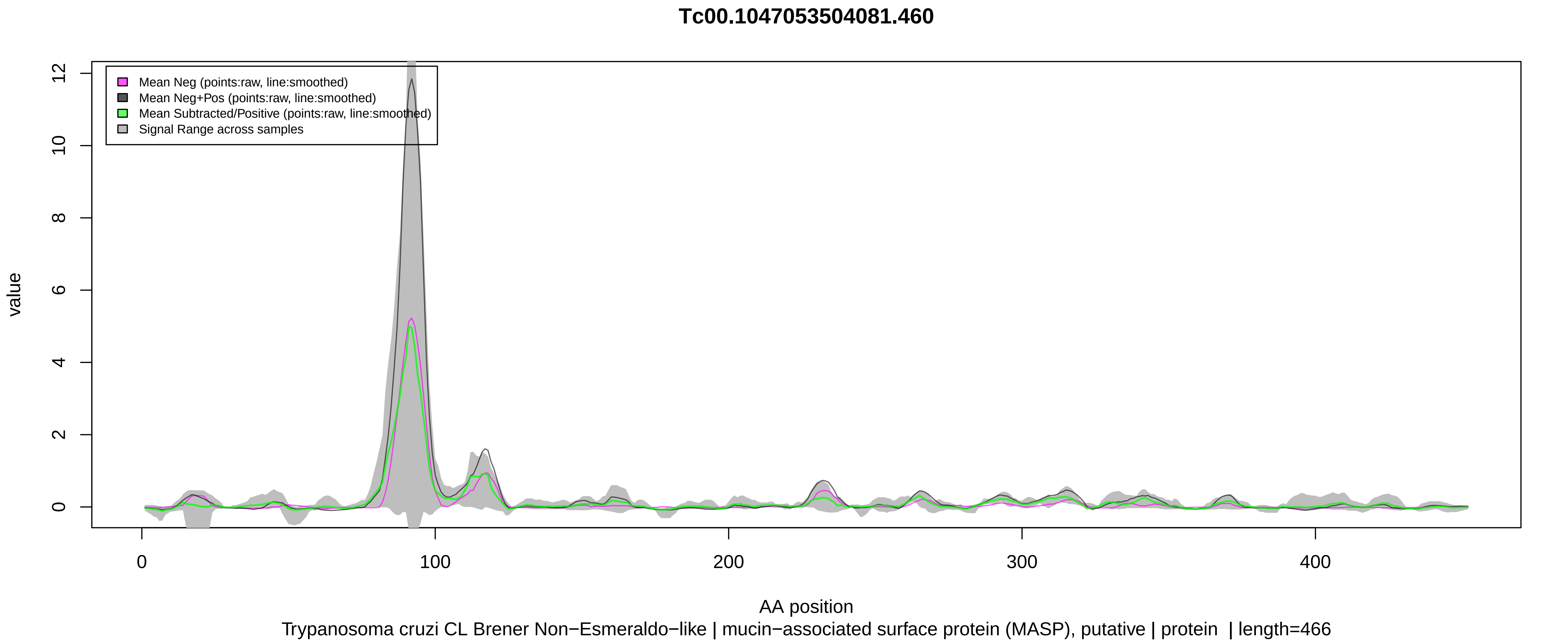
Tc00.1047053504081.460
Mean Neg (points:raw, line:smoothed)
Mean Neg+Pos (points:raw, line:smoothed)
Mean Subtracted/Positive (points:raw, line:smoothed)
Signal Range across samples
value
AA position
0
2
4
6
8
10
12
0
100
200
300
400
Trypanosoma cruzi CL Brener Non−Esmeraldo−like | mucin−associated surface protein (MASP), putative | protein | length=466

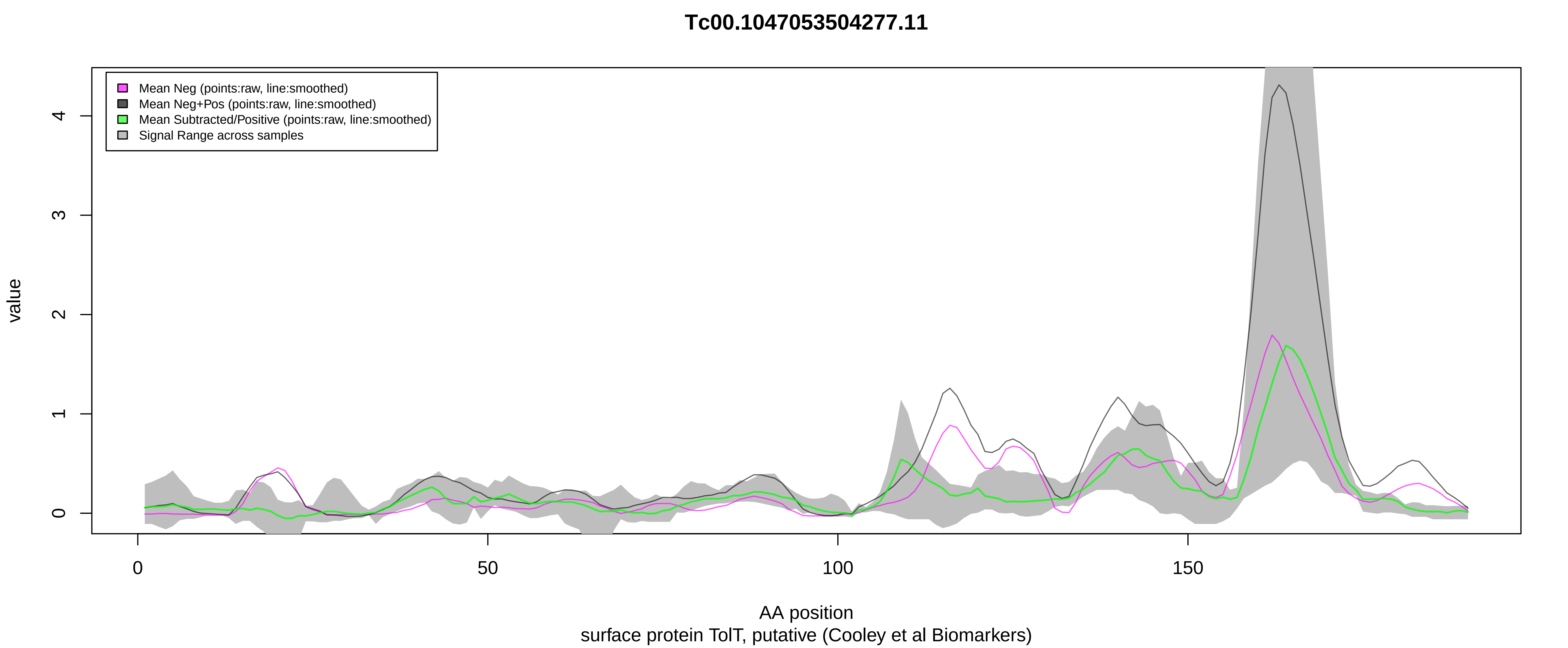

Tc00.1047053504277.11
Mean Neg (points:raw, line:smoothed)
Mean Neg+Pos (points:raw, line:smoothed)
Mean Subtracted/Positive (points:raw, line:smoothed)
Signal Range across samples
value
0
1
2
3
4
0
50
100
150
AA position
surface protein TolT, putative (Cooley et al Biomarkers)

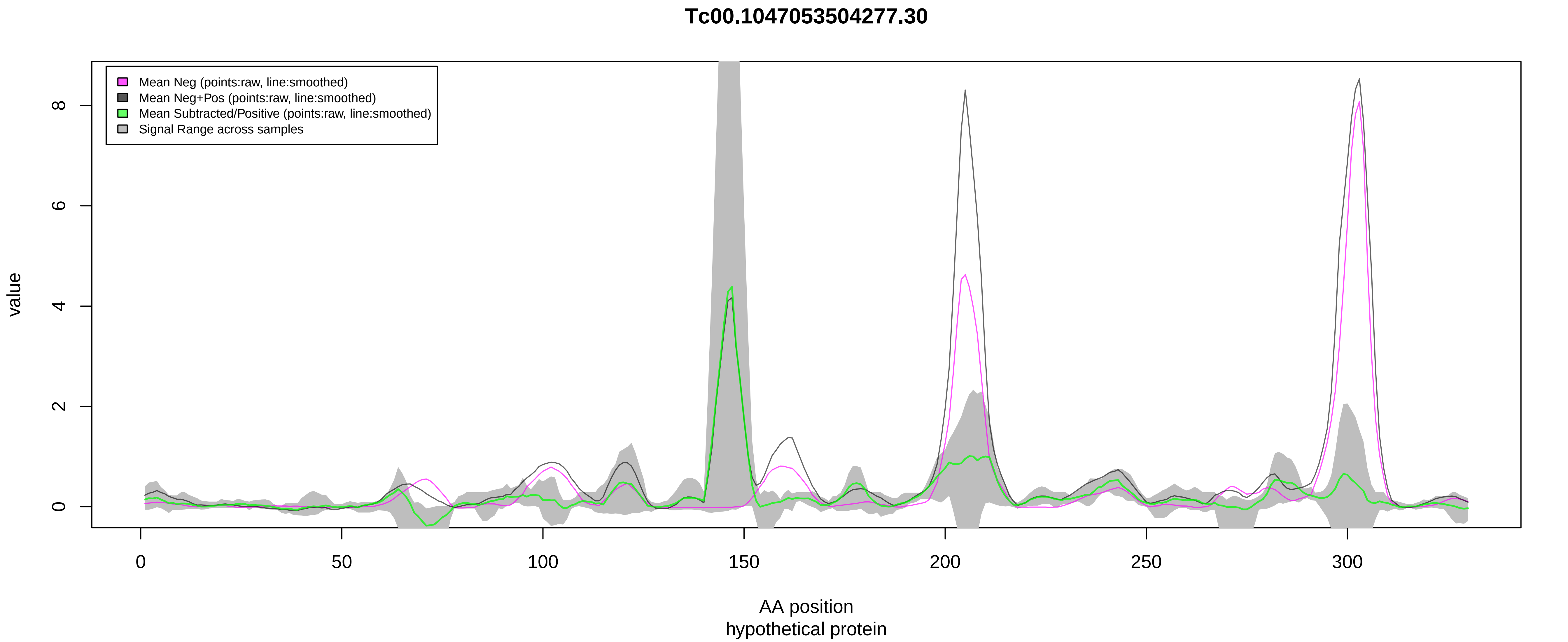

Tc00.1047053504277.30
Mean Neg (points:raw, line:smoothed)
Mean Neg+Pos (points:raw, line:smoothed)
Mean Subtracted/Positive (points:raw, line:smoothed)
Signal Range across samples
value
0
2
4
6
8
0
50
100
150
200
250
300
AA position
hypothetical protein

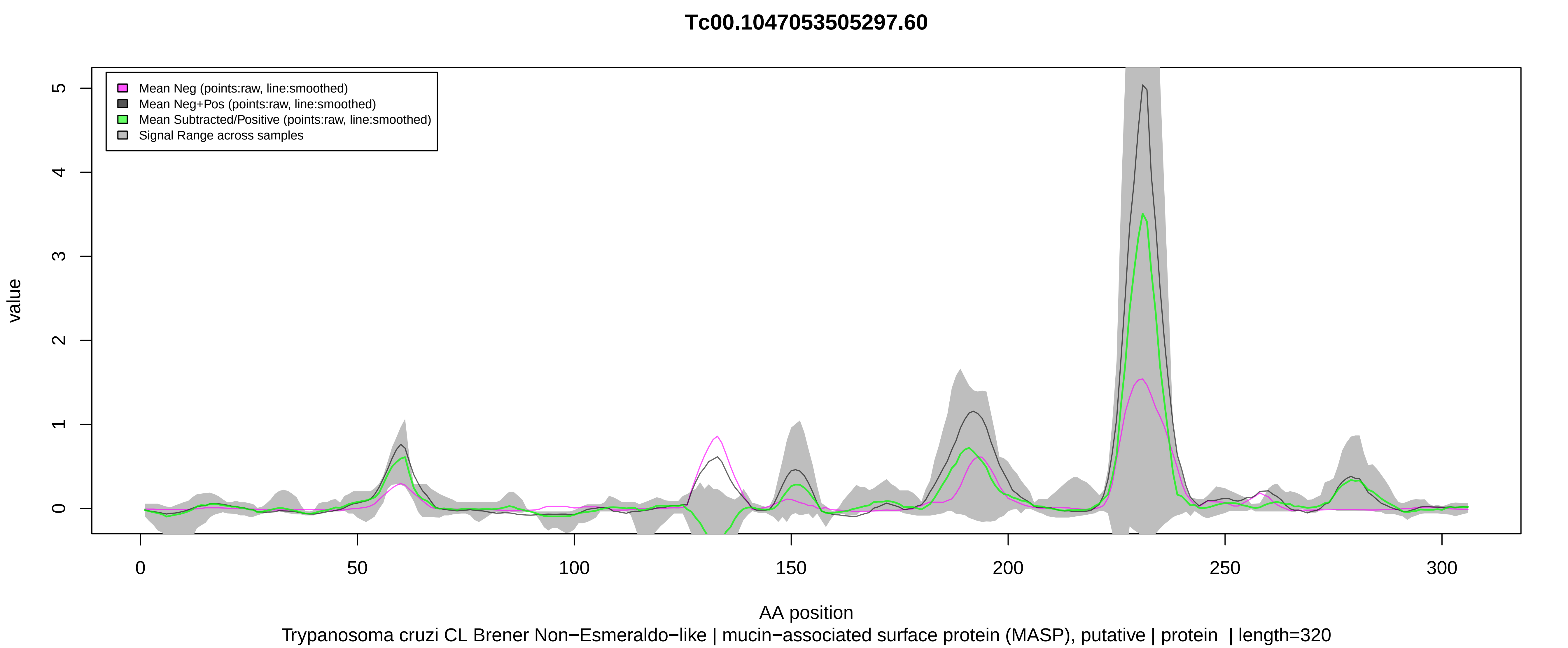

Tc00.1047053505297.60
Mean Neg (points:raw, line:smoothed)
Mean Neg+Pos (points:raw, line:smoothed)
Mean Subtracted/Positive (points:raw, line:smoothed)
Signal Range across samples
value
0
1
2
3
4
5
0
50
100
150
200
250
300
AA position
Trypanosoma cruzi CL Brener Non−Esmeraldo−like | mucin−associated surface protein (MASP), putative | protein | length=320

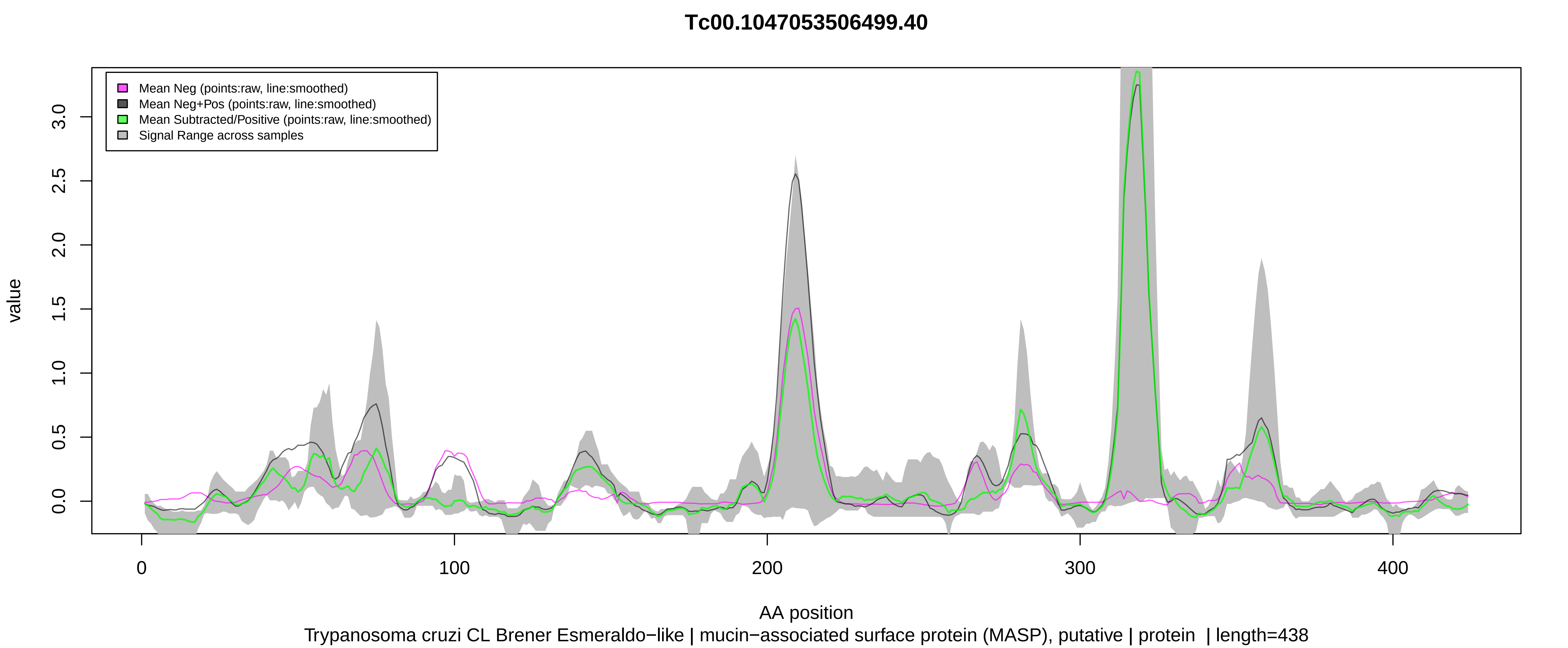

Tc00.1047053506499.40
Mean Neg (points:raw, line:smoothed)
Mean Neg+Pos (points:raw, line:smoothed)
Mean Subtracted/Positive (points:raw, line:smoothed)
Signal Range across samples
value
0.0
0.5
1.0
1.5
2.0
2.5
3.0
0
100
200
300
400
AA position
Trypanosoma cruzi CL Brener Esmeraldo−like | mucin−associated surface protein (MASP), putative | protein | length=438

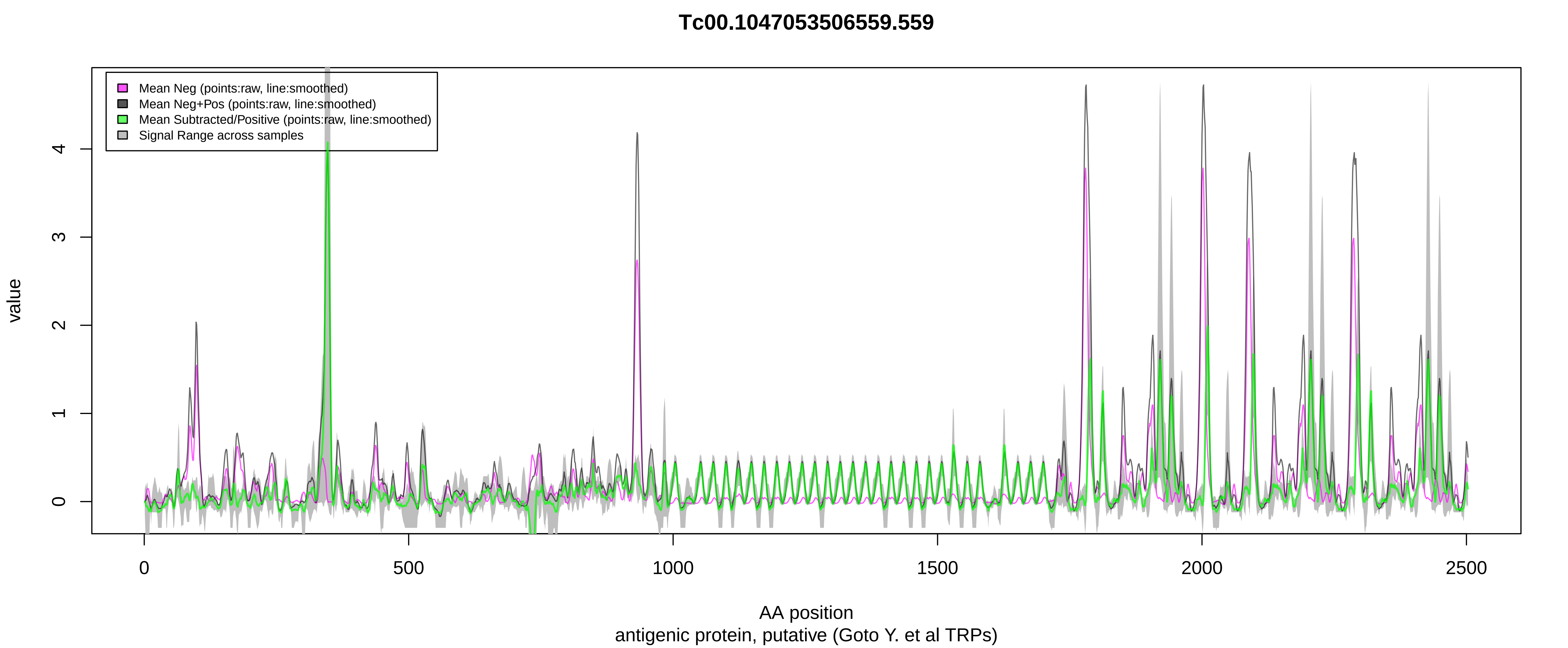

Tc00.1047053506559.559
Mean Neg (points:raw, line:smoothed)
Mean Neg+Pos (points:raw, line:smoothed)
Mean Subtracted/Positive (points:raw, line:smoothed)
Signal Range across samples
value
0
1
2
3
4
0
500
1000
1500
2000
2500
AA position
antigenic protein, putative (Goto Y. et al TRPs)

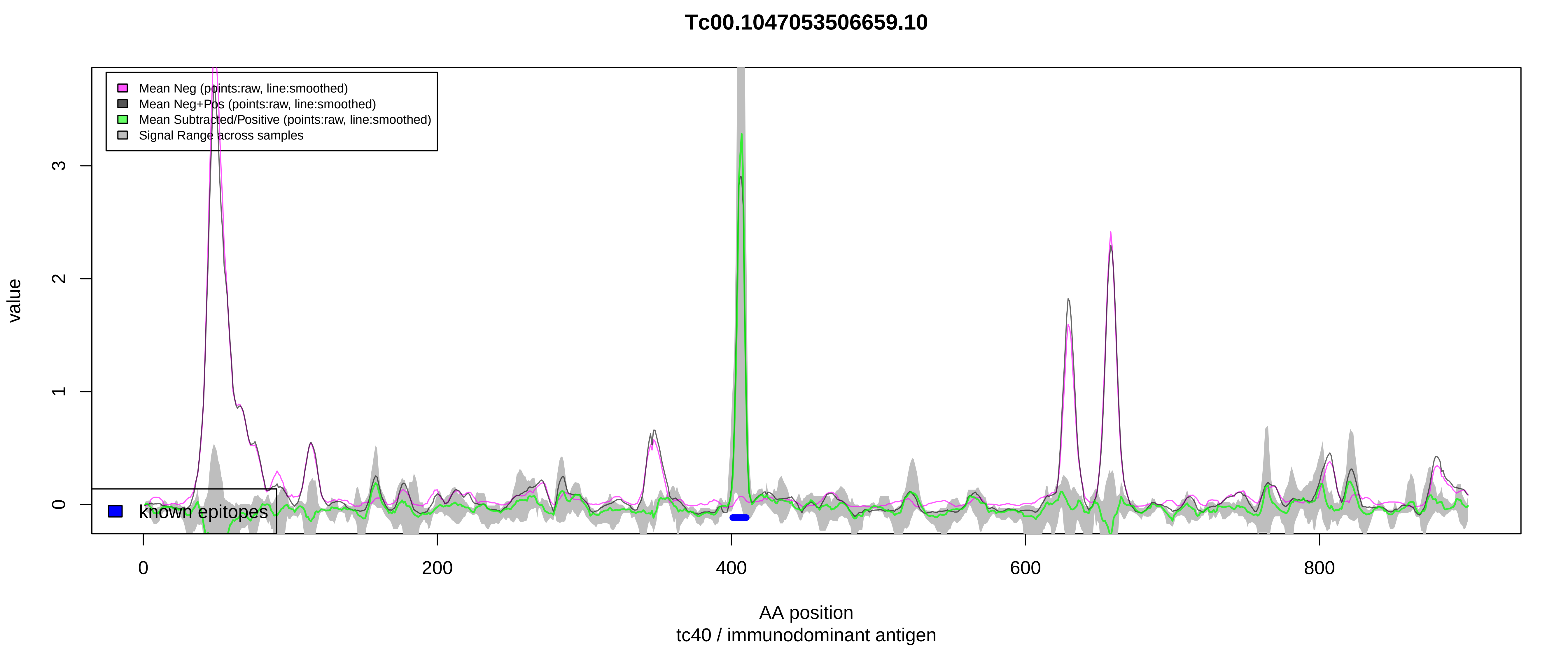

Tc00.1047053506659.10
Mean Neg (points:raw, line:smoothed)
Mean Neg+Pos (points:raw, line:smoothed)
Mean Subtracted/Positive (points:raw, line:smoothed)
Signal Range across samples
known epitopes
value
0
1
2
3
0
200
400
600
800
AA position
tc40 / immunodominant antigen

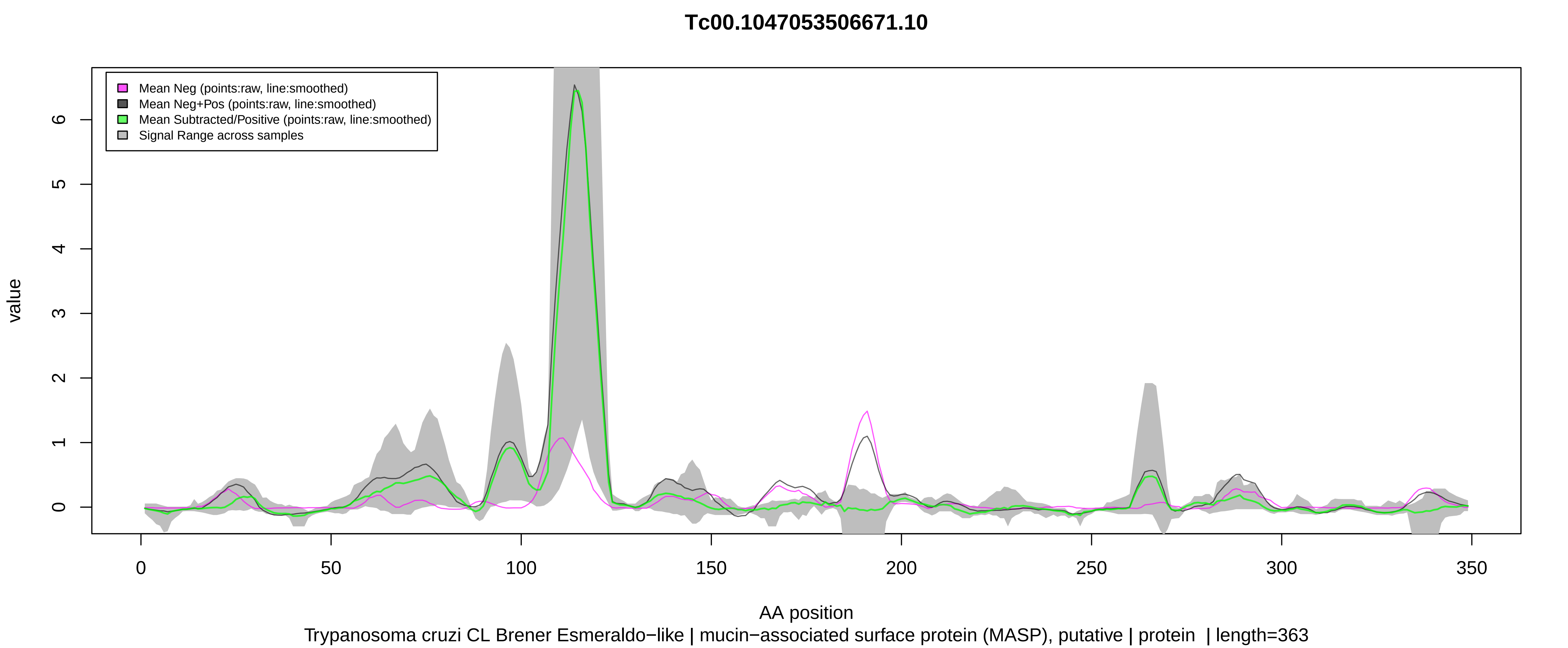

Tc00.1047053506671.10
Mean Neg (points:raw, line:smoothed)
Mean Neg+Pos (points:raw, line:smoothed)
Mean Subtracted/Positive (points:raw, line:smoothed)
Signal Range across samples
value
0
1
2
3
4
5
6
0
50
100
150
200
250
300
350
AA position
Trypanosoma cruzi CL Brener Esmeraldo−like | mucin−associated surface protein (MASP), putative | protein | length=363

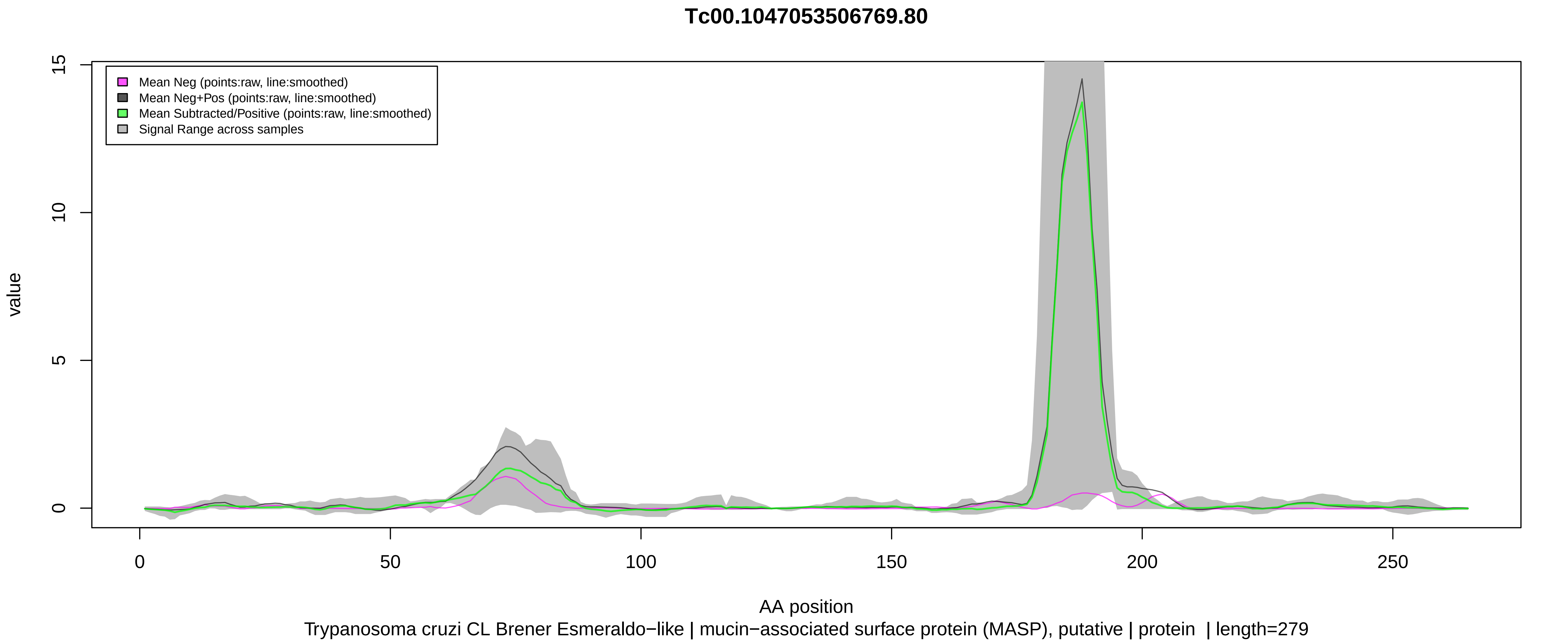

Tc00.1047053506769.80
Mean Neg (points:raw, line:smoothed)
Mean Neg+Pos (points:raw, line:smoothed)
Mean Subtracted/Positive (points:raw, line:smoothed)
Signal Range across samples
value
AA position
Trypanosoma cruzi CL Brener Esmeraldo−like | mucin−associated surface protein (MASP), putative | protein | length=279

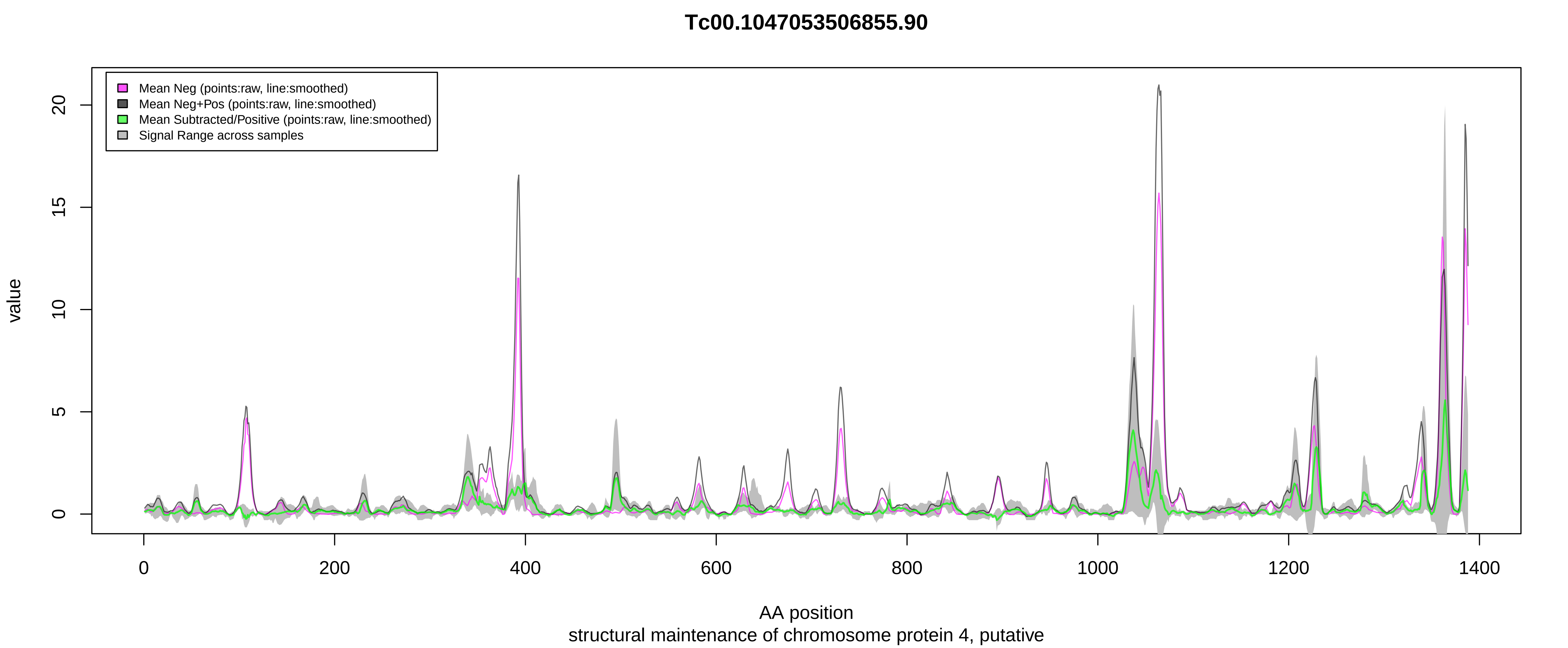

Tc00.1047053506855.90
Mean Neg (points:raw, line:smoothed)
Mean Neg+Pos (points:raw, line:smoothed)
Mean Subtracted/Positive (points:raw, line:smoothed)
Signal Range across samples
value
0
5
10
15
20
0
200
400
600
800
1000
1200
1400
AA position
structural maintenance of chromosome protein 4, putative

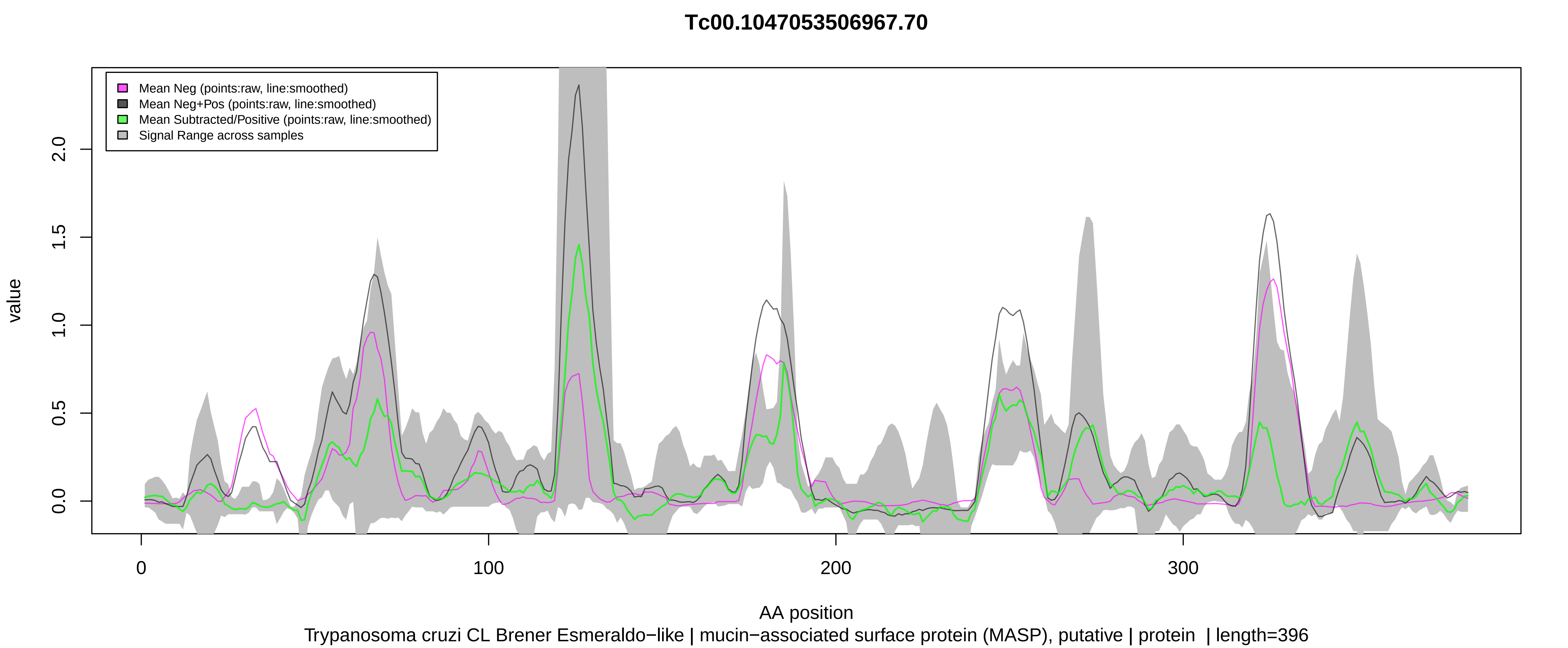
Tc00.1047053506967.70
Mean Neg (points:raw, line:smoothed)
Mean Neg+Pos (points:raw, line:smoothed)
Mean Subtracted/Positive (points:raw, line:smoothed)
Signal Range across samples
value
0.0
0.5
1.0
1.5
2.0
0
100
200
300
AA position
Trypanosoma cruzi CL Brener Esmeraldo−like | mucin−associated surface protein (MASP), putative | protein | length=396

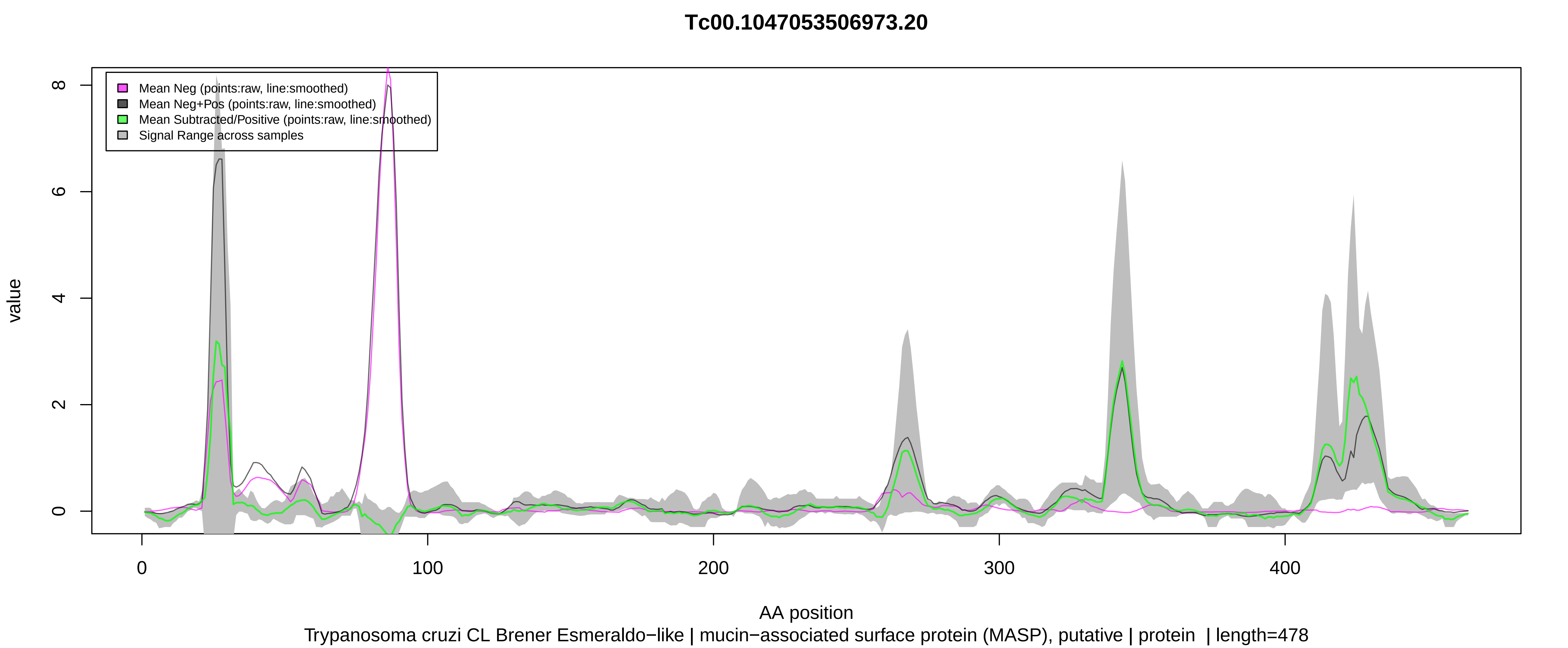
Tc00.1047053506973.20
Mean Neg (points:raw, line:smoothed)
Mean Neg+Pos (points:raw, line:smoothed)
Mean Subtracted/Positive (points:raw, line:smoothed)
Signal Range across samples
value
0
2
4
6
8
0
100
200
300
400
AA position
Trypanosoma cruzi CL Brener Esmeraldo−like | mucin−associated surface protein (MASP), putative | protein | length=478

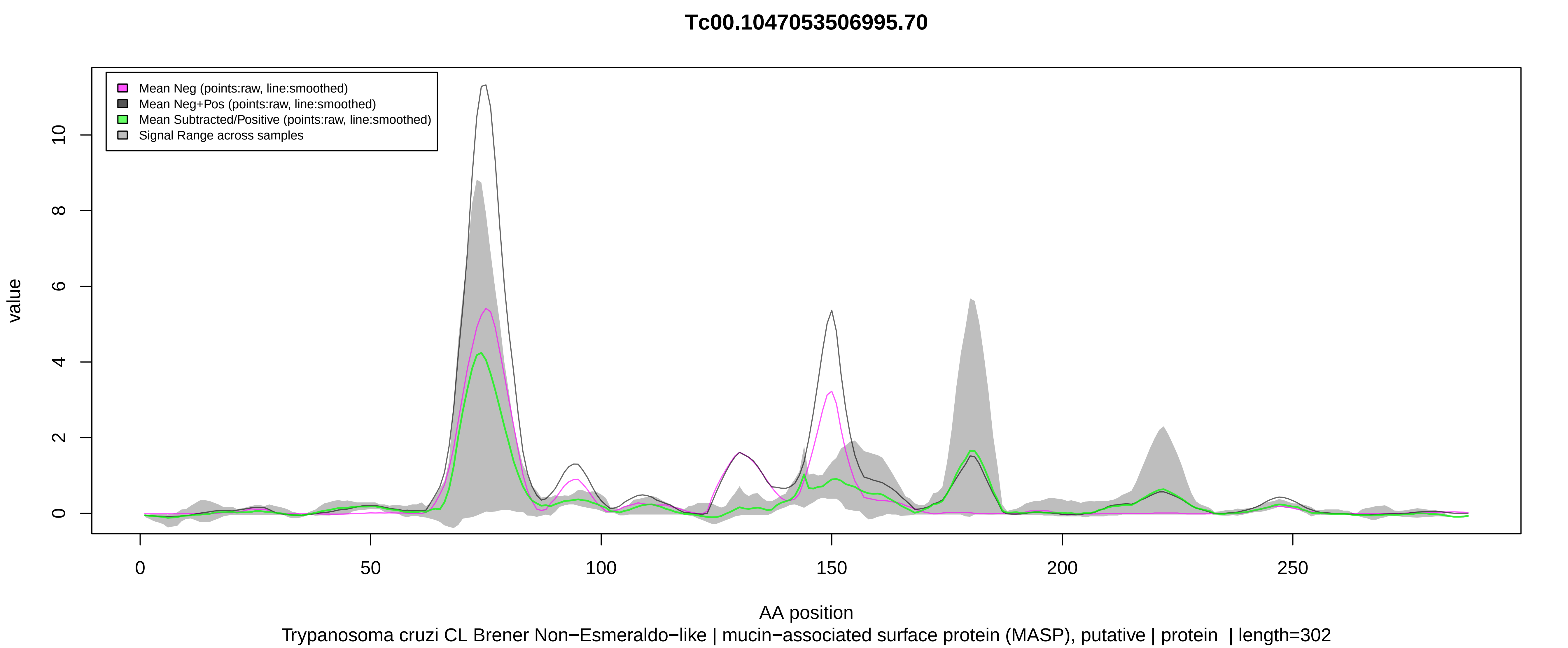

Tc00.1047053506995.70
Mean Neg (points:raw, line:smoothed)
Mean Neg+Pos (points:raw, line:smoothed)
Mean Subtracted/Positive (points:raw, line:smoothed)
Signal Range across samples
value
0
2
4
6
8
10
0
50
100
150
200
250
AA position
Trypanosoma cruzi CL Brener Non−Esmeraldo−like | mucin−associated surface protein (MASP), putative | protein | length=302

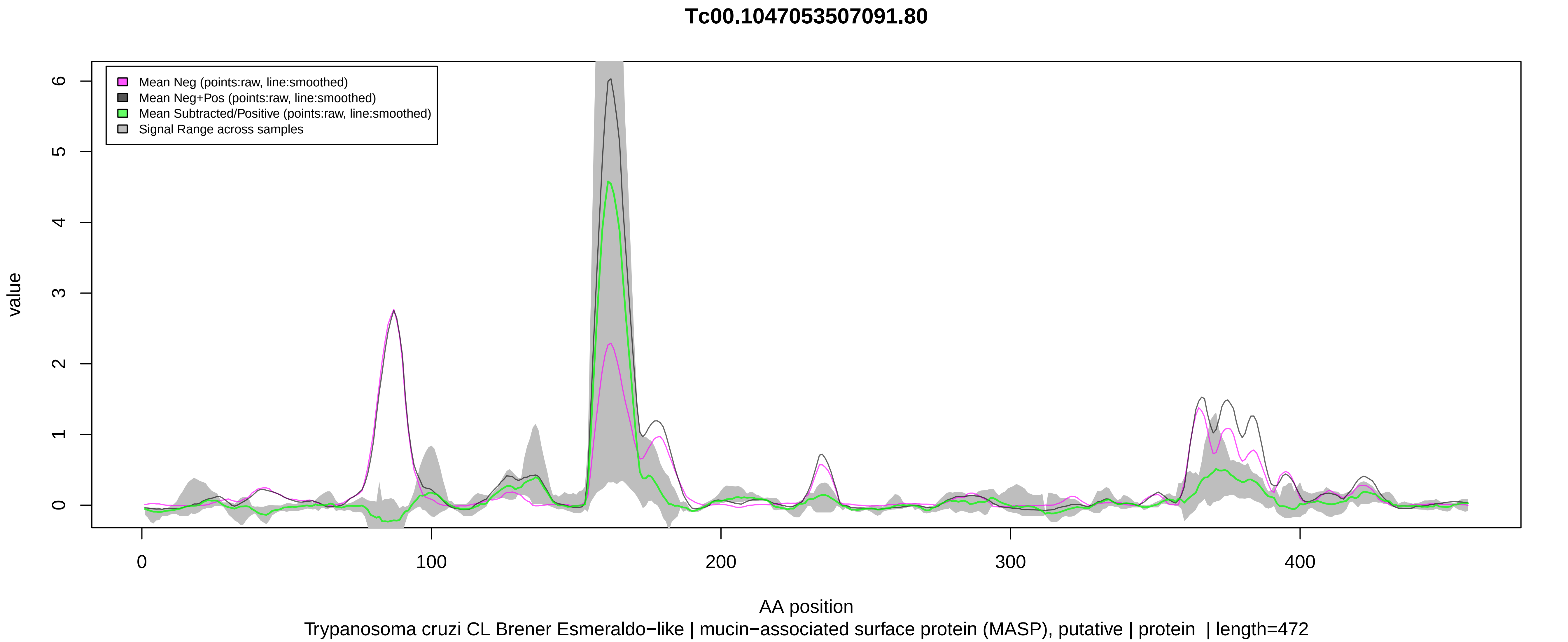
Tc00.1047053507091.80
Mean Neg (points:raw, line:smoothed)
Mean Neg+Pos (points:raw, line:smoothed)
Mean Subtracted/Positive (points:raw, line:smoothed)
Signal Range across samples
value
0
1
2
3
4
5
6
0
100
200
300
400
AA position
Trypanosoma cruzi CL Brener Esmeraldo−like | mucin−associated surface protein (MASP), putative | protein | length=472

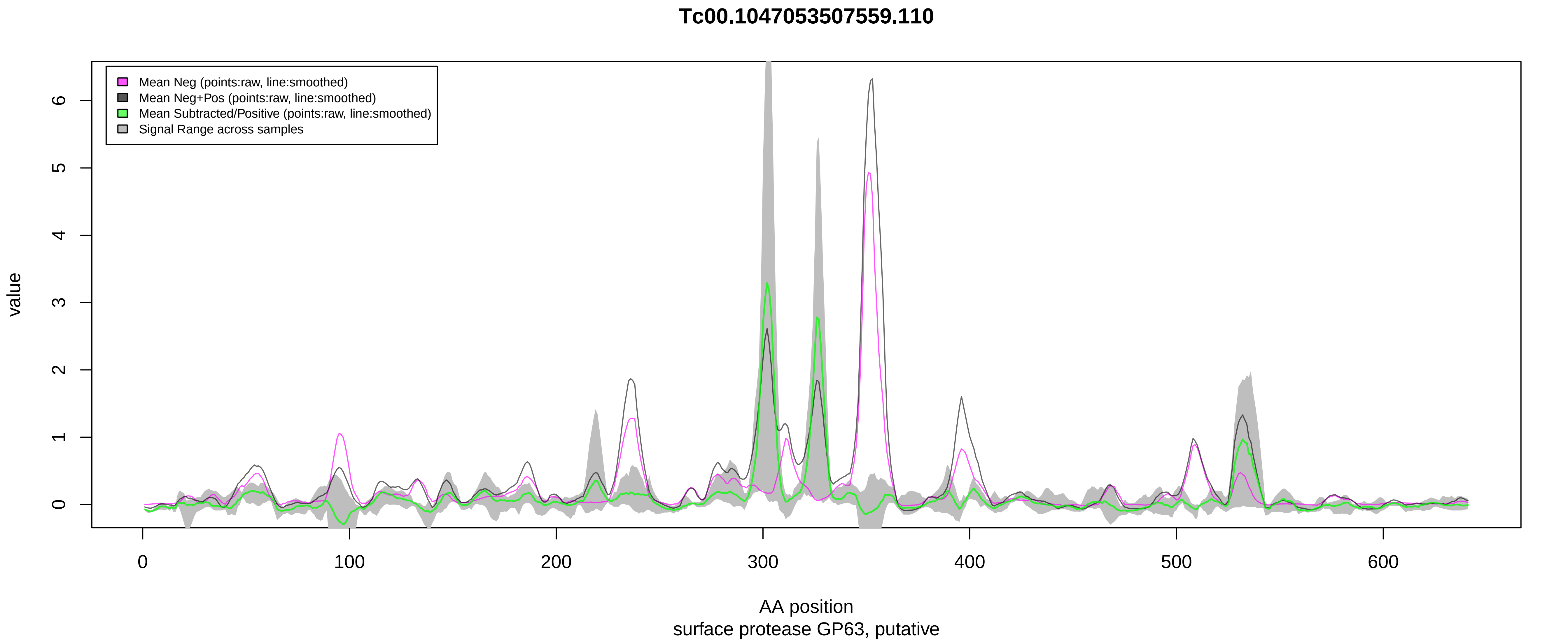
Tc00.1047053507559.110
Mean Neg (points:raw, line:smoothed)
Mean Neg+Pos (points:raw, line:smoothed)
Mean Subtracted/Positive (points:raw, line:smoothed)
Signal Range across samples
value
0
1
2
3
4
5
6
0
100
200
300
400
500
600
AA position
surface protease GP63, putative

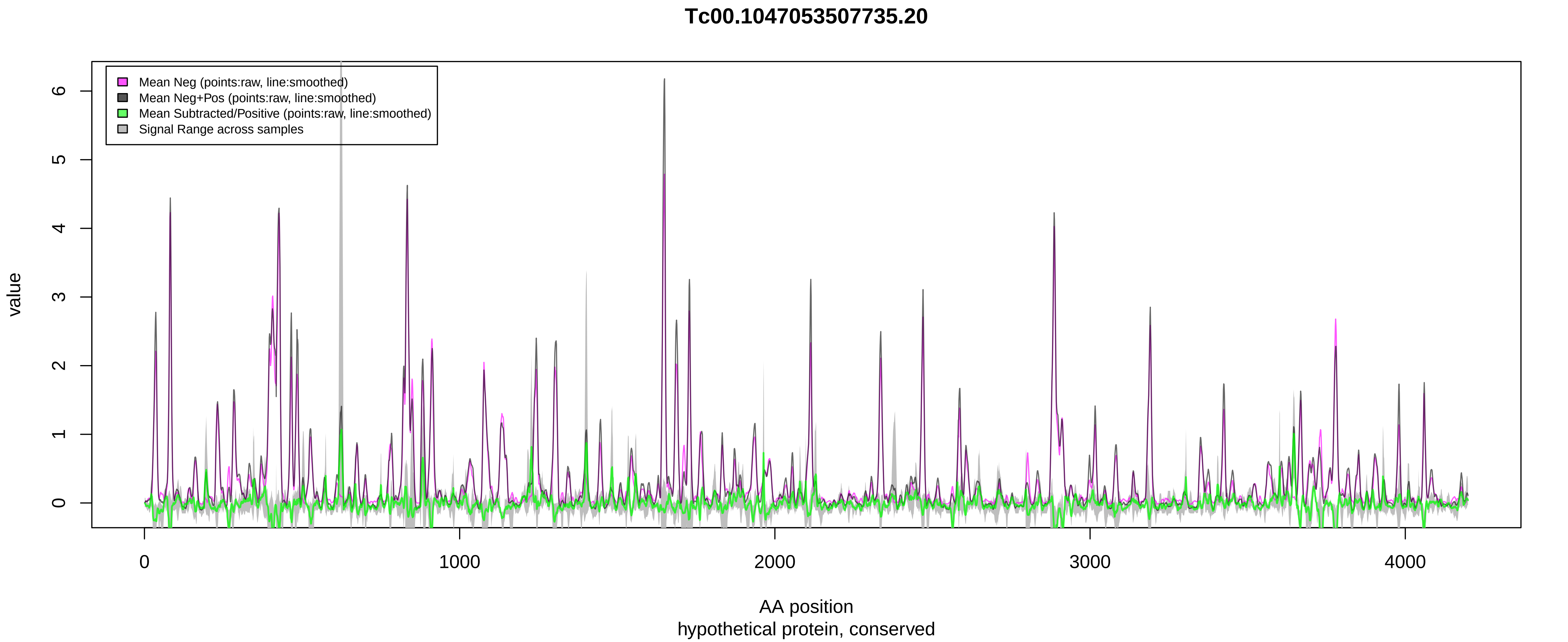

Tc00.1047053507735.20
Mean Neg (points:raw, line:smoothed)
Mean Neg+Pos (points:raw, line:smoothed)
Mean Subtracted/Positive (points:raw, line:smoothed)
Signal Range across samples
value
0
1
2
3
4
5
6
0
1000
2000
3000
4000
AA position
hypothetical protein, conserved

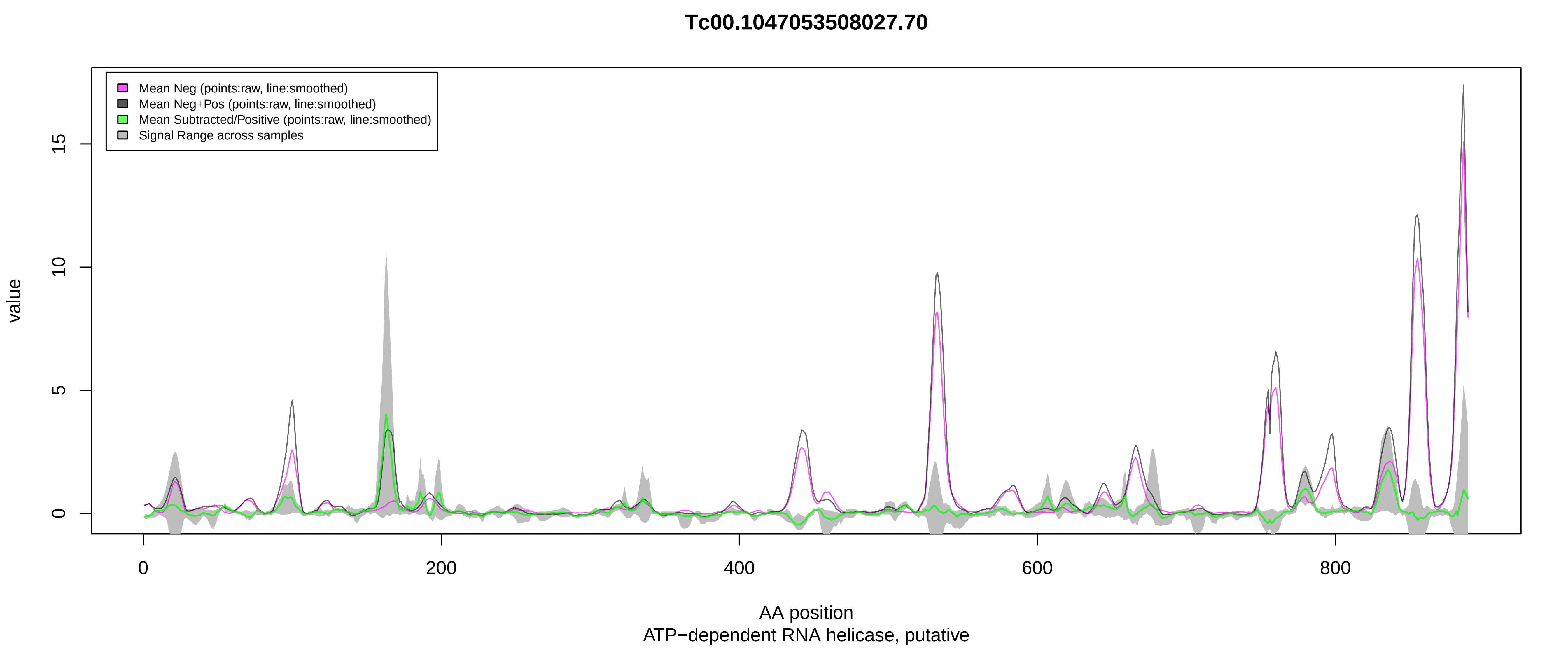

Tc00.1047053508027.70
Mean Neg (points:raw, line:smoothed)
Mean Neg+Pos (points:raw, line:smoothed)
Mean Subtracted/Positive (points:raw, line:smoothed)
Signal Range across samples
value
0
5
10
15
0
200
400
600
800
AA position
ATP−dependent RNA helicase, putative

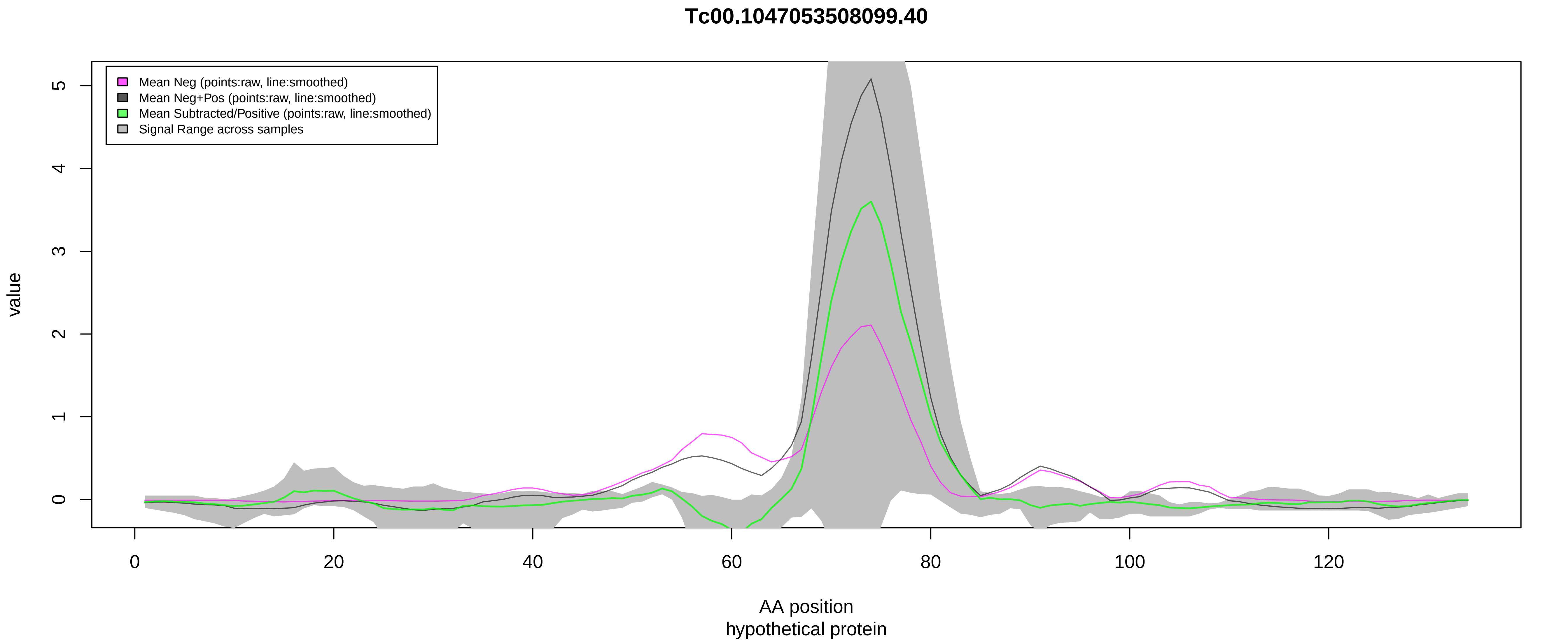
Tc00.1047053508099.40
Mean Neg (points:raw, line:smoothed)
Mean Neg+Pos (points:raw, line:smoothed)
Mean Subtracted/Positive (points:raw, line:smoothed)
Signal Range across samples
value
0
1
2
3
4
5
0
20
40
60
80
100
120
AA position
hypothetical protein

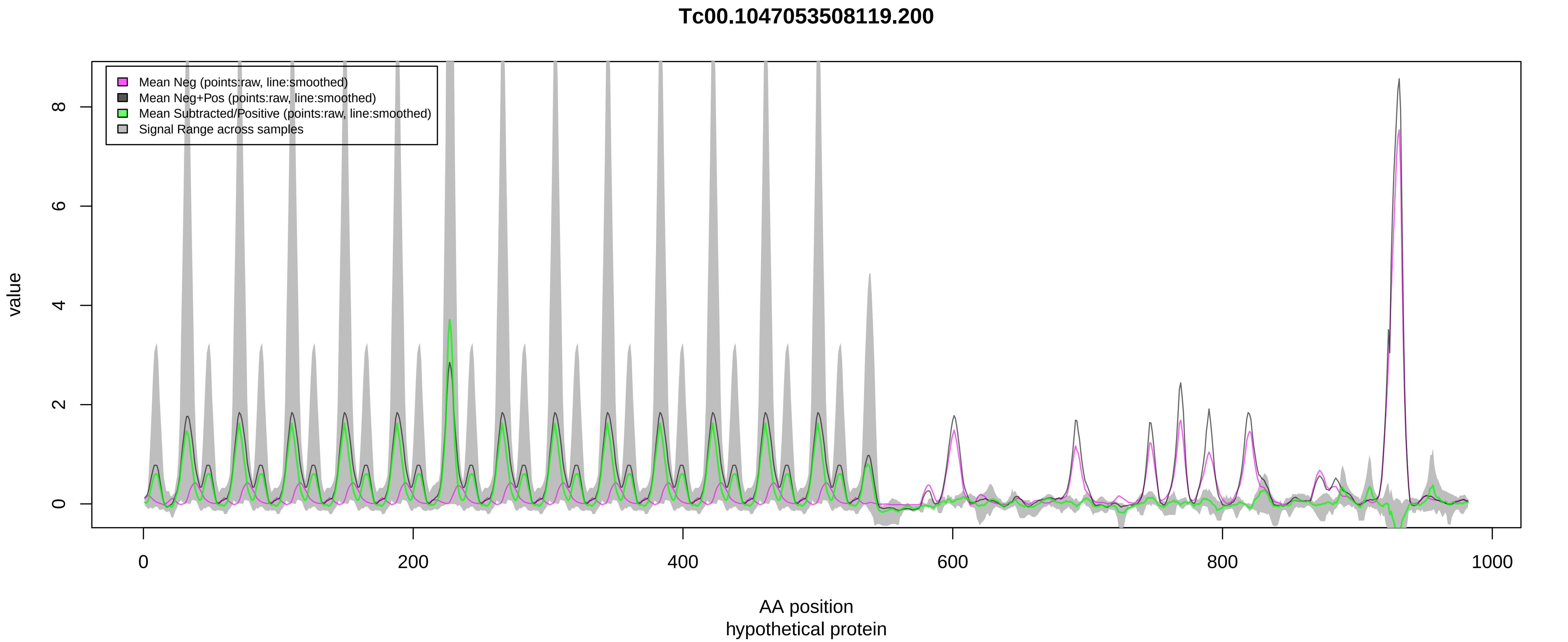
Tc00.1047053508119.200
Mean Neg (points:raw, line:smoothed)
Mean Neg+Pos (points:raw, line:smoothed)
Mean Subtracted/Positive (points:raw, line:smoothed)
Signal Range across samples
value
0
2
4
6
8
0
200
400
600
800
1000
AA position
hypothetical protein

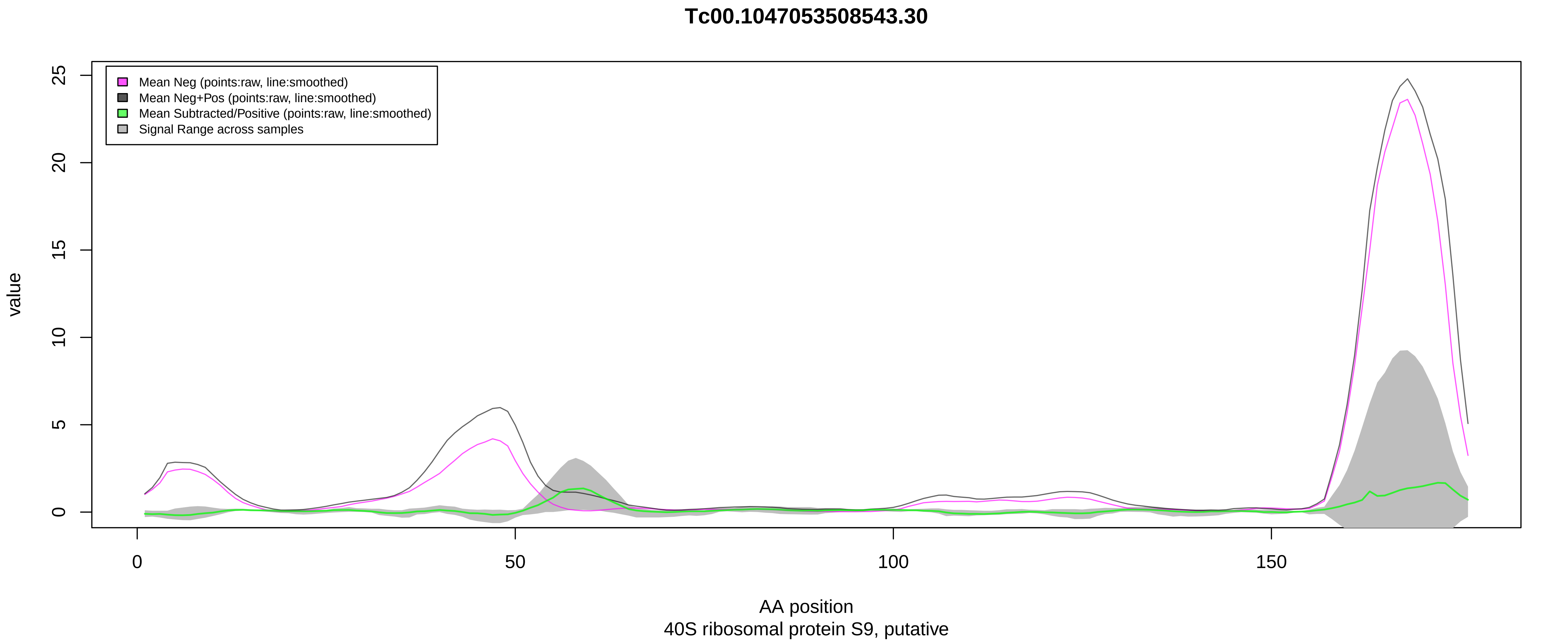

Tc00.1047053508543.30
Mean Neg (points:raw, line:smoothed)
Mean Neg+Pos (points:raw, line:smoothed)
Mean Subtracted/Positive (points:raw, line:smoothed)
Signal Range across samples
value
0
5
10
15
20
25
0
50
100
150
AA position
40S ribosomal protein S9, putative

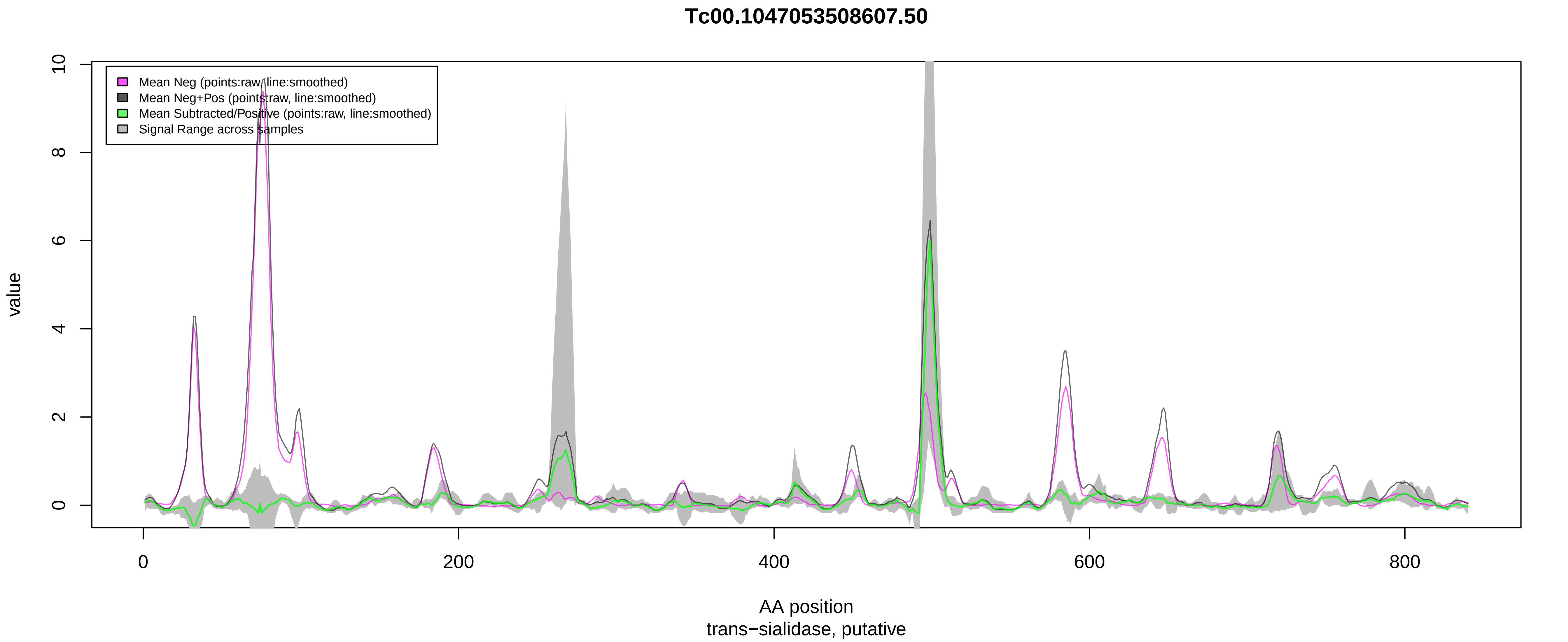
Tc00.1047053508607.50
Mean Neg (points:raw, line:smoothed)
Mean Neg+Pos (points:raw, line:smoothed)
Mean Subtracted/Positive (points:raw, line:smoothed)
Signal Range across samples
value
0
2
4
6
8
10
0
200
400
600
800
AA position
trans−sialidase, putative

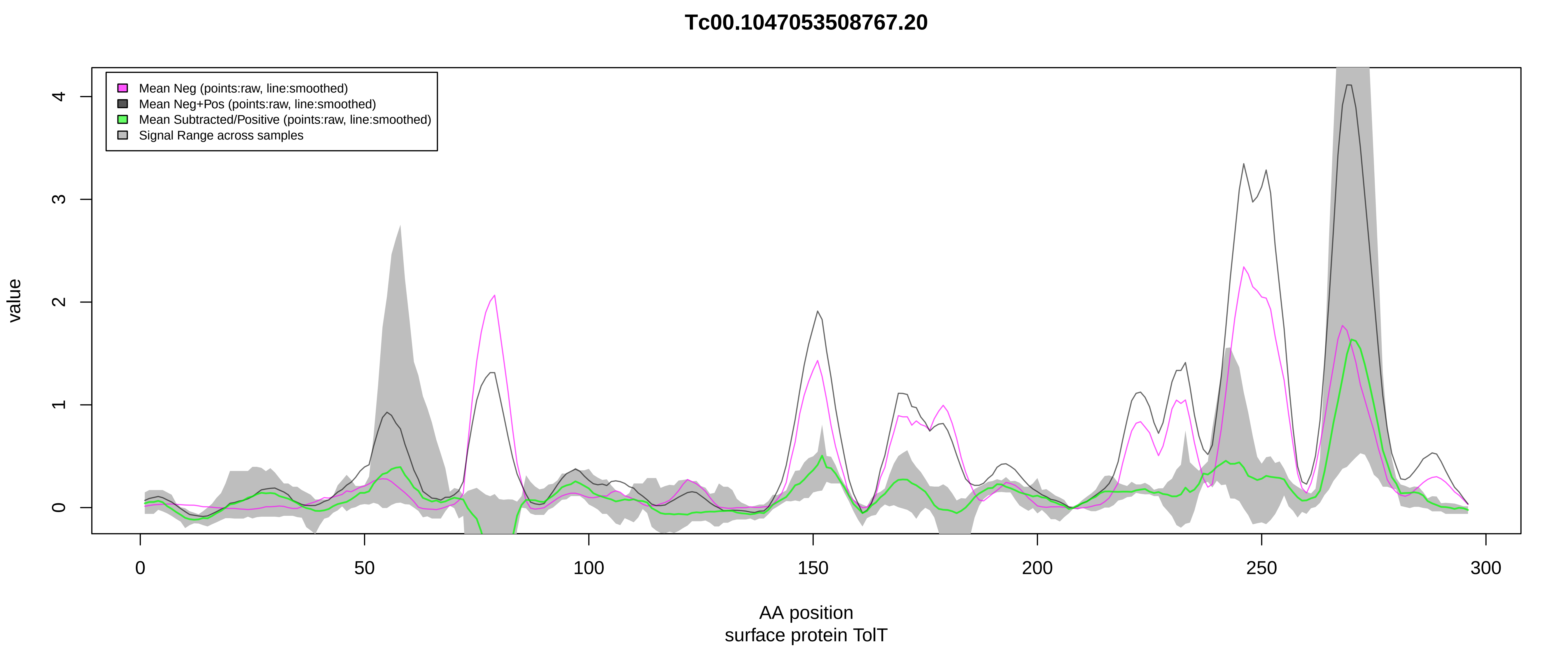

Tc00.1047053508767.20
Mean Neg (points:raw, line:smoothed)
Mean Neg+Pos (points:raw, line:smoothed)
Mean Subtracted/Positive (points:raw, line:smoothed)
Signal Range across samples
value
0
1
2
3
4
0
50
100
150
200
250
300
AA position
surface protein TolT

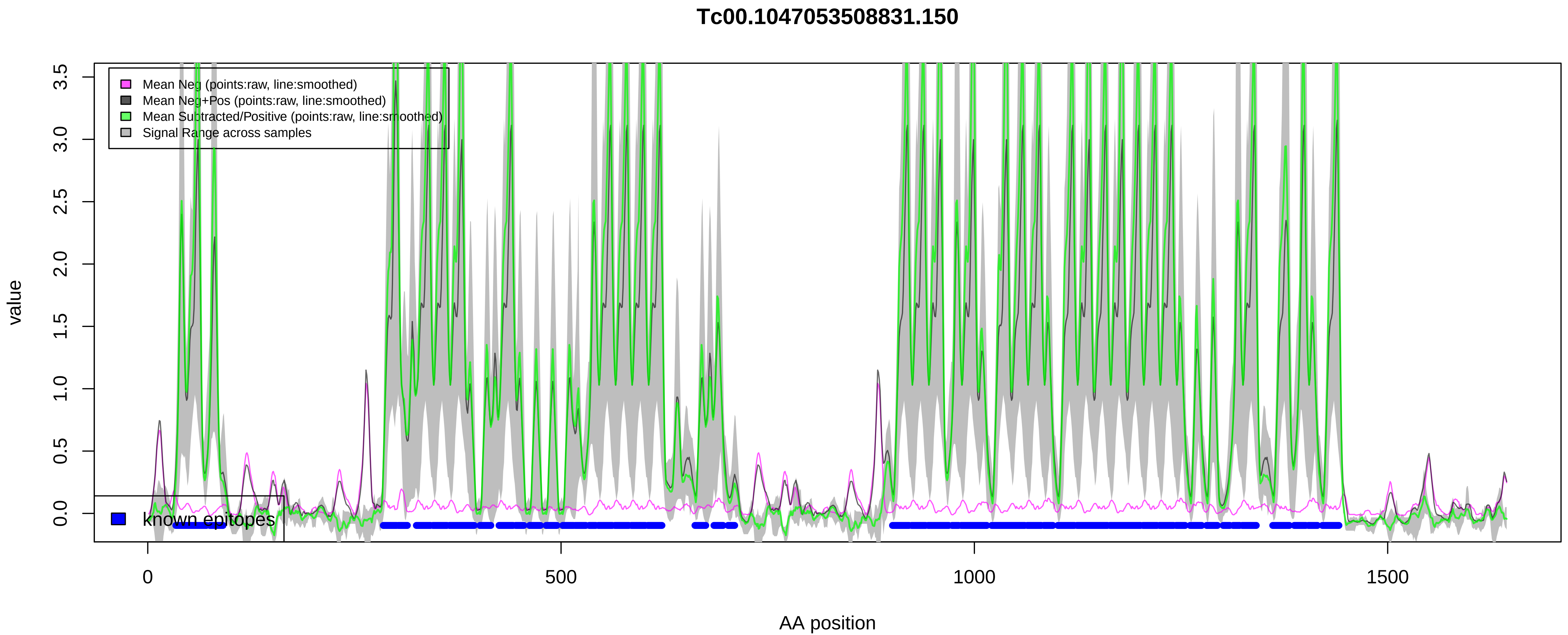

hypothetical protein, antigenic protein n86 (Carmona SJ et al 2012)

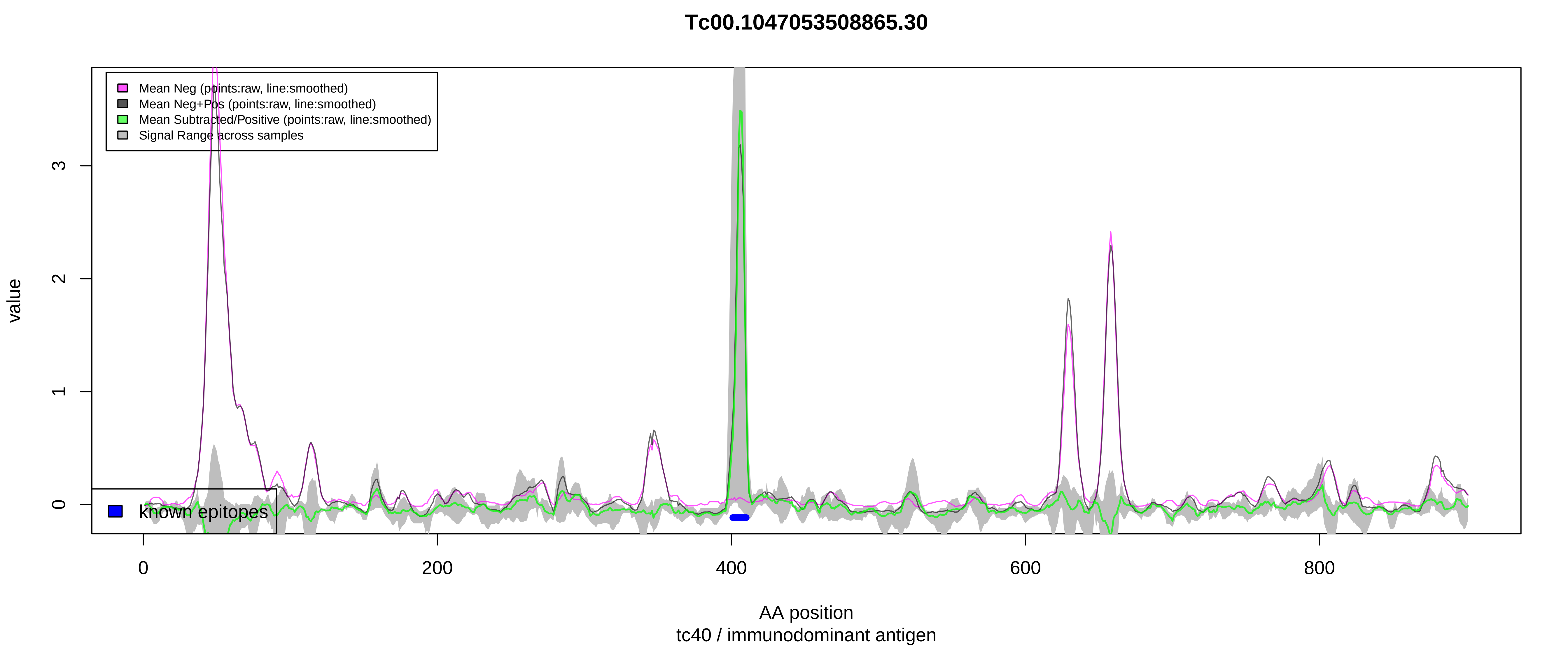
Tc00.1047053508865.30
Mean Neg (points:raw, line:smoothed)
Mean Neg+Pos (points:raw, line:smoothed)
Mean Subtracted/Positive (points:raw, line:smoothed)
Signal Range across samples
known epitopes
value
0
1
2
3
0
200
400
600
800
AA position
tc40 / immunodominant antigen

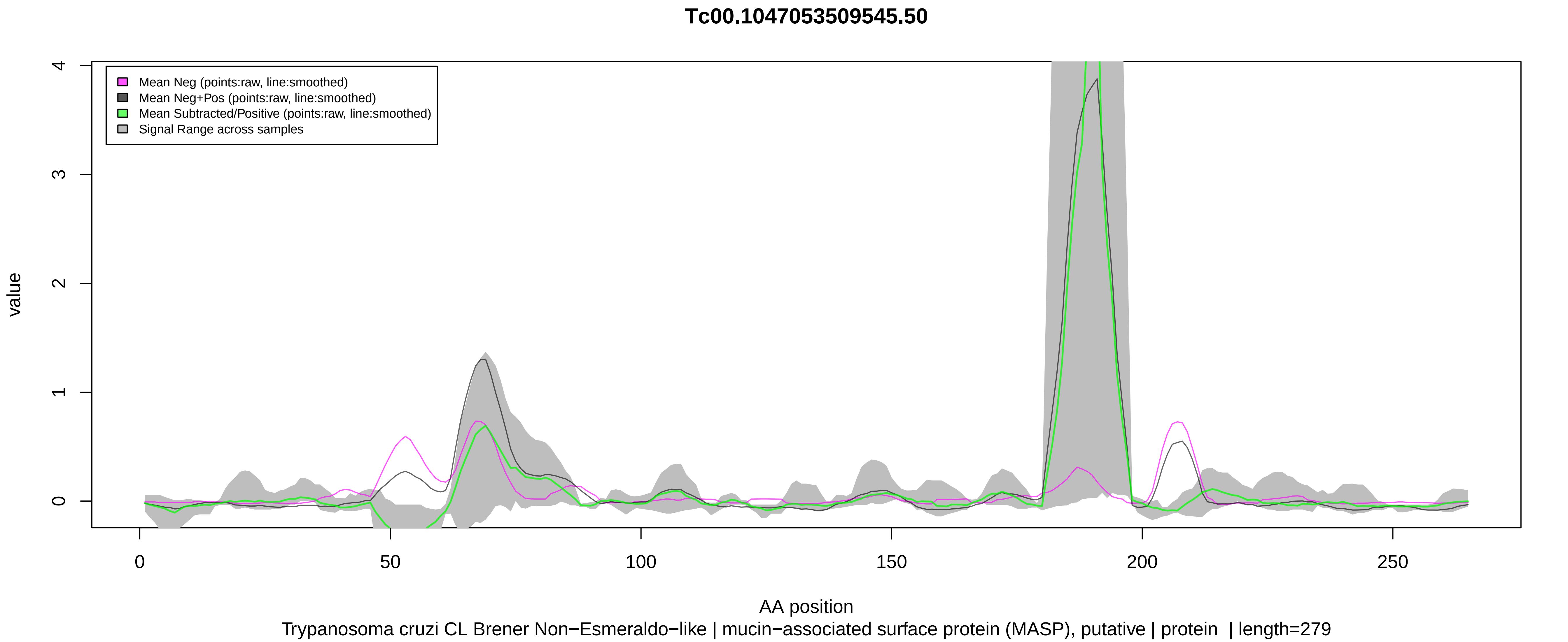

Tc00.1047053509545.50
Mean Neg (points:raw, line:smoothed)
Mean Neg+Pos (points:raw, line:smoothed)
Mean Subtracted/Positive (points:raw, line:smoothed)
Signal Range across samples
value
0
1
2
3
4
0
50
100
150
200
250
AA position
Trypanosoma cruzi CL Brener Non−Esmeraldo−like | mucin−associated surface protein (MASP), putative | protein | length=279

Tc00.1047053509755.40

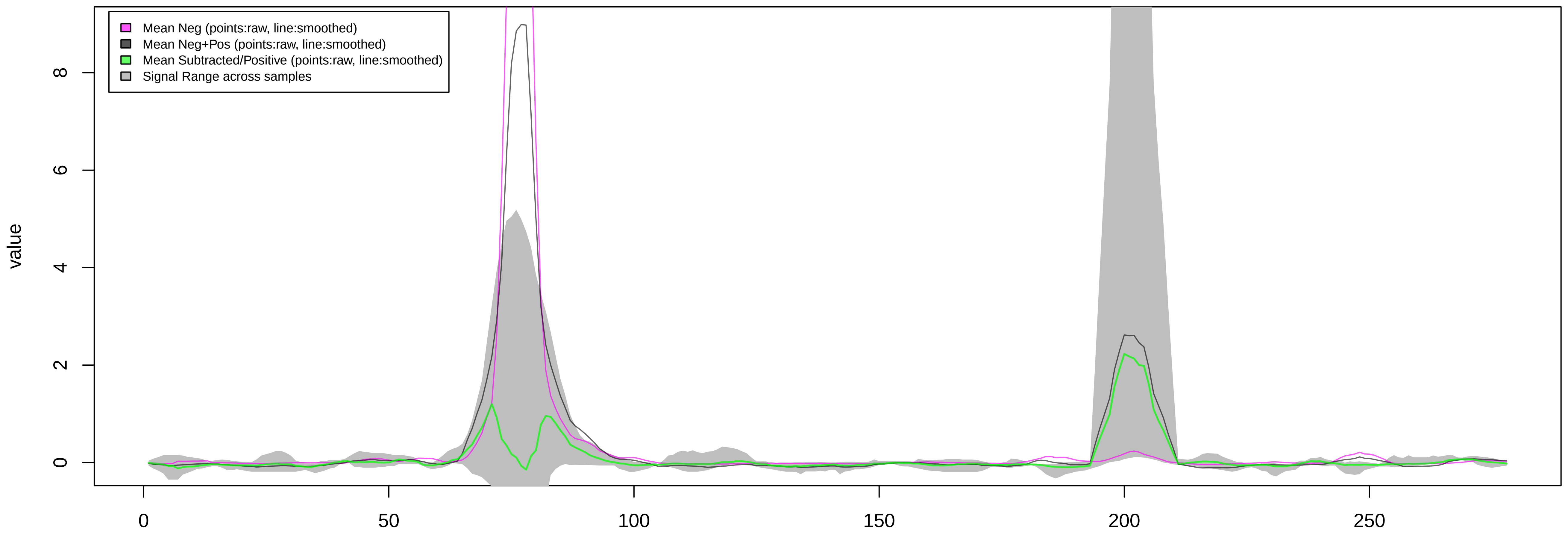

Trypanosoma cruzi CL Brener Non−Esmeraldo−like | mucin−associated surface protein (MASP), putative | protein | length=292

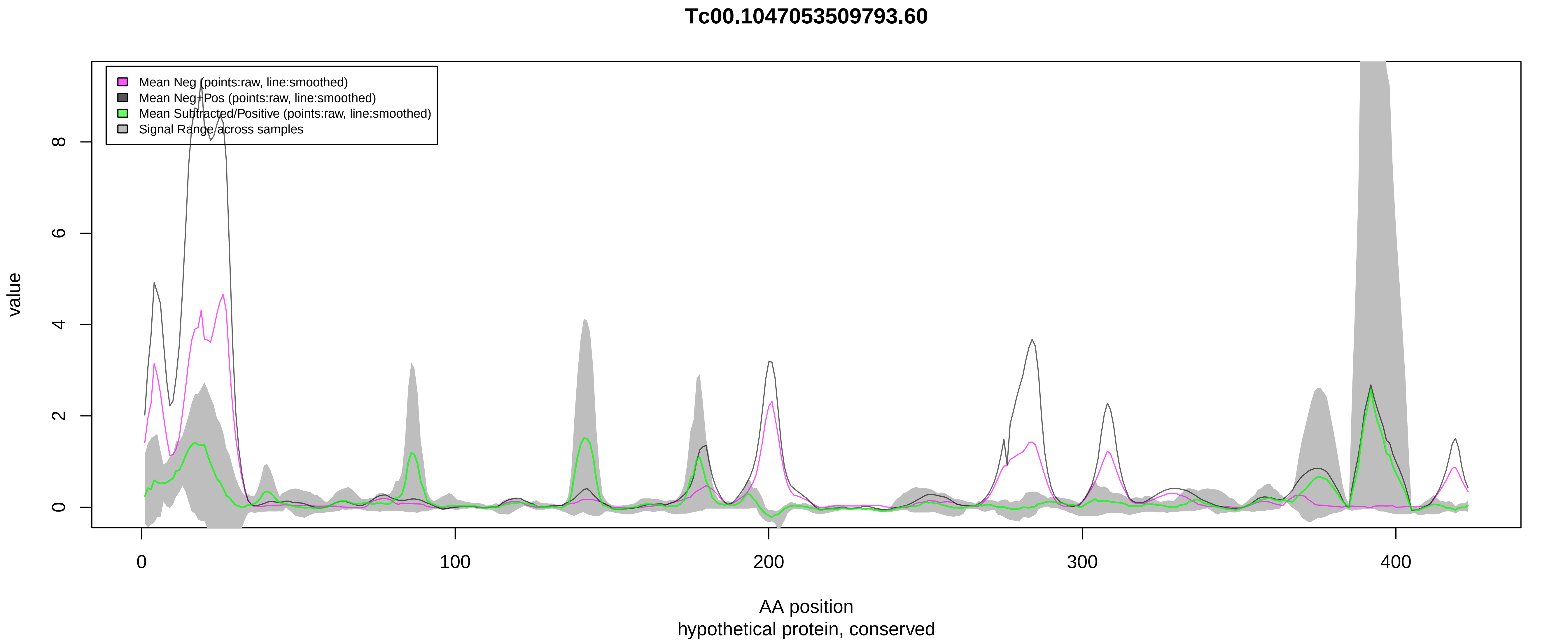

Tc00.1047053509793.60
Mean Neg (points:raw, line:smoothed)
Mean Neg+Pos (points:raw, line:smoothed)
Mean Subtracted/Positive (points:raw, line:smoothed)
Signal Range across samples
value
0
2
4
6
8
0
100
200
300
400
AA position
hypothetical protein, conserved

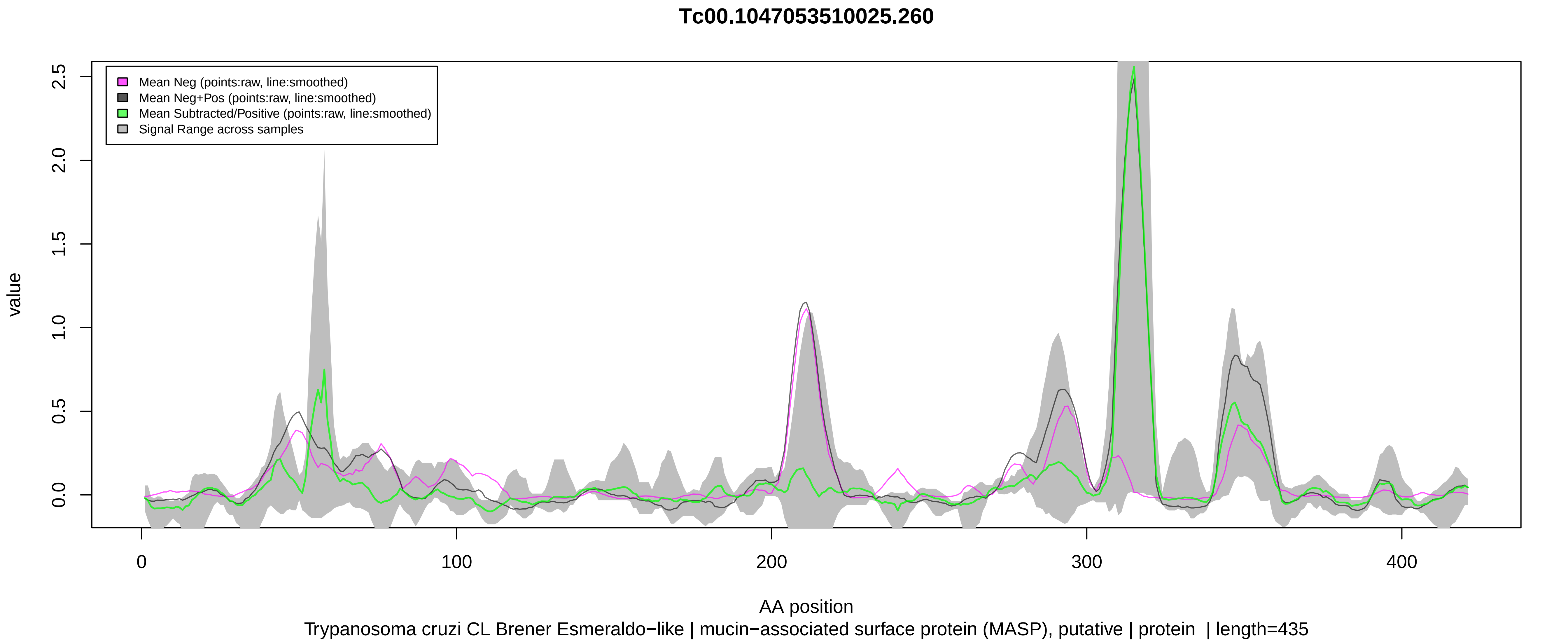
Tc00.1047053510025.260
Mean Neg (points:raw, line:smoothed)
Mean Neg+Pos (points:raw, line:smoothed)
Mean Subtracted/Positive (points:raw, line:smoothed)
Signal Range across samples
value
0.0
0.5
1.0
1.5
2.0
2.5
0
100
200
300
400
AA position
Trypanosoma cruzi CL Brener Esmeraldo−like | mucin−associated surface protein (MASP), putative | protein | length=435

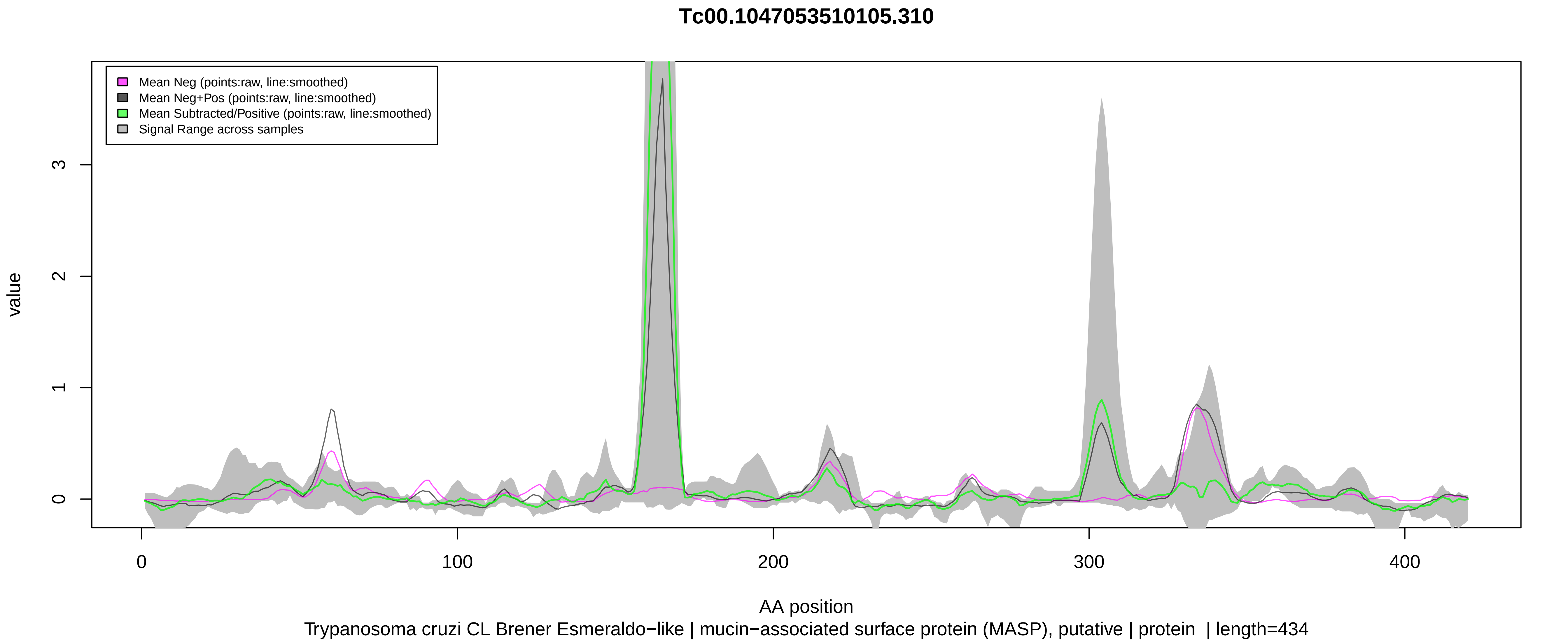

Tc00.1047053510105.310
Mean Neg (points:raw, line:smoothed)
Mean Neg+Pos (points:raw, line:smoothed)
Mean Subtracted/Positive (points:raw, line:smoothed)
Signal Range across samples
value
0
1
2
3
0
100
200
300
400
AA position
Trypanosoma cruzi CL Brener Esmeraldo−like | mucin−associated surface protein (MASP), putative | protein | length=434

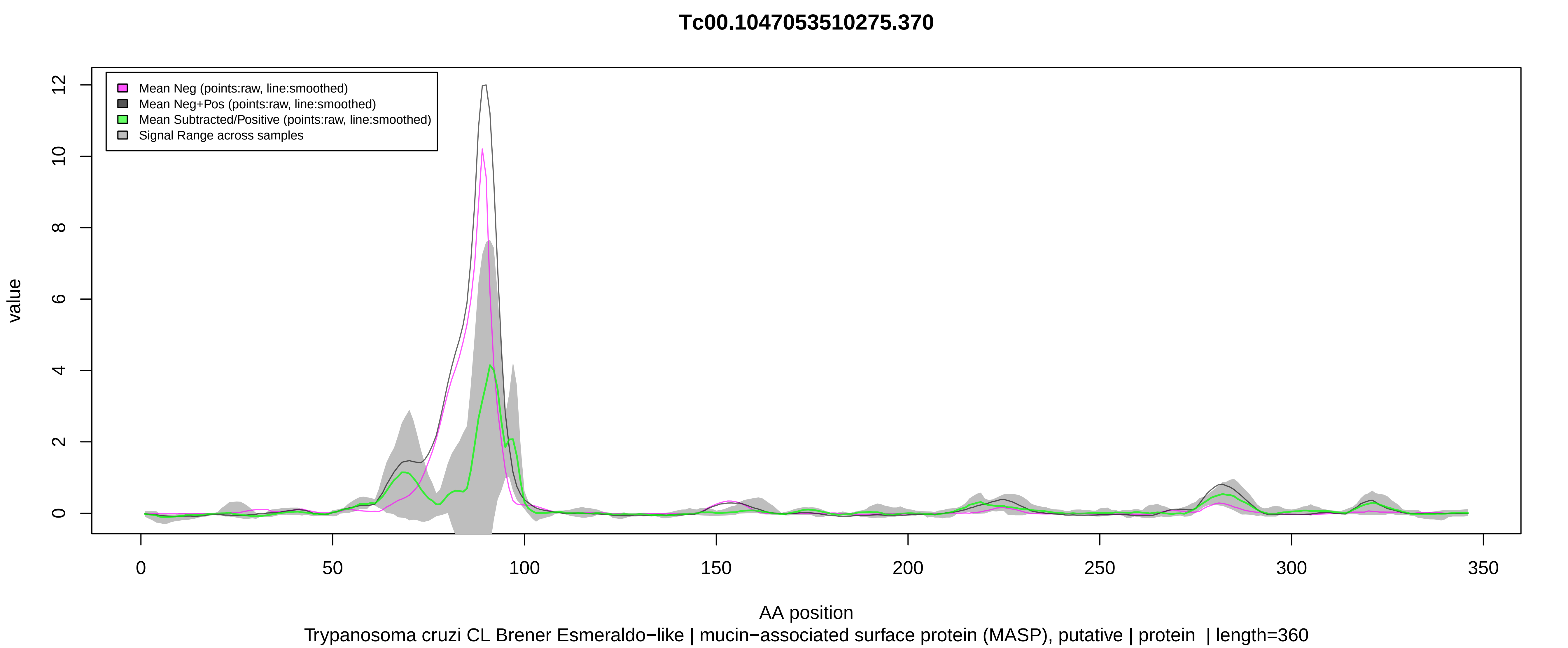

Tc00.1047053510275.370
Mean Neg (points:raw, line:smoothed)
Mean Neg+Pos (points:raw, line:smoothed)
Mean Subtracted/Positive (points:raw, line:smoothed)
Signal Range across samples
value
0
2
4
6
8
10
12
0
50
100
150
200
250
300
350
AA position
Trypanosoma cruzi CL Brener Esmeraldo−like | mucin−associated surface protein (MASP), putative | protein | length=360

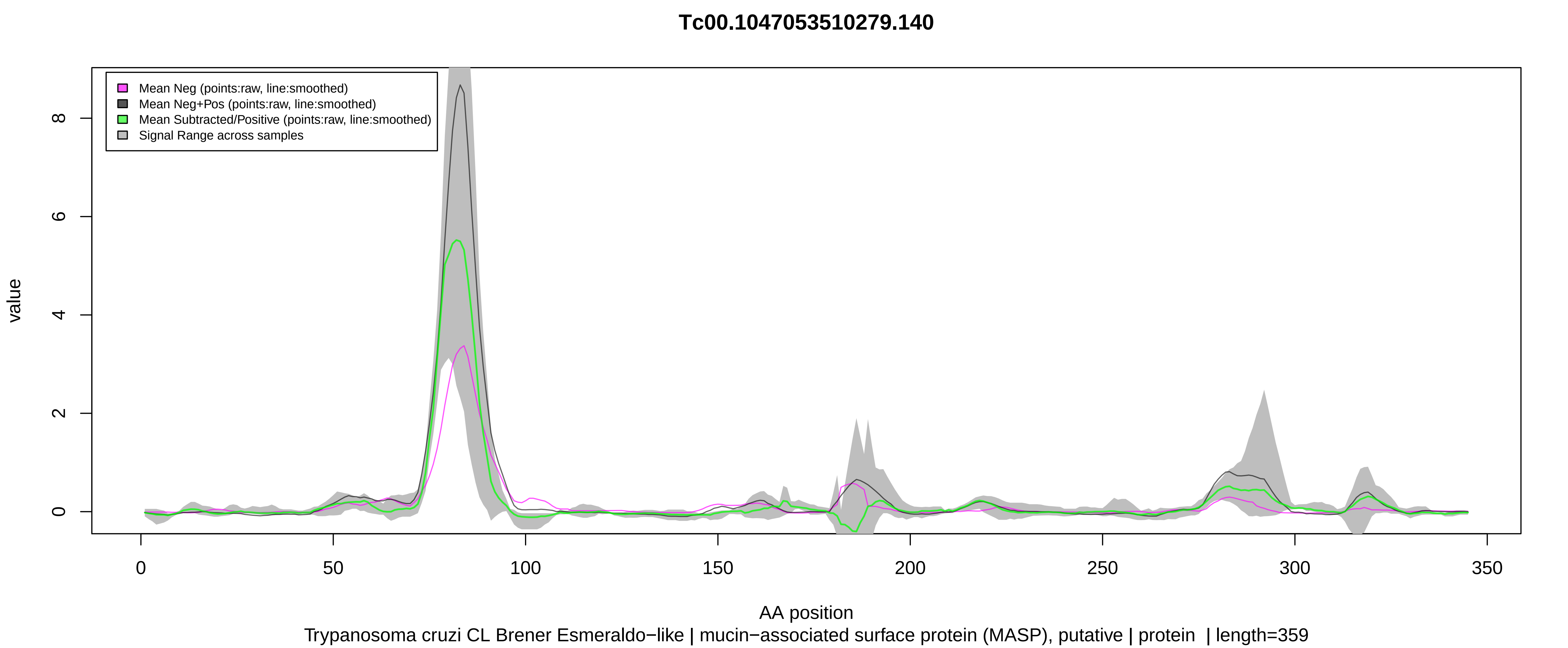
Tc00.1047053510279.140
Mean Neg (points:raw, line:smoothed)
Mean Neg+Pos (points:raw, line:smoothed)
Mean Subtracted/Positive (points:raw, line:smoothed)
Signal Range across samples
value
0
2
4
6
8
0
50
100
150
200
250
300
350
AA position
Trypanosoma cruzi CL Brener Esmeraldo−like | mucin−associated surface protein (MASP), putative | protein | length=359

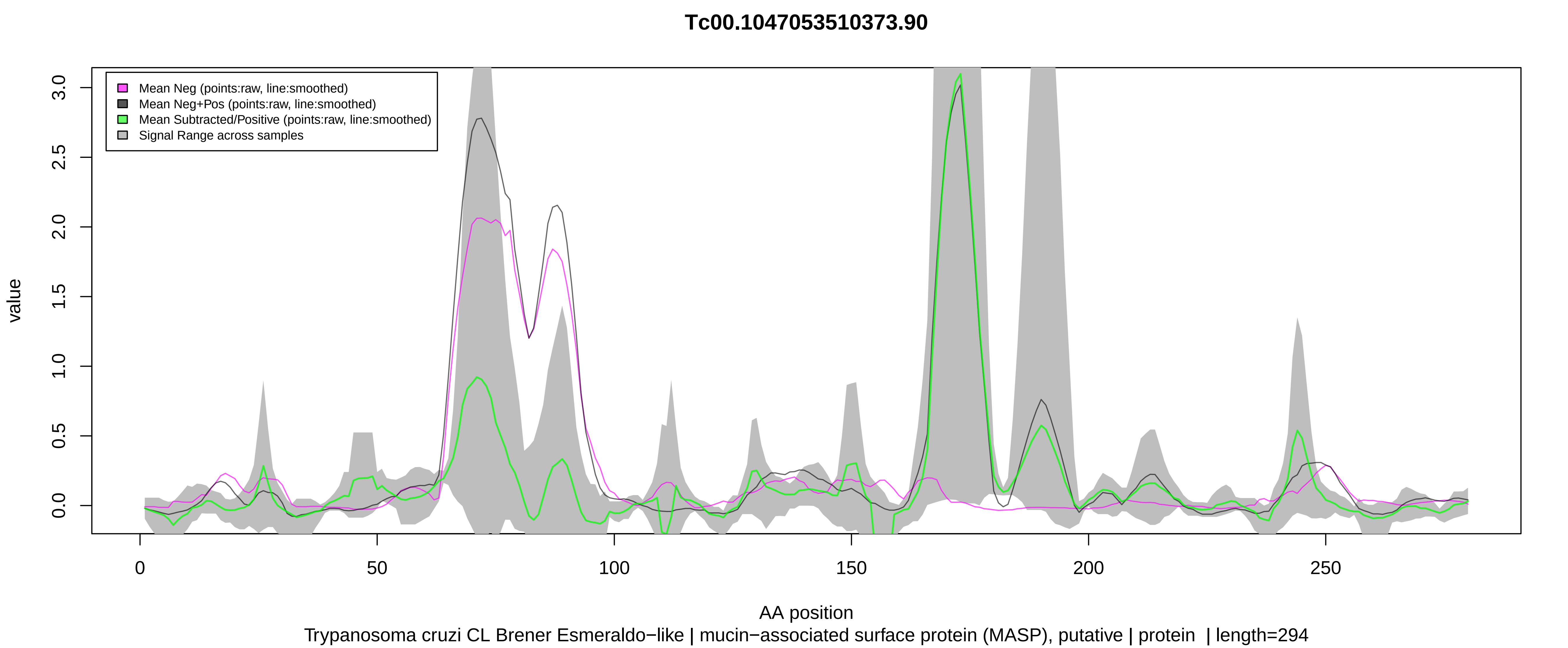
Tc00.1047053510373.90
Mean Neg (points:raw, line:smoothed)
Mean Neg+Pos (points:raw, line:smoothed)
Mean Subtracted/Positive (points:raw, line:smoothed)
Signal Range across samples
value
AA position
Trypanosoma cruzi CL Brener Esmeraldo−like | mucin−associated surface protein (MASP), putative | protein | length=294

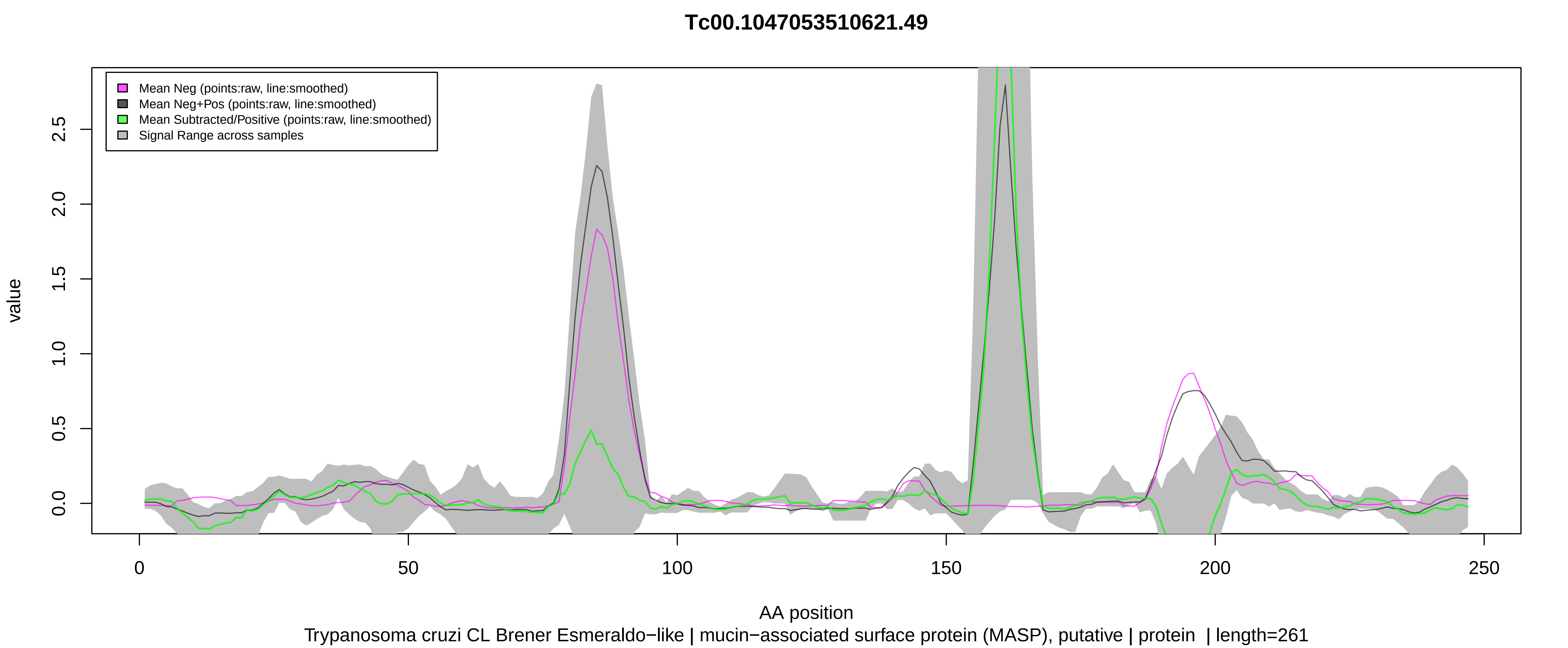
Tc00.1047053510621.49
Mean Neg (points:raw, line:smoothed)
Mean Neg+Pos (points:raw, line:smoothed)
Mean Subtracted/Positive (points:raw, line:smoothed)
Signal Range across samples
value
0.0
0.5
1.0
1.5
2.0
2.5
0
50
100
150
200
250
AA position
Trypanosoma cruzi CL Brener Esmeraldo−like | mucin−associated surface protein (MASP), putative | protein | length=261

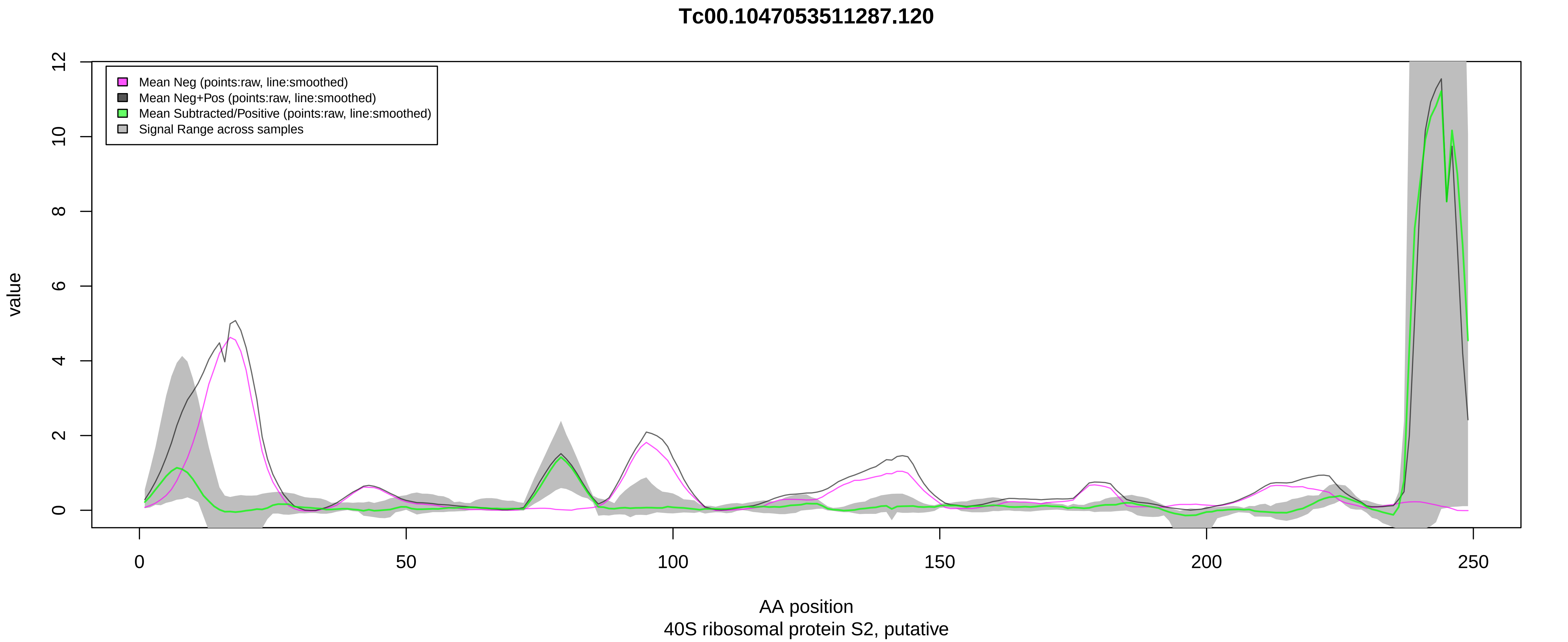

Tc00.1047053511287.120
Mean Neg (points:raw, line:smoothed)
Mean Neg+Pos (points:raw, line:smoothed)
Mean Subtracted/Positive (points:raw, line:smoothed)
Signal Range across samples
value
0
2
4
6
8
10
12
0
50
100
150
200
250
AA position
40S ribosomal protein S2, putative

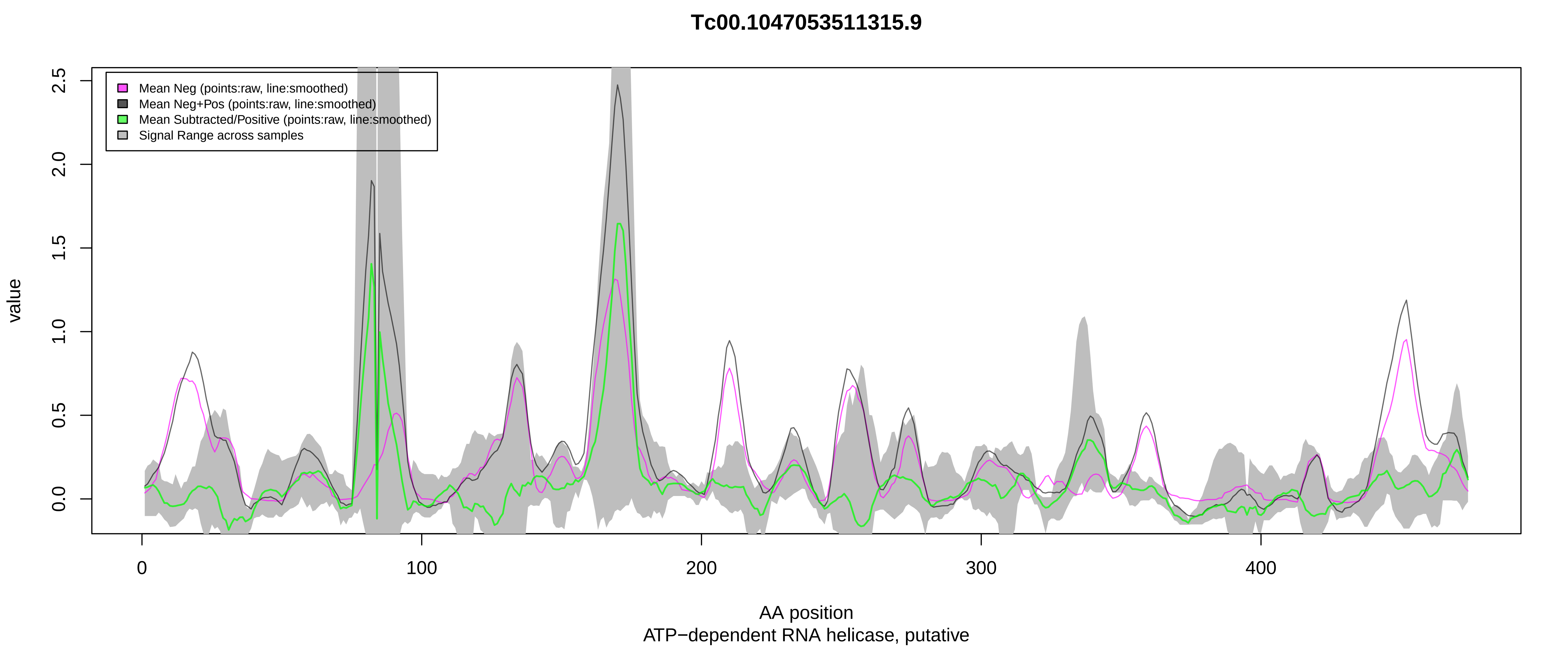

Tc00.1047053511315.9
Mean Neg (points:raw, line:smoothed)
Mean Neg+Pos (points:raw, line:smoothed)
Mean Subtracted/Positive (points:raw, line:smoothed)
Signal Range across samples
value
0.0
0.5
1.0
1.5
2.0
2.5
0
100
200
300
400
AA position
ATP−dependent RNA helicase, putative

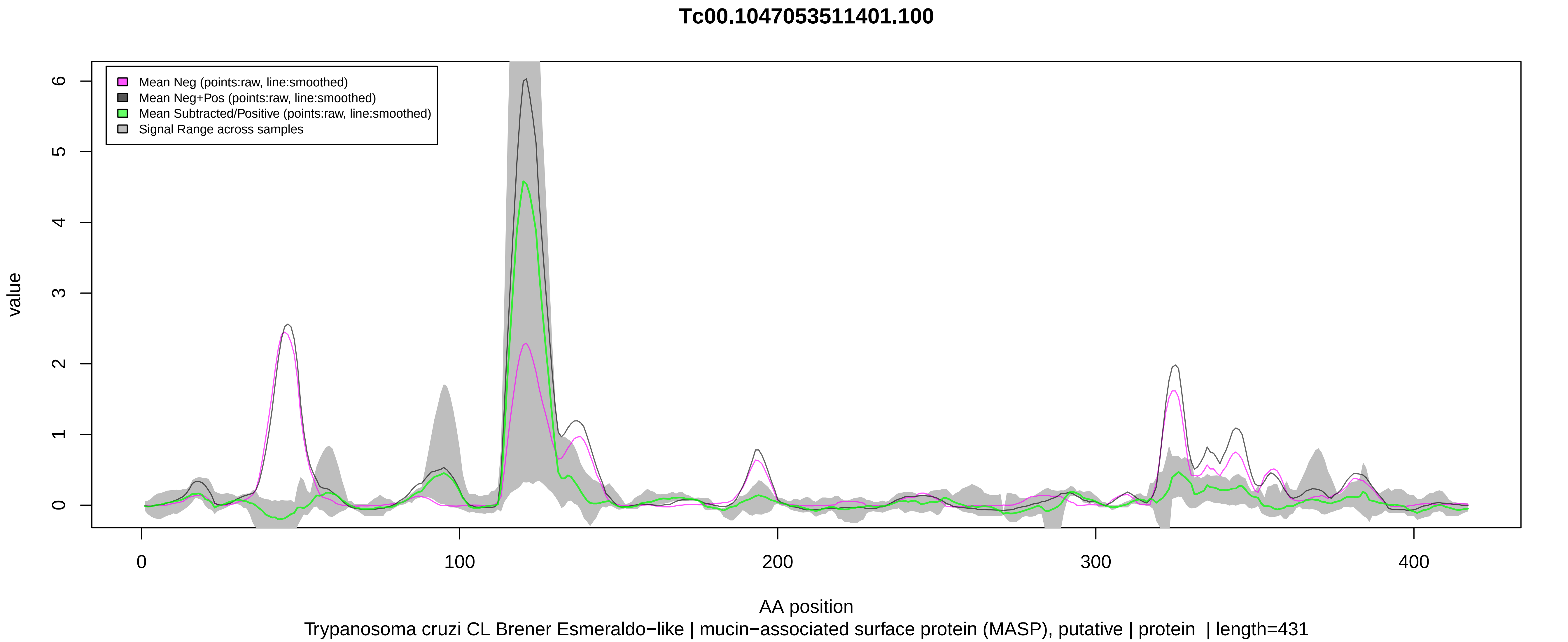
Tc00.1047053511401.100
Mean Neg (points:raw, line:smoothed)
Mean Neg+Pos (points:raw, line:smoothed)
Mean Subtracted/Positive (points:raw, line:smoothed)
Signal Range across samples
value
0
1
2
3
4
5
6
0
100
200
300
400
AA position
Trypanosoma cruzi CL Brener Esmeraldo−like | mucin−associated surface protein (MASP), putative | protein | length=431

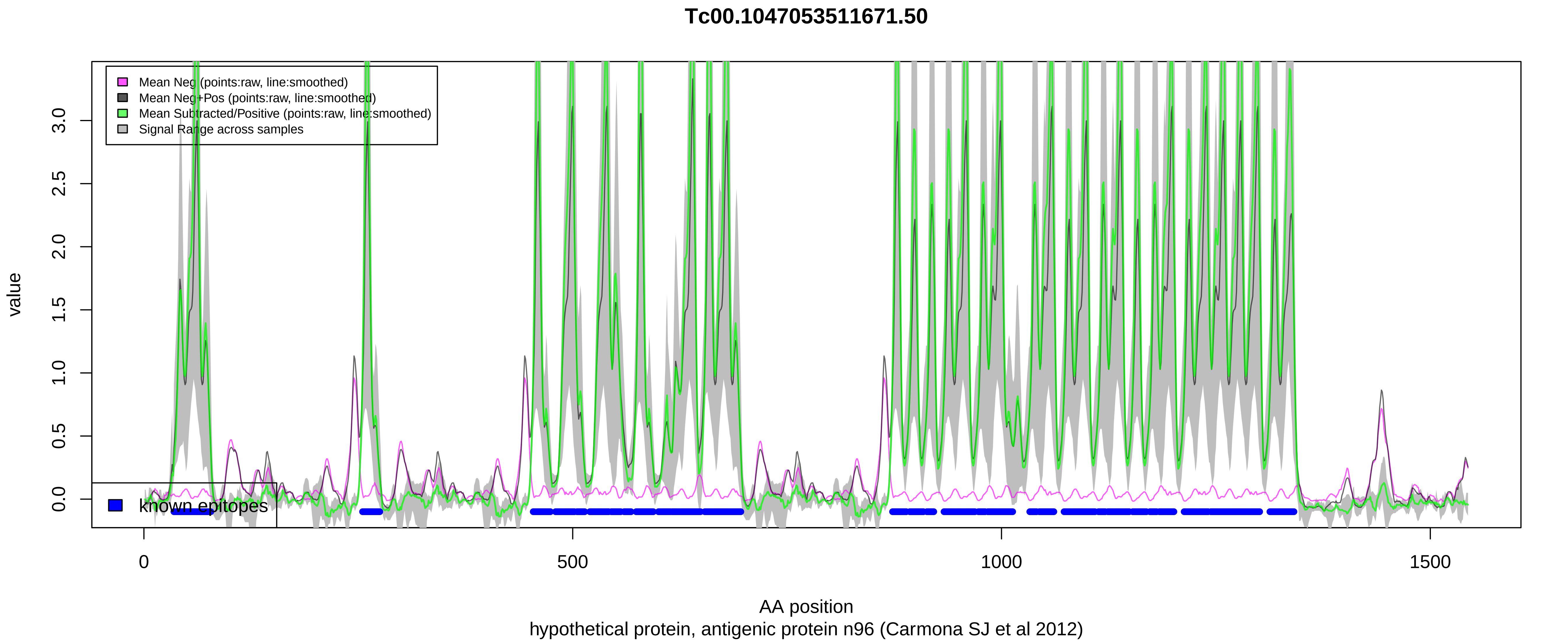
Tc00.1047053511671.50
Mean Neg (points:raw, line:smoothed)
Mean Neg+Pos (points:raw, line:smoothed)
Mean Subtracted/Positive (points:raw, line:smoothed)
Signal Range across samples
known epitopes
value
0.0
0.5
1.0
1.5
2.0
2.5
3.0
0
500
1000
1500
AA position
hypothetical protein, antigenic protein n96 (Carmona SJ et al 2012)
