# Supplementary material for: Towards High-throughput Immunomics for Infectious Diseases: Use of Next-generation Peptide Microarrays for Rapid Discovery and Mapping of Antigenic Determinants
Source: Mol Cell Proteomics. 2015 Jul;14(7):1871–84. doi: 10.1074/mcp.M114.045906 (PMC4587317; doi:10.1074/mcp.M114.045906)
Supplement: Supplemental Data [file supp_M114.045906_mcp.M114.045906-2.zip › Supplementary Figure 2 - Antigenicity Profiles of Positive Proteins/Legend for Supplementary Figure 2.docx]

**Supplementary Figure 2. Antigenicity Profiles of Positive Proteins**. The figure displays plots of normalized reactivity values along sequences for all proteins with at least one peptide above the selected cut-off (see main text). The zip file contains two PDF files: *proteinProfilesPositiveProteins.Average.pdf* containing antigenicity profiles showing the averaged signal for all chips and samples and the range of reactivity values corresponding to the 4 sera pools A, B, C and D; *proteinProfilesPositiveProteins.IndividualSamples.pdf* containing antigenicity profiles showing the antibody-binding signal obtained from each sera pool in a separate color.
