# Supplementary material for: Towards High-throughput Immunomics for Infectious Diseases: Use of Next-generation Peptide Microarrays for Rapid Discovery and Mapping of Antigenic Determinants
Source: Mol Cell Proteomics. 2015 Jul;14(7):1871–84. doi: 10.1074/mcp.M114.045906 (PMC4587317; doi:10.1074/mcp.M114.045906)
Supplement: Supplemental Data [file supp_M114.045906_mcp.M114.045906-8.doc]

**Supplementary Table 5** **– Carmona SJ et al (2015) Oligonucleotides and GST-fusion peptides used in this study.** These were used to generate the different forms of the TSSA antigen for validation in an ELISA format assay (results in Supplementary Table 1)

| **Oligonucleotide** | **Sequence (5' to 3')** ^1^ | **GST-fusion peptide generated (aa)** | | |
| --- | --- | --- | --- | --- |
| TSSA VI Ep 1 Fw | CA**GGATCC**ACAGCGAATGGTGGGTCTACTAGTTCT | | ^24^TANGGSTSSTPPSGT |  |
| TSSA VI Ep 1 Rv | CA**GAATTC**ACGTACCAGAAGGTGGGGTAGAACTAGT | |  |
| TSSA VI Ep 2 Fw | CA**GGATCC**ACTAGTTCTACCCCACCTTCTGGTACG | | ^30^TSSTPPSGTENKPAT |  |
| TSSA VI Ep 2 Rv | CA**GAATTC**ATGTAGCTGGTTTATTTTCCGTACCAGA | |  |
| TSSA VI Ep 3 Fw | CA**GGATCC**TCTGGTACGGAAAATAAACCAGCTACA | | ^36^SGTENKPATGEAPSQ |  |
| TSSA VI Ep 3 Rv | CA**GAATTC**ATTGAGATGGAGCTTCCCCTGTAGCTGG | |  |
| TSSA VI Ep 4 Fw | CA**GGATCC**CCAGCTACAGGGGAAGCTCCATCTCAA | | ^42^PATGEAPSQPGASSG |  |
| TSSA VI Ep 4 Rv | CA**GAATTC**AACCTGAAGAAGCCCCCGGTTGAGATGG | |  |
| TSSA VI Ep 5 Fw | CA**GGATCC**CCATCTCAACCGGGGGCTTCTTCAGGT | | ^48^PSQPGASSGEAEASS |  |
| TSSA VI Ep 5 Rv | CA**GAATTC**ATGAGGAGGCTTCTGCTTCACCTGAAGA | |  |

^1^ Restriction sites are indicated in bold letters.
